# Supplementary material for: Transcriptomic Profiling and Pathway Analysis of Mesenchymal Stem Cells Following Low Dose-Rate Radiation Exposure
Source: Antioxidants (Basel). 2023 Jan 21;12(2):241. doi: 10.3390/antiox12020241 (PMC9951969; doi:10.3390/antiox12020241)
Supplement: Supplementary file 1 [file antioxidants-12-00241-s001.zip › antioxidants-2144733-supplementary.pdf]

Table S1. Differentially expressed MSC genes at 1 Gy, 0.66 cGy/h

	baseMean	log2FoldChange	stat	pvalue	padj	**fold change**
LOXL4	4687.079425	2.136242786	40.21726295	0	0	4.396
GPNMB	8291.151351	-3.016596737	-67.05327882	0	0	-8.093
CTSK	2154.213244	-3.602709127	-47.47935324	0	0	-12.149
SERPINE1	135819.1676	1.348062475	35.3532081	8.95E-274	3.87E-270	2.546
ANGPTL4	4213.021645	1.930197026	35.09305544	8.60E-270	2.98E-266	3.811
IGFBP5	19405.30818	1.746457127	34.94550848	1.52E-267	4.37E-264	3.355
SCD	12628.59792	-1.672508439	-32.69006897	2.16E-234	5.35E-231	-3.188
KRT18	1716.191668	1.952796488	30.19082222	3.13E-200	6.76E-197	3.871
RP3-329A5.4	39987.43977	1.34543522	29.53666136	9.74E-192	1.87E-188	2.541
RCAN1	10504.23507	1.327301891	28.72733922	1.74E-181	3.01E-178	2.509
ITGA7	5111.158433	1.389992651	27.4158066	1.78E-165	2.80E-162	2.621
DUSP1	4187.383014	1.42135841	26.40852817	1.09E-153	1.58E-150	2.678
CCDC80	64994.19303	1.1346269	25.85397339	2.20E-147	2.92E-144	2.196
HAS2	3929.565788	1.330625006	25.40103101	2.46E-142	3.04E-139	2.515
ASAH1	6013.523083	-1.11565525	-25.0270067	3.11E-138	3.59E-135	-2.167
RGS4	16758.55977	0.983442957	24.83058162	4.19E-136	4.54E-133	1.977
INHBB	898.1846841	1.942222595	24.81461188	6.24E-136	6.35E-133	3.843
NPC2	4621.829869	-1.304702994	-24.76083788	2.37E-135	2.28E-132	-2.470
TNFRSF10D	5041.387633	1.122862205	24.59146106	1.56E-133	1.42E-130	2.178
INSIG1	2821.630952	-1.392856201	-24.57974625	2.08E-133	1.80E-130	-2.626
TPP1	5250.15046	-1.19203315	-24.48646378	2.06E-132	1.70E-129	-2.285
FTH1	104377.9983	-1.078259148	-24.39729435	1.83E-131	1.44E-128	-2.111
IER3	4400.510943	1.23065055	24.33105725	9.20E-131	6.93E-128	2.347
CYGB	1691.55426	-1.616380883	-24.27383119	3.71E-130	2.67E-127	-3.066
LAMA1	1189.788102	-1.699879541	-24.08842829	3.31E-128	2.29E-125	-3.249
SQSTM1	17066.61528	-1.054133359	-23.55420152	1.14E-122	7.57E-120	-2.076
FADS2	3727.762466	-1.347507813	-23.4315717	2.04E-121	1.31E-118	-2.545
ADAMTS1	30905.47598	0.99545186	22.97517859	8.26E-117	5.10E-114	1.994
TMEM119	842.5394988	-1.850192611	-22.85958736	1.17E-115	7.00E-113	-3.605
ERRFI1	7089.001734	1.006944458	22.82135443	2.81E-115	1.62E-112	2.010
IL6	1223.218926	1.618670157	22.33886572	1.55E-110	8.65E-108	3.071
SOD2	2610.492375	-1.251433535	-22.08529468	4.38E-108	2.37E-105	-2.381
PLOD2	37636.22046	1.033246844	21.49840748	1.61E-102	8.45E-100	2.047
PMP22	2270.792764	-1.23283832	-21.1628205	2.10E-99	1.07E-96	-2.350
IGFBP3	72876.66834	0.927458268	21.04748446	2.41E-98	1.19E-95	1.902
RP4-646B12.2	3835.165097	-1.13329328	-21.03455668	3.17E-98	1.52E-95	-2.194
CRABP2	740.1996514	1.770838009	20.99727732	6.95E-98	3.25E-95	3.413
CYR61	28567.00748	0.859424947	20.95899743	1.55E-97	7.08E-95	1.814

MSMO1	2473.062474	-1.131807156	-20.5609476	6.14E-94	2.73E-91	-2.191
TNFRSF11B	2348.893394	1.196561769	20.52360306	1.33E-93	5.74E-91	2.292
CTSD	18120.08204	-0.969805619	-20.37502595	2.79E-92	1.18E-89	-1.959
NPC1	3159.693303	-1.033187492	-20.13792263	3.43E-90	1.42E-87	-2.047
HMOX1	3061.415443	-1.131473054	-20.09010032	9.01E-90	3.63E-87	-2.191
FTHL10	3871.625737	-1.119144507	-19.71677045	1.55E-86	6.09E-84	-2.172
FADS1	2915.484461	-1.240802944	-19.51934998	7.52E-85	2.89E-82	-2.363
PROS1	1438.682367	-1.303612257	-19.50466759	1.00E-84	3.77E-82	-2.468
FTH1P7	2644.459983	-1.099713996	-19.49582877	1.19E-84	4.39E-82	-2.143
PSG5	506.5207581	2.126579168	19.44044842	3.51E-84	1.27E-81	4.367
LDHA	40069.43579	0.774212701	19.38572797	1.02E-83	3.60E-81	1.710
HMGCS1	2025.167276	-1.145445781	-19.37044716	1.37E-83	4.75E-81	-2.212
TINAGL1	2957.618912	1.08565008	19.25319353	1.33E-82	4.51E-80	2.122
GLIPR1	11907.06921	0.828657311	19.13181914	1.37E-81	4.57E-79	1.776
MME	611.3648248	-1.688282272	-19.07848482	3.81E-81	1.24E-78	-3.223
FTHL11	1776.285991	-1.132579291	-18.97339254	2.83E-80	9.07E-78	-2.193
LOX	113300.8756	0.855924539	18.84191117	3.42E-79	1.08E-76	1.810
OLFML2B	969.4270323	-1.5672192	-18.65337518	1.19E-77	3.66E-75	-2.963
EGR1	5187.443567	2.409058691	18.53705715	1.04E-76	3.15E-74	5.311
CHI3L1	4691.388297	-1.199680106	-18.45250501	4.98E-76	1.49E-73	-2.297
CTGF	81816.81119	0.760824528	18.20294451	4.89E-74	1.44E-71	1.694
MMP15	523.7760624	-1.717528737	-18.15364583	1.20E-73	3.47E-71	-3.289
CSF2RB	680.999393	1.50598052	17.99555725	2.11E-72	5.99E-70	2.840
NR4A1	507.9337001	2.099747311	17.85112313	2.83E-71	7.91E-69	4.286
FTL	54340.97093	-1.036794608	-17.78089244	9.94E-71	2.73E-68	-2.052
SLC2A1	5787.175194	0.765626324	17.77472933	1.11E-70	3.00E-68	1.700
HEXB	4684.897921	-0.816857937	-17.75645409	1.54E-70	4.09E-68	-1.762
PTGS2	507.536823	1.696290699	17.69911717	4.26E-70	1.12E-67	3.241
ARMC9	1176.285273	-1.218524355	-17.4401512	4.09E-68	1.06E-65	-2.327
PVR	4869.576686	0.771347832	17.36621935	1.49E-67	3.79E-65	1.707
RP4-681N20.1	7570.963907	-1.112299209	-17.33189952	2.70E-67	6.78E-65	-2.162
CTSA	10537.62787	-0.852883715	-17.32788971	2.90E-67	7.17E-65	-1.806
QPRT	411.8522315	-1.800292242	-17.26768441	8.24E-67	2.01E-64	-3.483
ACAN	7293.795516	-0.869702092	-17.20304364	2.52E-66	6.06E-64	-1.827
NMB	556.2937381	-1.600442944	-17.11138401	1.22E-65	2.89E-63	-3.032
MMP11	317.5036322	-1.971876608	-17.06905832	2.52E-65	5.90E-63	-3.923
BNIP3	3626.59544	0.85262856	17.04691526	3.68E-65	8.51E-63	1.806
LGALS3	2898.419024	-1.050460284	-17.04036036	4.12E-65	9.39E-63	-2.071
SLC7A8	423.5395811	-1.756334951	-16.97988027	1.16E-64	2.60E-62	-3.378
CEMIP	69452.90796	0.805632288	16.97240559	1.31E-64	2.92E-62	1.748
NEK7	18182.59498	0.735496102	16.95107406	1.89E-64	4.14E-62	1.665

MYH3	297.5295884	2.033005977	16.91878207	3.27E-64	7.08E-62	4.093
NREP	6082.589884	0.735384215	16.73451815	7.34E-63	1.57E-60	1.665
NEFM	462.1503556	1.681630433	16.67782335	1.90E-62	4.01E-60	3.208
FHL1	7813.790868	0.713381226	16.6230361	4.75E-62	9.90E-60	1.640
AK4	2996.986443	0.849979646	16.47386339	5.65E-61	1.17E-58	1.802
GPX1	2284.036407	-0.922926654	-16.46116049	6.98E-61	1.42E-58	-1.896
DNAJB4	3695.517511	0.783323748	16.44566886	9.01E-61	1.81E-58	1.721
FLNC	14386.63233	0.755938658	16.38941824	2.28E-60	4.53E-58	1.689
BDNF	2377.332849	0.865601787	16.38745528	2.35E-60	4.62E-58	1.822
TGM2	26331.76017	0.758133891	16.359413	3.73E-60	7.25E-58	1.691
CNN1	1673.746828	1.09533885	16.18775742	6.15E-59	1.18E-56	2.137
AMDHD2	691.326647	-1.3790491	-16.1822762	6.73E-59	1.28E-56	-2.601
GRN	12310.22965	-0.741331376	-16.18138355	6.82E-59	1.28E-56	-1.672
SEMA7A	8389.999225	0.743155745	16.16901061	8.34E-59	1.55E-56	1.674
ITPR3	3675.74493	-0.80613972	-16.11416218	2.03E-58	3.74E-56	-1.749
NDUFA4L2	770.6839072	1.254305873	16.11184143	2.11E-58	3.84E-56	2.386
PGK1	18035.44237	0.739990635	16.08901495	3.05E-58	5.49E-56	1.670
GNPDA1	1180.279517	-1.144247465	-16.07752218	3.67E-58	6.55E-56	-2.210
P4HA1	15585.57502	0.701850154	16.06574935	4.43E-58	7.83E-56	1.627
RP5-1102E8.2	7179.262791	0.790051593	16.01821271	9.54E-58	1.67E-55	1.729
SFRP4	650.1055055	-1.446637634	-15.93664137	3.53E-57	6.11E-55	-2.726
CSTB	3366.146983	-0.824317385	-15.92789774	4.06E-57	6.96E-55	-1.771
IDI1	1599.332389	-0.948584548	-15.9026871	6.07E-57	1.03E-54	-1.930
MAN1C1	320.6852079	-1.883039458	-15.89719466	6.63E-57	1.11E-54	-3.689
C1R	3212.896074	-0.920384747	-15.74458923	7.48E-56	1.25E-53	-1.893
SNX9	4467.589539	-0.773217109	-15.65507892	3.07E-55	5.06E-53	-1.709
RGMB	4741.753434	0.806730888	15.52836459	2.23E-54	3.64E-52	1.749
DMKN	237.5599199	-2.115331111	-15.50253892	3.33E-54	5.40E-52	-4.333
MICAL2	12326.68781	0.726298453	15.49673785	3.65E-54	5.85E-52	1.654
MYOCD	342.5635447	1.693504447	15.44937984	7.62E-54	1.21E-51	3.234
UAP1	4698.423064	0.703636717	15.40672116	1.48E-53	2.32E-51	1.629
FABP3	346.4700613	-1.66385248	-15.40141453	1.60E-53	2.50E-51	-3.169
NRG1	2709.393152	0.83334884	15.34293886	3.95E-53	6.10E-51	1.782
VEGFA	10166.4352	0.673164641	15.30905747	6.65E-53	1.02E-50	1.595
CLEC3B	910.9527179	1.139786288	15.2970918	8.00E-53	1.21E-50	2.203
PTPRF	4593.888172	0.702434571	15.22655943	2.36E-52	3.55E-50	1.627
ATP6V1B2	2824.820522	-0.786525924	-15.17021264	5.57E-52	8.31E-50	-1.725
ANKRD13A	2439.940941	0.83117012	15.04142592	3.93E-51	5.81E-49	1.779
TGFBI	186335.8353	0.610687976	15.03112564	4.59E-51	6.74E-49	1.527
BMPER	757.5802688	1.166149655	14.96436091	1.26E-50	1.83E-48	2.244
AP000892.6	3034.568504	0.769675481	14.79650545	1.54E-49	2.23E-47	1.705

CITED2	6785.195484	0.707178303	14.71361251	5.27E-49	7.54E-47	1.633
VAT1	13949.98227	-0.758472572	-14.67736931	9.00E-49	1.28E-46	-1.692
GLMP	2209.301559	-1.020168488	-14.63455845	1.69E-48	2.38E-46	-2.028
NES	1225.336027	0.956004581	14.55776299	5.21E-48	7.28E-46	1.940
ARL4C	2034.016402	-0.85086231	-14.52753421	8.11E-48	1.12E-45	-1.804
L29074.1	2572.852642	-1.127660337	-14.49623952	1.28E-47	1.76E-45	-2.185
ACAT2	510.1840836	-1.338554345	-14.49342402	1.33E-47	1.82E-45	-2.529
PHLDA1	3728.772642	-0.702736363	-14.44552861	2.67E-47	3.62E-45	-1.628
RAB27B	474.4712239	-1.475355055	-14.42151358	3.79E-47	5.09E-45	-2.781
UAP1L1	1153.312733	-0.980315942	-14.259715	3.90E-46	5.19E-44	-1.973
TPI1	14630.23805	0.731300746	14.24366149	4.91E-46	6.49E-44	1.660
FASN	5310.572602	-0.735062481	-14.22696265	6.23E-46	8.17E-44	-1.664
COL1A2	616809.7245	0.592120467	14.22172714	6.72E-46	8.74E-44	1.507
CLCN7	2270.448803	-0.796601799	-14.21755955	7.13E-46	9.21E-44	-1.737
RECK	4793.365753	0.709472466	14.18331751	1.16E-45	1.49E-43	1.635
EDN1	516.5091073	1.273280724	14.11941796	2.88E-45	3.67E-43	2.417
GYS1	2981.630146	0.701964312	14.06351203	6.36E-45	8.04E-43	1.627
LDLR	3254.635513	-0.819452904	-13.99253419	1.73E-44	2.17E-42	-1.765
HAS1	2900.364915	0.824993524	13.89956449	6.37E-44	7.94E-42	1.772
ADAMTSL1	4326.141901	0.749599227	13.89846497	6.47E-44	8.00E-42	1.681
BEX1	952.8443105	1.000777155	13.8120449	2.16E-43	2.65E-41	2.001
CYP26B1	183.0850095	1.981248206	13.7903888	2.91E-43	3.55E-41	3.948
KRTAP2-3	458.3028709	1.380856051	13.73460045	6.30E-43	7.63E-41	2.604
BNIP3P	1231.271033	0.87800924	13.71158598	8.65E-43	1.04E-40	1.838
ARSJ	2607.017905	0.783824015	13.59363382	4.37E-42	5.22E-40	1.722
ANGPTL2	1021.035652	-0.995808792	-13.47089599	2.32E-41	2.75E-39	-1.994
LIF	2993.100121	0.881859888	13.44928069	3.11E-41	3.66E-39	1.843
CDK4	2447.652512	-0.863652004	-13.43565838	3.74E-41	4.37E-39	-1.820
IFI16	2320.988081	-0.740772227	-13.43297469	3.88E-41	4.50E-39	-1.671
SMURF2	7096.989332	0.619885134	13.39624145	6.36E-41	7.34E-39	1.537
SCG2	211.8426613	-1.812050788	-13.36566303	9.60E-41	1.10E-38	-3.511
KRT7	4168.705884	0.667247518	13.35025226	1.18E-40	1.34E-38	1.588
LSS	2769.057731	-0.745034229	-13.35007155	1.18E-40	1.34E-38	-1.676
SCDP1	410.3548019	-1.562478766	-13.24737538	4.67E-40	5.25E-38	-2.954
C5orf30	1612.157989	0.80031597	13.21634591	7.06E-40	7.89E-38	1.741
GPRC5A	1843.758305	0.84518138	13.20219538	8.52E-40	9.46E-38	1.796
RRAGC	1410.002489	-0.830572954	-13.18346199	1.09E-39	1.20E-37	-1.778
MTSS1	290.986055	-1.499598117	-13.18041533	1.14E-39	1.25E-37	-2.828
DHCR24	4602.706199	-0.664002612	-13.1591541	1.51E-39	1.64E-37	-1.584
LOXL2	70098.88904	0.540240026	13.12122459	2.49E-39	2.69E-37	1.454
COL5A1	112778.7051	0.646663287	13.0851649	4.00E-39	4.30E-37	1.566

STX3	609.2677984	-1.105115887	-13.04924407	6.42E-39	6.86E-37	-2.151
GPX4	3228.420203	-0.7398049	-13.03440772	7.80E-39	8.28E-37	-1.670
PFKFB3	1267.605469	0.85433021	13.02073766	9.33E-39	9.85E-37	1.808
FRMD5	329.7429532	-1.405551189	-13.00748678	1.11E-38	1.16E-36	-2.649
OXTR	651.858793	1.098493763	13.00638776	1.13E-38	1.17E-36	2.141
CSF1	2561.623974	-0.770632964	-12.99904144	1.24E-38	1.28E-36	-1.706
JUN	3464.034775	0.642878542	12.97511571	1.69E-38	1.75E-36	1.561
C1S	5608.923547	-0.665896423	-12.93248455	2.95E-38	3.02E-36	-1.587
NEU1	1862.353213	-0.86112227	-12.9304947	3.03E-38	3.08E-36	-1.816
CTSL	5468.090275	-0.610050547	-12.8894208	5.16E-38	5.23E-36	-1.526
NDST1	14802.15284	0.506104089	12.86581536	7.01E-38	7.06E-36	1.420
PLAT	2510.753867	0.668935542	12.83073474	1.10E-37	1.10E-35	1.590
PGM1	4185.156943	0.606974996	12.81635949	1.33E-37	1.32E-35	1.523
ALPL	808.5993121	1.082111902	12.76374381	2.61E-37	2.59E-35	2.117
SERPINE2	158256.1333	0.478182621	12.74174604	3.47E-37	3.41E-35	1.393
RAB3IL1	245.5060398	-1.583319055	-12.73129945	3.96E-37	3.88E-35	-2.997
DHCR7	1832.196399	-0.846003099	-12.71128416	5.12E-37	4.98E-35	-1.798
LAMP2	8334.508865	-0.569667796	-12.70155654	5.80E-37	5.61E-35	-1.484
THBS1	269444.5996	0.581715334	12.69891612	6.00E-37	5.77E-35	1.497
DBI	2112.929533	-0.724643353	-12.68976698	6.74E-37	6.45E-35	-1.652
FILIP1L	821.6485122	0.964465028	12.58441556	2.57E-36	2.45E-34	1.951
CAV1	20605.5096	0.499006806	12.56406898	3.33E-36	3.15E-34	1.413
AXL	13001.171	0.529621477	12.55545352	3.71E-36	3.49E-34	1.444
RP11-94A24.1	211.5168988	1.684691283	12.53752283	4.65E-36	4.35E-34	3.215
ATP2B4	14019.31554	0.674961418	12.50689971	6.84E-36	6.37E-34	1.597
SLC16A3	11812.8066	0.573297119	12.48032175	9.56E-36	8.85E-34	1.488
PCSK9	167.0621936	-1.886545761	-12.46160691	1.21E-35	1.11E-33	-3.697
STAT2	2421.024644	-0.746089028	-12.44513268	1.49E-35	1.36E-33	-1.677
LHFPL2	1894.571864	-0.71667908	-12.42091324	2.01E-35	1.83E-33	-1.643
ASS1	1758.9734	0.736120038	12.40464027	2.47E-35	2.24E-33	1.666
COL1A1	803802.0585	0.553052888	12.35089387	4.82E-35	4.34E-33	1.467
FSTL1	101822.7657	0.485882875	12.32093997	6.99E-35	6.27E-33	1.400
IDH1	2074.255692	-0.719278133	-12.30735126	8.27E-35	7.38E-33	-1.646
KRT34	549.8766863	1.056954542	12.2774001	1.20E-34	1.06E-32	2.081
RAPH1	6097.838528	0.812534302	12.2713843	1.29E-34	1.14E-32	1.756
SPON2	374.6839843	-1.263592291	-12.25848384	1.51E-34	1.33E-32	-2.401
PSG2	322.020875	1.364990698	12.23420022	2.04E-34	1.78E-32	2.576
ADARB1	2623.291185	0.661844552	12.20335318	2.98E-34	2.59E-32	1.582
SYT14	627.9620384	-1.162453902	-12.18068419	3.94E-34	3.41E-32	-2.238
FDPS	1918.992829	-0.867602387	-12.16097556	5.02E-34	4.32E-32	-1.825
P3H2	3078.446878	0.63665495	12.1393209	6.54E-34	5.60E-32	1.555

NTN4	10408.1269	0.51788969	12.1339885	6.98E-34	5.95E-32	1.432
NOTCH3	14066.88015	0.604594838	12.09334304	1.15E-33	9.72E-32	1.521
RHOB	6096.659991	0.597902781	12.06812113	1.56E-33	1.31E-31	1.514
LRRC32	3785.627342	-0.606512863	-11.97205549	4.98E-33	4.18E-31	-1.523
RND3	7435.132988	-0.517642053	-11.9688299	5.18E-33	4.33E-31	-1.432
RAB31	3665.383471	-0.570788522	-11.95791294	5.90E-33	4.91E-31	-1.485
PLOD1	29474.54012	0.542259199	11.94728077	6.71E-33	5.56E-31	1.456
KCTD20	8738.592016	0.594919368	11.93869695	7.44E-33	6.13E-31	1.510
M6PR	2420.067209	-0.617671011	-11.93739922	7.55E-33	6.20E-31	-1.534
SLC1A3	316.5111926	-1.314441495	-11.93163151	8.10E-33	6.61E-31	-2.487
NEDD9	1598.641038	0.729906386	11.92305365	8.98E-33	7.30E-31	1.659
FZD7	3452.205398	0.598271635	11.85884954	1.94E-32	1.57E-30	1.514
PLEKHF1	472.1357632	-1.147612358	-11.71654573	1.05E-31	8.44E-30	-2.215
ICAM1	253.5975394	-1.40531641	-11.70606391	1.19E-31	9.51E-30	-2.649
ARHGAP29	1509.458044	0.740593582	11.66325304	1.96E-31	1.57E-29	1.671
WISP2	448.5275198	-1.125487876	-11.6478966	2.35E-31	1.87E-29	-2.182
SUGCT	669.4680128	-1.01125017	-11.64039922	2.57E-31	2.03E-29	-2.016
APP	21549.4014	-0.46231027	-11.60386181	3.94E-31	3.10E-29	-1.378
PLXNA3	4992.104829	0.517631913	11.58984235	4.64E-31	3.63E-29	1.432
CYBRD1	7753.225112	-0.604499838	-11.57769847	5.35E-31	4.17E-29	-1.520
SAMD4A	2862.347936	0.621275522	11.57510693	5.51E-31	4.28E-29	1.538
FLCN	852.6694092	-0.876254951	-11.50421458	1.26E-30	9.71E-29	-1.836
RUSC2	2885.338811	0.572883347	11.49039243	1.47E-30	1.13E-28	1.487
WNT5B	3636.732599	0.621496362	11.46802962	1.91E-30	1.46E-28	1.538
SPARC	285926.945	0.459561808	11.44910688	2.38E-30	1.81E-28	1.375
COL8A1	28255.05708	0.573780681	11.43892291	2.67E-30	2.03E-28	1.488
RAB7A	8474.609907	-0.474045118	-11.42783906	3.04E-30	2.29E-28	-1.389
HAPLN1	8891.80042	0.579889027	11.39576458	4.39E-30	3.30E-28	1.495
PTGDS	104.4761482	-2.087720642	-11.36741395	6.08E-30	4.55E-28	-4.251
PPT1	2042.790588	-0.627980525	-11.3589346	6.70E-30	5.00E-28	-1.545
CSRNP1	540.7498643	1.01286774	11.34574595	7.79E-30	5.78E-28	2.018
FDFT1	2710.950479	-0.639887085	-11.31611455	1.09E-29	8.08E-28	-1.558
HMGCR	1604.264738	-0.700981981	-11.27829141	1.68E-29	1.24E-27	-1.626
MYBL1	853.8542007	0.882037252	11.2488784	2.35E-29	1.72E-27	1.843
USP53	2899.829738	0.588433253	11.22389338	3.11E-29	2.27E-27	1.504
PTRF	46904.40569	0.413426721	11.19869188	4.14E-29	3.01E-27	1.332
SCPEP1	2897.839574	-0.625003765	-11.16059375	6.36E-29	4.60E-27	-1.542
FBXO32	1430.571183	-0.751431347	-11.14314867	7.73E-29	5.58E-27	-1.683
SLC1A1	2092.939289	0.640404356	11.13991895	8.02E-29	5.76E-27	1.559
DRAM1	2208.204647	-0.63821196	-11.13732816	8.26E-29	5.91E-27	-1.556
PFKFB4	539.0726523	1.018502428	11.12439068	9.55E-29	6.80E-27	2.026

CARS2	1042.51846	-0.781628669	-11.11890999	1.02E-28	7.20E-27	-1.719
KISS1	71.14026106	2.415649198	11.11130155	1.11E-28	7.81E-27	5.336
PENK	6416.064621	0.745092169	11.10956823	1.13E-28	7.93E-27	1.676
TXNIP	6144.938003	0.552570147	11.08690217	1.45E-28	1.02E-26	1.467
PGD	2750.417718	-0.61293231	-11.08581998	1.47E-28	1.03E-26	-1.529
FOXRED2	533.3090007	-1.002988794	-11.07568187	1.65E-28	1.14E-26	-2.004
NUPR1	5429.69941	-0.651120226	-11.07013329	1.75E-28	1.21E-26	-1.570
EVI2A	89.87546939	-2.175202548	-11.05953094	1.97E-28	1.36E-26	-4.516
RP5-857K21.6	27584.06052	-0.682369493	-11.05591087	2.05E-28	1.41E-26	-1.605
IGFBP2	27243.01832	0.583300983	11.05011107	2.19E-28	1.49E-26	1.498
P4HA2	8405.730469	0.55765071	11.05032365	2.18E-28	1.49E-26	1.472
NQO1	5930.226364	-0.49136714	-11.0444231	2.33E-28	1.58E-26	-1.406
SERINC5	1477.987758	-0.687397682	-11.03438324	2.61E-28	1.76E-26	-1.610
PRSS12	3663.874115	0.61410829	11.01400956	3.27E-28	2.20E-26	1.531
WNK4	212.5250281	1.503733009	10.99738947	3.93E-28	2.64E-26	2.836
FTHL20	464.7299049	-1.133588979	-10.98373685	4.58E-28	3.06E-26	-2.194
ERGIC1	10127.42918	0.478892342	10.95478547	6.30E-28	4.20E-26	1.394
SDCBP	4272.316366	-0.56309689	-10.95400911	6.36E-28	4.22E-26	-1.477
KIAA1644	3529.320853	-0.600910322	-10.95313647	6.42E-28	4.24E-26	-1.517
F2R	1759.930682	-0.620061183	-10.94814496	6.78E-28	4.46E-26	-1.537
ADAMTS6	583.3413084	1.007423521	10.94692125	6.87E-28	4.51E-26	2.010
NAV1	9765.205383	0.593519623	10.92017517	9.23E-28	6.03E-26	1.509
NPR3	7027.795675	0.618410261	10.91795842	9.46E-28	6.16E-26	1.535
GBA	3689.438989	-0.612710377	-10.88666127	1.33E-27	8.65E-26	-1.529
SLC40A1	98.65032012	-1.978329358	-10.87334946	1.54E-27	9.98E-26	-3.940
TSPAN10	348.4707665	-1.163959408	-10.85264483	1.94E-27	1.25E-25	-2.241
CD248	17431.46041	0.53087809	10.82904042	2.51E-27	1.61E-25	1.445
FAP	8061.338836	-0.486905951	-10.82343164	2.67E-27	1.70E-25	-1.401
SLC9A7	1898.29297	0.599326449	10.82100184	2.74E-27	1.74E-25	1.515
KLF6	4541.875886	0.513852186	10.81190959	3.02E-27	1.91E-25	1.428
COL5A2	40187.78455	0.489772225	10.81171758	3.03E-27	1.91E-25	1.404
EPHX1	1972.752465	-0.63854092	-10.79829421	3.51E-27	2.21E-25	-1.557
SDC4	3232.72661	0.585409805	10.77868061	4.34E-27	2.72E-25	1.500
CKAP4	23095.28599	0.428052455	10.74664486	6.15E-27	3.84E-25	1.345
MASP1	251.8086378	1.276403248	10.72832523	7.49E-27	4.67E-25	2.422
PDE1C	4184.482022	0.525974348	10.71174995	8.97E-27	5.56E-25	1.440
TNS3	1985.92503	-0.753024913	-10.66847253	1.43E-26	8.84E-25	-1.685
SULF2	727.6265102	-0.864041067	-10.66304608	1.52E-26	9.34E-25	-1.820
TM4SF1	3760.37961	0.627735827	10.57148798	4.04E-26	2.48E-24	1.545
TKT	4089.51845	-0.514775532	-10.54938217	5.11E-26	3.13E-24	-1.429
FOXD1	1633.78384	0.690378363	10.54016274	5.64E-26	3.44E-24	1.614

SDC1	524.3407508	-0.924853568	-10.53817266	5.76E-26	3.50E-24	-1.898
ARL8B	2733.091028	-0.531276596	-10.53756452	5.80E-26	3.51E-24	-1.445
CTSB	40711.44705	-0.458096361	-10.52120684	6.90E-26	4.16E-24	-1.374
FER1L4	245.3884708	1.329602879	10.52068412	6.94E-26	4.17E-24	2.513
GDF15	2395.71471	-0.615639956	-10.51852649	7.10E-26	4.25E-24	-1.532
ADAMTS12	2947.06965	0.644434575	10.51698738	7.21E-26	4.31E-24	1.563
BNIP3L	7604.682616	0.481701671	10.4944115	9.16E-26	5.45E-24	1.396
PCOLCE2	1084.270036	0.712298924	10.47895188	1.08E-25	6.40E-24	1.638
SERPING1	1996.46272	0.59999678	10.45492806	1.39E-25	8.22E-24	1.516
GNPTAB	2298.123771	-0.608534944	-10.44754242	1.50E-25	8.85E-24	-1.525
POSTN	107260.8591	-0.588510561	-10.43770806	1.67E-25	9.79E-24	-1.504
ROR1	1880.394289	0.654752088	10.39542324	2.60E-25	1.52E-23	1.574
MYADM	7769.167297	0.489694815	10.38505428	2.90E-25	1.69E-23	1.404
BST1	1571.457571	0.631163199	10.37871847	3.10E-25	1.80E-23	1.549
CRISPLD2	1760.34102	-0.672219311	-10.37252362	3.31E-25	1.91E-23	-1.594
POFUT2	5367.273385	0.493668067	10.34695199	4.32E-25	2.49E-23	1.408
BOD1	1192.510199	-0.661479175	-10.328977	5.21E-25	3.00E-23	-1.582
THBS3	5823.774533	-0.468928343	-10.32153635	5.63E-25	3.23E-23	-1.384
FOXQ1	112.5985554	-1.759026714	-10.31076991	6.30E-25	3.60E-23	-3.385
ECE1	7281.567397	0.434304071	10.28114993	8.57E-25	4.88E-23	1.351
TENM2	510.7210821	1.08820733	10.24052001	1.31E-24	7.41E-23	2.126
SMOC1	886.2632394	-0.901493289	-10.23852477	1.33E-24	7.54E-23	-1.868
LITAF	2726.908553	-0.525008479	-10.23602299	1.37E-24	7.71E-23	-1.439
ENC1	5170.447341	0.519495926	10.21559244	1.69E-24	9.49E-23	1.433
RAB3B	3146.33772	0.647984776	10.21245223	1.74E-24	9.77E-23	1.567
MT-ND3	3658.828841	-0.68221608	-10.19094722	2.18E-24	1.22E-22	-1.605
DSP	14176.77921	0.59526887	10.18069404	2.42E-24	1.35E-22	1.511
C10orf10	1665.066522	-0.602779706	-10.17884976	2.46E-24	1.37E-22	-1.519
WASHC5	2127.345963	-0.570962198	-10.16576238	2.82E-24	1.56E-22	-1.486
PEPD	1592.091203	-0.66878487	-10.16132538	2.95E-24	1.63E-22	-1.590
LMCD1	1007.726971	0.744292073	10.15966018	3.00E-24	1.65E-22	1.675
SPG21	1624.562394	-0.614664147	-10.13050531	4.05E-24	2.22E-22	-1.531
SQLE	1905.60033	-0.606116797	-10.12982951	4.07E-24	2.22E-22	-1.522
PLPPR2	2320.707053	0.58381705	10.08916792	6.17E-24	3.36E-22	1.499
GNS	12201.37891	-0.494111495	-10.0786443	6.87E-24	3.73E-22	-1.408
RP1-140K8.5	287.8835931	1.166155237	10.07742546	6.95E-24	3.76E-22	2.244
TMEM140	233.1789494	-1.304370423	-10.07695917	6.99E-24	3.77E-22	-2.470
EHD1	4327.842145	0.458834289	10.02019658	1.24E-23	6.68E-22	1.374
CYP51A1	1694.431375	-0.591536592	-10.01051418	1.37E-23	7.34E-22	-1.507
ACSF2	945.1280023	-0.753957987	-9.997202841	1.57E-23	8.38E-22	-1.686
RENBP	61.44566984	-2.219759866	-9.993853262	1.62E-23	8.64E-22	-4.658

CTNS	584.9839985	-0.85802546	-9.988470414	1.71E-23	9.09E-22	-1.813
OGFRL1	1779.621418	0.619418715	9.949285367	2.54E-23	1.34E-21	1.536
ADAM19	1847.604572	0.590936485	9.900370256	4.15E-23	2.19E-21	1.506
CREB5	253.2038231	1.167410694	9.894469306	4.40E-23	2.31E-21	2.246
IER2	1178.154992	0.741509023	9.88110007	5.03E-23	2.64E-21	1.672
LAMP1	17638.55277	-0.423002493	-9.874739219	5.36E-23	2.80E-21	-1.341
HBEGF	696.6918713	0.781948831	9.874407355	5.37E-23	2.80E-21	1.719
PCYT2	1009.478378	-0.664952314	-9.864842751	5.91E-23	3.07E-21	-1.586
LMOD1	2259.870657	0.527149723	9.858149285	6.32E-23	3.28E-21	1.441
FLG	7004.090388	1.306711805	9.832657883	8.14E-23	4.21E-21	2.474
LMAN1	10980.56548	0.525324763	9.819794677	9.25E-23	4.77E-21	1.439
HS1BP3	717.763836	-0.806917986	-9.818694697	9.35E-23	4.81E-21	-1.749
FKBP10	21016.33162	0.41445586	9.814672225	9.74E-23	4.99E-21	1.333
IL11	337.8987315	1.066639454	9.802797041	1.10E-22	5.59E-21	2.095
MCFD2	15603.84611	0.393317642	9.798921527	1.14E-22	5.79E-21	1.313
CACNA2D1	6647.064563	0.577546451	9.795150331	1.18E-22	6.00E-21	1.492
STC1	671.7513828	0.887851705	9.790806681	1.23E-22	6.24E-21	1.850
AFAP1	7254.212355	0.555540595	9.772572555	1.48E-22	7.45E-21	1.470
DCLK1	136.981007	-1.522873198	-9.759842057	1.67E-22	8.43E-21	-2.874
CLCF1	631.5355816	0.817242765	9.753210007	1.79E-22	8.97E-21	1.762
MVD	941.4141251	-0.765045753	-9.748649326	1.87E-22	9.35E-21	-1.699
GJA5	77.93681976	1.886906341	9.747315397	1.89E-22	9.45E-21	3.698
VLDLR	1209.438025	0.716906082	9.742653921	1.98E-22	9.87E-21	1.644
FLNB	14498.75391	0.486679032	9.740724129	2.02E-22	1.00E-20	1.401
RP11-16M8.2	741.3095564	0.738344848	9.734632727	2.15E-22	1.06E-20	1.668
BRI3	2629.719146	-0.607718677	-9.722657149	2.41E-22	1.19E-20	-1.524
RAB29	721.0748965	-0.767519773	-9.722469645	2.42E-22	1.19E-20	-1.702
KCNK6	1679.243155	0.585497031	9.716519569	2.56E-22	1.26E-20	1.501
PRRX1	5083.447819	-0.478957266	-9.689488906	3.34E-22	1.63E-20	-1.394
RALA	2209.712361	-0.541005667	-9.685987755	3.46E-22	1.69E-20	-1.455
GPT2	1398.046854	0.608406484	9.673229995	3.92E-22	1.91E-20	1.525
RP11-54D18.1	4031.496956	0.644637382	9.669473932	4.06E-22	1.97E-20	1.563
FSCN1	4977.72282	0.452236798	9.658223417	4.54E-22	2.19E-20	1.368
TBX3	1149.14361	-0.672849538	-9.655923993	4.64E-22	2.24E-20	-1.594
ATP6V1A	2604.595154	-0.502600881	-9.653798413	4.74E-22	2.28E-20	-1.417
RP11-516A11.1	2089.094463	0.583355424	9.63927997	5.46E-22	2.62E-20	1.498
ZNF395	1254.846965	0.61465106	9.634301943	5.73E-22	2.74E-20	1.531
PDE5A	3876.891098	0.604414732	9.611766297	7.13E-22	3.40E-20	1.520
ATP10A	2159.767796	0.555843253	9.610669466	7.21E-22	3.43E-20	1.470
C14orf1	780.1379951	-0.797949639	-9.578712282	9.83E-22	4.66E-20	-1.739
AMOTL2	6594.497185	0.448967585	9.576340605	1.01E-21	4.76E-20	1.365

RP5-864K19.4	103.2285187	-1.657628764	-9.561562827	1.16E-21	5.47E-20	-3.155
COL3A1	121161.6986	0.41524275	9.559707384	1.18E-21	5.56E-20	1.334
LAMB2	20110.95709	0.371089018	9.544247418	1.37E-21	6.43E-20	1.293
C14orf159	360.3880744	-0.966315555	-9.534969401	1.50E-21	7.02E-20	-1.954
LY96	456.3111793	-0.938951318	-9.526471788	1.63E-21	7.59E-20	-1.917
KDSR	1746.837364	-0.554946504	-9.498497544	2.13E-21	9.91E-20	-1.469
ATP6V0E1	8156.715385	-0.482232782	-9.487932709	2.36E-21	1.09E-19	-1.397
ALDOA	5924.379721	0.435351394	9.486072646	2.40E-21	1.11E-19	1.352
SRPX	916.9498665	-0.701667517	-9.483627124	2.46E-21	1.13E-19	-1.626
PDCD1LG2	1224.14787	0.634538575	9.476409217	2.63E-21	1.21E-19	1.552
ANKRD1	9174.008444	0.399311265	9.468695351	2.83E-21	1.30E-19	1.319
OLFML3	9318.534382	-0.442851263	-9.464334136	2.95E-21	1.35E-19	-1.359
FMNL2	841.7855046	-0.741777008	-9.449784752	3.40E-21	1.55E-19	-1.672
CAV2	2814.091206	0.493944677	9.447986481	3.45E-21	1.57E-19	1.408
PPP1R3B	1219.475749	-0.659083986	-9.441972284	3.66E-21	1.66E-19	-1.579
CDC25B	1216.917708	-0.711019507	-9.439426902	3.75E-21	1.70E-19	-1.637
CRIM1	27132.11255	0.43249742	9.43125893	4.05E-21	1.83E-19	1.350
TNC	26628.72731	0.500405494	9.405481191	5.18E-21	2.33E-19	1.415
ABCC4	878.689665	-0.74153353	-9.40253154	5.33E-21	2.40E-19	-1.672
AKR1C1	575.0128977	-0.875825057	-9.377113464	6.78E-21	3.04E-19	-1.835
EGR3	174.7611002	1.296758518	9.375454347	6.89E-21	3.08E-19	2.457
DVL2	773.4831475	-0.801713909	-9.372357062	7.09E-21	3.16E-19	-1.743
SLC38A6	361.732077	-0.955585683	-9.365699652	7.55E-21	3.36E-19	-1.939
NMT2	1260.02433	0.584751217	9.358750431	8.07E-21	3.58E-19	1.500
NETO2	1029.318042	0.65716832	9.356408298	8.25E-21	3.65E-19	1.577
CHIC2	993.0376221	0.638193172	9.353519563	8.48E-21	3.74E-19	1.556
TNFRSF21	771.8772234	0.702256554	9.329708917	1.06E-20	4.68E-19	1.627
NIPAL3	2163.782803	0.516840675	9.318131148	1.18E-20	5.20E-19	1.431
CTD-2324A24.1	10651.88305	0.402769677	9.283640622	1.64E-20	7.18E-19	1.322
SMYD3	700.7588439	-0.727315802	-9.283163342	1.65E-20	7.19E-19	-1.656
FOLR3	81.08709409	-1.814651892	-9.273212291	1.81E-20	7.88E-19	-3.518
DSTN	24665.38403	-0.380775347	-9.266554428	1.92E-20	8.36E-19	-1.302
CLTA	4352.399784	-0.486517068	-9.265744444	1.94E-20	8.41E-19	-1.401
SNX8	1162.507775	-0.65080881	-9.257389387	2.09E-20	9.07E-19	-1.570
QPCT	575.5815587	0.784564243	9.250094329	2.24E-20	9.68E-19	1.723
GAPDH	104310.2308	0.482846828	9.240364279	2.46E-20	1.06E-18	1.397
SNX2	2028.496906	-0.509241829	-9.221258929	2.94E-20	1.26E-18	-1.423
ATP6V1C1	1850.22419	-0.522412953	-9.195376773	3.74E-20	1.60E-18	-1.436
MMAB	387.8771002	-0.911563688	-9.193009767	3.82E-20	1.63E-18	-1.881
BTN2A1	1154.338377	0.597595033	9.186200661	4.07E-20	1.74E-18	1.513
DLC1	5451.331213	0.445025275	9.169383515	4.76E-20	2.02E-18	1.361

LPIN1	1696.271258	-0.584247043	-9.165147457	4.95E-20	2.10E-18	-1.499
COL4A3BP	1496.877648	-0.590502212	-9.156474537	5.36E-20	2.27E-18	-1.506
QSOX1	19062.8276	0.456603096	9.149425912	5.72E-20	2.42E-18	1.372
ANKRD37	734.6643744	0.704065648	9.132143554	6.72E-20	2.83E-18	1.629
RHOQ	4323.80057	-0.418576639	-9.127026862	7.04E-20	2.96E-18	-1.337
OXCT1	1249.951223	-0.607116565	-9.125601089	7.13E-20	2.99E-18	-1.523
VAC14	840.3206189	-0.705875783	-9.125431496	7.15E-20	2.99E-18	-1.631
DDIT4	2985.178029	-0.538180879	-9.1181555	7.64E-20	3.19E-18	-1.452
CREG1	2467.493938	-0.488590086	-9.117687031	7.67E-20	3.19E-18	-1.403
NFASC	6134.891314	0.433444709	9.114249054	7.92E-20	3.29E-18	1.350
SULF1	22632.53946	-0.606114391	-9.108428959	8.36E-20	3.46E-18	-1.522
RRAS2	2097.324711	0.4991279	9.103313457	8.76E-20	3.62E-18	1.413
CTNNAL1	2582.449432	0.467198856	9.079486036	1.09E-19	4.50E-18	1.382
PDK1	775.4766086	0.708830708	9.071445737	1.17E-19	4.83E-18	1.634
ZHX3	2340.464981	0.491827377	9.065980276	1.23E-19	5.07E-18	1.406
SIRPA	3606.81882	0.451713272	9.047204381	1.47E-19	6.00E-18	1.368
ORMDL3	2158.658497	0.561384001	9.03347082	1.66E-19	6.79E-18	1.476
TPST1	1275.743237	-0.56126944	-9.026125985	1.78E-19	7.24E-18	-1.476
ACAT1	1515.244801	-0.589345165	-9.012496487	2.01E-19	8.19E-18	-1.505
LRRC17	260.5168358	1.050358271	8.997873541	2.30E-19	9.33E-18	2.071
AOX1	938.0738133	0.660943351	8.993612839	2.39E-19	9.68E-18	1.581
SLC8A1	618.0496597	0.772538528	8.987115207	2.54E-19	1.02E-17	1.708
FGF1	618.0210917	0.731611383	8.977102271	2.78E-19	1.12E-17	1.660
SC5D	1942.87842	-0.592479417	-8.967818388	3.02E-19	1.21E-17	-1.508
IFIT3	193.4219588	-1.308927821	-8.961732895	3.20E-19	1.28E-17	-2.478
SCARB1	836.643306	-0.705832876	-8.960074822	3.24E-19	1.30E-17	-1.631
SLC20A1	8513.670897	0.373260529	8.94420098	3.75E-19	1.49E-17	1.295
CERCAM	10490.69628	0.403384832	8.917856907	4.75E-19	1.89E-17	1.323
TNS1	9558.38037	0.436307897	8.910829683	5.07E-19	2.01E-17	1.353
EMC1	4898.821918	0.402884177	8.909524555	5.13E-19	2.03E-17	1.322
IRAK1	4158.015713	-0.435137007	-8.884072827	6.45E-19	2.55E-17	-1.352
VEGFB	1941.50265	-0.580167402	-8.875615007	6.95E-19	2.74E-17	-1.495
TP53I11	5037.68949	0.434258651	8.87215274	7.17E-19	2.82E-17	1.351
ERP29	3521.013856	-0.485654079	-8.847622182	8.94E-19	3.51E-17	-1.400
OSGIN1	218.866335	-1.118249152	-8.834608876	1.00E-18	3.93E-17	-2.171
DUSP6	736.9496898	0.706072154	8.833932202	1.01E-18	3.95E-17	1.631
SREBF2	3898.15547	-0.441424111	-8.810562066	1.25E-18	4.86E-17	-1.358
MTHFD1	1072.486559	-0.59404991	-8.806205438	1.29E-18	5.04E-17	-1.509
GM2A	1679.044067	-0.537371586	-8.8027586	1.33E-18	5.18E-17	-1.451
PHLDB2	2165.145799	0.527737575	8.802067179	1.34E-18	5.20E-17	1.442
SRGN	12646.68287	0.43685809	8.79936362	1.38E-18	5.32E-17	1.354

KCTD11	1392.486824	0.539864863	8.794884439	1.43E-18	5.52E-17	1.454
GNG12	13803.71418	0.427701827	8.788649823	1.51E-18	5.82E-17	1.345
USP35	849.3174258	-0.686719063	-8.764587532	1.87E-18	7.20E-17	-1.610
HSP90B1	45713.25559	0.339537359	8.753128824	2.08E-18	7.91E-17	1.265
EPAS1	20875.94574	-0.386774103	-8.753108536	2.08E-18	7.91E-17	-1.307
AKR1C3	806.1361269	-0.700356592	-8.753383172	2.07E-18	7.91E-17	-1.625
CPA4	2736.204046	0.473851767	8.752643362	2.08E-18	7.93E-17	1.389
SRPX2	1914.188836	0.49848782	8.739205861	2.35E-18	8.91E-17	1.413
NTRK2	1140.593322	-0.698396059	-8.729549514	2.56E-18	9.69E-17	-1.623
LDHAP7	1140.498436	0.692336358	8.726970132	2.62E-18	9.89E-17	1.616
P4HB	61636.67751	0.361081574	8.720361941	2.77E-18	1.05E-16	1.284
SYT11	2873.347317	0.456542143	8.709138047	3.06E-18	1.15E-16	1.372
DUSP2	328.9521328	0.954624089	8.707373331	3.11E-18	1.17E-16	1.938
C6orf1	609.4338951	-0.763019144	-8.703770598	3.21E-18	1.20E-16	-1.697
NDRG1	4267.801667	0.415218506	8.702644361	3.24E-18	1.21E-16	1.334
SH3RF1	2261.17987	0.489435535	8.695287245	3.46E-18	1.29E-16	1.404
LOXL1	12757.90788	0.393346444	8.685130211	3.78E-18	1.41E-16	1.313
SBSN	168.9822088	-1.223802544	-8.676166305	4.09E-18	1.52E-16	-2.336
SH3D21	151.6607713	1.284731496	8.666955296	4.44E-18	1.65E-16	2.436
GSR	1369.10401	-0.538647057	-8.664018407	4.55E-18	1.68E-16	-1.453
SMTN	2786.019653	0.451349274	8.645794606	5.34E-18	1.97E-16	1.367
DAB2	8885.357377	0.371797575	8.645077761	5.38E-18	1.98E-16	1.294
WDR81	1043.021809	-0.605035671	-8.642499068	5.50E-18	2.02E-16	-1.521
PNPLA2	2606.093637	0.46276494	8.640883889	5.58E-18	2.05E-16	1.378
SLC25A4	1504.350866	0.548574804	8.626029095	6.35E-18	2.32E-16	1.463
VGLL3	7126.698358	0.548411012	8.609810401	7.32E-18	2.67E-16	1.462
KRT16	228.4814553	1.048552973	8.584922762	9.09E-18	3.31E-16	2.068
CSRP1	16934.13494	0.403210956	8.578275309	9.63E-18	3.50E-16	1.322
FNIP1	1023.563218	-0.648414745	-8.573181557	1.01E-17	3.65E-16	-1.567
CAPG	759.3323456	-0.720738849	-8.571199821	1.02E-17	3.71E-16	-1.648
PRKCA	4112.773385	0.443522953	8.57040311	1.03E-17	3.73E-16	1.360
EPDR1	2579.621558	-0.437930262	-8.558915346	1.14E-17	4.11E-16	-1.355
CRISPLD1	221.0014926	-1.067207834	-8.558282565	1.15E-17	4.12E-16	-2.095
PPFIA4	594.2976621	0.737824646	8.552273949	1.21E-17	4.33E-16	1.668
MYPN	808.3779594	0.646070351	8.54660803	1.27E-17	4.54E-16	1.565
MIR210HG	372.6685873	0.891525706	8.545569591	1.28E-17	4.57E-16	1.855
CYB561A3	1946.263936	-0.537510571	-8.531801181	1.44E-17	5.14E-16	-1.451
FBXL5	2012.66262	0.478186875	8.515710362	1.66E-17	5.90E-16	1.393
C6orf132	486.4736002	0.776433286	8.509070828	1.75E-17	6.22E-16	1.713
CD276	8433.62971	-0.376216303	-8.509003417	1.75E-17	6.22E-16	-1.298
P3H4	3585.187132	0.437380413	8.503918992	1.83E-17	6.49E-16	1.354

PHYH	574.4931591	-0.737123777	-8.502139369	1.86E-17	6.57E-16	-1.667
SQRDL	425.936026	-0.845520259	-8.501923721	1.86E-17	6.57E-16	-1.797
FOSL2	4314.060923	0.412443412	8.499794445	1.90E-17	6.68E-16	1.331
NRXN3	307.3072372	0.951230819	8.461775054	2.63E-17	9.25E-16	1.934
TET3	503.1923467	0.808711595	8.456596095	2.75E-17	9.65E-16	1.752
ADCY7	2021.044888	0.481835656	8.455345902	2.78E-17	9.73E-16	1.397
ARRDC4	1238.047451	0.547865133	8.432206629	3.39E-17	1.18E-15	1.462
MYCBP	181.9833907	1.115636727	8.411317453	4.05E-17	1.41E-15	2.167
IRS2	846.2499956	-0.653703525	-8.399869909	4.47E-17	1.55E-15	-1.573
EPHA2	1469.8696	0.510983546	8.398193993	4.53E-17	1.57E-15	1.425
VPS41	2877.671609	-0.444686872	-8.381089785	5.24E-17	1.82E-15	-1.361
FAM162A	768.5169653	0.633183697	8.378272435	5.37E-17	1.86E-15	1.551
ABL1	6390.958819	0.392156648	8.37783095	5.39E-17	1.86E-15	1.312
RP11-47I22.3	269.0454506	0.971984812	8.356221622	6.48E-17	2.23E-15	1.962
MT-ATP8	5758.667109	-0.477411133	-8.351897503	6.72E-17	2.31E-15	-1.392
NR4A2	189.6726404	1.132863827	8.348617102	6.91E-17	2.37E-15	2.193
C5orf46	177.2686135	1.142948506	8.345597478	7.09E-17	2.42E-15	2.208
CLEC11A	2131.68122	0.506354948	8.342488965	7.27E-17	2.48E-15	1.420
ACADVL	8136.258602	0.445678933	8.339851583	7.44E-17	2.53E-15	1.362
KIF26B	159.3193654	-1.211760631	-8.331006981	8.02E-17	2.73E-15	-2.316
PICALM	10630.19847	0.354299267	8.324283456	8.48E-17	2.88E-15	1.278
PPME1	2108.780343	0.447018488	8.322230907	8.63E-17	2.92E-15	1.363
KCTD9	1243.933942	0.549552143	8.318958939	8.87E-17	3.00E-15	1.464
SPG20	2106.653308	-0.462832332	-8.308501247	9.69E-17	3.27E-15	-1.378
BASP1	6043.774885	-0.39582571	-8.296189922	1.08E-16	3.62E-15	-1.316
RIMS1	664.3614414	0.664161518	8.294639002	1.09E-16	3.66E-15	1.585
GDI1	4149.79002	0.425085522	8.288318834	1.15E-16	3.85E-15	1.343
TMEM47	5478.185103	0.42200769	8.283738655	1.19E-16	4.00E-15	1.340
CRTAP	10244.74474	-0.343526014	-8.271490594	1.32E-16	4.42E-15	-1.269
PRELP	649.5539339	-0.683457883	-8.232723201	1.83E-16	6.10E-15	-1.606
PRICKLE1	668.2668691	0.675364153	8.229064204	1.89E-16	6.28E-15	1.597
ITGA5	36307.52344	0.378232317	8.228405263	1.90E-16	6.30E-15	1.300
KIRREL3	452.6620045	0.803459626	8.225678421	1.94E-16	6.44E-15	1.745
SVEP1	1657.190778	0.688309904	8.211695436	2.18E-16	7.22E-15	1.611
KCTD16	616.7266302	0.740708657	8.208174315	2.25E-16	7.42E-15	1.671
STEAP3	1382.634834	-0.55062202	-8.207179502	2.26E-16	7.47E-15	-1.465
TUFT1	871.9278395	0.627098317	8.185133256	2.72E-16	8.95E-15	1.544
TIMP1	35102.31093	0.518073724	8.18237766	2.78E-16	9.14E-15	1.432
CYP1B1	23997.80315	-0.449363683	-8.171606671	3.04E-16	9.98E-15	-1.365
PTPRQ	198.9549633	1.073401676	8.170468462	3.07E-16	1.01E-14	2.104
FHL2	13178.93761	0.389327927	8.170264831	3.08E-16	1.01E-14	1.310

METTL7A	118.6910792	-1.353093072	-8.168330815	3.13E-16	1.02E-14	-2.555
ID2	1559.794399	-0.502813978	-8.159865045	3.35E-16	1.09E-14	-1.417
RARRES2	166.4207449	-1.226793046	-8.153112429	3.55E-16	1.15E-14	-2.340
COL7A1	1133.084268	0.564388882	8.142754601	3.86E-16	1.25E-14	1.479
PNMA2	631.9217608	0.679578623	8.134673549	4.13E-16	1.34E-14	1.602
SHROOM3	375.6672604	0.840205194	8.111879898	4.98E-16	1.61E-14	1.790
H1F0	1833.554784	0.479192188	8.108562925	5.12E-16	1.65E-14	1.394
TMEM30A	7424.617395	0.394184302	8.09529914	5.71E-16	1.84E-14	1.314
OAS2	178.9609031	-1.137698662	-8.092785666	5.83E-16	1.87E-14	-2.200
MEDAG	1870.763712	0.485770899	8.088546077	6.04E-16	1.94E-14	1.400
ARID5B	3606.799884	0.394022943	8.07218447	6.91E-16	2.21E-14	1.314
GPR176	3786.459215	0.407926242	8.063757372	7.40E-16	2.36E-14	1.327
CDH2	10110.8148	0.381943264	8.058209401	7.74E-16	2.47E-14	1.303
SLC25A6	9464.424061	-0.32575125	-8.056990761	7.82E-16	2.49E-14	-1.253
ERO1A	3742.426413	0.391346953	8.047588075	8.44E-16	2.68E-14	1.312
PLEKHM1	1130.559685	-0.536366388	-8.045803453	8.57E-16	2.72E-14	-1.450
ADAM9	21926.3599	0.377937505	8.043922472	8.70E-16	2.75E-14	1.299
CRLF1	1932.442297	-0.452815717	-8.043471083	8.73E-16	2.76E-14	-1.369
LIMK1	2687.98358	-0.426169903	-8.041241154	8.89E-16	2.80E-14	-1.344
PDLIM1	1134.367265	0.543219021	8.036674728	9.23E-16	2.91E-14	1.457
LYST	928.1674377	-0.716226531	-8.035907503	9.29E-16	2.92E-14	-1.643
LIMCH1	1605.93924	0.513600584	8.03435622	9.41E-16	2.95E-14	1.428
MMP14	35401.27138	-0.346847466	-8.027389209	9.96E-16	3.12E-14	-1.272
NAV2	595.778449	-0.736056271	-8.026251964	1.00E-15	3.14E-14	-1.666
SCARF2	2673.363745	0.431260627	8.019351308	1.06E-15	3.32E-14	1.348
ERMN	306.57919	0.896146888	8.017630238	1.08E-15	3.36E-14	1.861
BMF	99.02184625	-1.374270788	-8.000482142	1.24E-15	3.85E-14	-2.592
PGK1P2	1032.286727	0.576499958	7.988235069	1.37E-15	4.25E-14	1.491
ZCCHC14	1103.295235	-0.550942161	-7.982902444	1.43E-15	4.43E-14	-1.465
DNAJC5	3283.749099	0.398043514	7.978185261	1.49E-15	4.59E-14	1.318
DBNDD1	166.1236692	1.117596459	7.973713607	1.54E-15	4.75E-14	2.170
NLGN2	2363.813974	0.430044698	7.967570199	1.62E-15	4.98E-14	1.347
MT-RNR2	115701.5586	-0.412569699	-7.967401383	1.62E-15	4.98E-14	-1.331
PSAP	44356.19241	-0.403980657	-7.962457486	1.69E-15	5.18E-14	-1.323
GBE1	5352.855882	0.352946715	7.947720048	1.90E-15	5.82E-14	1.277
PGAM1	2943.425825	0.412261679	7.938119967	2.05E-15	6.28E-14	1.331
LTBP1	8959.267079	0.401231212	7.935163513	2.10E-15	6.42E-14	1.321
ATOH8	1414.502018	-0.531663443	-7.928649927	2.22E-15	6.75E-14	-1.446
APLP1	934.4009179	0.637972178	7.911696932	2.54E-15	7.73E-14	1.556
MPRIP	9471.95007	0.331182583	7.896880531	2.86E-15	8.67E-14	1.258
NXPH4	289.6547471	0.887517112	7.895341997	2.90E-15	8.76E-14	1.850

GNA14	105.275856	1.456488364	7.89468674	2.91E-15	8.79E-14	2.744
PFKL	5418.183125	0.366036002	7.893688738	2.93E-15	8.85E-14	1.289
SMIM3	2800.470926	0.436955322	7.881094911	3.25E-15	9.77E-14	1.354
RP11-887P2.3	1235.347031	0.571120769	7.878651773	3.31E-15	9.95E-14	1.486
DDIT3	1169.389393	-0.552225509	-7.873971553	3.44E-15	1.03E-13	-1.466
MT-ND4L	12189.03165	-0.427313133	-7.853840576	4.03E-15	1.21E-13	-1.345
CALU	51289.69956	0.326483654	7.852247085	4.09E-15	1.22E-13	1.254
CDKN2A	990.3569934	-0.5347968	-7.84589004	4.30E-15	1.28E-13	-1.449
ASPH	14510.56646	0.347129795	7.834178635	4.72E-15	1.40E-13	1.272
CHSY1	2067.522004	0.424826656	7.802038686	6.09E-15	1.81E-13	1.342
FZD8	1222.179845	-0.557501622	-7.79403745	6.49E-15	1.92E-13	-1.472
PDE11A	123.3393279	1.239888525	7.790743509	6.66E-15	1.97E-13	2.362
MOSPD1	753.1675085	-0.638330193	-7.785030713	6.97E-15	2.06E-13	-1.557
DIAPH3	597.1648106	0.651662708	7.771056534	7.78E-15	2.30E-13	1.571
GALNT5	11793.08229	0.419880085	7.768764997	7.93E-15	2.33E-13	1.338
F2RL2	463.7363719	-0.730451217	-7.768524688	7.94E-15	2.33E-13	-1.659
LACTB	1569.534738	-0.467597176	-7.76692427	8.04E-15	2.36E-13	-1.383
GRAMD1B	898.7098195	0.549988434	7.738613317	1.01E-14	2.94E-13	1.464
ANXA4	1976.15942	-0.462450199	-7.737832146	1.01E-14	2.96E-13	-1.378
PRPS1	3081.94549	0.435585528	7.736344311	1.02E-14	2.99E-13	1.352
WDR19	608.7446994	-0.649394737	-7.734803828	1.04E-14	3.02E-13	-1.569
FN1	2297567.69	0.478145419	7.732743038	1.05E-14	3.06E-13	1.393
HTRA1	15783.90978	-0.335865327	-7.72609596	1.11E-14	3.22E-13	-1.262
PLK2	2146.789042	0.477159418	7.716562097	1.20E-14	3.47E-13	1.392
IER5	716.9587792	0.591770439	7.710237697	1.26E-14	3.64E-13	1.507
CSMD2	247.8207848	0.903319165	7.702523104	1.33E-14	3.86E-13	1.870
ZFP36L1	2593.822973	-0.396022821	-7.69565565	1.41E-14	4.06E-13	-1.316
MYO1D	1672.995197	-0.444143088	-7.695155149	1.41E-14	4.07E-13	-1.361
EBP	544.5508418	-0.736323494	-7.691012137	1.46E-14	4.20E-13	-1.666
IMPAD1	6365.787309	0.364149378	7.684367082	1.54E-14	4.41E-13	1.287
ZNF281	1912.654038	0.445608496	7.683178549	1.55E-14	4.44E-13	1.362
MT-RNR1	12404.31275	-0.411933984	-7.683144733	1.55E-14	4.44E-13	-1.330
MAP3K7CL	808.8169185	0.641312479	7.67648989	1.64E-14	4.67E-13	1.560
JUNB	1151.458042	0.526363044	7.664148178	1.80E-14	5.13E-13	1.440
THY1	25851.10435	0.404626252	7.655385289	1.93E-14	5.49E-13	1.324
FGF2	6199.507725	0.4091069	7.647359138	2.05E-14	5.83E-13	1.328
MID1IP1	1018.82474	-0.517287273	-7.635152814	2.26E-14	6.40E-13	-1.431
SUN2	4177.292456	0.377973471	7.629859695	2.35E-14	6.66E-13	1.300
RASEF	51.04638107	-1.645497377	-7.622609168	2.49E-14	7.03E-13	-3.129
AHNAK2	1308.076811	-0.619710037	-7.596931404	3.03E-14	8.56E-13	-1.537
DUSP8	188.2235564	0.990108865	7.590078152	3.20E-14	9.01E-13	1.986

ULBP2	530.2847513	0.677747452	7.574967583	3.59E-14	1.01E-12	1.600
TOM1L2	2845.893395	0.3973503	7.573852813	3.62E-14	1.02E-12	1.317
RP5-857K21.10	7456.834028	-0.527071336	-7.573611105	3.63E-14	1.02E-12	-1.441
TAF9B	202.8902866	-1.022172639	-7.570723529	3.71E-14	1.04E-12	-2.031
LDHB	5552.01692	-0.351598965	-7.567602274	3.80E-14	1.06E-12	-1.276
CYB561	1197.078382	0.484862135	7.566556358	3.83E-14	1.07E-12	1.399
KRTAP1-1	277.6685632	0.852547579	7.561926932	3.97E-14	1.11E-12	1.806
APOE	63.41544344	-1.625082753	-7.561362836	3.99E-14	1.11E-12	-3.085
TMCO3	5076.624678	0.344861571	7.555553475	4.17E-14	1.16E-12	1.270
KIAA1715	2277.897731	0.397064062	7.543752334	4.57E-14	1.27E-12	1.317
TMEM97	242.1549121	-0.90083158	-7.538421538	4.76E-14	1.32E-12	-1.867
FBLN1	3354.294074	-0.38591146	-7.535690287	4.86E-14	1.34E-12	-1.307
HAPLN3	1053.419242	-0.556051488	-7.5331777	4.95E-14	1.37E-12	-1.470
HLA-B	8502.584507	0.39088494	7.532349673	4.98E-14	1.37E-12	1.311
IGSF1	187.5201036	0.989601239	7.521824936	5.40E-14	1.48E-12	1.986
CASP4	816.2773037	-0.575164325	-7.521993826	5.39E-14	1.48E-12	-1.490
C19orf10	6697.18726	0.351238769	7.51910824	5.52E-14	1.51E-12	1.276
SFRP2	184.8132513	-1.074329637	-7.514871743	5.70E-14	1.56E-12	-2.106
TES	4045.375191	0.367453775	7.506407439	6.08E-14	1.66E-12	1.290
ATP8B1	4016.199677	0.415375058	7.506026805	6.09E-14	1.66E-12	1.334
HHIP	282.9254434	0.941378574	7.502756224	6.25E-14	1.70E-12	1.920
CNTNAP3B	830.2055535	0.634094956	7.500252884	6.37E-14	1.73E-12	1.552
NGF	479.0222968	0.724686105	7.499167081	6.42E-14	1.75E-12	1.653
PFKM	1669.260148	-0.461428752	-7.495015493	6.63E-14	1.80E-12	-1.377
TMEM87B	2328.471796	0.410533017	7.492691497	6.75E-14	1.83E-12	1.329
RNASEK	2956.290414	-0.370944263	-7.475410152	7.70E-14	2.08E-12	-1.293
ADGRL1	298.2051225	0.809040767	7.474035754	7.78E-14	2.10E-12	1.752
NCR3LG1	250.1518438	-0.944990683	-7.472709476	7.86E-14	2.12E-12	-1.925
TP53INP1	1045.507678	-0.516335365	-7.471234013	7.94E-14	2.14E-12	-1.430
ENO1	32942.34881	0.411943503	7.46845705	8.11E-14	2.18E-12	1.330
MSC	346.4896436	-0.777132492	-7.462126074	8.51E-14	2.29E-12	-1.714
G6PD	2854.262992	-0.429602564	-7.451347955	9.24E-14	2.48E-12	-1.347
SERPINB7	203.6569709	0.956697425	7.445031877	9.69E-14	2.59E-12	1.941
CREB3L2	3241.860445	0.405906161	7.437404754	1.03E-13	2.74E-12	1.325
SERTAD4	364.6312152	-0.76075005	-7.432173917	1.07E-13	2.85E-12	-1.694
B4GALT4	1699.648497	0.426320052	7.427148112	1.11E-13	2.96E-12	1.344
PLEKHA4	750.8025377	-0.570478484	-7.426312558	1.12E-13	2.97E-12	-1.485
TSPAN11	206.8899578	-0.969943109	-7.41555972	1.21E-13	3.22E-12	-1.959
DPP7	2367.824611	-0.399066713	-7.41435519	1.22E-13	3.24E-12	-1.319
TMEM38B	348.4220376	-0.766614244	-7.409029733	1.27E-13	3.37E-12	-1.701
MAP1A	15468.96992	0.344489894	7.407449859	1.29E-13	3.40E-12	1.270

PCK2	1605.443048	0.444392214	7.401951408	1.34E-13	3.54E-12	1.361
SLC17A9	605.8067934	0.694789097	7.397991594	1.38E-13	3.64E-12	1.619
AK4P1	418.3128393	0.727894881	7.39537463	1.41E-13	3.71E-12	1.656
C11orf87	695.9689442	0.644031384	7.392891571	1.44E-13	3.77E-12	1.563
LUM	14959.24776	-0.397747002	-7.390491322	1.46E-13	3.84E-12	-1.317
LTBP3	8992.83173	0.374001782	7.388309296	1.49E-13	3.89E-12	1.296
ID3	4669.113895	-0.43474331	-7.379370493	1.59E-13	4.16E-12	-1.352
HTR2B	30.88032605	-1.862210325	-7.378885808	1.60E-13	4.17E-12	-3.636
ZFP36L2	2158.287807	-0.454785547	-7.37182153	1.68E-13	4.38E-12	-1.371
KLHL24	885.7003348	-0.566673506	-7.371742928	1.68E-13	4.38E-12	-1.481
IL21R	333.8738815	-0.766366773	-7.365989978	1.76E-13	4.57E-12	-1.701
SLC35E1	3298.570804	0.387737071	7.353949677	1.92E-13	4.99E-12	1.308
DNASE2	2057.071956	-0.429742624	-7.351946872	1.95E-13	5.06E-12	-1.347
PER1	242.9935798	0.887896	7.346627346	2.03E-13	5.25E-12	1.850
CENPF	470.9089268	-0.748932409	-7.346640475	2.03E-13	5.25E-12	-1.681
NFIC	4091.418183	0.348711958	7.342299439	2.10E-13	5.42E-12	1.273
HEXA	3493.716048	-0.373224456	-7.330905073	2.29E-13	5.89E-12	-1.295
RP13-138P15.3	61.61055749	1.461892066	7.32748682	2.35E-13	6.03E-12	2.755
MAF	150.2188353	-1.074824167	-7.325159933	2.39E-13	6.13E-12	-2.106
TMEM126B	662.1639973	-0.63279934	-7.321253012	2.46E-13	6.30E-12	-1.551
TAGLN2	8739.334017	0.390037732	7.318078056	2.52E-13	6.44E-12	1.310
OSR1	274.2547034	0.833779418	7.314600956	2.58E-13	6.59E-12	1.782
SLC6A9	778.1042725	0.539651853	7.309411698	2.68E-13	6.84E-12	1.454
GALNT10	12106.31325	0.329966441	7.309051491	2.69E-13	6.85E-12	1.257
MPP2	116.6760079	1.175007822	7.308011755	2.71E-13	6.89E-12	2.258
DUSP10	438.7020725	-0.692205713	-7.306861251	2.73E-13	6.94E-12	-1.616
ABCA2	3146.419332	0.367110287	7.303684377	2.80E-13	7.10E-12	1.290
CCNA2	251.1961016	-0.920085577	-7.303295567	2.81E-13	7.11E-12	-1.892
PYCR1	4003.73521	0.390592317	7.285634334	3.20E-13	8.09E-12	1.311
ROS1	125.5725885	1.158357662	7.282733943	3.27E-13	8.26E-12	2.232
KCNH1	318.2506379	-0.813853013	-7.265493484	3.72E-13	9.37E-12	-1.758
NCKAP5	367.9527648	0.802623328	7.259982671	3.87E-13	9.74E-12	1.744
GYPC	647.3645035	-0.625852243	-7.253304304	4.07E-13	1.02E-11	-1.543
ETV1	237.0517764	-0.890966934	-7.241648694	4.43E-13	1.11E-11	-1.854
MT-CO1	142086.8539	-0.473979109	-7.240259946	4.48E-13	1.12E-11	-1.389
RP3-523C21.1	153.7600199	1.037313838	7.238280859	4.54E-13	1.14E-11	2.052
AC002480.3	193.8959	0.934120376	7.237892369	4.56E-13	1.14E-11	1.911
TSPAN13	481.9027256	0.679798222	7.225032627	5.01E-13	1.25E-11	1.602
SACS	1499.681122	0.580353966	7.220387258	5.18E-13	1.29E-11	1.495
NMNAT2	519.278917	0.625455526	7.207441303	5.70E-13	1.42E-11	1.543
ITGA8	1187.250277	0.549914129	7.201276346	5.97E-13	1.48E-11	1.464

KRT14	91.75177878	1.246137979	7.19341369	6.32E-13	1.56E-11	2.372
NPAS1	800.086052	0.551130382	7.187141654	6.62E-13	1.63E-11	1.465
EHBP1L1	3946.324984	0.350478835	7.183599441	6.79E-13	1.67E-11	1.275
SOX4	1530.846811	-0.55070501	-7.176820815	7.14E-13	1.75E-11	-1.465
RDH5	183.3186106	0.94589912	7.169291947	7.54E-13	1.85E-11	1.926
KANK2	4474.832461	-0.34939665	-7.167926715	7.61E-13	1.86E-11	-1.274
CPEB1	90.40178976	-1.26277239	-7.164983653	7.78E-13	1.90E-11	-2.400
ELL2	3331.164526	0.345242551	7.146479531	8.90E-13	2.17E-11	1.270
RHOC	8031.686529	0.392108013	7.14618364	8.92E-13	2.18E-11	1.312
OAS3	175.8514726	-1.023753482	-7.145364602	8.98E-13	2.19E-11	-2.033
LEP	102.5776812	1.197058	7.139475666	9.37E-13	2.28E-11	2.293
PLA2G4C	554.4657441	-0.631536495	-7.136143806	9.60E-13	2.33E-11	-1.549
ARMCX1	1066.969447	-0.490486614	-7.128370557	1.02E-12	2.46E-11	-1.405
SEC23A	8355.247083	0.367452985	7.112271254	1.14E-12	2.76E-11	1.290
SOAT1	1500.153343	-0.426601974	-7.107223248	1.18E-12	2.86E-11	-1.344
TGFBR1	5037.112407	0.4005178	7.095406801	1.29E-12	3.11E-11	1.320
TOP2A	836.8758841	-0.551570288	-7.081802921	1.42E-12	3.43E-11	-1.466
COL13A1	408.293819	0.679720018	7.078108583	1.46E-12	3.51E-11	1.602
RP11-490H24.5	852.7877483	0.531660909	7.076011451	1.48E-12	3.56E-11	1.446
ACP5	36.70885519	-1.645506228	-7.061306036	1.65E-12	3.95E-11	-3.129
IER5L	976.0192601	-0.529152027	-7.060113414	1.66E-12	3.98E-11	-1.443
MAP2K1	1755.216831	0.471052193	7.058035163	1.69E-12	4.03E-11	1.386
LBP	39.83511164	-1.646710516	-7.058044044	1.69E-12	4.03E-11	-3.131
MGST3	2453.960718	-0.399952192	-7.048980745	1.80E-12	4.29E-11	-1.319
CHST3	3622.444438	0.350232276	7.038452557	1.94E-12	4.62E-11	1.275
TGFA	105.3759245	1.156621337	7.036337793	1.97E-12	4.68E-11	2.229
MAP1B	15990.00521	0.409223484	7.030818716	2.05E-12	4.86E-11	1.328
CTHRC1	3683.389277	-0.37169308	-7.028636299	2.09E-12	4.93E-11	-1.294
AGRN	2943.212618	0.393172918	7.018812777	2.24E-12	5.28E-11	1.313
PTGIS	1817.240621	0.488569163	7.015063092	2.30E-12	5.42E-11	1.403
SUN1	2682.237725	0.377111	7.012369253	2.34E-12	5.52E-11	1.299
TMEM245	2838.224767	0.420985391	7.010657441	2.37E-12	5.57E-11	1.339
SLC4A4	1354.10109	0.527135896	7.009958163	2.38E-12	5.59E-11	1.441
TMEM192	782.3012256	-0.550352361	-7.005927495	2.45E-12	5.75E-11	-1.464
GPI	7352.79639	0.352821191	7.003340061	2.50E-12	5.85E-11	1.277
TRIM16L	379.3643657	-0.753575806	-7.002427121	2.52E-12	5.88E-11	-1.686
GOLGA3	3525.703202	0.355799836	6.99141866	2.72E-12	6.35E-11	1.280
OXSR1	2167.271374	0.381020597	6.982313916	2.90E-12	6.77E-11	1.302
PFKP	6966.5819	0.330511002	6.981975943	2.91E-12	6.77E-11	1.257
CTPS1	1991.763023	0.419678606	6.981306701	2.92E-12	6.80E-11	1.338
INA	141.6756107	1.066699963	6.979299223	2.97E-12	6.88E-11	2.095

ECM1	1416.766655	-0.486852851	-6.974028874	3.08E-12	7.14E-11	-1.401
MAPKAPK3	1145.017125	-0.524796679	-6.96425733	3.30E-12	7.63E-11	-1.439
WBP2	2026.733226	-0.42548817	-6.9618088	3.36E-12	7.75E-11	-1.343
TANC2	1316.657923	0.587265361	6.957003444	3.48E-12	8.01E-11	1.502
FAM43A	1201.081698	-0.445810578	-6.948661497	3.69E-12	8.49E-11	-1.362
RPS27L	3745.736047	-0.365267076	-6.947016963	3.73E-12	8.58E-11	-1.288
MLPH	728.2958991	-0.644216014	-6.942194277	3.86E-12	8.86E-11	-1.563
STX12	2559.052882	0.358284318	6.936510085	4.02E-12	9.22E-11	1.282
MAGED1	15178.93609	0.324205122	6.933335842	4.11E-12	9.41E-11	1.252
PLEKHM2	2705.222497	-0.369718636	-6.920124191	4.51E-12	1.03E-10	-1.292
ECHDC1	1221.607578	-0.444004198	-6.914569578	4.69E-12	1.07E-10	-1.360
IFFO2	938.8886369	0.499408465	6.91105672	4.81E-12	1.10E-10	1.414
SYNPO2	8169.309069	0.425088522	6.907626977	4.93E-12	1.12E-10	1.343
CADPS2	116.0417627	-1.109510329	-6.902037355	5.13E-12	1.16E-10	-2.158
CD274	107.2518616	1.168954788	6.896252895	5.34E-12	1.21E-10	2.248
GLB1	4161.065977	-0.376094953	-6.89462873	5.40E-12	1.22E-10	-1.298
CLU	1032.773223	0.521466836	6.893238094	5.45E-12	1.23E-10	1.435
PGM3	3666.073344	0.340421963	6.892867308	5.47E-12	1.24E-10	1.266
LMF2	2976.529992	0.3861981	6.8912444	5.53E-12	1.25E-10	1.307
RCBTB1	945.5671793	-0.535731088	-6.890714465	5.55E-12	1.25E-10	-1.450
PLAUR	2170.104374	0.444660764	6.874722837	6.21E-12	1.40E-10	1.361
P3H1	7460.094821	0.330130387	6.865944804	6.61E-12	1.48E-10	1.257
QSOX2	1313.611757	0.431611473	6.861127671	6.83E-12	1.53E-10	1.349
ZYX	10410.97952	0.338911121	6.858232608	6.97E-12	1.56E-10	1.265
NDEL1	2268.187248	0.361666881	6.857975289	6.98E-12	1.56E-10	1.285
KIAA1191	3411.91434	0.341958379	6.848723169	7.45E-12	1.66E-10	1.267
GEM	354.031174	-0.714673999	-6.848209096	7.48E-12	1.67E-10	-1.641
TOR1B	2727.316774	0.350615039	6.84652461	7.57E-12	1.68E-10	1.275
KRTAP1-5	1651.646592	0.432513287	6.841019892	7.86E-12	1.75E-10	1.350
NLRP10	139.1445612	1.090939127	6.829099923	8.54E-12	1.90E-10	2.130
CYTH3	1987.426544	-0.398464611	-6.822987897	8.92E-12	1.98E-10	-1.318
LPAR6	160.4235664	-0.977120239	-6.817140232	9.29E-12	2.06E-10	-1.969
SNAI2	1688.426234	-0.386922572	-6.811576876	9.65E-12	2.13E-10	-1.308
SIDT2	3114.526622	0.348430212	6.805566023	1.01E-11	2.22E-10	1.273
HSPH1	3067.875525	-0.367667713	-6.797743419	1.06E-11	2.34E-10	-1.290
RP11-81A1.3	125.450533	-1.038086167	-6.796918203	1.07E-11	2.35E-10	-2.054
CPNE8	277.4155834	-0.818670557	-6.795146636	1.08E-11	2.38E-10	-1.764
ERCC6	3319.640163	0.422976596	6.790611084	1.12E-11	2.45E-10	1.341
APOL1	1178.304944	-0.456814827	-6.79045561	1.12E-11	2.45E-10	-1.373
MROH1	886.7280705	-0.49523392	-6.78763516	1.14E-11	2.49E-10	-1.410
PKD1	6501.514656	0.407046833	6.778504377	1.21E-11	2.65E-10	1.326

RNF41	2601.793337	0.363735041	6.773244793	1.26E-11	2.75E-10	1.287
DEPTOR	1350.456143	-0.442863976	-6.761020391	1.37E-11	2.99E-10	-1.359
GGCX	2223.774261	0.366761823	6.75836028	1.40E-11	3.04E-10	1.289
SEC14L2	606.6533435	0.618290573	6.751932114	1.46E-11	3.17E-10	1.535
VASP	3185.833781	0.36279394	6.748644179	1.49E-11	3.23E-10	1.286
SPP1	80.51220361	-1.270610811	-6.748818157	1.49E-11	3.23E-10	-2.413
SPHK1	1521.343902	-0.446376052	-6.744705135	1.53E-11	3.32E-10	-1.363
PPP1R15B	1248.333602	0.447759148	6.735365642	1.64E-11	3.53E-10	1.364
IRF2BP2	3186.283601	-0.379957195	-6.728942796	1.71E-11	3.68E-10	-1.301
DGKI	1460.79129	0.48897484	6.723437204	1.77E-11	3.82E-10	1.403
ANKRD10	425.749099	-0.666765021	-6.720876235	1.81E-11	3.88E-10	-1.588
GFPT1	5997.004923	0.331197336	6.71023805	1.94E-11	4.17E-10	1.258
RP11-383H13.1	663.2548741	-0.530458081	-6.707154299	1.98E-11	4.25E-10	-1.444
SH3RF2	223.4653277	0.811711321	6.696728442	2.13E-11	4.55E-10	1.755
DTX3L	409.3914085	-0.663037225	-6.695058716	2.16E-11	4.60E-10	-1.583
SHANK1	88.08762873	1.188190191	6.687674969	2.27E-11	4.83E-10	2.279
IFI35	141.1203917	-1.019713577	-6.675457559	2.46E-11	5.24E-10	-2.028
ABCA3	301.0119306	0.713896995	6.650983587	2.91E-11	6.18E-10	1.640
SLC7A1	4368.220274	0.329386917	6.648298972	2.96E-11	6.29E-10	1.256
CYB5RL	189.7795615	-0.872985836	-6.647330639	2.98E-11	6.32E-10	-1.831
RASSF7	179.5008558	0.881379059	6.640964505	3.12E-11	6.60E-10	1.842
APOLD1	244.7420659	0.80822047	6.636427867	3.21E-11	6.79E-10	1.751
KIF20A	224.2460085	-0.843229635	-6.626161452	3.45E-11	7.27E-10	-1.794
FAM107B	1369.009888	0.423526535	6.624177032	3.49E-11	7.36E-10	1.341
CKS2	270.9882873	-0.769347429	-6.620418139	3.58E-11	7.54E-10	-1.704
SPAG4	117.8987217	1.05922707	6.604475679	3.99E-11	8.39E-10	2.084
KATNAL1	1147.498721	0.500886337	6.602641661	4.04E-11	8.49E-10	1.415
ASAP3	1042.306103	-0.454110113	-6.599781452	4.12E-11	8.64E-10	-1.370
MANBA	1701.067441	-0.420847858	-6.598817029	4.14E-11	8.69E-10	-1.339
EXTL1	829.8825806	0.496319394	6.592748501	4.32E-11	9.02E-10	1.411
ENPP2	1925.45775	-0.401777642	-6.592857806	4.31E-11	9.02E-10	-1.321
ACSS2	811.9531365	-0.569625098	-6.583901084	4.58E-11	9.56E-10	-1.484
KDM4B	1613.693428	0.436388599	6.576847868	4.81E-11	9.99E-10	1.353
CD82	413.3983047	-0.636782595	-6.574414249	4.88E-11	1.01E-09	-1.555
CMAHP	303.7914408	0.705142914	6.557284487	5.48E-11	1.13E-09	1.630
PIK3IP1	633.9110774	-0.546311142	-6.54705634	5.87E-11	1.21E-09	-1.460
PPP1R12A	3737.752594	0.376483217	6.539130086	6.19E-11	1.28E-09	1.298
CCNB1	487.9578141	-0.605058926	-6.535205947	6.35E-11	1.31E-09	-1.521
CCND3	939.1608031	0.47040855	6.532189311	6.48E-11	1.33E-09	1.386
PODN	1022.389807	-0.4756295	-6.531560428	6.51E-11	1.34E-09	-1.391
GTF2H1	1545.830404	-0.396994774	-6.528084251	6.66E-11	1.37E-09	-1.317

FZD4	757.2712962	-0.518481545	-6.527896641	6.67E-11	1.37E-09	-1.432
AC092066.1	354.7555046	0.716942331	6.524936431	6.80E-11	1.39E-09	1.644
VSIR	2028.935779	0.389055644	6.524667845	6.82E-11	1.39E-09	1.310
NUDT14	151.9653702	-0.961400291	-6.524557234	6.82E-11	1.39E-09	-1.947
SLC38A7	1471.921076	-0.413509829	-6.52291534	6.90E-11	1.41E-09	-1.332
AMPD2	2258.089023	0.363262752	6.50960073	7.54E-11	1.54E-09	1.286
FOXM1	359.670302	-0.689208573	-6.50893536	7.57E-11	1.54E-09	-1.612
ADCY6	2561.336341	0.341149507	6.508727138	7.58E-11	1.54E-09	1.267
WLS	1553.957927	-0.386190431	-6.490572559	8.55E-11	1.74E-09	-1.307
SPRED3	396.9658748	0.622401939	6.481903397	9.06E-11	1.84E-09	1.539
TNFRSF14	262.3930844	-0.771808754	-6.47119436	9.72E-11	1.96E-09	-1.707
TEK	233.8078395	0.787613439	6.450608539	1.11E-10	2.25E-09	1.726
FOS	426.1819917	1.297225012	6.443804909	1.17E-10	2.34E-09	2.458
FLRT2	985.1811274	-0.450525557	-6.443640133	1.17E-10	2.34E-09	-1.367
KLHL21	3214.327254	-0.322184928	-6.438460879	1.21E-10	2.42E-09	-1.250
TCEAL9	3079.911277	-0.329747034	-6.434266605	1.24E-10	2.48E-09	-1.257
LRP3	1757.01535	-0.364977924	-6.429821002	1.28E-10	2.55E-09	-1.288
DYNC2H1	762.0413752	-0.64511479	-6.426160194	1.31E-10	2.61E-09	-1.564
PIR	302.1018791	-0.689959015	-6.424221971	1.33E-10	2.64E-09	-1.613
VPS18	1274.342968	-0.431701605	-6.418878179	1.37E-10	2.73E-09	-1.349
SPEG	1840.82312	0.37574304	6.414355115	1.41E-10	2.81E-09	1.298
SCN9A	1425.216991	0.526428988	6.407154989	1.48E-10	2.94E-09	1.440
SLC43A3	312.6078992	-0.709396071	-6.404011839	1.51E-10	3.00E-09	-1.635
GNPTG	1632.867337	-0.401906178	-6.400601804	1.55E-10	3.06E-09	-1.321
SCRG1	686.456735	0.51918281	6.398861662	1.57E-10	3.09E-09	1.433
ANKRD28	1205.214683	-0.423848666	-6.39884794	1.57E-10	3.09E-09	-1.342
MLXIP	1608.667509	0.382551113	6.397530617	1.58E-10	3.11E-09	1.304
TMSL6	1347.00198	-0.490131543	-6.394690331	1.61E-10	3.16E-09	-1.405
SERPINF1	1088.502741	-0.425834116	-6.383589449	1.73E-10	3.40E-09	-1.343
RARG	1656.790337	-0.392300851	-6.382030773	1.75E-10	3.43E-09	-1.312
STAC	610.2898981	0.546383525	6.375510874	1.82E-10	3.57E-09	1.460
KIAA2013	1822.588192	0.374311979	6.37436439	1.84E-10	3.59E-09	1.296
PLAU	3713.166218	0.34030946	6.37299208	1.85E-10	3.62E-09	1.266
RAC2	858.2129205	0.461887545	6.362932385	1.98E-10	3.86E-09	1.377
ITGA2	1485.312448	0.48315131	6.360830904	2.01E-10	3.91E-09	1.398
RP11-123J14.2	1221.123831	-0.44027633	-6.357716865	2.05E-10	3.98E-09	-1.357
RCOR1	1135.988575	0.42578947	6.354620975	2.09E-10	4.06E-09	1.343
ANKLE2	2345.279316	0.329410333	6.344750068	2.23E-10	4.32E-09	1.256
UPP1	173.9438428	-0.85450951	-6.342675451	2.26E-10	4.38E-09	-1.808
ARL10	839.4937165	0.523361688	6.33951049	2.30E-10	4.46E-09	1.437
PDPK1	1531.421297	0.401149038	6.329206	2.46E-10	4.76E-09	1.321

LAMTOR1	2224.472947	-0.418400704	-6.327346912	2.49E-10	4.81E-09	-1.336
SEC24A	2518.147449	0.387658877	6.325222569	2.53E-10	4.87E-09	1.308
TLE3	1171.491642	0.452046699	6.3101341	2.79E-10	5.34E-09	1.368
GNPNAT1	2132.999436	0.35174491	6.306354562	2.86E-10	5.46E-09	1.276
GDF6	875.9644284	0.51926091	6.303758535	2.91E-10	5.55E-09	1.433
YEATS2	1971.454579	0.372731307	6.303348926	2.91E-10	5.55E-09	1.295
MXI1	1079.902617	0.433368049	6.297256407	3.03E-10	5.76E-09	1.350
PTN	312.8125114	-0.686059251	-6.295857357	3.06E-10	5.80E-09	-1.609
CC2D2A	454.0478827	-0.57607476	-6.291271263	3.15E-10	5.97E-09	-1.491
DDR1	1873.485502	0.354140197	6.276969579	3.45E-10	6.52E-09	1.278
TAF9	1289.365418	-0.413455166	-6.27642628	3.46E-10	6.54E-09	-1.332
TMEM2	2473.639206	0.380918761	6.275715556	3.48E-10	6.56E-09	1.302
SPECC1	1078.265228	-0.423303681	-6.274518846	3.51E-10	6.61E-09	-1.341
HES1	134.7413141	-0.984714573	-6.273726561	3.53E-10	6.63E-09	-1.979
KIAA1755	203.2803442	0.803385005	6.271565744	3.57E-10	6.72E-09	1.745
SH2D5	121.272105	-1.031679572	-6.261179721	3.82E-10	7.17E-09	-2.044
NECTIN2	2393.969273	0.333603499	6.245138136	4.23E-10	7.92E-09	1.260
CORO2B	815.9508567	0.470694463	6.24492927	4.24E-10	7.93E-09	1.386
ST6GAL1	162.5631793	-0.861397072	-6.239430548	4.39E-10	8.19E-09	-1.817
ID1	1155.378525	-0.414335614	-6.238903709	4.41E-10	8.21E-09	-1.333
LDHBP	795.7271437	-0.486527107	-6.238564931	4.42E-10	8.22E-09	-1.401
CHAC1	1303.08845	0.423103091	6.237227414	4.45E-10	8.28E-09	1.341
CHST15	912.7398693	0.456138268	6.231536526	4.62E-10	8.58E-09	1.372
SYNPO	2034.493487	0.344480453	6.227180527	4.75E-10	8.81E-09	1.270
IL1R1	2409.407277	-0.389761096	-6.214297418	5.16E-10	9.55E-09	-1.310
BCL10	687.726928	0.490251434	6.212386358	5.22E-10	9.65E-09	1.405
GLTSCR2	1280.543846	-0.406647017	-6.202936789	5.54E-10	1.02E-08	-1.326
MCOLN1	794.6581064	-0.493882906	-6.199646276	5.66E-10	1.04E-08	-1.408
AC073115.6	76.26050746	1.134466898	6.198457717	5.70E-10	1.05E-08	2.195
RP1-239B22.5	67.48148185	-1.19334964	-6.195781811	5.80E-10	1.07E-08	-2.287
HIP1	1828.333405	-0.365992493	-6.195511354	5.81E-10	1.07E-08	-1.289
PJA2	5067.484065	0.371239979	6.194922746	5.83E-10	1.07E-08	1.293
SLC39A10	1279.701808	0.428667526	6.194741656	5.84E-10	1.07E-08	1.346
FGF7	5244.890419	-0.342771848	-6.190587944	5.99E-10	1.10E-08	-1.268
SPTLC3	214.4160981	-0.7640348	-6.189202947	6.05E-10	1.11E-08	-1.698
CRELD1	1040.692938	0.481932328	6.187046598	6.13E-10	1.12E-08	1.397
TOR4A	222.0213148	0.752185348	6.183392286	6.27E-10	1.15E-08	1.684
COBLL1	549.5567234	0.543425002	6.168984177	6.87E-10	1.25E-08	1.457
DDX39A	484.5455675	-0.601087521	-6.158930606	7.32E-10	1.33E-08	-1.517
ISCU	1461.475777	-0.391741407	-6.151990033	7.65E-10	1.39E-08	-1.312
GDNF	598.436417	0.527204487	6.146081593	7.94E-10	1.44E-08	1.441

IMPDH1	1742.652435	0.373520803	6.137055392	8.41E-10	1.52E-08	1.296
PXDC1	4358.392839	0.322198265	6.13584445	8.47E-10	1.53E-08	1.250
SPSB2	167.8585162	-0.889956539	-6.13360678	8.59E-10	1.55E-08	-1.853
GRAMD3	2369.696986	0.337363881	6.125094552	9.06E-10	1.63E-08	1.263
PGAM4	260.624237	0.720672506	6.119092444	9.41E-10	1.70E-08	1.648
STOM	1909.149779	-0.350024972	-6.112915365	9.78E-10	1.76E-08	-1.275
AVPI1	754.2893837	-0.503680039	-6.110030072	9.96E-10	1.79E-08	-1.418
ALKBH5	1995.532046	0.365809086	6.108463586	1.01E-09	1.80E-08	1.289
TRIM47	164.2652574	-0.863510604	-6.104612352	1.03E-09	1.85E-08	-1.819
ETNK1	596.5010811	-0.510154415	-6.10402306	1.03E-09	1.85E-08	-1.424
GPR155	415.5843577	0.589581975	6.096449781	1.08E-09	1.94E-08	1.505
PACSIN3	504.1203849	0.54917288	6.093363623	1.11E-09	1.98E-08	1.463
BCYRN1	263.6064018	-0.78700513	-6.088025366	1.14E-09	2.04E-08	-1.725
SAT2	638.7105989	-0.522859064	-6.081610592	1.19E-09	2.12E-08	-1.437
RP11-644F5.10	167.9428221	0.834104425	6.080711326	1.20E-09	2.13E-08	1.783
ALDH1B1	2440.67597	0.357558811	6.079949207	1.20E-09	2.13E-08	1.281
MAT2A	4464.43873	-0.33584899	-6.060336251	1.36E-09	2.41E-08	-1.262
TTC7A	964.1408536	0.429731215	6.05987035	1.36E-09	2.41E-08	1.347
CORO1B	2052.060621	-0.373202876	-6.056008037	1.40E-09	2.47E-08	-1.295
ADH5	3431.752866	-0.339039548	-6.054634468	1.41E-09	2.48E-08	-1.265
FAM212B	130.0269517	0.943261406	6.048555111	1.46E-09	2.58E-08	1.923
SNTB1	1354.874325	-0.402860581	-6.047540579	1.47E-09	2.59E-08	-1.322
ACVR2A	728.2968476	0.485334604	6.035283283	1.59E-09	2.79E-08	1.400
MGAM	176.2734059	0.828272782	6.018732168	1.76E-09	3.08E-08	1.776
MAFB	113.760925	-1.003489075	-6.017136574	1.78E-09	3.11E-08	-2.005
NOV	112.8391724	-0.951369096	-6.011904902	1.83E-09	3.21E-08	-1.934
RP11-75A9.2	2221.727923	0.364619443	6.009137086	1.87E-09	3.26E-08	1.288
CELSR2	391.9689218	0.594521993	6.007965129	1.88E-09	3.28E-08	1.510
TMTC1	1060.051995	-0.436568664	-6.005177665	1.91E-09	3.33E-08	-1.353
BRIX1	571.5556619	-0.507635519	-6.005263595	1.91E-09	3.33E-08	-1.422
EEF1A1	30880.86868	-0.333533835	-5.996624772	2.01E-09	3.50E-08	-1.260
TMEM261	534.901571	-0.55878408	-5.995527543	2.03E-09	3.52E-08	-1.473
NT5DC2	5575.544026	0.323497835	5.994564079	2.04E-09	3.54E-08	1.251
LCNL1	31.29805869	-1.392278653	-5.992564425	2.07E-09	3.58E-08	-2.625
HIPK2	2061.848871	0.445328193	5.991813037	2.08E-09	3.59E-08	1.362
LRRFIP1	2937.749656	0.351720389	5.990180923	2.10E-09	3.62E-08	1.276
FAM168A	2224.994247	0.336281755	5.988566639	2.12E-09	3.65E-08	1.262
PHACTR2	1483.840465	0.425241882	5.986727751	2.14E-09	3.69E-08	1.343
CREB3	1779.044701	0.338724389	5.985301073	2.16E-09	3.72E-08	1.265
FAM171A1	1796.715537	0.345892441	5.984847017	2.17E-09	3.72E-08	1.271
KDM5B	3156.42169	0.331666791	5.984684443	2.17E-09	3.72E-08	1.258

CTD-2195M18.1	148.8557374	0.891397803	5.983108861	2.19E-09	3.76E-08	1.855
MMP17	441.7193862	-0.586383014	-5.981516334	2.21E-09	3.79E-08	-1.501
PPP2R5B	997.6514896	0.436448003	5.977729681	2.26E-09	3.87E-08	1.353
LAPTM4B	2043.524382	-0.362457394	-5.975142609	2.30E-09	3.93E-08	-1.286
ADAM15	3569.251704	0.331292956	5.96857206	2.39E-09	4.08E-08	1.258
MGP	702.9754104	-0.516189331	-5.968637893	2.39E-09	4.08E-08	-1.430
PCDH1	222.0553889	0.725701851	5.966916123	2.42E-09	4.12E-08	1.654
EPGN	317.3946197	0.644400605	5.964447438	2.45E-09	4.18E-08	1.563
CD200	71.90615678	1.105118307	5.954177846	2.61E-09	4.45E-08	2.151
MOGS	2298.448618	0.32447381	5.953324431	2.63E-09	4.46E-08	1.252
TXNDC16	231.8221176	-0.719211944	-5.951881549	2.65E-09	4.50E-08	-1.646
IFI44L	101.0841515	-1.016457743	-5.950150459	2.68E-09	4.54E-08	-2.023
B4GALT2	2956.313279	0.32651303	5.947794773	2.72E-09	4.60E-08	1.254
ZNF703	981.9750915	0.411054507	5.944653205	2.77E-09	4.69E-08	1.330
PLA1A	42.48831308	-1.283970778	-5.944535346	2.77E-09	4.69E-08	-2.435
ANTXR1	8386.874859	-0.329877879	-5.943898625	2.78E-09	4.70E-08	-1.257
AK5	591.1445695	-0.510111048	-5.943618493	2.79E-09	4.70E-08	-1.424
LIPG	283.816949	-0.704086339	-5.94284693	2.80E-09	4.72E-08	-1.629
PADI2	270.1313835	0.691027003	5.941303602	2.83E-09	4.76E-08	1.614
BAIAP2L1	698.9874861	0.456520494	5.930844641	3.01E-09	5.06E-08	1.372
BTN3A1	446.445282	-0.544557745	-5.928292276	3.06E-09	5.13E-08	-1.459
SLC9A3R2	1268.31667	0.381495243	5.926546033	3.09E-09	5.18E-08	1.303
FNDC3B	9744.784092	0.337819615	5.924391927	3.13E-09	5.24E-08	1.264
IFIT2	109.6637358	-0.990938682	-5.924377802	3.13E-09	5.24E-08	-1.987
MAFF	614.4617717	0.521825083	5.916705634	3.28E-09	5.48E-08	1.436
MRVI1	1485.138213	0.365088934	5.909616497	3.43E-09	5.71E-08	1.288
IFIT1	149.2597259	-1.00573317	-5.908928372	3.44E-09	5.73E-08	-2.008
SNN	708.3966023	-0.469611896	-5.907658361	3.47E-09	5.77E-08	-1.385
TMED9	11502.28827	0.332118962	5.902795513	3.57E-09	5.93E-08	1.259
COL11A1	1802.087664	-0.374569613	-5.898969767	3.66E-09	6.07E-08	-1.296
FAM129A	1084.257032	-0.463452964	-5.898561032	3.67E-09	6.07E-08	-1.379
CCL2	785.9717153	0.459592334	5.894106704	3.77E-09	6.23E-08	1.375
C17orf107	190.9812216	0.772827797	5.890216796	3.86E-09	6.37E-08	1.709
SORCS2	459.3166441	0.547121009	5.888781922	3.89E-09	6.42E-08	1.461
PDLIM2	2509.953068	0.340499217	5.888194633	3.90E-09	6.44E-08	1.266
GLIS3	494.2240464	0.56426872	5.884683243	3.99E-09	6.57E-08	1.479
MVK	303.7787169	-0.650765003	-5.88353441	4.02E-09	6.61E-08	-1.570
GRASP	40.22996475	-1.300165222	-5.880450302	4.09E-09	6.73E-08	-2.463
WASHC2A	1722.6999	-0.452279924	-5.877539341	4.16E-09	6.84E-08	-1.368
MAP3K12	560.13483	-0.609565099	-5.874491893	4.24E-09	6.96E-08	-1.526
RP11-761N21.2	1722.514837	-0.390323958	-5.87054572	4.34E-09	7.12E-08	-1.311

SLC24A3	468.6654511	0.546192002	5.86304016	4.54E-09	7.44E-08	1.460
LINC00595	107.216197	-0.972570105	-5.862646853	4.56E-09	7.45E-08	-1.962
UBR3	2007.105366	0.398560889	5.858235299	4.68E-09	7.65E-08	1.318
DNMBP	1525.414697	0.343076453	5.855745208	4.75E-09	7.74E-08	1.268
LAYN	1229.847311	0.373057996	5.853428437	4.82E-09	7.84E-08	1.295
MET	8287.242362	0.348299439	5.852907244	4.83E-09	7.86E-08	1.273
VPS26A	1869.673689	-0.33170129	-5.84547166	5.05E-09	8.20E-08	-1.258
ZNF330	651.2590234	-0.484655952	-5.843710633	5.11E-09	8.28E-08	-1.399
FAM149A	97.21106884	-0.982874743	-5.841880923	5.16E-09	8.35E-08	-1.976
SOGA1	2356.207744	0.395382536	5.837680538	5.29E-09	8.56E-08	1.315
C2CD2	1590.172774	-0.345930792	-5.832961259	5.45E-09	8.78E-08	-1.271
APOL6	449.0532995	-0.549234225	-5.831539313	5.49E-09	8.84E-08	-1.463
CDK6	2222.786247	0.362597507	5.825377826	5.70E-09	9.16E-08	1.286
ZNF117	437.3439316	-0.635789038	-5.821073576	5.85E-09	9.39E-08	-1.554
SPTY2D1	1587.635187	0.355635342	5.820308083	5.87E-09	9.42E-08	1.280
HSD17B11	495.9282124	-0.527295138	-5.817367554	5.98E-09	9.58E-08	-1.441
PRR16	951.4695144	0.421450272	5.816990583	5.99E-09	9.59E-08	1.339
ACAA2	1746.892251	0.380051617	5.809143202	6.28E-09	1.00E-07	1.301
TCAF1	2121.42108	0.388359812	5.799443289	6.65E-09	1.06E-07	1.309
GPC6	1335.101871	-0.408003526	-5.798151663	6.70E-09	1.07E-07	-1.327
CCDC190	66.37855478	1.107347552	5.79611923	6.79E-09	1.08E-07	2.154
IFI44	217.9346146	-0.747889846	-5.795004278	6.83E-09	1.09E-07	-1.679
MCOLN3	40.13530603	-1.275392035	-5.793214737	6.91E-09	1.10E-07	-2.421
MAFK	1099.66743	0.378190372	5.792608866	6.93E-09	1.10E-07	1.300
PFKFB2	279.8796474	-0.683376044	-5.789214578	7.07E-09	1.12E-07	-1.606
FOSB	84.86352684	1.145666695	5.776652689	7.62E-09	1.21E-07	2.212
AC012667.1	40.05536935	1.263349231	5.769636309	7.94E-09	1.26E-07	2.401
ALDOAP2	1058.364044	0.400897043	5.768590998	7.99E-09	1.26E-07	1.320
TGIF1	784.2145612	-0.445321827	-5.766012058	8.12E-09	1.28E-07	-1.362
STK39	1491.42688	0.340579506	5.753507989	8.74E-09	1.38E-07	1.266
TST	767.6871412	0.465973403	5.75308813	8.76E-09	1.38E-07	1.381
RP1-222H5.1	1036.570502	-0.400234091	-5.752909282	8.77E-09	1.38E-07	-1.320
SETD3	1457.841653	0.353288047	5.751061235	8.87E-09	1.39E-07	1.277
C20orf82	89.54743205	-0.979672657	-5.713799861	1.10E-08	1.73E-07	-1.972
RPL26P19	2024.16549	-0.34231993	-5.712458197	1.11E-08	1.74E-07	-1.268
AKIRIN1	2179.886713	0.334604537	5.711397155	1.12E-08	1.75E-07	1.261
IFI6	335.401859	-0.642515475	-5.708095	1.14E-08	1.79E-07	-1.561
PLEKHA5	608.9863132	-0.484829755	-5.707794684	1.14E-08	1.79E-07	-1.399
MCM2	377.4593303	-0.582316177	-5.702940092	1.18E-08	1.84E-07	-1.497
KCNK15	78.39573436	-1.04441134	-5.698052612	1.21E-08	1.89E-07	-2.063
CDCP1	159.9020593	0.788323467	5.697819426	1.21E-08	1.89E-07	1.727

PPP1R7	1379.313727	-0.366370577	-5.696001809	1.23E-08	1.90E-07	-1.289
CD55	353.9648448	-0.580980085	-5.694336141	1.24E-08	1.92E-07	-1.496
GLUL	1297.582146	-0.353716028	-5.68538619	1.31E-08	2.02E-07	-1.278
TUBA4A	1032.797205	0.424797149	5.68351892	1.32E-08	2.04E-07	1.342
CMIP	655.2617775	0.452434562	5.677810038	1.36E-08	2.11E-07	1.368
NSMAF	1287.246093	-0.368426468	-5.674639234	1.39E-08	2.14E-07	-1.291
ENOPH1	877.0972873	-0.409614736	-5.67246966	1.41E-08	2.17E-07	-1.328
ACTG2	135.9819354	0.86004061	5.668983903	1.44E-08	2.21E-07	1.815
RP11-838N2.4	22.71313168	-1.357442701	-5.665221337	1.47E-08	2.26E-07	-2.562
MYCT1	67.78259199	1.061538149	5.662765492	1.49E-08	2.29E-07	2.087
PARP9	286.5740367	-0.633960763	-5.661675342	1.50E-08	2.30E-07	-1.552
GPX3	668.1338348	-0.498550792	-5.658119258	1.53E-08	2.35E-07	-1.413
FRRS1	153.1689684	-0.850976754	-5.650899441	1.60E-08	2.44E-07	-1.804
FNDC1	4625.427728	-0.389411466	-5.649770882	1.61E-08	2.45E-07	-1.310
MALL	137.1071599	0.878290163	5.649156076	1.61E-08	2.46E-07	1.838
STRIP2	188.5693491	-0.777913445	-5.643014726	1.67E-08	2.55E-07	-1.715
RP5-848E13.1	524.0772081	-0.506665022	-5.640165022	1.70E-08	2.58E-07	-1.421
CEP55	165.7022515	-0.769810317	-5.632615934	1.77E-08	2.70E-07	-1.705
AC073346.2	535.2383936	-0.490270112	-5.631152212	1.79E-08	2.72E-07	-1.405
RP11-142G7.1	218.9691547	-0.682746113	-5.630291931	1.80E-08	2.73E-07	-1.605
PTHLH	48.46969325	1.167619203	5.62902611	1.81E-08	2.74E-07	2.246
TP53I3	856.9201593	-0.408522726	-5.628771117	1.81E-08	2.74E-07	-1.327
AC005251.3	1832.540302	-0.367575228	-5.624847872	1.86E-08	2.80E-07	-1.290
AFF2	121.8713455	-0.879719167	-5.623969054	1.87E-08	2.82E-07	-1.840
SUSD1	338.8763397	0.597777539	5.617311789	1.94E-08	2.92E-07	1.513
ANKRD44	438.4102193	-0.53312264	-5.607553238	2.05E-08	3.08E-07	-1.447
GSTO1	2041.85116	-0.377070799	-5.606797498	2.06E-08	3.09E-07	-1.299
TAGLN2P1	1007.356317	0.404628787	5.605330388	2.08E-08	3.12E-07	1.324
MIR22HG	1453.634246	0.401157823	5.602983809	2.11E-08	3.16E-07	1.321
FAM219A	1385.290475	0.364370882	5.598546265	2.16E-08	3.23E-07	1.287
NCEH1	1202.683658	0.384406262	5.595717299	2.20E-08	3.28E-07	1.305
PTGIR	438.1427612	0.517812429	5.584967957	2.34E-08	3.48E-07	1.432
EFS	357.2724832	0.587974672	5.582530248	2.37E-08	3.53E-07	1.503
SREBF1	1354.290823	-0.340619006	-5.573211216	2.50E-08	3.71E-07	-1.266
FBXO10	633.5400881	-0.44765879	-5.572519979	2.51E-08	3.72E-07	-1.364
EYA1	77.45272666	-1.012817994	-5.571575923	2.52E-08	3.74E-07	-2.018
HDAC9	251.9977465	-0.650199929	-5.571039953	2.53E-08	3.74E-07	-1.569
TVP23B	1627.140121	0.322764511	5.569463664	2.56E-08	3.77E-07	1.251
ATF3	154.7541973	0.781014689	5.569062575	2.56E-08	3.78E-07	1.718
PGDP1	221.9128691	-0.672110979	-5.56683648	2.59E-08	3.82E-07	-1.593
CKB	373.3996968	0.555628271	5.561575344	2.67E-08	3.93E-07	1.470

STEAP1	229.7791429	-0.67946937	-5.556992287	2.74E-08	4.02E-07	-1.602
DNM3OS	251.4735862	-0.684304668	-5.556957744	2.75E-08	4.02E-07	-1.607
RP11-77G22.3	294.802428	-0.650511691	-5.554332512	2.79E-08	4.07E-07	-1.570
APLN	66.79405293	1.056910695	5.553321669	2.80E-08	4.09E-07	2.080
RP11-280F2.2	335.8148902	0.589278839	5.553249958	2.80E-08	4.09E-07	1.504
SAMD8	1578.762401	0.390536946	5.549679555	2.86E-08	4.17E-07	1.311
SLC43A1	202.4028782	-0.749516253	-5.547031393	2.91E-08	4.23E-07	-1.681
FAM210B	1377.51462	-0.345394368	-5.546845671	2.91E-08	4.23E-07	-1.270
RBM43	345.6243162	-0.571892755	-5.546445006	2.92E-08	4.23E-07	-1.486
RP11-366M4.7	1238.057399	-0.381651462	-5.545677721	2.93E-08	4.25E-07	-1.303
RNF24	1560.941646	0.368107779	5.533046377	3.15E-08	4.56E-07	1.291
WIPF2	1552.441581	0.350124087	5.531888479	3.17E-08	4.58E-07	1.275
VAMP2	868.2374909	0.452229496	5.53119917	3.18E-08	4.60E-07	1.368
NHLRC3	361.3154216	-0.562523865	-5.528221968	3.23E-08	4.67E-07	-1.477
TMEM60	264.0201021	-0.627342382	-5.526854344	3.26E-08	4.70E-07	-1.545
OSBPL2	1323.985356	0.343763001	5.518245117	3.42E-08	4.94E-07	1.269
IFITM1	356.5080811	-0.614464372	-5.508855053	3.61E-08	5.20E-07	-1.531
ENPP1	1260.004907	0.343427555	5.499300503	3.81E-08	5.49E-07	1.269
TMEM147	1310.904288	-0.432269395	-5.491605208	3.98E-08	5.72E-07	-1.349
C4orf46	267.5084305	-0.616382553	-5.488422795	4.06E-08	5.81E-07	-1.533
MYEF2	285.811521	0.611121511	5.485786102	4.12E-08	5.89E-07	1.527
SLC22A17	720.7155186	0.425136376	5.484493086	4.15E-08	5.93E-07	1.343
NAMPT	675.7514393	-0.427339646	-5.483384613	4.17E-08	5.96E-07	-1.345
KCNJ15	76.36002398	-1.012203991	-5.478552616	4.29E-08	6.12E-07	-2.017
PALMD	503.7728321	0.485151724	5.47680813	4.33E-08	6.17E-07	1.400
PLA2G4A	303.7235058	-0.597676636	-5.46827957	4.54E-08	6.47E-07	-1.513
GBAP1	400.6054828	-0.568778376	-5.464545733	4.64E-08	6.60E-07	-1.483
HPS1	1747.22035	-0.387871119	-5.453499571	4.94E-08	7.02E-07	-1.308
TCAF2	102.7550243	0.891415145	5.453162635	4.95E-08	7.03E-07	1.855
STMN1	897.4773806	-0.398777739	-5.448346626	5.08E-08	7.20E-07	-1.318
VOPP1	1559.724404	0.363780512	5.435488129	5.46E-08	7.72E-07	1.287
MOXD1	574.5185042	0.45395104	5.433018219	5.54E-08	7.82E-07	1.370
MPV17	1277.72067	-0.370728388	-5.430978718	5.60E-08	7.90E-07	-1.293
MBNL1-AS1	811.0796064	0.414337431	5.429364739	5.66E-08	7.97E-07	1.333
FMN2	1514.946614	0.337708675	5.424489736	5.81E-08	8.16E-07	1.264
MAX	1349.332747	-0.344923466	-5.419393949	5.98E-08	8.38E-07	-1.270
PTGS1	111.3796847	0.859005183	5.41689932	6.06E-08	8.49E-07	1.814
MIER2	862.8991343	0.405414667	5.415306243	6.12E-08	8.56E-07	1.324
LAMTOR4	757.2824655	-0.44119338	-5.413504148	6.18E-08	8.64E-07	-1.358
GNG11	652.6285941	-0.439879354	-5.405845319	6.45E-08	9.00E-07	-1.356
AMPD3	167.7053541	0.753768087	5.401682828	6.60E-08	9.19E-07	1.686

TLDC1	1133.526906	0.35163672	5.39145247	6.99E-08	9.72E-07	1.276
BAALC	427.3290101	-0.532205675	-5.389999369	7.05E-08	9.79E-07	-1.446
RP11-334A14.2	457.7385395	0.524324012	5.386041241	7.20E-08	1.00E-06	1.438
KCNS3	65.67621427	-1.0244316	-5.381679234	7.38E-08	1.02E-06	-2.034
NRP2	2314.381527	-0.344612574	-5.375771581	7.63E-08	1.06E-06	-1.270
MEX3B	395.7874254	-0.574867815	-5.370186356	7.87E-08	1.09E-06	-1.490
DOCK5	3437.737717	0.328337816	5.364106944	8.14E-08	1.12E-06	1.256
RP5-1043F6.1	814.4121389	0.529290894	5.35814512	8.41E-08	1.16E-06	1.443
MT-ND5	45050.30531	-0.32614351	-5.356245212	8.50E-08	1.17E-06	-1.254
SMAD7	2006.536872	-0.342557113	-5.355567463	8.53E-08	1.17E-06	-1.268
UBE2L6	716.9608727	-0.423061488	-5.355706887	8.52E-08	1.17E-06	-1.341
MICALL1	1469.638447	0.32553968	5.350263438	8.78E-08	1.21E-06	1.253
FGF5	1215.322515	0.354565527	5.344578772	9.06E-08	1.24E-06	1.279
EVI2B	29.0844264	-1.224378148	-5.341189002	9.23E-08	1.27E-06	-2.337
PODNL1	680.2565733	-0.455597972	-5.337225577	9.44E-08	1.29E-06	-1.371
RP11-278C7.1	566.0590133	-0.494293714	-5.321562591	1.03E-07	1.40E-06	-1.409
TBC1D12	635.2520317	-0.451470476	-5.319305971	1.04E-07	1.42E-06	-1.367
SH3BP5L	1509.732852	0.329731341	5.315552246	1.06E-07	1.45E-06	1.257
UBA7	574.9534003	-0.487840788	-5.313947253	1.07E-07	1.46E-06	-1.402
PLEKHA2	1114.366525	0.349099562	5.311492685	1.09E-07	1.48E-06	1.274
COA6	284.8949468	-0.584715691	-5.307809668	1.11E-07	1.50E-06	-1.500
RP11-173B14.5	773.876629	0.45268289	5.300360252	1.16E-07	1.56E-06	1.369
SGCE	1085.100817	0.365086823	5.293550698	1.20E-07	1.62E-06	1.288
DMPK	1864.843183	0.343287523	5.294058403	1.20E-07	1.62E-06	1.269
CPNE7	290.1405551	-0.585559921	-5.293818206	1.20E-07	1.62E-06	-1.501
FMN1	454.0416068	0.510595008	5.289818212	1.22E-07	1.65E-06	1.425
EGLN1	1997.216305	0.322160616	5.288300196	1.23E-07	1.66E-06	1.250
ALDH4A1	794.4824517	-0.460374648	-5.286848584	1.24E-07	1.67E-06	-1.376
NOL8	649.1536252	-0.427814466	-5.280887839	1.29E-07	1.73E-06	-1.345
RP11-398K22.9	896.1466518	-0.384197927	-5.278646852	1.30E-07	1.74E-06	-1.305
EPS8L2	630.2504854	0.423765014	5.278312052	1.30E-07	1.74E-06	1.341
DARS2	412.414559	-0.539001345	-5.278247178	1.30E-07	1.74E-06	-1.453
RIC1	1746.070349	0.348119498	5.271951682	1.35E-07	1.80E-06	1.273
CYTIP	53.42273805	1.057113526	5.266740631	1.39E-07	1.85E-06	2.081
C12orf49	1320.979212	-0.336376614	-5.264500383	1.41E-07	1.87E-06	-1.263
ATG3	1343.405818	-0.327279926	-5.263299918	1.41E-07	1.88E-06	-1.255
AC097639.4	40.24917034	1.150296615	5.262681308	1.42E-07	1.88E-06	2.220
ASB1	1364.915504	0.329846865	5.258530866	1.45E-07	1.93E-06	1.257
ADA	242.4868436	-0.635795775	-5.254269659	1.49E-07	1.97E-06	-1.554
RGL1	1130.412002	-0.352176673	-5.249245156	1.53E-07	2.02E-06	-1.276
TBC1D2	1035.614039	-0.349530325	-5.24585197	1.56E-07	2.05E-06	-1.274

TAF13	1343.465908	0.325917979	5.242313599	1.59E-07	2.09E-06	1.253
SH2D4A	1242.199635	0.338239183	5.242100437	1.59E-07	2.09E-06	1.264
GRPEL1	893.0397069	0.381867684	5.237303476	1.63E-07	2.14E-06	1.303
AKAP6	228.3836684	0.684892889	5.236761817	1.63E-07	2.15E-06	1.608
TTC13	647.9240086	0.422497676	5.23400413	1.66E-07	2.18E-06	1.340
ZNF185	148.6511135	0.757250225	5.231969689	1.68E-07	2.20E-06	1.690
NSDHL	643.6665642	-0.474234234	-5.229990221	1.70E-07	2.22E-06	-1.389
S100A13	1947.787426	-0.341524808	-5.228444374	1.71E-07	2.24E-06	-1.267
SUCO	1587.421517	0.347989803	5.223974741	1.75E-07	2.28E-06	1.273
STARD5	145.4946786	-0.775170209	-5.221492358	1.77E-07	2.31E-06	-1.711
FAM117A	151.2978158	-0.733755854	-5.217240308	1.82E-07	2.37E-06	-1.663
DGKA	962.6758279	0.378258459	5.214304147	1.85E-07	2.40E-06	1.300
EGR2	164.4611342	0.740185153	5.212182694	1.87E-07	2.43E-06	1.670
ZNF609	1568.875538	0.322055034	5.209362763	1.89E-07	2.46E-06	1.250
NFKBIZ	645.7642539	0.488629823	5.203364831	1.96E-07	2.54E-06	1.403
BIRC5	228.8671627	-0.630777757	-5.201619789	1.98E-07	2.56E-06	-1.548
SHB	314.7311471	0.552165462	5.198868464	2.01E-07	2.59E-06	1.466
HMMR	96.64231835	-0.868402305	-5.194653271	2.05E-07	2.65E-06	-1.826
CDR2L	1604.176848	0.332467557	5.193741839	2.06E-07	2.66E-06	1.259
DCUN1D3	819.0342269	0.379078147	5.190447293	2.10E-07	2.70E-06	1.301
ASMTL	490.5831623	0.492328766	5.188193143	2.12E-07	2.74E-06	1.407
NPDC1	2117.85409	0.322089214	5.184929505	2.16E-07	2.78E-06	1.250
CFLAR	1547.749062	0.334276065	5.184016722	2.17E-07	2.79E-06	1.261
POM121C	1149.635077	0.361140221	5.179940476	2.22E-07	2.85E-06	1.284
CLDN23	29.09942628	-1.186710257	-5.179059666	2.23E-07	2.86E-06	-2.276
G0S2	115.4232375	-0.804469824	-5.178591932	2.24E-07	2.87E-06	-1.747
TSPAN31	807.8399907	0.398502704	5.165137348	2.40E-07	3.08E-06	1.318
EVI5L	1139.097787	0.33879199	5.163723143	2.42E-07	3.10E-06	1.265
COA7	559.2752891	-0.431087806	-5.154098497	2.55E-07	3.26E-06	-1.348
CENPT	1062.997425	0.396702227	5.14827047	2.63E-07	3.36E-06	1.316
COL8A2	981.7981708	0.370819466	5.147329121	2.64E-07	3.37E-06	1.293
TNFAIP6	213.8199419	-0.710551172	-5.145844733	2.66E-07	3.40E-06	-1.636
STARD10	179.2251017	-0.7136797	-5.145925441	2.66E-07	3.40E-06	-1.640
RP3-417G15.1	1282.685386	-0.370213758	-5.14418009	2.69E-07	3.42E-06	-1.293
SLC5A6	311.6723673	-0.564225227	-5.141691359	2.72E-07	3.47E-06	-1.479
AKR1C2	291.8051784	-0.618784265	-5.138998221	2.76E-07	3.51E-06	-1.536
SYNGR2	850.5582316	0.37962443	5.138562577	2.77E-07	3.51E-06	1.301
HSPE1	797.7086492	-0.455248764	-5.136708223	2.80E-07	3.55E-06	-1.371
TBCK	785.1063731	-0.402036364	-5.13476578	2.82E-07	3.58E-06	-1.321
TRPM7	2139.681494	-0.365751944	-5.133783389	2.84E-07	3.59E-06	-1.289
ACOT13	554.143029	-0.445785532	-5.125397856	2.97E-07	3.75E-06	-1.362

REPIN1	1130.138652	-0.349176819	-5.12512231	2.97E-07	3.76E-06	-1.274
RSRP1	508.6057053	-0.466069809	-5.119798865	3.06E-07	3.86E-06	-1.381
ERV3-1	253.9257064	-0.59770019	-5.119723315	3.06E-07	3.86E-06	-1.513
LRRC15	482.446808	-0.475006363	-5.111204811	3.20E-07	4.03E-06	-1.390
WI2-80423F1.1	840.7540885	-0.421811126	-5.109614737	3.23E-07	4.06E-06	-1.340
KIF2C	131.0288477	-0.768787064	-5.106896592	3.27E-07	4.11E-06	-1.704
KLF2	1178.444284	0.395942749	5.103876806	3.33E-07	4.17E-06	1.316
AP5Z1	970.5103162	-0.379768825	-5.103176678	3.34E-07	4.18E-06	-1.301
SMAP2	946.8179662	0.354318685	5.102800998	3.35E-07	4.19E-06	1.278
TDG	579.5219791	0.424097964	5.1009033	3.38E-07	4.22E-06	1.342
ZFYVE26	840.7354447	-0.407403916	-5.099958156	3.40E-07	4.24E-06	-1.326
RASSF1	783.9497599	0.393623322	5.099783276	3.40E-07	4.24E-06	1.314
KAZN	555.2742867	-0.455450546	-5.098001596	3.43E-07	4.28E-06	-1.371
ELMSAN1	795.855022	0.389732216	5.097789779	3.44E-07	4.28E-06	1.310
ABHD14B	1041.727425	-0.357148435	-5.096430834	3.46E-07	4.31E-06	-1.281
SERTAD1	661.0986084	0.404928833	5.095357148	3.48E-07	4.33E-06	1.324
RP11-320L13.2	2315.315849	-0.329093364	-5.093454047	3.52E-07	4.37E-06	-1.256
PQLC2	327.304463	-0.558433214	-5.082707445	3.72E-07	4.61E-06	-1.473
MLST8	765.9694137	-0.397158138	-5.07979593	3.78E-07	4.68E-06	-1.317
ZDHHC17	912.2613262	0.372206354	5.078481078	3.80E-07	4.70E-06	1.294
RP11-415I12.6	1394.998359	-0.364830384	-5.077360976	3.83E-07	4.73E-06	-1.288
CDCA7L	262.1792971	-0.580146806	-5.076891948	3.84E-07	4.74E-06	-1.495
KRT8	110.0788462	0.829626714	5.067658516	4.03E-07	4.97E-06	1.777
GFPT2	192.3734733	0.662086027	5.066236237	4.06E-07	5.00E-06	1.582
RP11-393M18.2	139.1363883	-0.797580709	-5.064213849	4.10E-07	5.05E-06	-1.738
HNMT	346.901734	-0.508880955	-5.06336178	4.12E-07	5.07E-06	-1.423
TBC1D8B	853.7845316	0.372509812	5.061910329	4.15E-07	5.11E-06	1.295
TNIK	295.9927042	-0.554161273	-5.056044951	4.28E-07	5.26E-06	-1.468
FBN1	112886.7691	0.609742115	5.05406762	4.32E-07	5.31E-06	1.526
RP1-278E11.3	2084.292217	-0.358733094	-5.052100645	4.37E-07	5.36E-06	-1.282
PLXDC2	1572.780684	-0.360547101	-5.051874986	4.37E-07	5.36E-06	-1.284
RGS5	117.7548227	0.804758008	5.050813727	4.40E-07	5.38E-06	1.747
ADRA1D	71.2934346	0.942544665	5.042314663	4.60E-07	5.61E-06	1.922
SNX16	240.885139	-0.598534091	-5.035157625	4.77E-07	5.81E-06	-1.514
MARCKSL1	981.08578	-0.365660111	-5.032050675	4.85E-07	5.90E-06	-1.288
RP11-327L3.5	1701.625038	-0.340674529	-5.026792729	4.99E-07	6.05E-06	-1.266
CALB2	27.39104394	-1.161746015	-5.024659932	5.04E-07	6.12E-06	-2.237
ASNA1	1359.557566	-0.358707751	-5.022569517	5.10E-07	6.18E-06	-1.282
TMEM55B	676.4588421	-0.452200065	-5.019587161	5.18E-07	6.27E-06	-1.368
PLEKHO1	1116.907878	-0.361856499	-5.012783058	5.36E-07	6.48E-06	-1.285
RILPL2	891.7360157	-0.36931955	-5.012063618	5.38E-07	6.50E-06	-1.292

COL4A4	180.6450705	0.664068537	5.011556274	5.40E-07	6.51E-06	1.585
ZNF503	979.806941	-0.344709739	-5.010227253	5.44E-07	6.55E-06	-1.270
CGREF1	1022.878446	0.360720894	5.008896257	5.47E-07	6.60E-06	1.284
RAB9A	489.3972788	-0.46174009	-5.007035226	5.53E-07	6.65E-06	-1.377
ERAP1	1756.542439	-0.330905146	-5.005622894	5.57E-07	6.69E-06	-1.258
SEPP1	67.89475141	-0.931935798	-5.003792808	5.62E-07	6.75E-06	-1.908
SEPHS2	1385.100323	-0.337913411	-5.001983437	5.67E-07	6.81E-06	-1.264
SYTL2	426.244906	-0.47606261	-5.002074843	5.67E-07	6.81E-06	-1.391
INPP5A	727.6869033	0.397168178	4.998573723	5.78E-07	6.92E-06	1.317
RPL22L1	790.7737561	-0.394243341	-4.993542462	5.93E-07	7.09E-06	-1.314
RP11-354P17.9	278.2600422	0.573504784	4.991692225	5.99E-07	7.15E-06	1.488
SLC46A1	630.8952152	0.403513718	4.986216806	6.16E-07	7.34E-06	1.323
RPS27P27	431.6269175	-0.489398208	-4.982053521	6.29E-07	7.49E-06	-1.404
SLC16A6	32.82587003	-1.113128182	-4.981029617	6.32E-07	7.51E-06	-2.163
COMMD7	770.8156156	-0.384504726	-4.976707741	6.47E-07	7.67E-06	-1.305
HADH	481.2954717	-0.447820233	-4.975210729	6.52E-07	7.73E-06	-1.364
GOLGA8A	748.9965136	0.472664204	4.974138208	6.55E-07	7.76E-06	1.388
ADM2	1011.342564	0.335893761	4.969894424	6.70E-07	7.92E-06	1.262
LRFN4	560.094045	-0.43299828	-4.969621512	6.71E-07	7.92E-06	-1.350
JADE1	751.8243228	0.377985759	4.967992331	6.76E-07	7.98E-06	1.300
SLFN5	5298.625449	-0.350906606	-4.967395838	6.79E-07	8.00E-06	-1.275
DOCK11	300.0348485	-0.544906659	-4.963359274	6.93E-07	8.14E-06	-1.459
AKR1A1	1616.769979	-0.33038039	-4.96110424	7.01E-07	8.22E-06	-1.257
NCAM2	97.44361401	-0.841390984	-4.946145322	7.57E-07	8.83E-06	-1.792
HPS3	803.5888654	-0.361739814	-4.925943607	8.40E-07	9.75E-06	-1.285
PAPPA2	1381.906653	0.767881839	4.916610182	8.81E-07	1.02E-05	1.703
CD2AP	1222.807459	0.338853898	4.915949688	8.84E-07	1.02E-05	1.265
TRANK1	353.9213736	-0.50497589	-4.91598569	8.83E-07	1.02E-05	-1.419
SNCG	186.2753845	0.655312392	4.913877613	8.93E-07	1.03E-05	1.575
CNRIP1	816.1768733	-0.354875709	-4.913984541	8.92E-07	1.03E-05	-1.279
FZD5	90.14917061	-0.834133703	-4.912372398	9.00E-07	1.04E-05	-1.783
C1QTNF1	326.6519896	-0.559539514	-4.911243961	9.05E-07	1.05E-05	-1.474
ZNF274	442.3417332	0.463779073	4.900672469	9.55E-07	1.10E-05	1.379
SEMA3F	1064.288596	-0.349362559	-4.897272652	9.72E-07	1.12E-05	-1.274
PSMB8	503.7151421	-0.44289913	-4.893805486	9.89E-07	1.14E-05	-1.359
FAM118B	474.3745981	-0.435841967	-4.891947036	9.98E-07	1.15E-05	-1.353
RP11-268J15.5	59.10497535	0.968936919	4.890216771	1.01E-06	1.16E-05	1.957
DOCK4	553.6796956	-0.465314632	-4.888088851	1.02E-06	1.17E-05	-1.381
ZNF521	85.70835039	-0.867912574	-4.883098896	1.04E-06	1.19E-05	-1.825
TMEM144	79.20574449	-0.872883524	-4.882414257	1.05E-06	1.20E-05	-1.831
HGF	868.3723445	-0.403435958	-4.881904798	1.05E-06	1.20E-05	-1.323

SLC2A5	129.3102079	0.760743069	4.879959207	1.06E-06	1.21E-05	1.694
ADGRA3	479.6622885	-0.448712994	-4.879258751	1.06E-06	1.22E-05	-1.365
KLF16	867.5718442	0.362212254	4.875015047	1.09E-06	1.24E-05	1.285
SLC2A12	229.1836293	-0.599268879	-4.858723494	1.18E-06	1.34E-05	-1.515
CD9	800.5972088	-0.408418735	-4.858412553	1.18E-06	1.35E-05	-1.327
OSGIN2	602.5279443	-0.412694691	-4.855069882	1.20E-06	1.37E-05	-1.331
SLC9A3R1	412.9237528	-0.498754082	-4.844541032	1.27E-06	1.44E-05	-1.413
FAM89B	1372.249421	0.326335047	4.840506245	1.30E-06	1.46E-05	1.254
SLC25A13	408.5824912	-0.490767481	-4.837242142	1.32E-06	1.48E-05	-1.405
RP11-452N17.1	410.6815585	-0.469707347	-4.833362246	1.34E-06	1.51E-05	-1.385
RP11-498J9.1	182.1912914	0.658187016	4.832355788	1.35E-06	1.52E-05	1.578
EAF1	806.6386375	0.367709697	4.813578858	1.48E-06	1.66E-05	1.290
TMEM178B	415.3353437	0.510582684	4.812580241	1.49E-06	1.67E-05	1.425
LIMS2	1241.412315	0.331758559	4.809392459	1.51E-06	1.69E-05	1.259
MIGA1	938.5767792	0.376560051	4.794996701	1.63E-06	1.81E-05	1.298
RNF13	694.0494882	-0.368916108	-4.788824109	1.68E-06	1.87E-05	-1.291
RORA	559.9454886	0.425097945	4.787320775	1.69E-06	1.88E-05	1.343
CEBPB	1302.260992	-0.362177618	-4.785754116	1.70E-06	1.89E-05	-1.285
DHRS3	1159.253471	-0.333819122	-4.784624828	1.71E-06	1.90E-05	-1.260
TNS2	1261.185457	-0.333542838	-4.780381262	1.75E-06	1.94E-05	-1.260
AP001422.3	30.03493729	-1.082448972	-4.779661823	1.76E-06	1.94E-05	-2.118
HSD11B1	28.47576925	-1.094740252	-4.778512822	1.77E-06	1.95E-05	-2.136
TRAPPC2L	794.6443505	-0.432780848	-4.773745043	1.81E-06	2.00E-05	-1.350
E2F7	443.5880761	-0.467052712	-4.773699431	1.81E-06	2.00E-05	-1.382
ARVCF	618.997238	-0.395062025	-4.772166974	1.82E-06	2.01E-05	-1.315
NDNF	127.9605589	0.72621062	4.769201544	1.85E-06	2.04E-05	1.654
GATA6	869.9062086	0.382541098	4.769026353	1.85E-06	2.04E-05	1.304
RP11-589N15.2	12.41158245	-1.158631065	-4.766869015	1.87E-06	2.06E-05	-2.232
CDC20	156.7647748	-0.721597848	-4.759079761	1.94E-06	2.14E-05	-1.649
AC009945.3	657.4420764	-0.392761705	-4.755585739	1.98E-06	2.17E-05	-1.313
RP11-443P15.2	57.42305089	-0.957397422	-4.75339977	2.00E-06	2.19E-05	-1.942
CXCL16	360.3355204	-0.496527258	-4.751060516	2.02E-06	2.22E-05	-1.411
CAMK2G	997.107311	0.337361319	4.749494308	2.04E-06	2.23E-05	1.263
ATP7B	432.2209444	0.45458522	4.748570608	2.05E-06	2.24E-05	1.370
BRSK1	233.1775086	0.577969999	4.748203426	2.05E-06	2.24E-05	1.493
BTBD3	504.430338	0.411179798	4.744048161	2.09E-06	2.29E-05	1.330
EEF1A2	58.43657971	0.935772847	4.742482148	2.11E-06	2.30E-05	1.913
SEMA3A	341.091853	0.495759134	4.737077191	2.17E-06	2.36E-05	1.410
BUB1B	145.3256919	-0.692188101	-4.735677035	2.18E-06	2.38E-05	-1.616
EFCAB7	137.3513153	-0.693460798	-4.734911792	2.19E-06	2.39E-05	-1.617
NUAK2	171.4666127	0.662497734	4.731389151	2.23E-06	2.43E-05	1.583

MGARP	242.0788917	0.548992152	4.72542547	2.30E-06	2.50E-05	1.463
MAPRE3	1020.711563	0.33602167	4.723081609	2.32E-06	2.52E-05	1.262
CHKA	236.1182794	-0.580820004	-4.722964529	2.32E-06	2.52E-05	-1.496
RP11-370B11.1	541.3450185	0.497861082	4.718918101	2.37E-06	2.56E-05	1.412
TMEM158	497.7168506	0.452620342	4.717576503	2.39E-06	2.58E-05	1.369
FAM84A	67.94830644	-0.895146989	-4.715988218	2.41E-06	2.60E-05	-1.860
EPN2	937.2322308	0.336354517	4.714717742	2.42E-06	2.61E-05	1.263
COMMD4	694.6827782	-0.422662996	-4.714802271	2.42E-06	2.61E-05	-1.340
PBK	110.5579486	-0.764092419	-4.710560414	2.47E-06	2.66E-05	-1.698
PXYLP1	236.8476097	0.563224871	4.707722126	2.51E-06	2.70E-05	1.478
ARPIN	577.5173575	-0.390811499	-4.704264697	2.55E-06	2.74E-05	-1.311
TLR3	126.5157166	-0.715840558	-4.702746584	2.57E-06	2.75E-05	-1.642
ZFP36	693.2957677	0.371686916	4.700593376	2.59E-06	2.78E-05	1.294
FAM131B	160.8805254	0.646731759	4.698998821	2.61E-06	2.80E-05	1.566
DMD	1250.734178	0.322114923	4.696583393	2.65E-06	2.83E-05	1.250
RP4-773A18.4	138.9902921	0.741243846	4.695956819	2.65E-06	2.84E-05	1.672
RP11-119H12.2	644.9357513	-0.371202623	-4.694640648	2.67E-06	2.86E-05	-1.293
PUS7L	732.0207662	0.40352792	4.691958004	2.71E-06	2.89E-05	1.323
RP11-288G3.4	119.3038225	-0.736036697	-4.691380709	2.71E-06	2.89E-05	-1.666
LRRN3	282.9226322	0.518271072	4.690971738	2.72E-06	2.90E-05	1.432
CYSTM1	993.2764328	-0.357982017	-4.688867216	2.75E-06	2.92E-05	-1.282
DDIT4L	55.40363541	-0.961018257	-4.688286812	2.76E-06	2.93E-05	-1.947
AC016722.2	101.8303438	0.770542889	4.686537754	2.78E-06	2.95E-05	1.706
LAMA3	784.1944658	-0.41199522	-4.68169437	2.85E-06	3.02E-05	-1.331
NSA2	1020.954266	-0.339474119	-4.679832148	2.87E-06	3.04E-05	-1.265
PIFO	22.10209393	-1.095927794	-4.67986162	2.87E-06	3.04E-05	-2.138
KAT6B	861.6863629	0.365512495	4.678065838	2.90E-06	3.07E-05	1.288
SLC22A23	718.7123189	0.365489364	4.677082199	2.91E-06	3.08E-05	1.288
VPS11	884.7864988	-0.375517747	-4.674700092	2.94E-06	3.11E-05	-1.297
TBC1D8	138.6242421	-0.687768498	-4.673334981	2.96E-06	3.13E-05	-1.611
NSMCE2	329.6770322	-0.494603751	-4.669316017	3.02E-06	3.19E-05	-1.409
AC079780.3	162.7697076	0.660867558	4.667497852	3.05E-06	3.21E-05	1.581
DHRS9	22.98179229	1.087628128	4.665340921	3.08E-06	3.24E-05	2.125
LAMTOR3	850.4285137	-0.346761499	-4.663166374	3.11E-06	3.28E-05	-1.272
TMEM138	689.1717348	-0.39799899	-4.662725187	3.12E-06	3.28E-05	-1.318
BEX4	63.76683463	-0.880709319	-4.657098173	3.21E-06	3.37E-05	-1.841
ST3GAL6	37.42389367	-1.01548694	-4.652668517	3.28E-06	3.44E-05	-2.022
RTKN2	191.2675303	-0.643437362	-4.651622421	3.29E-06	3.45E-05	-1.562
RTN4RL1	140.6968836	-0.67433718	-4.649228405	3.33E-06	3.49E-05	-1.596
ARL2	668.6430455	-0.365429532	-4.64308482	3.43E-06	3.59E-05	-1.288
MAPK7	765.1905912	0.345563575	4.641895656	3.45E-06	3.60E-05	1.271

CGNL1	174.2264248	-0.632474831	-4.636887927	3.54E-06	3.69E-05	-1.550
CCNB2	116.9545351	-0.746396306	-4.63396576	3.59E-06	3.74E-05	-1.678
HSD17B7	73.7385749	-0.841211439	-4.633632727	3.59E-06	3.74E-05	-1.792
ACOX3	411.3147352	0.448937959	4.632981866	3.60E-06	3.75E-05	1.365
REEP2	156.3156562	0.645815295	4.632815149	3.61E-06	3.75E-05	1.565
TLE1	317.9136762	-0.509664993	-4.625930668	3.73E-06	3.87E-05	-1.424
CLCN5	557.89181	-0.422370532	-4.62288764	3.78E-06	3.92E-05	-1.340
PKD1P1	1125.903176	0.431998788	4.622608212	3.79E-06	3.92E-05	1.349
FOXC2	829.5744663	0.332270623	4.621619561	3.81E-06	3.94E-05	1.259
NR4A3	52.69004247	0.940646545	4.621264326	3.81E-06	3.94E-05	1.919
AC073115.7	63.3590574	0.895967777	4.620776133	3.82E-06	3.95E-05	1.861
MOK	609.9861603	0.403492508	4.619143443	3.85E-06	3.97E-05	1.323
LARGE	938.892654	-0.327424648	-4.617293295	3.89E-06	4.00E-05	-1.255
CCDC93	1404.941685	-0.339768083	-4.613797199	3.95E-06	4.06E-05	-1.266
TAPT1	558.9174879	0.399752695	4.612174687	3.98E-06	4.09E-05	1.319
CLDN1	269.5354495	-0.527058869	-4.610904916	4.01E-06	4.11E-05	-1.441
PSMB9	61.88121591	-0.891682343	-4.606448721	4.10E-06	4.19E-05	-1.855
AC009302.3	879.3940937	-0.433600447	-4.60256116	4.17E-06	4.27E-05	-1.351
PNPLA3	368.9258599	-0.510060208	-4.598257596	4.26E-06	4.35E-05	-1.424
CTXN1	222.5540972	0.567181126	4.597338255	4.28E-06	4.37E-05	1.482
RP11-523D22.1	249.817678	-0.564444965	-4.596430377	4.30E-06	4.38E-05	-1.479
CST6	72.37444704	0.841381076	4.595746317	4.31E-06	4.40E-05	1.792
IL32	85.8145943	-0.790847694	-4.594901079	4.33E-06	4.41E-05	-1.730
KDM7A	402.5698018	0.505414755	4.590170394	4.43E-06	4.51E-05	1.420
NSD1	1853.580744	0.325163992	4.589348658	4.45E-06	4.52E-05	1.253
AP4E1	1004.78419	0.35501571	4.588859138	4.46E-06	4.53E-05	1.279
RP11-1H8.3	245.6843823	0.557115872	4.588280693	4.47E-06	4.54E-05	1.471
HERC3	684.7759315	0.365555012	4.585974394	4.52E-06	4.59E-05	1.288
MT-CO3	60474.17344	-0.5480979	-4.584232387	4.56E-06	4.62E-05	-1.462
RPP25	54.05979078	-0.927460374	-4.582074302	4.60E-06	4.66E-05	-1.902
DUT	816.9628911	-0.335096658	-4.579543449	4.66E-06	4.72E-05	-1.261
CLK1	674.8193187	-0.408425002	-4.573955144	4.79E-06	4.84E-05	-1.327
LYPD1	874.0545395	0.335942939	4.573316183	4.80E-06	4.85E-05	1.262
JAM2	207.0298778	0.573016712	4.571730173	4.84E-06	4.88E-05	1.488
DYRK3	453.4679716	-0.435114182	-4.569082684	4.90E-06	4.94E-05	-1.352
DLX2	204.7307114	-0.576201538	-4.569055054	4.90E-06	4.94E-05	-1.491
PRSS3	64.55647688	0.866653496	4.566628293	4.96E-06	4.99E-05	1.823
SLF2	847.6438761	-0.374800252	-4.565339247	4.99E-06	5.02E-05	-1.297
DLGAP5	152.8251922	-0.658930909	-4.563211894	5.04E-06	5.07E-05	-1.579
CCDC68	69.09280835	-0.857602573	-4.561306908	5.08E-06	5.11E-05	-1.812
HIP1R	426.5331612	-0.435879076	-4.561023557	5.09E-06	5.11E-05	-1.353

CNTNAP3	249.58988	0.594635519	4.557873089	5.17E-06	5.18E-05	1.510
SLC38A5	785.5147144	0.362489476	4.557638374	5.17E-06	5.19E-05	1.286
BCAR3	1246.481739	0.329429794	4.556817331	5.19E-06	5.20E-05	1.257
BTN3A2	432.346837	-0.455395257	-4.556506661	5.20E-06	5.20E-05	-1.371
PIEZO2	791.6528262	-0.402371517	-4.551499684	5.33E-06	5.32E-05	-1.322
RPLP0P2	113.1884636	-0.712346512	-4.551310082	5.33E-06	5.33E-05	-1.638
NRSN2	1224.305842	0.333194984	4.549011598	5.39E-06	5.38E-05	1.260
PTMAP5	769.6653435	-0.365378737	-4.547395278	5.43E-06	5.42E-05	-1.288
RP11-1A15.2	153.6195927	-0.652867806	-4.545826606	5.47E-06	5.45E-05	-1.572
PSME2	648.1021331	0.442616416	4.541341048	5.59E-06	5.56E-05	1.359
DYNC1I1	304.7525983	0.492097552	4.538686649	5.66E-06	5.63E-05	1.406
DAPK2	57.97330484	-0.894613885	-4.536839986	5.71E-06	5.67E-05	-1.859
RP4-702J19.1	277.0758589	-0.502787193	-4.530817003	5.88E-06	5.82E-05	-1.417
MELK	251.6716897	-0.527091271	-4.526753944	5.99E-06	5.93E-05	-1.441
SDE2	593.8440624	0.389688806	4.526448391	6.00E-06	5.93E-05	1.310
AQP1	30.94337361	1.017873764	4.519644881	6.19E-06	6.11E-05	2.025
SV2A	303.0802924	-0.513583968	-4.518989764	6.21E-06	6.12E-05	-1.428
WASHC2C	1685.941312	-0.65156738	-4.518900243	6.22E-06	6.12E-05	-1.571
BCL2L11	99.28103508	-0.760926895	-4.517857017	6.25E-06	6.14E-05	-1.695
ODZ4	2142.101209	0.342407212	4.512468457	6.41E-06	6.28E-05	1.268
PCSK5	369.8624732	0.48324702	4.511295941	6.44E-06	6.31E-05	1.398
TRIM16	423.9147849	-0.427750229	-4.506869394	6.58E-06	6.43E-05	-1.345
GMNN	120.7450571	-0.683587672	-4.484656076	7.30E-06	7.09E-05	-1.606
PDK4	98.49279718	0.755155883	4.484181791	7.32E-06	7.10E-05	1.688
FAM199X	948.2543127	0.361249175	4.481043369	7.43E-06	7.20E-05	1.285
GLE1	553.0163745	-0.38053042	-4.480801546	7.44E-06	7.20E-05	-1.302
SETDB2	264.7902803	-0.510660587	-4.477505092	7.55E-06	7.31E-05	-1.425
CHORDC1	656.9750459	-0.359142869	-4.477262883	7.56E-06	7.31E-05	-1.283
C9orf172	139.169228	0.681216041	4.477040055	7.57E-06	7.32E-05	1.603
CD36	753.6167968	-0.342803957	-4.473986931	7.68E-06	7.41E-05	-1.268
TACC3	261.6341003	-0.533965634	-4.4670934	7.93E-06	7.63E-05	-1.448
NAGPA	592.8606697	-0.367601731	-4.462234885	8.11E-06	7.81E-05	-1.290
CLCN6	458.6886695	-0.423723248	-4.461140757	8.15E-06	7.84E-05	-1.341
C11orf31	940.3533102	-0.4106444	-4.460709511	8.17E-06	7.85E-05	-1.329
ULK3	352.5804511	-0.473261857	-4.459002879	8.23E-06	7.90E-05	-1.388
GULP1	314.309348	-0.506074794	-4.458995807	8.23E-06	7.90E-05	-1.420
PCLAF	70.87606721	-0.824881917	-4.45779743	8.28E-06	7.94E-05	-1.771
PI4KAP2	239.9669258	0.549752641	4.451869775	8.51E-06	8.16E-05	1.464
COG1	987.5029067	0.329345979	4.450097721	8.58E-06	8.22E-05	1.256
SYPL2	43.42613773	-0.938312988	-4.449248571	8.62E-06	8.25E-05	-1.916
TMEM233	73.64594763	-0.814588535	-4.443687266	8.84E-06	8.45E-05	-1.759

AC013394.2	702.9925384	0.344458632	4.442507639	8.89E-06	8.49E-05	1.270
SLC25A37	891.8177088	-0.330637309	-4.440626113	8.97E-06	8.56E-05	-1.258
BLVRB	740.5602769	-0.366149503	-4.435143181	9.20E-06	8.77E-05	-1.289
SIAH1	529.1334855	-0.391584815	-4.431254069	9.37E-06	8.92E-05	-1.312
KRT19	625.0054633	0.367945283	4.424917816	9.65E-06	9.18E-05	1.291
TBC1D4	862.2547421	0.35074594	4.424194277	9.68E-06	9.20E-05	1.275
KCNK3	64.9821607	0.840915636	4.422656964	9.75E-06	9.26E-05	1.791
RP11-40H20.2	552.0419664	-0.381320819	-4.419862298	9.88E-06	9.38E-05	-1.303
MIPEP	347.0671236	-0.455751434	-4.415913798	1.01E-05	9.54E-05	-1.371
C20orf27	422.268469	-0.415707482	-4.413290991	1.02E-05	9.64E-05	-1.334
UTP18	381.7259151	-0.435800731	-4.40973673	1.03E-05	9.79E-05	-1.353
PRR7	311.3250031	0.499784807	4.409469029	1.04E-05	9.79E-05	1.414
ARID5A	152.3745523	0.637016983	4.409185608	1.04E-05	9.80E-05	1.555
IL7	23.06577448	-1.020604087	-4.407400169	1.05E-05	9.86E-05	-2.029
RHBDL2	58.20381129	0.889729468	4.404428777	1.06E-05	9.98E-05	1.853
SCN1B	196.8338462	-0.56134917	-4.403497047	1.07E-05	0.00010022	-1.476
RP11-305E17.5	176.7839954	-0.607464962	-4.396773005	1.10E-05	0.0001031	-1.524
SLC27A4	827.3589816	-0.341092982	-4.394588249	1.11E-05	0.00010402	-1.267
DLX5	216.2256105	-0.566223345	-4.391490534	1.13E-05	0.00010538	-1.481
CYP4V2	318.6877895	-0.493679441	-4.388571273	1.14E-05	0.00010671	-1.408
CRYBB2P1	545.4601138	0.398588558	4.387632564	1.15E-05	0.00010712	1.318
NR1D2	1076.461778	-0.322556677	-4.38326419	1.17E-05	0.00010911	-1.251
RP11-598P12.1	342.6410424	-0.475088253	-4.379720377	1.19E-05	0.00011066	-1.390
SPNS1	219.8415225	-0.57074745	-4.377444891	1.20E-05	0.00011158	-1.485
VSIG10	331.1736046	-0.461013994	-4.372503431	1.23E-05	0.00011396	-1.377
CHRDL1	780.5157491	0.329565078	4.371950186	1.23E-05	0.00011418	1.257
HELZ	1273.516599	0.324083515	4.371827419	1.23E-05	0.00011419	1.252
STOML1	585.8307504	0.368169084	4.370307601	1.24E-05	0.00011486	1.291
MAN2A1	2127.560661	0.328975892	4.365576999	1.27E-05	0.00011725	1.256
ARHGEF3	285.8699302	-0.4770423	-4.363417474	1.28E-05	0.00011824	-1.392
SMIM16	36.34618072	0.944026748	4.358415719	1.31E-05	0.00012083	1.924
NAALADL2	336.6235793	0.478663057	4.356642182	1.32E-05	0.00012168	1.393
BOC	393.1391166	0.436602033	4.353554997	1.34E-05	0.00012315	1.353
SLC31A2	407.5697369	-0.431820482	-4.351021985	1.36E-05	0.00012445	-1.349
KLHL28	398.1644484	0.416746354	4.348147432	1.37E-05	0.00012582	1.335
TMEM150A	485.4206418	-0.396482649	-4.34836246	1.37E-05	0.00012582	-1.316
RP11-944L7.2	707.6379505	0.344236386	4.347751426	1.38E-05	0.00012593	1.269
RP1-167F1.1	38.32141618	0.946273727	4.344177685	1.40E-05	0.00012785	1.927
CTD-2302E22.3	107.6321651	-0.70329516	-4.344058675	1.40E-05	0.00012785	-1.628
HSD17B14	14.09349668	-1.014515648	-4.340713345	1.42E-05	0.00012968	-2.020
SLC7A7	52.97631972	-0.875393656	-4.337793192	1.44E-05	0.0001312	-1.835

RP11-422P24.9	720.9170582	-0.360920049	-4.336813752	1.45E-05	0.00013172	-1.284
EPOP	147.1776609	0.685619571	4.332709048	1.47E-05	0.000134	1.608
FAM101A	923.518309	0.324396748	4.331166121	1.48E-05	0.00013485	1.252
TXNDC5	1243.795222	0.695604574	4.329928717	1.49E-05	0.00013537	1.620
PLEKHG4	763.7590086	0.395852728	4.326798895	1.51E-05	0.00013706	1.316
PLK1	166.2501439	-0.58420097	-4.322886698	1.54E-05	0.00013929	-1.499
S1PR3	268.5457543	0.483541281	4.321735276	1.55E-05	0.00013995	1.398
VWA5A	309.8982445	-0.504214821	-4.319211589	1.57E-05	0.00014141	-1.418
TMEM255B	117.4385527	-0.671688552	-4.314864754	1.60E-05	0.000144	-1.593
ZBED8	100.4306692	-0.736642513	-4.308044885	1.65E-05	0.0001482	-1.666
AGT	44.72122357	-0.900090546	-4.30776652	1.65E-05	0.00014831	-1.866
BBC3	336.6032365	-0.442286327	-4.303280831	1.68E-05	0.00015098	-1.359
ORAI3	482.0217144	-0.411222888	-4.296675276	1.73E-05	0.00015504	-1.330
POTEE	655.797115	0.394705365	4.293455611	1.76E-05	0.00015714	1.315
ZNF84	547.656038	-0.367230749	-4.291715957	1.77E-05	0.0001583	-1.290
TCN2	212.5332549	-0.545464062	-4.29137405	1.78E-05	0.00015846	-1.459
KMT2D	1857.33878	0.344719353	4.289897772	1.79E-05	0.00015943	1.270
SEMA3B	457.3477093	0.388836283	4.287108558	1.81E-05	0.0001612	1.309
WDR12	621.8267826	-0.356018284	-4.287142951	1.81E-05	0.0001612	-1.280
LFNG	430.941145	0.40500126	4.28236307	1.85E-05	0.00016434	1.324
BOP1	863.3560144	0.337828249	4.279705772	1.87E-05	0.00016623	1.264
RP11-367E12.4	425.2583844	-0.46434921	-4.279266533	1.88E-05	0.00016639	-1.380
DNPH1	420.7528301	-0.406732082	-4.274653874	1.91E-05	0.00016961	-1.326
GALNT18	589.6779405	0.373914603	4.27214991	1.94E-05	0.00017135	1.296
RP11-286H14.4	641.5658083	-0.366204022	-4.269509491	1.96E-05	0.0001733	-1.289
WDR54	239.9973944	0.521388498	4.26282875	2.02E-05	0.00017811	1.435
MRPS25	524.8140458	-0.372259328	-4.262344559	2.02E-05	0.00017841	-1.294
CARD10	184.4829112	0.562897656	4.26054923	2.04E-05	0.00017975	1.477
RP11-492E3.2	68.48634589	0.788746889	4.25979583	2.05E-05	0.00018027	1.728
TMEM106A	341.2317172	0.45241549	4.257644266	2.07E-05	0.00018192	1.368
IL1RAP	442.138217	0.413203064	4.254713592	2.09E-05	0.00018413	1.332
PASK	69.04238328	-0.808112405	-4.248574805	2.15E-05	0.00018886	-1.751
RP11-121P12.1	82.50256785	0.736056806	4.246590564	2.17E-05	0.00019035	1.666
INPP5F	674.660362	0.350525997	4.246336104	2.17E-05	0.00019037	1.275
UTP20	495.8715434	-0.432382561	-4.242172084	2.21E-05	0.00019365	-1.349
CLCA2	149.4712214	-0.603962892	-4.24192215	2.22E-05	0.00019377	-1.520
HAGHL	151.0383947	-0.599198961	-4.235809334	2.28E-05	0.00019851	-1.515
GLA	651.9506022	-0.358199385	-4.224730557	2.39E-05	0.0002077	-1.282
BRWD1	1025.181099	0.337947025	4.222209713	2.42E-05	0.00020988	1.264
TCEAL1	306.5448029	-0.455684523	-4.222147075	2.42E-05	0.00020988	-1.371
DLG4	735.640351	0.345379834	4.219883984	2.44E-05	0.00021189	1.270

RP11-401M16.7	666.3630247	0.330302843	4.219529438	2.45E-05	0.00021198	1.257
UTP15	294.4181953	-0.45953903	-4.216153834	2.49E-05	0.000215	-1.375
EDNRA	149.3622759	-0.619162061	-4.215645237	2.49E-05	0.00021538	-1.536
MAPK13	365.7209644	0.42362956	4.213911962	2.51E-05	0.00021693	1.341
SLC27A1	544.2872822	-0.360033202	-4.212052401	2.53E-05	0.00021839	-1.283
ARHGAP27	65.30197058	-0.78427126	-4.205543541	2.60E-05	0.00022455	-1.722
STON1	460.9107673	-0.400419506	-4.203611989	2.63E-05	0.00022625	-1.320
BTG2	481.9703632	-0.383390354	-4.198990759	2.68E-05	0.00023035	-1.304
RP11-756G12.1	2027.28536	0.324195119	4.19858496	2.69E-05	0.00023064	1.252
MT-ATP6	22027.83321	-0.541312854	-4.19646976	2.71E-05	0.00023269	-1.455
TPX2	582.4775023	-0.358314504	-4.175031613	2.98E-05	0.00025472	-1.282
CLEC2B	21.33194069	-0.970612309	-4.174795971	2.98E-05	0.00025485	-1.960
FITM2	653.3587427	0.332019371	4.171656975	3.02E-05	0.00025826	1.259
TRIM21	240.5596353	-0.491725844	-4.167995563	3.07E-05	0.00026219	-1.406
TCF7	487.8773806	-0.376670612	-4.166402008	3.09E-05	0.00026364	-1.298
ANKRD33B	451.6787985	0.429683526	4.161667838	3.16E-05	0.00026864	1.347
RUNDC1	609.4295041	0.342223299	4.160887098	3.17E-05	0.00026942	1.268
SETBP1	166.5169198	-0.586808055	-4.160309531	3.18E-05	0.00026997	-1.502
RP11-259K5.2	147.8990219	-0.618655028	-4.156970151	3.22E-05	0.00027354	-1.535
RP11-704G19.1	681.6154191	-0.342314825	-4.1552186	3.25E-05	0.00027551	-1.268
COMMD3	515.8363966	-0.412677216	-4.154728123	3.26E-05	0.00027584	-1.331
FAHD1	607.0378134	-0.342443185	-4.15458427	3.26E-05	0.00027587	-1.268
RHBDF2	260.0467256	-0.467911328	-4.154016694	3.27E-05	0.00027642	-1.383
ADGRB2	512.2517015	0.360831729	4.152514213	3.29E-05	0.00027797	1.284
SAMD14	154.5898955	0.608787182	4.149927858	3.33E-05	0.00028086	1.525
NAP1L1P1	482.0537353	-0.418249198	-4.149287976	3.34E-05	0.00028137	-1.336
FAM102B	225.3868855	-0.530780928	-4.145949618	3.38E-05	0.00028509	-1.445
ELFN1	122.3280588	-0.65310181	-4.144099257	3.41E-05	0.00028726	-1.573
HRCT1	72.33447063	0.773070835	4.139702415	3.48E-05	0.00029239	1.709
RP11-175B9.3	319.3441044	-0.441584824	-4.139516966	3.48E-05	0.00029249	-1.358
RARRES1	48.79263996	0.854047878	4.138026991	3.50E-05	0.00029411	1.808
SLITRK6	69.61964396	0.823157005	4.135137441	3.55E-05	0.00029769	1.769
RP11-522H2.3	291.9316289	-0.482271622	-4.131820286	3.60E-05	0.00030158	-1.397
GAS1	755.9997185	-0.393100661	-4.129470723	3.64E-05	0.00030453	-1.313
B4GALNT4	162.7346309	0.58325642	4.12701827	3.67E-05	0.00030765	1.498
CHD6	1106.430815	0.370540782	4.123544305	3.73E-05	0.00031202	1.293
KIAA0114	152.5980471	-0.575489209	-4.122720257	3.74E-05	0.00031299	-1.490
CEP162	149.0778536	-0.618503143	-4.121274693	3.77E-05	0.00031481	-1.535
RP11-874J12.2	61.08562368	-0.791264559	-4.118658359	3.81E-05	0.00031794	-1.731
HJURP	128.2894804	-0.634352128	-4.117015221	3.84E-05	0.00031976	-1.552
MPP4	61.80459538	0.796663874	4.115023152	3.87E-05	0.00032222	1.737

LACTB2	241.4570792	-0.481091536	-4.113384056	3.90E-05	0.00032421	-1.396
LAMC2	168.4724843	0.565312852	4.100485036	4.12E-05	0.00034183	1.480
RP5-831D17.2	203.123482	-0.557697046	-4.100528304	4.12E-05	0.00034183	-1.472
RP11-600K15.1	41.20726787	0.868023394	4.094697256	4.23E-05	0.00034982	1.825
RP11-245M24.1	54.19083812	0.832539623	4.091362233	4.29E-05	0.00035455	1.781
CDC42EP2	572.705141	0.365990259	4.088115231	4.35E-05	0.00035903	1.289
RP13-77O11.2	498.0082104	-0.36987249	-4.087043796	4.37E-05	0.00036035	-1.292
ESF1	497.2294692	-0.367806109	-4.084830103	4.41E-05	0.00036345	-1.290
RP11-17A4.1	405.4494151	-0.412118063	-4.084327132	4.42E-05	0.00036407	-1.331
FOXF2	351.7905869	-0.41497077	-4.084125147	4.42E-05	0.00036421	-1.333
LMNB1	171.3885288	-0.57451625	-4.081563417	4.47E-05	0.00036773	-1.489
TCP11L1	665.3555727	0.35777063	4.080648876	4.49E-05	0.000369	1.281
AC009413.1	144.6876837	0.599174999	4.079566965	4.51E-05	0.00037055	1.515
KLHL36	471.0066428	0.379187999	4.070469416	4.69E-05	0.00038368	1.301
PBX3	364.9935118	-0.417035997	-4.070040472	4.70E-05	0.00038403	-1.335
IMPA2	165.7355596	0.554844537	4.068875951	4.72E-05	0.0003855	1.469
RP11-527H14.3	46.19748029	-0.839868883	-4.068817321	4.73E-05	0.0003855	-1.790
GFRA1	220.5580784	0.505414422	4.067703263	4.75E-05	0.00038717	1.420
CDK1	158.4717736	-0.581752471	-4.066003255	4.78E-05	0.00038945	-1.497
PARP14	488.3410677	-0.363738599	-4.058750783	4.93E-05	0.00040042	-1.287
GTPBP6	673.5556471	-0.327248965	-4.057378461	4.96E-05	0.00040241	-1.255
PCDHGA2	237.5313994	0.481818083	4.055349692	5.01E-05	0.00040572	1.397
PWWP2B	302.3815387	-0.479565255	-4.054559933	5.02E-05	0.00040691	-1.394
CNIH3	38.30040472	-0.871421611	-4.054118487	5.03E-05	0.00040749	-1.829
LAMA2	716.1660832	-0.360389012	-4.053386423	5.05E-05	0.00040857	-1.284
ATP1B1	517.2843651	0.35818638	4.052891428	5.06E-05	0.00040925	1.282
TPD52L1	634.2121932	0.326511972	4.051414657	5.09E-05	0.00041165	1.254
AAK1	569.577085	0.45234363	4.045525132	5.22E-05	0.00042095	1.368
LYPLAL1	263.959826	-0.460172584	-4.039567931	5.35E-05	0.00043139	-1.376
PLXNB3	692.4529975	0.343147964	4.038066439	5.39E-05	0.00043375	1.269
CASP1	30.45091872	-0.90124859	-4.037549299	5.40E-05	0.00043451	-1.868
PAFAH1B3	236.9873583	-0.490615908	-4.031326562	5.55E-05	0.00044453	-1.405
AC138972.1	703.5385593	-0.347782353	-4.031068754	5.55E-05	0.00044481	-1.273
C3orf58	285.2966862	0.45208921	4.029004939	5.60E-05	0.00044844	1.368
SUSD5	427.3646363	-0.371881277	-4.028936691	5.60E-05	0.00044844	-1.294
PEBP1P2	386.2685788	-0.398799541	-4.02755212	5.64E-05	0.00045026	-1.318
EBF2	212.4495935	0.526512825	4.025727277	5.68E-05	0.00045314	1.440
GK5	592.4935127	0.342534507	4.021392802	5.79E-05	0.00046092	1.268
CD3EAP	333.6400606	-0.415792388	-4.017224861	5.89E-05	0.00046893	-1.334
UNKL	598.6081269	0.332524202	4.013433703	5.98E-05	0.00047588	1.259
BUB1	145.8360473	-0.579879943	-4.012957058	6.00E-05	0.00047662	-1.495

IGSF23	14.44167723	0.931688949	4.012737931	6.00E-05	0.00047684	1.908
C8orf59	509.6223982	-0.367343793	-4.011545964	6.03E-05	0.0004786	-1.290
CIDECP	342.1941391	0.414020838	4.007496085	6.14E-05	0.00048621	1.332
NALCN	533.5111844	0.364152386	4.003521402	6.24E-05	0.00049378	1.287
CCDC85C	342.1672232	0.407545227	4.002298222	6.27E-05	0.00049611	1.326
RELT	228.2246487	0.48727232	3.997511025	6.40E-05	0.00050509	1.402
COL4A5	526.7733812	0.354683307	3.996074286	6.44E-05	0.0005077	1.279
LRRC20	113.5562334	-0.631867949	-3.993432801	6.51E-05	0.00051293	-1.550
RP11-241I20.3	608.1166313	-0.326048398	-3.989824054	6.61E-05	0.00052032	-1.254
ABCC3	500.7597501	0.349411977	3.983289399	6.80E-05	0.00053315	1.274
VEPH1	592.8002458	0.331110076	3.982768857	6.81E-05	0.00053408	1.258
RP1-186E20.2	182.6378637	0.55763826	3.981120519	6.86E-05	0.00053731	1.472
RP11-135F9.1	470.6325655	-0.371291653	-3.980793407	6.87E-05	0.0005378	-1.294
AC090602.1	878.1989976	-0.329782456	-3.979177771	6.92E-05	0.00054123	-1.257
HTR2A	105.4120532	0.636310598	3.978406167	6.94E-05	0.00054274	1.554
PRSS53	200.6585709	0.552347802	3.976859994	6.98E-05	0.00054579	1.466
NUSAP1	171.3542789	-0.550664048	-3.972771487	7.10E-05	0.00055449	-1.465
MRPL34	364.2062639	-0.400450373	-3.971927493	7.13E-05	0.00055621	-1.320
CADM1	96.64810446	-0.659789403	-3.967765274	7.25E-05	0.00056499	-1.580
EPB41L4B	91.64033834	0.688522047	3.962957763	7.40E-05	0.00057469	1.612
WDSUB1	240.8504611	-0.470722245	-3.960810633	7.47E-05	0.00057962	-1.386
SMPDL3A	263.3491586	-0.46294525	-3.958486802	7.54E-05	0.00058476	-1.378
STEAP1B	416.0722629	-0.387874337	-3.957492165	7.57E-05	0.00058694	-1.308
RP11-320N7.3	725.9899659	-0.36945598	-3.954728863	7.66E-05	0.0005935	-1.292
BMPR1B	70.42001689	-0.723700928	-3.948385177	7.87E-05	0.00060809	-1.651
KRT81	80.88110211	0.695616326	3.945354622	7.97E-05	0.00061482	1.620
DHX58	80.33018601	-0.693521213	-3.945211406	7.97E-05	0.00061482	-1.617
PAK1IP1	405.4221627	-0.37392935	-3.944738793	7.99E-05	0.00061576	-1.296
TSNARE1	112.4242277	-0.625372285	-3.937568388	8.23E-05	0.00063276	-1.543
KCNJ2	94.78224066	-0.649184337	-3.93214935	8.42E-05	0.00064605	-1.568
TNFRSF1B	189.1001664	-0.522189744	-3.931134388	8.45E-05	0.00064821	-1.436
TFAP4	49.03601062	-0.809192839	-3.930289462	8.48E-05	0.00065021	-1.752
RDH10	440.6291276	0.358804804	3.930160564	8.49E-05	0.00065027	1.282
ALS2CL	183.3595292	0.524194269	3.925511433	8.65E-05	0.00066149	1.438
FANK1	273.0880307	0.450398823	3.925348674	8.66E-05	0.00066165	1.366
AR	162.8561158	-0.538541639	-3.924478737	8.69E-05	0.00066375	-1.453
SLC12A7	238.1770243	-0.459473127	-3.923862085	8.71E-05	0.00066516	-1.375
ZNF608	85.34938206	-0.732140819	-3.923212775	8.74E-05	0.00066666	-1.661
KB-1589B1.1	550.0588286	-0.354673334	-3.922442682	8.77E-05	0.00066821	-1.279
NR2F1	580.5029933	0.327281628	3.915377155	9.03E-05	0.00068627	1.255
STX1A	581.8347555	0.35022062	3.914849129	9.05E-05	0.00068747	1.275

SSH2	571.8127514	0.330225794	3.913076675	9.11E-05	0.00069193	1.257
DCAF4	338.2562988	-0.414376086	-3.911706122	9.16E-05	0.00069526	-1.333
LSM11	271.3197797	0.436326925	3.90948035	9.25E-05	0.00070078	1.353
MT-ND4	90261.5366	-0.447157504	-3.906172459	9.38E-05	0.0007095	-1.363
C3orf14	158.1567345	-0.5444844	-3.898158332	9.69E-05	0.00073179	-1.458
RFX8	87.36655078	-0.670915504	-3.896651523	9.75E-05	0.00073572	-1.592
CCDC56	518.1095016	-0.354405863	-3.890486983	0.000100043	0.00075335	-1.278
SERPINB2	207.1420494	0.497915732	3.885378534	0.000102171	0.00076803	1.412
NFKBIA	344.7158978	-0.403997604	-3.884374602	0.000102594	0.00077021	-1.323
PSME1	705.0596507	-0.323986078	-3.881106005	0.000103983	0.00077962	-1.252
COX7A1	344.551736	0.488066032	3.880773054	0.000104125	0.00078035	1.403
IFIH1	50.25807499	-0.799132102	-3.873762772	0.000107168	0.00080142	-1.740
RUNX2	409.7491327	-0.394629965	-3.871414934	0.000108205	0.00080883	-1.315
PCDHGB4	463.783613	0.368046768	3.871219029	0.000108292	0.00080913	1.291
RASA2	513.1012147	0.37017188	3.871025772	0.000108378	0.00080942	1.293
NCAPG	178.2351054	-0.549911226	-3.870621161	0.000108558	0.00081042	-1.464
AGAP2-AS1	335.1855223	-0.402208433	-3.87008821	0.000108796	0.00081184	-1.322
CNNM2	289.275876	-0.443310606	-3.867301454	0.000110046	0.00082082	-1.360
RP11-449M6.1	146.1152208	-0.554806726	-3.864734356	0.00011121	0.00082843	-1.469
PRKAA2	125.062517	0.589404774	3.862855282	0.000112069	0.00083447	1.505
CPED1	570.7511356	0.353124768	3.86250992	0.000112228	0.00083529	1.277
RP11-255M2.3	224.0167582	-0.462495832	-3.861976797	0.000112473	0.0008366	-1.378
HMGB2	540.1781807	-0.331850096	-3.861129844	0.000112864	0.00083894	-1.259
MKI67	503.6134987	-0.402203915	-3.860190619	0.000113299	0.00084181	-1.322
FNIP2	893.9134594	-0.322028402	-3.855438285	0.000115522	0.00085723	-1.250
ABRACL	380.8074929	-0.394886182	-3.853775458	0.00011631	0.00086257	-1.315
MMP1	49.03660045	-0.795549181	-3.853710044	0.000116341	0.00086257	-1.736
CCDC109B	404.3020599	-0.366460927	-3.853557612	0.000116414	0.00086274	-1.289
SERTAD3	482.9296429	0.353356879	3.850027684	0.000118104	0.00087489	1.278
CTD-2033D24.2	91.95387977	-0.648988131	-3.849501583	0.000118358	0.0008764	-1.568
NCKIPSD	649.0321587	0.327010996	3.84848572	0.00011885	0.00087929	1.254
PTGER4	130.9742127	-0.58803455	-3.848358303	0.000118912	0.00087937	-1.503
GALM	359.7095533	-0.397250796	-3.845253453	0.000120428	0.00088944	-1.317
SCD5	464.5573831	0.347139904	3.841414547	0.000122327	0.00090155	1.272
RCAN2	561.6516883	-0.337197046	-3.841461216	0.000122304	0.00090155	-1.263
RPS6KA6	233.979838	0.4638793	3.836956376	0.000124569	0.00091689	1.379
MCC	405.22031	-0.398090566	-3.835614132	0.000125251	0.00092113	-1.318
SYT7	145.9432924	0.552662344	3.831847533	0.000127185	0.00093319	1.467
HMGB3	333.6431116	-0.39374124	-3.831869709	0.000127173	0.00093319	-1.314
GAS2L3	58.93352779	-0.765307291	-3.828619791	0.000128864	0.00094489	-1.700
ABCA6	144.6665383	-0.570587457	-3.824373916	0.000131105	0.00096051	-1.485

KIFC1	156.8665739	-0.538033561	-3.823316932	0.000131668	0.00096301	-1.452
MAP6	211.0677873	0.476245799	3.82091489	0.000132958	0.00097121	1.391
ZNF33A	537.8476084	-0.336730108	-3.81248016	0.000137579	0.00100243	-1.263
NXPH3	234.3926337	0.480377978	3.811236584	0.000138273	0.00100706	1.395
GALNT6	246.951277	0.438285795	3.806509402	0.000140942	0.00102477	1.355
TMEM220	117.3169438	-0.603814674	-3.802200588	0.000143417	0.00104233	-1.520
CLN3	130.2729733	-0.580921675	-3.801006638	0.000144109	0.00104649	-1.496
SPINT2	103.6448696	0.639351695	3.798093927	0.000145813	0.00105599	1.558
TROAP	57.92167119	-0.741642233	-3.798039358	0.000145845	0.00105599	-1.672
CTC-281F24.5	57.83353206	0.734507898	3.796291704	0.000146877	0.00106213	1.664
ALPK1	262.3765282	-0.441933644	-3.795092612	0.000147588	0.00106683	-1.358
ARHGAP11A	294.4935896	-0.424746443	-3.794076043	0.000148194	0.00107031	-1.342
GALNT16	261.5342784	-0.43838603	-3.792723658	0.000149004	0.00107482	-1.355
KANSL1L	145.2934254	-0.551779016	-3.782475242	0.000155277	0.00111588	-1.466
CTD-3035D6.1	183.6871962	-0.509442441	-3.78071741	0.000156377	0.00112239	-1.423
RBM19	532.0808796	-0.346721774	-3.773909329	0.000160709	0.00115062	-1.272
DLX3	152.7552685	-0.529396742	-3.773946896	0.000160685	0.00115062	-1.443
ABHD15	459.226716	0.355141864	3.773264535	0.000161125	0.00115265	1.279
NAMPTL	356.8993194	-0.397605598	-3.770402051	0.000162985	0.00116402	-1.317
PLSCR1	221.886638	-0.472374383	-3.770504899	0.000162918	0.00116402	-1.387
ADH5P4	544.9580399	-0.357056794	-3.770000789	0.000163247	0.00116542	-1.281
TIMELESS	330.1687364	-0.393099536	-3.764448817	0.000166917	0.00119063	-1.313
GRIK2	443.6619292	0.360994771	3.763581265	0.000167497	0.00119379	1.284
MATN2	431.6017604	-0.364554492	-3.758179424	0.000171154	0.00121735	-1.287
TFAP2A	302.4751424	0.403250327	3.75227779	0.000175235	0.00124433	1.322
MITD1	225.8416915	-0.453569734	-3.749914622	0.000176895	0.00125354	-1.369
NRIP3	196.7687513	0.486048555	3.746071029	0.000179626	0.00127133	1.401
CTD-3065B20.1	59.68251404	-0.73225472	-3.743961265	0.000181142	0.00128049	-1.661
UBE2C	132.118261	-0.587672073	-3.736763437	0.000186404	0.00131608	-1.503
TMEM251	111.1071552	-0.631852593	-3.732280421	0.000189754	0.00133755	-1.550
SOD3	74.6823528	-0.694788245	-3.729634579	0.000191758	0.00134948	-1.619
TRIM2	772.1169859	-0.325213445	-3.728314671	0.000192765	0.00135546	-1.253
HOMER1	292.0757912	0.423764473	3.726561245	0.00019411	0.00136236	1.341
ZNF302	550.8250977	-0.325654384	-3.726522326	0.00019414	0.00136236	-1.253
PKP1	66.94558493	0.704201636	3.725508565	0.000194922	0.00136619	1.629
SLC16A13	76.29412001	-0.66077067	-3.724332695	0.000195833	0.00137146	-1.581
DPH5	377.4302736	-0.372490545	-3.723375083	0.000196577	0.00137556	-1.295
TMEM194A	336.3714382	-0.38187506	-3.717684816	0.000201057	0.00140521	-1.303
RP11-366L20.2	38.75848225	-0.796624559	-3.71745412	0.000201241	0.00140592	-1.737
KCNMB1	110.893743	0.60440112	3.716952016	0.000201641	0.00140758	1.520
HSPBAP1	77.43932804	-0.658566476	-3.712629305	0.000205117	0.00142954	-1.579

SPR	469.5747267	-0.342616594	-3.70961447	0.000207575	0.00144551	-1.268
RP11-511H9.4	59.65678576	-0.711797803	-3.702077121	0.000213842	0.00148616	-1.638
FAM167A	154.2443835	0.525706272	3.696463299	0.000218624	0.00151524	1.440
TM7SF2	57.1789423	-0.722104015	-3.696229634	0.000218825	0.00151593	-1.650
PI4K2B	450.9236572	0.341519495	3.694677354	0.000220166	0.00152461	1.267
FDPSL5	55.11670881	-0.737152614	-3.694559916	0.000220268	0.0015247	-1.667
CHN1	97.10748923	-0.615898754	-3.691628323	0.000222823	0.00154054	-1.533
SOCS3	485.0847299	-0.353186367	-3.690078239	0.000224185	0.00154934	-1.277
TMEM25	134.2700521	0.543407042	3.689623357	0.000224586	0.00155149	1.457
CD80	27.81276092	-0.831215752	-3.686959849	0.000226949	0.00156656	-1.779
RAD51D	261.662608	-0.423911248	-3.684747934	0.000228929	0.00157772	-1.342
KIF11	263.1425647	-0.423084419	-3.680465878	0.000232808	0.00160063	-1.341
RASL11A	88.07942058	-0.620280043	-3.678323403	0.000234772	0.00161349	-1.537
AIM1	163.4481817	-0.507033003	-3.677183909	0.000235823	0.00162007	-1.421
TWIST1	311.0317386	-0.395508871	-3.676550342	0.000236409	0.00162345	-1.315
CLN6	358.9872472	-0.412096193	-3.675420167	0.000237458	0.00163001	-1.331
CCDC97	428.5155675	-0.35676494	-3.675306632	0.000237564	0.00163009	-1.281
IRF2	335.2215744	-0.374223565	-3.671116931	0.000241493	0.00165573	-1.296
RP11-295K3.1	121.9571856	-0.554924102	-3.670735504	0.000241854	0.00165755	-1.469
FAM196B	106.6246616	0.593685228	3.666228894	0.000246154	0.00168302	1.509
ITGA4	328.2831691	-0.38664072	-3.664279909	0.000248035	0.00169522	-1.307
HIST1H1C	601.1472281	0.340962444	3.663774429	0.000248526	0.00169723	1.267
PCCA	272.2175138	-0.405493988	-3.662701653	0.000249569	0.00170368	-1.325
CENPP	94.6708533	-0.61100229	-3.661077791	0.000251156	0.00171317	-1.527
VTN	59.89876164	0.712805568	3.65906301	0.000253139	0.00172485	1.639
TYMS	275.9285955	-0.408509628	-3.65746333	0.000254724	0.00173408	-1.327
AP1AR	394.8969238	-0.36418317	-3.65437628	0.000257808	0.00175439	-1.287
CDKL5	447.0512516	0.351552117	3.65266203	0.000259536	0.00176545	1.276
FAXDC2	248.7560886	-0.429254171	-3.650612173	0.000261616	0.00177821	-1.347
LSM2	254.1400687	-0.438062553	-3.649617274	0.000262631	0.00178371	-1.355
ABCC5	683.3389442	-0.327874123	-3.646236668	0.000266109	0.00180379	-1.255
SLC12A8	193.7612178	-0.46437015	-3.646067797	0.000266284	0.00180427	-1.380
MICB	296.0678674	-0.391990116	-3.643477763	0.000268979	0.0018211	-1.312
SELENBP1	402.7774299	-0.385625951	-3.64310361	0.00026937	0.00182257	-1.306
NQO2	647.8454242	-0.325679265	-3.640535494	0.000272072	0.00183845	-1.253
OLFML1	55.07692647	-0.717822591	-3.639756977	0.000272895	0.0018433	-1.645
CENPE	230.3720643	-0.470575668	-3.639225969	0.000273459	0.00184638	-1.386
RFTN2	161.4220025	-0.494268982	-3.63599203	0.000276913	0.00186389	-1.409
KIAA1524	181.8648656	-0.475754341	-3.631838669	0.000281409	0.00189121	-1.391
CABP1	26.00804596	0.825608088	3.624204154	0.000289853	0.00194117	1.772
ABCA7	154.8858364	-0.505777313	-3.622920841	0.000291295	0.00194932	-1.420

TLL2	120.5764503	0.549915748	3.606613862	0.000310219	0.00206956	1.464
CTC-228N24.3	388.969038	-0.353528987	-3.606223134	0.000310686	0.00207188	-1.278
PIGC	240.6781545	-0.418730839	-3.605353344	0.000311728	0.00207803	-1.337
RP11-97E23.3	157.9398741	-0.506041861	-3.604581158	0.000312657	0.00208261	-1.420
FAM83D	91.43322079	-0.609401302	-3.603975753	0.000313386	0.00208587	-1.526
FHOD3	545.7910604	-0.333479347	-3.603398157	0.000314084	0.0020889	-1.260
RP5-1039K5.12	304.1269294	0.417504022	3.599231851	0.000319159	0.00211859	1.336
RP11-214O11.1	310.0894729	-0.388701448	-3.597656157	0.000321098	0.00212983	-1.309
HSPG2	41242.08766	0.441527159	3.597120241	0.00032176	0.0021334	1.358
RP11-178C3.3	27.87502047	0.823339775	3.595876372	0.000323301	0.00214116	1.769
WWOX	103.0891868	-0.590027671	-3.594431973	0.0003251	0.00215225	-1.505
PRR5L	426.5615702	0.349862802	3.593901215	0.000325763	0.00215499	1.274
TLE2	79.38719529	-0.634645102	-3.593952607	0.000325699	0.00215499	-1.553
PPM1K	316.423168	-0.376850534	-3.592685064	0.000327288	0.0021626	-1.299
PIK3R3	324.2196336	-0.37689684	-3.589926298	0.000330771	0.00218312	-1.299
DEPDC1	89.31040808	-0.621945395	-3.588950733	0.000332012	0.00219047	-1.539
EIF4EBP3	28.17580848	-0.805709263	-3.587769462	0.000333519	0.00219957	-1.748
YRDC	416.7788213	0.370155675	3.584850512	0.000337271	0.00222178	1.292
DSCC1	151.9815043	-0.501115216	-3.583230872	0.00033937	0.00223306	-1.415
PSG1	171.3487941	0.543583496	3.580844381	0.000342486	0.00225014	1.458
ZCWPW1	55.13980607	-0.701298304	-3.580412285	0.000343052	0.00225301	-1.626
PAG1	154.0211822	-0.506191644	-3.580213962	0.000343313	0.00225386	-1.420
RP11-463C14.1	118.4787786	0.546312277	3.579564594	0.000344167	0.00225861	1.460
ZXDC	364.2602058	0.364617825	3.573587079	0.000352124	0.00230852	1.288
PPARG	162.1809795	-0.543579403	-3.572471166	0.000353628	0.00231631	-1.458
RITA1	387.7360176	-0.348231139	-3.566359066	0.000361975	0.00236651	-1.273
CDCA8	141.0379293	-0.534923679	-3.565264914	0.000363489	0.00237282	-1.449
LRIG3	375.6047603	-0.354661077	-3.563847913	0.000365458	0.00238388	-1.279
CENPA	38.90776904	-0.766662801	-3.56243319	0.000367433	0.00239586	-1.701
DDX60	125.3226075	-0.541782902	-3.56139833	0.000368885	0.00240442	-1.456
IGF2BP3	133.9401559	0.523583461	3.560647561	0.000369941	0.0024104	1.438
C19orf66	471.358816	-0.334681178	-3.559817037	0.000371113	0.0024144	-1.261
RP11-577H5.5	75.92443317	0.723905519	3.553331956	0.000380384	0.00247007	1.652
RRAGB	385.0643448	-0.346898512	-3.550465102	0.000384551	0.00249339	-1.272
FAM167B	39.70453494	-0.753953337	-3.547969784	0.000388213	0.00251243	-1.686
IL16	91.09474522	-0.624396005	-3.547260741	0.000389259	0.00251668	-1.542
RP11-501M7.2	211.1611726	-0.468858109	-3.546868091	0.00038984	0.00251825	-1.384
AC010877.1	463.0097288	-0.332253052	-3.545000832	0.000392612	0.00253426	-1.259
PRRT3	262.6520354	0.407393438	3.539707024	0.000400571	0.00258083	1.326
RP11-274H2.5	25.8928485	0.802872803	3.538829621	0.000401905	0.00258846	1.745
WDR60	501.3166557	0.32515848	3.537620126	0.00040375	0.00259841	1.253

FUT11	579.7963743	0.333509759	3.531700807	0.000412896	0.00265136	1.260
C2CD2L	411.0779056	0.343780687	3.529139444	0.000416913	0.00267319	1.269
METTL5	400.7699363	-0.353810085	-3.528409079	0.000418066	0.00267958	-1.278
MFSD14A	455.0134142	-0.325489597	-3.525210903	0.000423146	0.00271014	-1.253
NTF3	97.02257637	0.577660413	3.524643715	0.000424053	0.00271293	1.492
CTB-67I13.1	479.7699498	0.328112818	3.523614826	0.000425703	0.00272248	1.255
ZNF354A	223.6577121	-0.444122787	-3.514882802	0.000439948	0.00280426	-1.360
CTD-2081C10.2	100.5656615	0.59672908	3.514088336	0.000441266	0.00281112	1.512
DSTNP2	487.8191122	-0.377574437	-3.512932727	0.00044319	0.0028187	-1.299
SNRPE	320.7338533	-0.372278803	-3.509285773	0.000449312	0.0028524	-1.294
ANO1	112.6173433	0.554517373	3.506247002	0.000454473	0.00288199	1.469
GMPR	138.1286862	-0.511265143	-3.503706839	0.00045883	0.00290856	-1.425
SIX1	324.7256	-0.380514268	-3.500725806	0.000463993	0.00293806	-1.302
VMO1	186.5232396	-0.468752864	-3.498094875	0.000468594	0.00296329	-1.384
RP4-539M6.12	163.3564989	0.486353462	3.494440122	0.000475057	0.00299825	1.401
GRK5	308.4427851	0.388219503	3.492085402	0.000479265	0.0030226	1.309
CTD-2003C8.1	77.62291702	-0.630947456	-3.490924546	0.000481352	0.00303245	-1.549
KRT80	63.91074566	0.654736663	3.489114539	0.000484623	0.00305084	1.574
MPLKIP	443.4751383	-0.337057826	-3.488743737	0.000485296	0.00305396	-1.263
CHST7	246.3031259	0.402642057	3.488246951	0.000486199	0.00305853	1.322
AHNAK	45345.52004	0.390190482	3.482693015	0.000496397	0.00311815	1.311
LYRM4	426.7903966	-0.332669051	-3.480271668	0.000500906	0.00314166	-1.259
HIST3H2A	42.39397286	-0.732564811	-3.479531561	0.000502291	0.00314718	-1.662
RP11-58E21.3	63.60281069	0.653361457	3.477379465	0.000506341	0.00316797	1.573
RP11-267D19.2	74.58107345	0.629220923	3.477399582	0.000506303	0.00316797	1.547
ZNF480	318.4626952	-0.366795364	-3.47122443	0.000518091	0.0032333	-1.289
AC027763.2	77.69851335	-0.63185947	-3.470393482	0.000519696	0.00324099	-1.550
A2M	199.9825183	-0.444285394	-3.46723715	0.000525838	0.00327339	-1.361
BLVRA	479.2431334	-0.322061286	-3.465572639	0.000529104	0.00329048	-1.250
UBE2T	118.3669578	-0.541944104	-3.463914678	0.000532375	0.00330814	-1.456
RP11-42I10.1	17.67913621	-0.802601599	-3.460677501	0.000538818	0.00334337	-1.744
RP5-857K21.8	281.6020901	-0.43540706	-3.459477573	0.000541224	0.0033571	-1.352
SWT1	80.90476959	-0.599907637	-3.452886542	0.000554622	0.00343283	-1.516
SMIM10	152.2028148	-0.479309205	-3.452199059	0.000556037	0.00344036	-1.394
RP11-114F3.2	217.1458338	-0.425891159	-3.450042927	0.000560497	0.00346548	-1.343
EPHA5	358.6670978	0.358655909	3.449240594	0.000562166	0.00347455	1.282
ESCO2	37.35310391	-0.744401746	-3.445240065	0.000570552	0.00351885	-1.675
WDR63	81.12494441	-0.625151988	-3.436954039	0.000588295	0.00361796	-1.542
RCBTB2	363.7614969	-0.340638327	-3.435706553	0.000591011	0.00363208	-1.266
JUP	225.1958014	0.438958638	3.435068326	0.000592404	0.00363807	1.356
SERTAD4-AS1	44.82606712	-0.710702222	-3.43516393	0.000592195	0.00363807	-1.637

NUDT18	335.2162586	0.354074081	3.434521757	0.0005936	0.00364412	1.278
RP11-550E22.4	248.5340754	-0.416425917	-3.434112686	0.000594497	0.00364747	-1.335
CYFIP2	134.9801572	-0.512240539	-3.43400449	0.000594734	0.00364747	-1.426
CARD16	13.76497475	-0.784764717	-3.432305959	0.000598472	0.00366752	-1.723
CTA-392C11.1	9.495678159	0.759464886	3.431439689	0.000600387	0.00367795	1.693
DES	29.02523047	0.770693593	3.43120736	0.000600901	0.0036798	1.706
DTNBP1	203.6362785	-0.456049777	-3.430794749	0.000601816	0.0036841	-1.372
RP11-400N13.3	8.483182446	-0.744398819	-3.42428916	0.00061641	0.00376412	-1.675
WDR62	73.39961786	-0.616097521	-3.42119852	0.000623458	0.00379912	-1.533
CLK4	241.181471	-0.40538028	-3.41994427	0.00062634	0.00381266	-1.324
ITIH5	51.53393549	0.683768157	3.419002326	0.000628512	0.00382453	1.606
ZNF836	69.45183841	-0.667145907	-3.41806095	0.00063069	0.00383593	-1.588
LDB3	119.7301248	0.522949123	3.41506997	0.000637656	0.00387201	1.437
RP11-706J10.1	49.37045664	0.708953138	3.409154084	0.000651647	0.00394865	1.635
FAM110A	76.17374363	-0.605211135	-3.404922779	0.000661828	0.00400614	-1.521
FAM111A	421.453608	-0.34240178	-3.399525223	0.00067503	0.00407608	-1.268
EDARADD	381.5704947	0.340898777	3.395820874	0.000684231	0.00412588	1.267
CLDN11	539.2930792	0.336882237	3.395706739	0.000684517	0.00412617	1.263
CCDC81	64.1792873	0.648669214	3.395180983	0.000685832	0.00412979	1.568
SAMD11	416.1100384	-0.355476554	-3.394836388	0.000686696	0.00413355	-1.279
DIO2	61.42391445	0.663447139	3.39332524	0.000690496	0.00415353	1.584
DNAJA4	129.0718527	0.518449265	3.393199425	0.000690813	0.004154	1.432
AC073869.22	157.0382922	-0.493382703	-3.392426832	0.000692764	0.00416428	-1.408
SLC45A1	85.69343905	0.583691929	3.391895696	0.000694109	0.00417092	1.499
ANP32BP1	137.3192208	-0.493955575	-3.388530601	0.000702682	0.00421512	-1.408
SOCS7	526.476461	0.323359595	3.388097679	0.000703792	0.00422031	1.251
TSPAN15	108.715475	-0.553508347	-3.387323973	0.00070578	0.00423077	-1.468
MECOM	288.2652334	-0.375011174	-3.384725175	0.000712496	0.00426659	-1.297
FAM20A	267.6388606	-0.400198825	-3.383399324	0.000715945	0.00428377	-1.320
VPS50	489.5022727	-0.330570653	-3.380593032	0.000723296	0.0043223	-1.258
INHBE	90.91270758	0.57044672	3.379546149	0.000726056	0.0043358	1.485
PARP10	426.50386	-0.332293835	-3.37654578	0.000734022	0.00437281	-1.259
STX1B	68.25536977	0.634494739	3.376437705	0.00073431	0.00437302	1.552
CYP27A1	78.01446709	-0.60384133	-3.374507101	0.00073948	0.00440078	-1.520
AC125232.1	85.71571504	0.655459999	3.373315112	0.000742689	0.00441684	1.575
RHCE	43.19279004	0.696595673	3.362398526	0.000772685	0.0045781	1.621
RP11-569G13.1	443.9342002	-0.328127089	-3.358782116	0.000782868	0.00462952	-1.255
CASC5	123.026715	-0.53097217	-3.358736606	0.000782997	0.00462952	-1.445
PALB2	276.6764048	-0.388065753	-3.350770733	0.00080587	0.00474693	-1.309
CEBPA	43.05227726	-0.706427941	-3.349150614	0.000810597	0.00477151	-1.632
FBXL19-AS1	175.9174773	0.4541446	3.345284428	0.000821982	0.00482215	1.370

RMND1	422.512462	-0.328659567	-3.345481547	0.000821398	0.00482215	-1.256
FTLL2	33.06678421	-0.733819919	-3.3443949	0.000824623	0.00483436	-1.663
RP11-378J18.6	118.4287909	-0.51364585	-3.341947396	0.000831928	0.00487349	-1.428
HOXA7	217.2990019	-0.413803054	-3.3415207	0.000833208	0.00487809	-1.332
CTNNBIP1	366.6375084	0.334800269	3.335301619	0.000852069	0.00497841	1.261
SH3RF3	292.1241426	-0.374865625	-3.331950566	0.000862396	0.00503025	-1.297
PLEKHH2	275.2377039	-0.380426185	-3.324565413	0.000885565	0.00515497	-1.302
SHMT1	134.7150785	-0.491320339	-3.320149466	0.000899693	0.00523018	-1.406
COA5	236.6139091	-0.387299166	-3.311159659	0.000929102	0.0053939	-1.308
RP11-289I10.2	328.3262881	0.346008488	3.306558681	0.000944496	0.00547592	1.271
PLPP2	64.57252094	-0.640755315	-3.304768048	0.00095055	0.00550918	-1.559
NLGN4X	77.31999321	-0.592929535	-3.303880615	0.000953564	0.0055248	-1.508
RP11-21L23.2	54.28587129	0.650805637	3.29874616	0.000971177	0.00561557	1.570
RP11-480P3.1	303.3917791	-0.350375532	-3.294907498	0.000984541	0.00568525	-1.275
SNORD3A	31.15897909	0.731140691	3.291766538	0.000995602	0.00573955	1.660
RP5-1053E7.3	46.22273586	-0.682858893	-3.284529974	0.001021526	0.00587335	-1.605
GMIP	304.6344001	0.354262678	3.277150151	0.001048606	0.00600909	1.278
ASPM	241.8910362	-0.393649225	-3.270403991	0.00107394	0.00613396	-1.314
AC078791.3	130.7019402	0.492805769	3.268821997	0.001079962	0.00616226	1.407
ZDBF2	424.7681401	0.339309495	3.264460377	0.001096728	0.00624763	1.265
RP11-1024P17.1	53.56230431	0.661900964	3.263309163	0.001101193	0.00626894	1.582
AC004696.2	217.8479376	-0.408898924	-3.260370277	0.001112669	0.00632803	-1.328
DTNA	131.5717368	-0.484845347	-3.258152836	0.0011214	0.00636723	-1.399
CDK15	244.0239744	0.393116453	3.256394354	0.001128369	0.0064047	1.313
RP11-69I8.3	68.67186263	0.603199068	3.253997762	0.001137932	0.00645474	1.519
RP4-753D5.4	12.82773169	-0.71973328	-3.25206539	0.001145697	0.00649453	-1.647
CHI3L2	20.1510463	-0.749784574	-3.251687983	0.001147219	0.00650103	-1.682
AC068057.2	44.13171901	0.673464344	3.251226485	0.001149083	0.00650946	1.595
RP11-119P22.1	85.42547785	-0.567540285	-3.247873452	0.00116271	0.00657805	-1.482
CDA	61.42450278	0.635055663	3.247776204	0.001163107	0.00657815	1.553
PCDHB5	69.03590085	-0.595920841	-3.24711931	0.001165795	0.0065912	-1.511
STRBP	165.7031858	-0.450703473	-3.242678697	0.001184117	0.00668605	-1.367
RASSF2	290.8328854	0.371672699	3.242141504	0.001186351	0.00668994	1.294
MAP2K1P1	159.4308348	0.478994704	3.239468859	0.001197525	0.00674855	1.394
GPER1	140.7061155	0.468805076	3.234633647	0.001217989	0.00684827	1.384
SPRN	54.70685027	0.637235803	3.23290841	0.001225368	0.00688529	1.555
ZNF182	117.7682433	-0.520470327	-3.232643188	0.001226507	0.00688945	-1.434
CTD-2066L21.3	28.83320648	0.720510353	3.229423772	0.001240399	0.00696523	1.648
RASGRP1	35.0781787	0.701817842	3.228896245	0.00124269	0.00697583	1.627
C10orf11	31.35667255	-0.710423739	-3.228350714	0.001245062	0.00698235	-1.636
FOXO1	122.5128232	-0.490299436	-3.227516678	0.001248698	0.0069982	-1.405

SHROOM2	90.11800496	0.549144087	3.22435168	0.001262582	0.00707144	1.463
EGF	127.6570706	0.490189489	3.22347444	0.001266456	0.00708626	1.405
PANK1	124.4411535	-0.490838901	-3.22184811	0.001273666	0.0071197	-1.405
ARL17B	54.5434118	0.637534085	3.217648648	0.00129246	0.00720846	1.556
CKAP2L	107.9162016	-0.52959063	-3.215450914	0.001302398	0.00725687	-1.444
RP5-907D15.2	144.2928722	-0.452026301	-3.205429853	0.001348609	0.00748305	-1.368
TMSB4XP4	111.9307879	-0.575309631	-3.205216685	0.001349608	0.0074862	-1.490
LBX2	28.54966461	-0.714853243	-3.20158572	0.001366734	0.00756665	-1.641
C8orf58	371.9237415	0.322211701	3.19913114	0.001378425	0.00761917	1.250
AC069303.1	126.7642729	-0.493448012	-3.198947827	0.001379301	0.00761917	-1.408
ZNF33B	182.0324111	-0.421487969	-3.19473145	0.00139961	0.00772888	-1.339
GPRC5C	163.5328561	-0.46884516	-3.191197854	0.001416842	0.00782155	-1.384
RARRES3	47.93990233	-0.664725791	-3.187766496	0.001433763	0.00790487	-1.585
S1PR1	62.34505986	-0.605956106	-3.18733374	0.00143591	0.00791167	-1.522
LAMP5	58.36651145	-0.611302351	-3.183367035	0.00145573	0.00800813	-1.528
CASQ1	24.94512517	0.726884111	3.181435087	0.001465474	0.0080515	1.655
HDHD3	142.6531275	-0.462278441	-3.179150793	0.001477072	0.00811008	-1.378
AKNA	279.052463	-0.353284653	-3.177997022	0.001482963	0.00813468	-1.277
CCDC102B	40.0408027	-0.689200984	-3.174908594	0.001498837	0.00821135	-1.612
FBLN7	311.5342273	0.33404401	3.174248052	0.001502252	0.00822362	1.261
RP11-796G6.1	405.0920628	-0.338137581	-3.174199872	0.001502502	0.00822362	-1.264
NDC80	83.08078676	-0.56083285	-3.172451534	0.001511578	0.00826807	-1.475
RP11-170M17.2	319.9211445	-0.339042881	-3.170708126	0.001520679	0.00831522	-1.265
LZTS3	180.167976	0.422003059	3.170510786	0.001521712	0.00831824	1.340
CDKL1	59.01357797	-0.605671365	-3.17020314	0.001523324	0.00832443	-1.522
CCDC136	175.2836672	0.440176974	3.167841797	0.001535751	0.00838969	1.357
RP3-340B19.2	166.0429644	-0.446030194	-3.165107788	0.001550255	0.00846091	-1.362
RP11-518K17.3	12.77568039	-0.71102343	-3.163375464	0.001559511	0.00850606	-1.637
RP11-403F21.4	22.77024992	0.723403779	3.162521719	0.001564091	0.00852354	1.651
NEK2	48.60195404	-0.64865726	-3.162502758	0.001564193	0.00852354	-1.568
SNAPC5	208.6481886	-0.410081197	-3.153553358	0.001612957	0.0087562	-1.329
LSM6	140.4874786	-0.455309936	-3.152864624	0.001616768	0.00877138	-1.371
SLC15A3	64.23863761	-0.600480129	-3.152453107	0.001619048	0.00877825	-1.516
WDR66	96.38257876	0.526354839	3.149559556	0.001635168	0.00884626	1.440
STAT5A	232.2608102	-0.391885473	-3.149402981	0.001636044	0.00884823	-1.312
RPUSD4	351.743271	-0.329702614	-3.144637991	0.001662925	0.00898519	-1.257
DUSP23	192.8211503	-0.424031371	-3.143678631	0.001668386	0.00901188	-1.342
ZNF181	230.3853242	-0.387342778	-3.142903431	0.00167281	0.00903297	-1.308
HILPDA	193.5618478	0.415683869	3.142586999	0.001674619	0.00903389	1.334
SHCBP1	239.9187929	-0.385294204	-3.140126281	0.00168875	0.00909634	-1.306
ZNF195	207.7844362	-0.425933981	-3.137472579	0.001704112	0.00917338	-1.343

PLCB1	347.3525617	0.323161361	3.136571623	0.001709357	0.00919875	1.251
GPR85	55.02512517	-0.640137988	-3.13412849	0.001723654	0.00926705	-1.558
MT-ND1	16825.29546	-0.362986842	-3.128036188	0.001759785	0.00945066	-1.286
SYT12	263.3100911	0.367687163	3.127016029	0.001765903	0.00947948	1.290
RMRP	42.55037588	0.659389994	3.125926682	0.001772457	0.00950876	1.579
PDK3	73.47781964	0.568624565	3.123056512	0.001789834	0.00958416	1.483
RP11-76P2.3	80.67509412	-0.550998693	-3.119544037	0.001811312	0.00968719	-1.465
IL6R	95.08759805	-0.530026598	-3.118385187	0.00181845	0.00971636	-1.444
SGIP1	171.9644028	-0.414310618	-3.113138872	0.001851089	0.00986032	-1.333
FAM217B	186.1262255	-0.431197263	-3.112534492	0.001854883	0.0098775	-1.348
MMRN2	56.92978108	0.605565959	3.112015342	0.001858148	0.00989184	1.522
SRGAP2D	41.34106608	0.68080423	3.105246462	0.001901205	0.01009409	1.603
C20orf100	115.4946954	-0.50078491	-3.101159633	0.001927643	0.01021781	-1.415
PRKAR1B	270.4887611	0.358231259	3.100965154	0.00192891	0.01022139	1.282
HCFC1R1	298.7560567	-0.386666756	-3.100730553	0.001930439	0.01022636	-1.307
RP5-857K21.9	2252.865624	-0.453401379	-3.099535724	0.001938242	0.01025515	-1.369
TNFSF9	156.5530593	-0.442472186	-3.098560878	0.00194463	0.0102858	-1.359
KIF20B	176.440607	-0.411449264	-3.092320205	0.001985985	0.01047255	-1.330
GJC2	46.45114224	0.63085288	3.090307863	0.001999491	0.01053094	1.548
CCDC74A	157.7520676	-0.464810705	-3.086908491	0.002022498	0.0106327	-1.380
TATDN1	251.8847164	-0.374122202	-3.080877757	0.002063914	0.01083313	-1.296
GSTM4	159.2198075	-0.42266103	-3.078970583	0.002077172	0.01089697	-1.340
KIF4A	117.7587524	-0.477309965	-3.061399022	0.002203053	0.01148427	-1.392
SHANK2	93.23964741	0.536689417	3.061253525	0.002204124	0.01148639	1.451
CHST2	261.9673844	-0.365319468	-3.056525816	0.002239182	0.01165155	-1.288
MIR210	16.7383948	0.704552974	3.04819724	0.002302188	0.01195067	1.630
RILP	75.42988821	-0.563878583	-3.047573468	0.002306971	0.01197191	-1.478
PRKAR2B	49.08780991	-0.619268201	-3.039469706	0.00236995	0.01225832	-1.536
POLA1	215.2216802	-0.37067235	-3.038315214	0.00237905	0.01229436	-1.293
CH17-118O6.2	28.6739197	-0.682030231	-3.037079766	0.002388822	0.01234051	-1.604
BDH2P1	219.7492176	-0.376764163	-3.035436296	0.00240188	0.01239693	-1.298
STBD1	69.3498459	0.556479837	3.030917741	0.002438117	0.01256585	1.471
CTSH	75.63655382	-0.552291177	-3.027390414	0.002466751	0.01269831	-1.466
FIGN	120.6876866	-0.464998057	-3.023317633	0.002500197	0.01283993	-1.380
NPIPA3	33.80261769	0.674307862	3.017562058	0.002548169	0.01307295	1.596
GPR34	19.28655296	0.694834821	3.016997737	0.002552917	0.01309126	1.619
STK26	233.8406974	-0.360144952	-3.016781251	0.002554741	0.01309674	-1.284
AMZ1	51.75909735	0.605603442	3.01437334	0.002575107	0.01318593	1.522
DHRS4-AS1	196.2052187	-0.382857892	-3.012908487	0.002587569	0.0132415	-1.304
CDKN3	74.78925416	-0.543608874	-3.012442878	0.002591542	0.01325791	-1.458
C15orf41	203.8573696	-0.395355751	-3.0065058	0.00264269	0.01349565	-1.315

PRKG1	247.9386601	-0.355139854	-3.005640787	0.002650219	0.01353011	-1.279
DISC1	97.23556026	-0.511883079	-3.005437103	0.002651994	0.01353518	-1.426
RP11-656G9.3	32.14679607	0.658753847	3.0012533	0.002688708	0.01369439	1.579
PER2	162.9849501	0.426226416	2.998455643	0.002713517	0.01381659	1.344
MTERFD3	87.22412462	-0.514045751	-2.991972825	0.002771809	0.01406376	-1.428
RP11-96H19.1	43.08119779	0.620470967	2.990709732	0.002783299	0.01410864	1.537
FAM175A	299.3451941	-0.337724182	-2.990100757	0.002788854	0.01412954	-1.264
3-Mar	83.59649566	0.520829859	2.983322436	0.002851374	0.01442097	1.435
ABCA5	272.7361412	-0.341569314	-2.981675559	0.002866756	0.01448607	-1.267
RP11-473N11.2	430.6201917	-0.323196536	-2.979049964	0.002891436	0.01459197	-1.251
HSPB2	259.8644555	-0.345250721	-2.978656232	0.002895154	0.01460399	-1.270
RNF122	88.99036773	0.507629634	2.978186505	0.002899595	0.01462213	1.422
AC097523.3	24.2559063	-0.679077476	-2.977873998	0.002902553	0.01463279	-1.601
ARHGDIB	82.87932404	0.528866788	2.977274844	0.002908232	0.01465715	1.443
AC006014.6	45.6534745	0.641988629	2.975556972	0.00292457	0.0147352	1.560
KLHL35	72.72713937	0.533604766	2.970002251	0.002977976	0.01497813	1.448
SCLY	206.9330886	-0.376834806	-2.967742203	0.002999958	0.01507993	-1.298
PEG10	178.0341687	0.390505317	2.966903614	0.003008152	0.01510796	1.311
EMC9	191.084193	0.406742619	2.965167705	0.00302518	0.01517147	1.326
LINC00294	279.6166967	0.342197416	2.96403252	0.003036362	0.01522314	1.268
RP11-152C15.1	150.3373032	-0.425447769	-2.962761304	0.003048929	0.0152773	-1.343
PTPN2	285.8737179	-0.327974976	-2.958635776	0.003090041	0.01546049	-1.255
FAM76A	177.4498793	-0.39015767	-2.958432645	0.003092078	0.01546218	-1.311
ASB13	148.0977764	-0.431907371	-2.956861108	0.003107881	0.01552326	-1.349
ADAM33	152.6868101	-0.422144314	-2.955849853	0.003118088	0.01556078	-1.340
KRT17	115.7161349	0.465263412	2.952093734	0.003156271	0.01572247	1.381
MOCOS	255.9238036	-0.334904644	-2.950358622	0.003174053	0.01579378	-1.261
PKNOX2	86.69089623	0.504006713	2.949048686	0.003187538	0.01584343	1.418
CDCA3	88.04450758	-0.553808628	-2.948803294	0.003190069	0.01585146	-1.468
HOMEZ	189.8485136	-0.392374785	-2.947856058	0.00319986	0.01589099	-1.313
SLC22A15	172.6857506	-0.400518107	-2.946388536	0.003215083	0.01596083	-1.320
SPC25	26.79097821	-0.66224491	-2.944840784	0.003231209	0.01602137	-1.583
FDX1L	166.0953268	-0.41172363	-2.939978562	0.00328235	0.01623544	-1.330
GALNT12	50.15364505	-0.594384169	-2.939991203	0.003282216	0.01623544	-1.510
PPP1R14C	81.47851591	-0.518134669	-2.938722914	0.003295676	0.01629204	-1.432
ICAM2	94.20565282	-0.524219672	-2.93784464	0.003305026	0.01632428	-1.438
CTD-2552B11.4	68.43569859	0.53522216	2.932518134	0.003362253	0.01658328	1.449
FAM225B	71.03414191	-0.562426642	-2.929861465	0.003391132	0.01670668	-1.477
PAPPA	6091.098683	0.344678026	2.926117564	0.003432212	0.01689464	1.270
RP1-228P16.7	86.39418629	-0.511911004	-2.923518215	0.003461	0.01701218	-1.426
RP11-175K6.2	57.06480868	-0.565476385	-2.920119133	0.003498976	0.01718907	-1.480

RP11-867G23.10	16.4649038	0.672828286	2.918591826	0.003516163	0.0172686	1.594
RP11-141J13.4	148.248511	-0.450410054	-2.917005069	0.0035341	0.01734685	-1.366
RP11-192P3.3	76.96222699	0.522123619	2.909109222	0.003624602	0.01775583	1.436
AP006621.5	44.41665277	-0.59626961	-2.907081588	0.00364818	0.01786123	-1.512
FRMD4B	64.59677963	0.540342505	2.906266139	0.003657702	0.01790278	1.454
P2RX5	64.03188391	0.551103436	2.904033967	0.003683882	0.01800547	1.465
RP1-249H1.4	85.87576359	-0.522509146	-2.896406852	0.003774627	0.01839188	-1.436
U47924.6	64.23884122	-0.54875727	-2.893856874	0.003805416	0.01853147	-1.463
SKA1	42.69237351	-0.602264739	-2.887195302	0.003886929	0.01887003	-1.518
SULT1A1	248.9611467	-0.359654293	-2.885202478	0.00391162	0.01897393	-1.283
DNAJC12	58.5459285	-0.549918076	-2.879080247	0.003988368	0.01930294	-1.464
TOMM6	147.5562878	-0.420026955	-2.878660986	0.003993674	0.01931782	-1.338
HLA-DMA	147.245779	-0.406788326	-2.875208761	0.004037604	0.01948133	-1.326
SLC25A27	40.03934429	-0.606698769	-2.871524943	0.004084965	0.01967889	-1.523
CTC-444N24.11	176.4914452	-0.473099196	-2.869393937	0.004112592	0.01978799	-1.388
RP11-874J12.1	32.22778387	-0.636433226	-2.868803401	0.004120278	0.01981947	-1.554
RP11-346D6.6	33.41444396	0.641902139	2.865929968	0.004157862	0.01995715	1.560
RP13-516M14.8	37.04939188	0.614027232	2.858183853	0.004260734	0.02040438	1.531
TRMT10B	77.03467351	-0.510731609	-2.857711414	0.004267083	0.02042913	-1.425
C16orf71	53.71360599	0.571967184	2.857438325	0.004270756	0.02044106	1.487
AC002480.4	41.50065936	0.595260018	2.855354697	0.004298879	0.02055294	1.511
RP11-737O24.3	76.78718538	-0.518422492	-2.854500578	0.004310455	0.0206026	-1.432
SLC25A42	274.7165775	0.336389939	2.851986368	0.004344696	0.02074335	1.263
CEND1	27.85703364	0.639198546	2.849418339	0.004379925	0.02089426	1.557
IQGAP3	179.2863776	-0.419284222	-2.846866761	0.004415184	0.02104506	-1.337
CTB-36O1.5	391.6709265	-0.551838346	-2.845491667	0.004434292	0.02112451	-1.466
SPATA13	139.1761403	-0.411757996	-2.844464975	0.004448608	0.02117523	-1.330
GEMIN7	255.5719695	0.32801745	2.843676843	0.004459625	0.02122183	1.255
NACAD	158.6075403	0.397274014	2.841797597	0.004485996	0.02133686	1.317
MT-ND2	13762.36737	-0.334832041	-2.840468614	0.004504731	0.02139932	-1.261
NCAPH	65.18350675	-0.532733238	-2.839112422	0.004523922	0.02147467	-1.447
TOP1MT	249.036831	-0.33778799	-2.837557812	0.004546011	0.0215677	-1.264
GPR89B	82.38306516	-0.494702298	-2.836145564	0.004566163	0.02165144	-1.409
DPF3	71.8956367	0.516826707	2.835544869	0.004574759	0.02168032	1.431
SLC2A11	207.634279	-0.380119056	-2.833399451	0.004605579	0.02180848	-1.301
ACOT1	201.8159586	-0.358206921	-2.831209172	0.004637238	0.02194638	-1.282
EPHA4	160.307423	0.395179973	2.827659525	0.004688965	0.02216089	1.315
MYOM1	224.0181296	0.340624739	2.825969735	0.004713772	0.02226598	1.266
PQLC2L	68.53885846	-0.523401089	-2.822050789	0.004771762	0.02247245	-1.437
SAMD12	71.59829897	-0.525690503	-2.821032906	0.004786929	0.0225255	-1.440
TIGD2	221.5484708	-0.344029329	-2.820160908	0.004799957	0.0225684	-1.269

RP11-434D2.6	174.4970751	-0.405637983	-2.815315853	0.004872931	0.02286804	-1.325
NKAPL	44.45442902	0.581057427	2.814860668	0.004879839	0.02289425	1.496
RP11-778J16.1	249.0277692	-0.339386263	-2.810485569	0.004946681	0.0231639	-1.265
IRF1	274.5437317	-0.327951633	-2.810277061	0.004949887	0.02317265	-1.255
TM4SF4	23.72669111	0.638668826	2.808474807	0.004977678	0.02327757	1.557
RP11-603K19.1	168.3189221	-0.400152704	-2.806154851	0.005013659	0.02341421	-1.320
TMEM51	29.17096267	-0.621937049	-2.805879857	0.00501794	0.02342788	-1.539
RP11-90O23.1	62.11667018	-0.53319626	-2.800502029	0.005102319	0.02378334	-1.447
RP11-367G6.2	76.51402	0.500639304	2.800005257	0.005110177	0.02380716	1.415
FTHL23	45.10883839	-0.584454496	-2.798525188	0.005133657	0.02390213	-1.499
FRY	249.7994914	0.326666025	2.798172634	0.005139264	0.02391692	1.254
GLRX2	248.0403225	-0.328807361	-2.794036433	0.005205461	0.02419247	-1.256
SYNE1	3520.471368	0.336933401	2.793796568	0.005209324	0.02420392	1.263
ADAMTS15	130.723379	0.418018804	2.789839148	0.005273423	0.02446235	1.336
RP11-800A3.7	69.05922242	0.508247678	2.787551344	0.005310804	0.02457649	1.422
TMEM171	89.80189051	0.487425815	2.786347781	0.005330565	0.02466134	1.402
ACTR3B	93.33059648	0.473533786	2.786249725	0.005332178	0.02466221	1.389
LRRC1	144.783206	0.396075745	2.785512167	0.005344323	0.02471179	1.316
CASP6	246.6226768	-0.32578337	-2.785381273	0.005346482	0.02471516	-1.253
CMC1	209.5649377	-0.344510326	-2.779291415	0.005447763	0.02514307	-1.270
FAM174B	48.90965269	0.561827498	2.775703269	0.005508246	0.02538838	1.476
HTR1F	97.47774677	0.475044793	2.774428514	0.005529879	0.0254542	1.390
SKA3	47.46175697	-0.583263454	-2.772040313	0.005570614	0.02559407	-1.498
IGFBPL1	19.90809746	0.637819893	2.768601053	0.005629752	0.02583151	1.556
ADCK3	201.6748737	-0.350881031	-2.767945674	0.005641086	0.02586294	-1.275
POMK	77.30178193	0.501231851	2.767091089	0.005655895	0.02592398	1.415
RP4-621F18.2	212.5204852	0.364349104	2.765163746	0.005689423	0.02605695	1.287
COL21A1	108.6081049	0.440917967	2.764630086	0.005698738	0.02609271	1.357
RP11-298J20.4	36.78958075	0.5937429	2.764495031	0.005701098	0.02609662	1.509
TPK1	102.9107852	-0.448787265	-2.761814221	0.005748118	0.02628405	-1.365
AKR1B10	102.2959893	-0.45127175	-2.759575272	0.005787655	0.02645087	-1.367
NCRNA00107	9.651262653	-0.608877486	-2.758361476	0.005809192	0.0265245	-1.525
RP11-40H20.1	127.4880182	-0.414639367	-2.756462487	0.005843031	0.02665469	-1.333
RP11-350G24.2	110.6025289	-0.450446717	-2.756126094	0.005849044	0.02667509	-1.366
PPP1R14A	249.3102227	0.328158104	2.75312825	0.005902877	0.02685693	1.255
SLC18B1	157.2782097	-0.376429532	-2.753209739	0.005901408	0.02685693	-1.298
KLHDC7B	29.61476387	-0.614464121	-2.75088238	0.005943498	0.02702754	-1.531
RP11-820K3.6	14.54126866	0.631889969	2.749476975	0.005969046	0.02712234	1.550
RP11-624L4.1	136.0249266	0.400369034	2.748377278	0.005989105	0.02719921	1.320
PTK2B	223.8867679	0.332069769	2.746485386	0.006023757	0.02733507	1.259
BPHL	184.885184	-0.362585283	-2.74421326	0.006065612	0.02748897	-1.286

TRAF1	205.4268633	0.343506952	2.74066285	0.006131539	0.02777321	1.269
SERP2	50.54429302	0.548906798	2.739791028	0.006147826	0.02783242	1.463
DRP2	109.8012178	0.455650315	2.733730067	0.006262136	0.02827598	1.371
TNFRSF19	206.1695379	-0.349751044	-2.729178836	0.006349226	0.02863934	-1.274
FBXL22	36.79274475	-0.583861525	-2.729059524	0.006351524	0.02864225	-1.499
AC144831.3	61.00601618	0.516863387	2.725587912	0.006418708	0.02888502	1.431
PECR	110.2824073	-0.42812625	-2.724832807	0.006433406	0.02894364	-1.345
LINC00340	33.75729634	-0.592001696	-2.723966469	0.006450305	0.02899706	-1.507
GAREM2	221.8516759	0.32833819	2.723161969	0.006466035	0.02906023	1.256
RPPH1	26.21536051	0.618104475	2.722699802	0.006475087	0.02909336	1.535
RP5-854E16.2	180.5766849	-0.357774789	-2.721939691	0.006489999	0.02913012	-1.281
RP11-159F24.1	209.4866743	-0.33964187	-2.71735831	0.006580532	0.0294906	-1.265
ELOVL4	221.3181362	-0.327368237	-2.71679552	0.006591732	0.02953314	-1.255
RNASEL	217.8996887	-0.340773924	-2.716049448	0.006606605	0.02958447	-1.266
HIST1H4H	153.5407521	-0.374800464	-2.714921776	0.006629143	0.02963174	-1.297
PKMYT1	65.87641222	-0.526387892	-2.71494964	0.006628585	0.02963174	-1.440
AC008268.3	48.98607396	-0.546668924	-2.713630994	0.006655025	0.02973208	-1.461
PLTP	200.3906528	-0.365922053	-2.710410701	0.006719994	0.02997592	-1.289
LIPT1	45.92453982	-0.557520201	-2.706209923	0.006805601	0.03028755	-1.472
PTPN3	229.189115	0.341250886	2.705853142	0.006812917	0.03031231	1.267
RP11-90L1.8	55.2497117	0.54481545	2.705698386	0.006816092	0.03031865	1.459
IL17RD	142.7313241	-0.391337657	-2.702949834	0.006872712	0.0305548	-1.312
GLMN	146.4536455	-0.386736658	-2.696572133	0.007005722	0.03098699	-1.307
FGD4	209.2020907	0.344438962	2.696021733	0.007017309	0.03103031	1.270
RP11-480I12.3	113.6646923	0.421470494	2.695300416	0.007032519	0.03108963	1.339
PTTG1	126.9773338	-0.401553873	-2.695081159	0.007037149	0.03110215	-1.321
CITED4	157.4285347	0.390980677	2.694956159	0.007039789	0.0311055	1.311
RP11-452L6.5	104.1534257	0.439943457	2.69454386	0.007048505	0.0311285	1.357
ARG2	86.85927621	0.466378375	2.693633537	0.007067783	0.03118902	1.382
ADRA1B	33.61478542	0.591233175	2.693209573	0.007076777	0.03120559	1.507
PDE1A	96.64756386	-0.461307879	-2.692918017	0.007082968	0.03122494	-1.377
PCDHB14	167.108959	-0.37089888	-2.689693704	0.007151763	0.03145613	-1.293
CMTM8	46.54037306	-0.564100871	-2.689212614	0.007162079	0.0314855	-1.478
AC068580.5	11.20305803	-0.605480821	-2.686171806	0.007227591	0.03170105	-1.521
BHLHB9	121.1780102	-0.407159339	-2.685212433	0.007248372	0.03178161	-1.326
CDH15	151.4303958	0.371682712	2.683735805	0.007280461	0.03188448	1.294
PITPNC1	160.7783862	-0.378066035	-2.679650539	0.007369906	0.03219131	-1.300
SPATA6	97.41455374	-0.439719814	-2.679698782	0.007368844	0.03219131	-1.356
PRELID2	81.46366345	0.469643521	2.679457414	0.007374158	0.03219708	1.385
RP11-6B6.3	124.2875637	-0.41647481	-2.675772656	0.00745572	0.03246314	-1.335
ISOC1	137.0639531	-0.390476557	-2.673764072	0.00750052	0.03261718	-1.311

DPM3	195.7443318	-0.371003296	-2.664293751	0.007715016	0.03341565	-1.293
LBX2-AS1	47.27333432	-0.539188767	-2.66331796	0.007737426	0.03349596	-1.453
EFNA5	205.2326826	-0.334537187	-2.657983269	0.00786098	0.03392901	-1.261
ZNF815P	33.86660224	-0.579438498	-2.655768093	0.007912801	0.03411866	-1.494
SGO2	181.1275264	-0.352056061	-2.654164436	0.007950507	0.03424712	-1.276
ALG11	352.5041286	0.324397381	2.652132729	0.007998509	0.03444532	1.252
CTD-2037K23.2	36.28359324	-0.573707171	-2.6504751	0.008037865	0.03458899	-1.488
LAGE3	189.2365666	-0.337520184	-2.650019301	0.008048717	0.03462709	-1.264
C20orf96	106.4690785	-0.422785766	-2.649527558	0.00806044	0.03466736	-1.341
CDH4	216.676269	0.327099257	2.646014566	0.008144631	0.03497888	1.254
P2RY6	85.84631548	0.467292192	2.645838772	0.008148865	0.0349797	1.383
RP11-326I19.1	54.1358266	0.529160111	2.644485368	0.008181524	0.03510249	1.443
SAMD10	38.30574097	0.566547473	2.643074926	0.008215685	0.03522287	1.481
RP11-382M14.1	62.28983318	0.506492138	2.642884892	0.008220298	0.0352252	1.421
RP11-138I17.1	20.92938365	0.605097272	2.641771019	0.008247379	0.03532376	1.521
RP4-798C17.5	98.26339677	0.43935287	2.641089785	0.008263982	0.03537736	1.356
RP11-73M18.7	164.8515826	0.367522024	2.638280891	0.008332753	0.03558377	1.290
RBM24	153.1621264	0.365377428	2.635992564	0.008389157	0.03580697	1.288
N4BP2L1	28.57474248	-0.589343191	-2.635545509	0.008400216	0.03584534	-1.505
APBB3	207.8881968	-0.348770897	-2.634223406	0.008432998	0.03595864	-1.273
ZNF689	182.9304616	-0.34141948	-2.629892644	0.008541184	0.03634832	-1.267
RELB	148.0208924	-0.380017465	-2.629551887	0.008549748	0.03637583	-1.301
CORO6	195.9771941	0.365679054	2.626987801	0.008614442	0.03659711	1.288
ADAT2	78.71560667	-0.475533963	-2.623873941	0.008693595	0.03688812	-1.390
YEATS4	193.8082496	-0.333132184	-2.617399298	0.008860263	0.03753092	-1.260
RP11-126F18.1	8.384049188	-0.560164381	-2.61497367	0.008923433	0.03776154	-1.474
RP11-134L10.1	17.21015107	-0.601676544	-2.613552106	0.008960642	0.03790047	-1.517
RP11-582J16.5	81.42464131	0.451072767	2.612089671	0.008999065	0.03802583	1.367
C12orf45	126.0406141	-0.392618644	-2.60987077	0.009057643	0.0382267	-1.313
DKK2	32.2570481	0.577326697	2.607867289	0.009110827	0.03844178	1.492
RP11-392A22.2	126.6563146	0.397561045	2.59052873	0.009582862	0.04013997	1.317
HLA-L	206.9420126	0.34812257	2.579460233	0.009895485	0.04126973	1.273
RP11-818F20.5	23.47085987	-0.584189362	-2.574507664	0.010038286	0.04173458	-1.499
KCTD9P2	94.85304467	0.445045522	2.572867121	0.010085992	0.04185819	1.361
RRM2	162.6913065	-0.354107786	-2.566946568	0.010259842	0.04246196	-1.278
CEP95	197.9609537	-0.337692412	-2.56674899	0.010265689	0.04247601	-1.264
SUV39H2	96.64571289	-0.419020215	-2.564612161	0.010329119	0.04270784	-1.337
SMIM4	200.8642515	-0.330896868	-2.562929874	0.010379301	0.04285465	-1.258
FAM98C	173.6251518	0.344823529	2.560789294	0.010443467	0.04308801	1.270
ZNF589	138.821948	-0.36518738	-2.553983993	0.010649815	0.04382448	-1.288
U2AF1L5	75.31751221	0.407975984	2.553137548	0.010675733	0.04389983	1.327

PLAG1	172.5905524	0.340100295	2.551195663	0.010735404	0.04411377	1.266
PSMA2	178.0584309	-0.383869773	-2.550531632	0.010755876	0.04417692	-1.305
ITPKB	176.9862851	-0.358057932	-2.550376407	0.010760667	0.04418612	-1.282
RP11-399B17.1	57.75317734	0.494029982	2.550152456	0.010767582	0.04420403	1.408
RP11-1415C14.4	8.64222983	0.501616497	2.549964997	0.010773374	0.04421732	1.416
SPATA7	126.782323	-0.378521302	-2.54927296	0.010794777	0.04429466	-1.300
RP11-45F23.1	99.44218617	-0.422434716	-2.546538304	0.010879727	0.04454821	-1.340
PLEKHG3	87.4507853	-0.448471688	-2.546583081	0.010878331	0.04454821	-1.365
COL11A2	69.51334239	0.49900044	2.543038857	0.010989301	0.0449225	1.413
CCDC169	20.22510414	-0.583531806	-2.543053585	0.010988838	0.0449225	-1.499
ZNF653	58.55832766	0.48812973	2.541565648	0.011035722	0.04508033	1.403
AL449209.1	23.47003438	-0.57783614	-2.541183859	0.011047781	0.0451083	-1.493
RP11-305L7.6	8.222826777	0.543971252	2.540612731	0.011065841	0.04517139	1.458
RP11-365O16.1	135.7290907	-0.373552161	-2.537129254	0.011176568	0.045556	-1.296
TTK	87.37860882	-0.44296002	-2.53701923	0.011180081	0.045556	-1.359
FCER1G	10.17548352	-0.55523586	-2.53215275	0.011336459	0.04608667	-1.469
PRICKLE3	133.866355	-0.379095705	-2.530675535	0.011384311	0.04623157	-1.301
RUNX3	109.5284433	0.395331177	2.528444172	0.011456931	0.04647197	1.315
SPESP1	63.23751587	-0.4883937	-2.527771285	0.01147891	0.04655022	-1.403
FMO3	36.59780132	-0.545443108	-2.522957241	0.011637255	0.0471151	-1.459
RFC3	123.8329235	-0.377310405	-2.522517727	0.011651808	0.04715196	-1.299
RGS7	121.6376595	0.401861598	2.517350437	0.011824116	0.0477488	1.321
BHMT2	71.00534327	0.457621401	2.516874263	0.011840107	0.04779108	1.373
HLA-H	975.200119	0.365795081	2.515718875	0.011878989	0.04790335	1.289
RP11-317N8.2	177.0005763	-0.350672313	-2.513654164	0.011948754	0.04813983	-1.275
CTC-444N24.8	40.69103306	-0.548708473	-2.513411335	0.011956983	0.048145	-1.463
RP11-1K20.2	226.8405421	0.345634125	2.512934089	0.01197317	0.04817668	1.271
RP11-112J1.3	113.7807597	-0.393101114	-2.512891963	0.0119746	0.04817668	-1.313
CFAP44	101.3408675	0.407381557	2.509189302	0.012100862	0.04862816	1.326
SCN3A	107.6186477	0.395591834	2.504415293	0.012265397	0.04921809	1.315
2-Mar	106.8645411	-0.397035108	-2.502811536	0.012321113	0.04937572	-1.317
RP11-181C3.4	88.54293659	0.42551199	2.502053457	0.012347528	0.04947012	1.343
PLCXD2	114.8303184	0.38526969	2.501653242	0.012361493	0.04951462	1.306
DDO	30.31009626	-0.549914836	-2.500335826	0.012407563	0.04967616	-1.464
PSRC1	119.9248282	-0.387427439	-2.497673326	0.012501134	0.04999298	-1.308

Table S2. Differentially expressed MSC genes at 2 Gy, 0.66 cGy/h

	baseMean	log2FoldChange	stat	pvalue	padj	fold change
GPNMB	7539.125515	-2.697196046	-40.09763165	0	0	-6.49
CTSK	1466.868781	-3.054430477	-34.92481089	3.12E-267	2.61E-263	-8.31
ANGPTL4	5785.886841	1.841466281	21.4405241	5.60E-102	3.12E-98	3.58
SCD	11190.58489	-1.413881816	-19.55015117	4.11E-85	1.72E-81	-2.66
LOXL4	6118.414502	1.43937648	19.0888305	3.13E-81	1.04E-77	2.71
PSG5	587.1507275	1.980340047	17.94916375	4.87E-72	1.36E-68	3.95
LAMA1	897.4489368	-1.918606609	-17.50015995	1.43E-68	3.41E-65	-3.78
EGR1	2063.444476	1.25053355	17.09851188	1.52E-65	3.18E-62	2.38
KRT18	1011.612784	1.467791962	16.76562907	4.35E-63	8.08E-60	2.77
CYGB	1487.84651	-1.352814324	-16.67376477	2.03E-62	3.40E-59	-2.55
MME	540.3094504	-1.803257361	-16.43320417	1.11E-60	1.68E-57	-3.49
INHBB	917.4511016	1.477892318	16.40903915	1.65E-60	2.29E-57	2.79
HAS2	3513.708904	1.178921337	16.05966298	4.89E-58	6.29E-55	2.26
SFRP4	574.355108	-1.706636331	-15.72588992	1.01E-55	1.20E-52	-3.26
FADS2	3227.627804	-1.138163093	-15.08801521	1.94E-51	2.16E-48	-2.20
FER1L4	351.8762539	1.787077885	15.0443134	3.76E-51	3.93E-48	3.45
MAN1C1	288.1780106	-1.849069037	-14.78333003	1.88E-49	1.84E-46	-3.60
SERPINE1	137437.4969	0.939482636	14.66469115	1.09E-48	1.01E-45	1.92
DUSP1	2977.737934	1.033745913	14.63681957	1.64E-48	1.44E-45	2.05
CLEC3B	1107.699534	1.423064075	14.61083827	2.40E-48	2.00E-45	2.68
SCDP1	369.5826398	-1.888109543	-14.47205336	1.82E-47	1.45E-44	-3.70
INSIG1	2255.791393	-1.08747701	-14.41689264	4.05E-47	3.08E-44	-2.13
PLOD2	41493.78489	0.895477628	14.28687946	2.64E-46	1.92E-43	1.86
POSTN	100651.7126	-0.947485839	-14.24412194	4.88E-46	3.39E-43	-1.93
FADS1	2588.36388	-1.157357408	-13.99940893	1.57E-44	1.05E-41	-2.23
PROS1	1314.155886	-1.146692958	-13.98174777	2.01E-44	1.29E-41	-2.21
CRABP2	923.5822165	1.500136252	13.93498974	3.88E-44	2.40E-41	2.83
PLAT	4549.918096	0.965198022	13.70978743	8.87E-43	5.29E-40	1.95
SLC7A8	465.2313057	-1.540124158	-13.67320809	1.47E-42	8.45E-40	-2.91
HAS1	2636.074669	0.968446761	13.66294118	1.69E-42	9.41E-40	1.96
ARMC9	1004.933712	-1.173052018	-13.63237542	2.57E-42	1.39E-39	-2.25
PMP22	1849.276281	-1.061671966	-13.52089656	1.18E-41	6.15E-39	-2.09
TMEM119	642.5733593	-1.552139405	-13.36061982	1.03E-40	5.20E-38	-2.93
HMGCS1	1614.423905	-1.016637493	-13.18932951	1.01E-39	4.97E-37	-2.02
TNFRSF10D	5462.848567	0.96065428	13.08015534	4.28E-39	2.04E-36	1.95
IL6	1243.793135	1.222366643	13.05118926	6.26E-39	2.90E-36	2.33
ACAN	3113.843346	-1.447968345	-12.9974993	1.26E-38	5.71E-36	-2.73
MMP15	466.0092712	-1.407893992	-12.93926667	2.70E-38	1.19E-35	-2.65

SYT14	569.0350342	-1.356878001	-12.90052647	4.47E-38	1.91E-35	-2.56
TPP1	4530.124995	-1.04509849	-12.87929099	5.89E-38	2.46E-35	-2.06
IGFBP3	68035.08797	0.775800846	12.74643764	3.26E-37	1.33E-34	1.71
RP5-857K21.10	6486.232527	-0.870769936	-12.62671469	1.50E-36	5.98E-34	-1.83
COL14A1	14634.2481	-1.114356322	-12.58870807	2.44E-36	9.46E-34	-2.16
DMKN	130.7987235	-2.158845624	-12.48401806	9.13E-36	3.46E-33	-4.47
PENK	10280.4005	1.010828137	12.38701852	3.07E-35	1.14E-32	2.02
TINAGL1	2604.424198	0.953523752	12.38119597	3.30E-35	1.20E-32	1.94
ITGA7	4587.179838	1.03720858	12.24804276	1.72E-34	6.12E-32	2.05
SQSTM1	16046.74607	-0.804654857	-12.2021615	3.03E-34	1.05E-31	-1.75
OLFML2B	682.3734129	-1.378219921	-12.18654452	3.67E-34	1.25E-31	-2.60
SOD2	2632.471171	-0.921836503	-12.16542271	4.75E-34	1.59E-31	-1.89
RAB3IL1	202.252298	-1.638069696	-11.79633495	4.08E-32	1.34E-29	-3.11
ARL4C	1640.308064	-0.903789259	-11.78107162	4.89E-32	1.57E-29	-1.87
PFKFB4	601.7242936	1.152696527	11.73602393	8.33E-32	2.63E-29	2.22
RCAN1	8972.966447	0.836684727	11.71763	1.04E-31	3.20E-29	1.79
SULF1	20883.51307	-0.878166945	-11.71025953	1.13E-31	3.43E-29	-1.84
IGFBP5	18675.41339	1.170641414	11.68598207	1.50E-31	4.48E-29	2.25
BNIP3	3943.043454	0.959245451	11.68075301	1.60E-31	4.69E-29	1.94
SEMA7A	8051.962229	0.747270155	11.57638615	5.43E-31	1.56E-28	1.68
SCG2	235.126057	-1.526835689	-11.57380379	5.59E-31	1.58E-28	-2.88
MSMO1	2093.573702	-0.846180753	-11.54862943	7.50E-31	2.09E-28	-1.80
BEX1	539.6718095	1.264060776	11.43838203	2.69E-30	7.36E-28	2.40
NPC1	2913.960266	-1.088222367	-11.43591432	2.77E-30	7.45E-28	-2.13
ERRFI1	6926.395571	0.751245228	11.25094872	2.29E-29	6.08E-27	1.68
ASAH1	5406.28814	-0.794043091	-11.17771016	5.24E-29	1.37E-26	-1.73
ICAM1	187.9465189	-1.590884193	-11.14574161	7.51E-29	1.93E-26	-3.01
CYP26B1	200.1728018	1.588235722	11.05737595	2.02E-28	5.11E-26	3.01
CSF2RB	654.4588315	1.110273493	10.9348295	7.86E-28	1.96E-25	2.16
FOS	217.0868586	1.507932371	10.89495637	1.22E-27	2.99E-25	2.84
TNFRSF11B	2334.514332	0.826350807	10.84327672	2.15E-27	5.20E-25	1.77
FRMD5	395.4983707	-1.2606165	-10.76574727	5.00E-27	1.19E-24	-2.40
MMP11	251.7535351	-1.372753214	-10.76071193	5.28E-27	1.24E-24	-2.59
KISS1	109.6845525	2.073330761	10.72893192	7.45E-27	1.73E-24	4.21
P4HA1	16531.52903	0.693735748	10.68315686	1.22E-26	2.79E-24	1.62
STX3	502.5252908	-1.076881532	-10.67830161	1.29E-26	2.90E-24	-2.11
RP11-94A24.1	238.3108502	1.518825571	10.67383088	1.35E-26	3.01E-24	2.87
MT-ATP6	19115.7028	-0.792236092	-10.66042684	1.56E-26	3.43E-24	-1.73
TM4SF1	4642.649378	0.948909199	10.62928632	2.18E-26	4.72E-24	1.93
LDHA	41179.09575	0.684164723	10.60962409	2.69E-26	5.76E-24	1.61
FASN	4513.155843	-0.828997448	-10.46230241	1.29E-25	2.72E-23	-1.78

SNX9	4057.849162	-0.762592957	-10.45934777	1.33E-25	2.77E-23	-1.70
NETO2	1029.032729	0.940620363	10.41824358	2.05E-25	4.20E-23	1.92
MT-ATP8	5035.992715	-0.989947027	-10.41667706	2.08E-25	4.20E-23	-1.99
NTRK2	923.8777513	-1.036953927	-10.4163962	2.09E-25	4.20E-23	-2.05
DCLK1	129.0137944	-1.697666063	-10.41422447	2.14E-25	4.25E-23	-3.24
RP5-857K21.6	24822.08022	-0.836436654	-10.41244619	2.18E-25	4.28E-23	-1.79
MT-CO1	130078.8243	-0.718295975	-10.33806834	4.74E-25	9.21E-23	-1.65
FAM129A	1020.050292	-1.074249121	-10.31042261	6.32E-25	1.21E-22	-2.11
APLP1	1040.5016	0.880614809	10.25779683	1.09E-24	2.07E-22	1.84
NMB	630.768877	-1.181241771	-10.22917203	1.47E-24	2.75E-22	-2.27
IER3	4146.891328	0.946216388	10.19697765	2.05E-24	3.75E-22	1.93
TNS3	1924.835428	-1.010193498	-10.19700281	2.04E-24	3.75E-22	-2.01
RP3-329A5.4	36966.53928	0.954041196	10.17060931	2.68E-24	4.87E-22	1.94
GNPDA1	987.4339342	-0.945970691	-10.16520861	2.84E-24	5.09E-22	-1.93
RGS4	13872.28125	0.699214013	10.15861439	3.03E-24	5.39E-22	1.62
EDN1	533.5110484	1.058449072	10.08204728	6.63E-24	1.17E-21	2.08
PCOLCE2	1608.210444	0.793022092	10.06701417	7.73E-24	1.34E-21	1.73
MTSS1	211.9364786	-1.402398857	-10.04918511	9.26E-24	1.60E-21	-2.64
LAMP5	140.9130002	-1.571295485	-10.02089061	1.23E-23	2.10E-21	-2.97
LGALS3	2466.155239	-0.751626961	-10.01911267	1.26E-23	2.12E-21	-1.68
CTSA	10055.30367	-0.633217509	-9.999333314	1.53E-23	2.54E-21	-1.55
MT-CO3	50505.73617	-0.857990574	-9.999583311	1.53E-23	2.54E-21	-1.81
GNA14	179.2048854	1.447529669	9.995151888	1.60E-23	2.62E-21	2.73
HMGCR	1397.635039	-0.790885171	-9.955915313	2.38E-23	3.85E-21	-1.73
C5orf30	1661.120567	0.888899597	9.951074598	2.49E-23	4.01E-21	1.85
BDNF	2451.069954	0.698459762	9.930797876	3.06E-23	4.87E-21	1.62
PTGS2	331.7962696	1.17415782	9.911699439	3.70E-23	5.84E-21	2.26
DHCR24	4011.73883	-0.760603573	-9.872677649	5.47E-23	8.54E-21	-1.69
QPCT	569.3479735	0.99828737	9.856584391	6.42E-23	9.93E-21	2.00
NPC2	4222.062618	-0.820467663	-9.820607392	9.18E-23	1.41E-20	-1.77
THBS3	4881.195909	-0.793432512	-9.803028741	1.09E-22	1.66E-20	-1.73
PSG2	337.5652957	1.237518041	9.797422481	1.15E-22	1.74E-20	2.36
NDUFA4L2	1052.045138	1.128224189	9.756145579	1.74E-22	2.59E-20	2.19
IFI16	2114.81403	-0.709762563	-9.746379733	1.91E-22	2.83E-20	-1.64
AK4	3047.104564	0.72622728	9.742940537	1.98E-22	2.90E-20	1.65
DRAM1	2052.795407	-0.720505582	-9.724570248	2.37E-22	3.44E-20	-1.65
SRPX2	2318.802309	0.708734568	9.710721773	2.71E-22	3.91E-20	1.63
PGK1	18736.41854	0.693750324	9.684685022	3.50E-22	5.00E-20	1.62
TPI1	15607.79309	0.820300576	9.653910583	4.73E-22	6.70E-20	1.77
ADAMTS1	26772.9728	0.667561246	9.641465745	5.34E-22	7.50E-20	1.59
NES	1099.107496	0.96327303	9.593266609	8.53E-22	1.19E-19	1.95

TSPAN13	559.8963911	1.088452102	9.542441533	1.40E-21	1.93E-19	2.13
PPP1R3B	960.5680806	-0.889727393	-9.513195664	1.85E-21	2.53E-19	-1.85
C1S	5804.263642	-0.637572888	-9.49692854	2.16E-21	2.94E-19	-1.56
LMF2	3346.0386	0.644239653	9.488764048	2.34E-21	3.15E-19	1.56
DEPTOR	1003.31721	-0.776524487	-9.462279824	3.01E-21	4.03E-19	-1.71
UAP1L1	943.5212052	-0.827495698	-9.414612477	4.75E-21	6.30E-19	-1.77
GLMP	2027.187267	-0.69671199	-9.40939854	4.99E-21	6.56E-19	-1.62
MKI67	245.3799105	-1.252019801	-9.392979477	5.83E-21	7.61E-19	-2.38
CENPF	249.2000928	-1.217456369	-9.364319225	7.65E-21	9.91E-19	-2.33
DNAJB4	3764.169186	0.694712446	9.357530321	8.16E-21	1.05E-18	1.62
AMDHD2	615.9021647	-0.935656796	-9.346916881	9.02E-21	1.15E-18	-1.91
C1R	3315.323878	-0.857548152	-9.339331427	9.69E-21	1.23E-18	-1.81
QPRT	328.1905509	-1.132901051	-9.28194025	1.66E-20	2.09E-18	-2.19
BNIP3P	1249.504312	0.884399706	9.279185967	1.71E-20	2.13E-18	1.85
SERPING1	2023.991242	0.737333404	9.23857702	2.50E-20	3.09E-18	1.67
CHI3L1	7561.232105	-0.984147283	-9.207664765	3.33E-20	4.09E-18	-1.98
RP11-47I22.3	243.3985051	1.178867052	9.181414196	4.25E-20	5.19E-18	2.26
VAT1	12184.08359	-0.605491071	-9.141280753	6.17E-20	7.47E-18	-1.52
LDLR	2590.887594	-0.949856808	-9.093034049	9.63E-20	1.16E-17	-1.93
ITPR3	3368.220087	-1.010796861	-9.084323455	1.04E-19	1.25E-17	-2.02
RP4-646B12.2	3491.473765	-0.692430513	-9.06585409	1.24E-19	1.46E-17	-1.62
EVI2A	79.38008788	-1.752422205	-9.059606706	1.31E-19	1.54E-17	-3.37
RND3	6451.458246	-0.618874706	-9.047445543	1.46E-19	1.71E-17	-1.54
PLPPR2	2549.275776	0.636295414	9.028013512	1.75E-19	2.03E-17	1.55
HS1BP3	626.4437456	-0.839640312	-9.020923053	1.87E-19	2.15E-17	-1.79
COMP	9803.834113	-0.891823272	-9.014568997	1.98E-19	2.26E-17	-1.86
RP11-4I17.1	2670.031209	1.02804715	8.999569978	2.27E-19	2.58E-17	2.04
ATP6V1B2	2396.213593	-0.67093241	-8.985686963	2.57E-19	2.90E-17	-1.59
SLC16A3	11915.31788	0.563583591	8.969679525	2.97E-19	3.33E-17	1.48
PLEKHF1	381.6308677	-1.02410083	-8.968733352	3.00E-19	3.34E-17	-2.03
VEGFA	11156.4387	0.598902784	8.956891619	3.34E-19	3.69E-17	1.51
FNIP2	761.884553	-0.869164532	-8.912874329	4.97E-19	5.46E-17	-1.83
WASHC2C	1387.063098	-0.835932792	-8.909549563	5.12E-19	5.59E-17	-1.79
TNFRSF21	955.3277482	0.774010694	8.899466274	5.61E-19	6.09E-17	1.71
RARRES2	131.0877019	-1.436433735	-8.860811492	7.94E-19	8.56E-17	-2.71
BMPER	662.0968344	0.874643961	8.852371019	8.57E-19	9.17E-17	1.83
CDKN1A	15954.60772	0.55894277	8.81540756	1.19E-18	1.27E-16	1.47
SPAG4	155.8533769	1.425925651	8.738597581	2.36E-18	2.50E-16	2.69
P3H2	2856.204365	0.702215763	8.670844176	4.29E-18	4.51E-16	1.63
GAPDH	105419.2099	0.537812778	8.629141595	6.18E-18	6.45E-16	1.45
LHFPL2	1746.72967	-0.806729894	-8.627513521	6.27E-18	6.47E-16	-1.75

LYST	742.6555636	-1.058143845	-8.627574616	6.27E-18	6.47E-16	-2.08
RP5-1102E8.2	7276.066305	0.710847688	8.620242886	6.68E-18	6.85E-16	1.64
CYR61	23699.74975	0.620549575	8.617388272	6.85E-18	6.98E-16	1.54
STAT2	2378.276067	-0.699798766	-8.614394822	7.03E-18	7.12E-16	-1.62
ASS1	1599.350611	0.664668043	8.604404719	7.67E-18	7.72E-16	1.59
RP4-681N20.1	7033.116928	-0.702216174	-8.57482061	9.92E-18	9.93E-16	-1.63
PLAU	4687.352143	0.6075302	8.573025916	1.01E-17	1.00E-15	1.52
PCSK9	113.3427323	-1.491372404	-8.563264139	1.10E-17	1.08E-15	-2.81
GPRC5A	1817.589434	0.824006373	8.555256024	1.18E-17	1.16E-15	1.77
GLIPR1	10966.11253	0.680919126	8.527153615	1.50E-17	1.47E-15	1.60
CADM1	161.1615447	-1.249733378	-8.505873949	1.80E-17	1.75E-15	-2.38
TGFA	147.095925	1.322929225	8.50498787	1.82E-17	1.75E-15	2.50
FABP3	257.405652	-1.070123463	-8.500470343	1.89E-17	1.81E-15	-2.10
FHL2	12699.55853	0.525554502	8.493753342	2.00E-17	1.91E-15	1.44
LRRC17	383.3298754	1.007394688	8.457738309	2.73E-17	2.59E-15	2.01
KIF26B	144.2350175	-1.430104037	-8.456162275	2.76E-17	2.61E-15	-2.69
P4HA2	8504.25095	0.582574331	8.443992845	3.07E-17	2.88E-15	1.50
NR4A1	231.7323454	1.097254113	8.424926776	3.61E-17	3.37E-15	2.14
FTL	51358.40928	-0.68383284	-8.401348571	4.41E-17	4.10E-15	-1.61
GOLGA8A	1181.871249	0.834895886	8.361964828	6.17E-17	5.69E-15	1.78
SH3D21	207.1802918	1.152279529	8.341438324	7.34E-17	6.67E-15	2.22
NRP2	2095.872707	-0.744470115	-8.341390746	7.34E-17	6.67E-15	-1.68
IRS2	831.9324566	-0.78689215	-8.340844432	7.38E-17	6.67E-15	-1.73
SERTAD4	418.2307153	-0.915967202	-8.340758267	7.38E-17	6.67E-15	-1.89
RAB31	3138.184282	-0.613899388	-8.340034035	7.43E-17	6.67E-15	-1.53
DYNC2H1	613.4831302	-0.996940572	-8.321372394	8.70E-17	7.77E-15	-2.00
OSR1	351.6217881	0.936178062	8.318785729	8.89E-17	7.90E-15	1.91
TXNIP	6501.695838	0.539124276	8.317952986	8.95E-17	7.91E-15	1.45
FOXRED2	426.889159	-1.006686175	-8.306039441	9.89E-17	8.70E-15	-2.01
TMED9	12370.25554	0.617862853	8.302559979	1.02E-16	8.91E-15	1.53
FAP	8801.29795	-0.516221116	-8.28708087	1.16E-16	1.01E-14	-1.43
CLCF1	614.0096229	0.783986301	8.285265006	1.18E-16	1.02E-14	1.72
ACAT2	400.0906775	-0.923380835	-8.284349806	1.19E-16	1.02E-14	-1.90
SLC1A1	2068.962556	0.651057178	8.243963951	1.67E-16	1.43E-14	1.57
TGFBI	178201.149	0.496216879	8.217190372	2.08E-16	1.78E-14	1.41
TOP2A	449.8523971	-0.875155314	-8.214805176	2.13E-16	1.80E-14	-1.83
KRTAP2-3	394.8531916	1.022225392	8.211539293	2.18E-16	1.84E-14	2.03
PTGDS	72.54660669	-1.610619577	-8.20679099	2.27E-16	1.91E-14	-3.05
GNPTAB	2076.960839	-0.826799902	-8.188786127	2.64E-16	2.20E-14	-1.77
FTH1	107046.8648	-0.663508082	-8.158629501	3.39E-16	2.82E-14	-1.58
SULF2	764.8361081	-0.909634503	-8.151480924	3.59E-16	2.97E-14	-1.88

SERPINE2	207278.166	0.527977659	8.146376654	3.75E-16	3.09E-14	1.44
SLC1A3	328.9389315	-1.061010046	-8.127402706	4.39E-16	3.59E-14	-2.09
MIR210HG	446.869775	0.949865091	8.104799154	5.28E-16	4.31E-14	1.93
PFKFB3	1219.724459	0.694776808	8.091004584	5.92E-16	4.80E-14	1.62
CTNS	503.9597533	-0.845434268	-8.081093116	6.42E-16	5.18E-14	-1.80
FNIP1	939.9038015	-0.777619026	-8.076859609	6.65E-16	5.34E-14	-1.71
SERINC5	1180.550409	-0.752294968	-8.05959631	7.65E-16	6.12E-14	-1.68
LIF	2416.067371	0.647158279	8.043731067	8.71E-16	6.93E-14	1.57
PPFIA4	653.8421317	0.756842056	8.036519174	9.24E-16	7.32E-14	1.69
SLC22A17	744.8998896	0.722498716	8.029299581	9.80E-16	7.72E-14	1.65
HDAC9	259.0329748	-1.006220175	-8.017996205	1.07E-15	8.43E-14	-2.01
WNK4	160.2502895	1.199042043	8.009098375	1.16E-15	9.02E-14	2.30
IDI1	1371.281237	-0.672779704	-7.943116139	1.97E-15	1.53E-13	-1.59
NEFM	396.2123546	1.160433738	7.939149327	2.04E-15	1.57E-13	2.24
PGF	1328.624158	0.70850093	7.900892744	2.77E-15	2.13E-13	1.63
SRGN	14322.32696	0.687361495	7.881001663	3.25E-15	2.49E-13	1.61
APP	20827.66642	-0.494642107	-7.88057656	3.26E-15	2.49E-13	-1.41
DACT1	1690.639528	-0.756322048	-7.854167208	4.02E-15	3.06E-13	-1.69
PER1	283.4807991	0.93605816	7.840190683	4.50E-15	3.40E-13	1.91
GRN	11955.52365	-0.557871292	-7.821687496	5.21E-15	3.92E-13	-1.47
RASSF7	206.8323736	1.083011721	7.808773669	5.77E-15	4.33E-13	2.12
RAC2	799.532153	0.705740668	7.805617926	5.92E-15	4.42E-13	1.63
KCNH1	173.3072739	-1.249894807	-7.801477974	6.12E-15	4.54E-13	-2.38
SLC40A1	83.21088649	-1.414748835	-7.796565016	6.36E-15	4.70E-13	-2.67
FTHL11	1773.99668	-0.689038637	-7.785042613	6.97E-15	5.13E-13	-1.61
CSF1	2541.72822	-0.578791959	-7.771253028	7.77E-15	5.69E-13	-1.49
IGFBP4	147041.4308	0.568372908	7.768518202	7.94E-15	5.79E-13	1.48
CLCN7	2004.274168	-0.57534427	-7.749819987	9.20E-15	6.68E-13	-1.49
DBNDD1	197.2430025	1.069277075	7.726481209	1.11E-14	8.00E-13	2.10
PEPD	1410.316114	-0.624348002	-7.724343501	1.12E-14	8.10E-13	-1.54
EEF1A2	85.32840746	1.43558543	7.722285798	1.14E-14	8.19E-13	2.70
LSS	2546.316063	-0.613762688	-7.702171724	1.34E-14	9.51E-13	-1.53
POFUT2	5602.960448	0.539499565	7.686498166	1.51E-14	1.07E-12	1.45
MYOCD	208.0998308	1.113049886	7.684350098	1.54E-14	1.08E-12	2.16
STC1	777.7799459	0.755968651	7.67675379	1.63E-14	1.15E-12	1.69
ALPL	547.2738336	0.755099522	7.672938822	1.68E-14	1.17E-12	1.69
DVL2	675.0905317	-0.738818645	-7.664245435	1.80E-14	1.25E-12	-1.67
CD248	15992.69216	0.510487183	7.628892147	2.37E-14	1.64E-12	1.42
OXCT1	1178.490997	-0.611394763	-7.624371432	2.45E-14	1.69E-12	-1.53
CCDC80	65347.94245	0.51089676	7.61822587	2.57E-14	1.77E-12	1.42
ZCCHC14	1047.766737	-0.721080366	-7.609074929	2.76E-14	1.89E-12	-1.65

PDK4	120.4475242	1.24422722	7.603213857	2.89E-14	1.97E-12	2.37
CRELD1	1240.935588	0.6886105	7.600432702	2.95E-14	2.00E-12	1.61
CYB561	1227.881668	0.634432374	7.581586775	3.41E-14	2.31E-12	1.55
PGM1	3923.190712	0.504370452	7.578312918	3.50E-14	2.36E-12	1.42
IL11	288.4390052	0.924540172	7.576790397	3.54E-14	2.38E-12	1.90
PRELP	792.7794757	-0.727621383	-7.560219794	4.02E-14	2.69E-12	-1.66
MCOLN3	68.06936241	-1.524977985	-7.55795022	4.09E-14	2.73E-12	-2.88
NMT2	1224.928331	0.635013101	7.553385423	4.24E-14	2.81E-12	1.55
NCR3LG1	194.6733895	-1.294124524	-7.54829834	4.41E-14	2.91E-12	-2.45
MT-ND3	3021.273262	-0.624263137	-7.540270157	4.69E-14	3.08E-12	-1.54
MYCBP	183.4488493	1.055348972	7.520300921	5.47E-14	3.58E-12	2.08
COL11A1	1394.644076	-0.734696929	-7.49732753	6.51E-14	4.25E-12	-1.66
RENBP	47.3661866	-1.660317472	-7.47198503	7.90E-14	5.13E-12	-3.16
CLEC11A	2271.704004	0.706146013	7.463931263	8.40E-14	5.44E-12	1.63
BNIP3L	7798.823948	0.471685687	7.456502994	8.88E-14	5.73E-12	1.39
FAM162A	868.296021	0.783175682	7.454960709	8.99E-14	5.78E-12	1.72
WNT5B	3684.008101	0.609103901	7.452939366	9.13E-14	5.84E-12	1.53
RAB27B	532.355701	-0.870978787	-7.448270384	9.46E-14	6.03E-12	-1.83
PLIN2	3229.195578	0.564804085	7.445140707	9.68E-14	6.15E-12	1.48
GJA5	54.1707363	1.560483106	7.443935891	9.77E-14	6.18E-12	2.95
SNCG	247.5129101	1.015352816	7.43558174	1.04E-13	6.56E-12	2.02
CTSD	17655.7936	-0.560571216	-7.431654172	1.07E-13	6.74E-12	-1.47
ASPM	134.0073211	-1.163480056	-7.402892571	1.33E-13	8.34E-12	-2.24
DHCR7	1682.548033	-0.744505828	-7.394862032	1.42E-13	8.82E-12	-1.68
ABCC4	752.411467	-0.764448354	-7.32945205	2.31E-13	1.44E-11	-1.70
SMOC1	844.7284737	-0.653701381	-7.326546796	2.36E-13	1.46E-11	-1.57
MYO1D	1637.539522	-0.644987693	-7.325262236	2.38E-13	1.47E-11	-1.56
FNDC1	3984.757376	-0.854713124	-7.317964737	2.52E-13	1.55E-11	-1.81
H1F0	1962.032593	0.57662638	7.310473879	2.66E-13	1.63E-11	1.49
PIEZO2	557.5809195	-0.952618227	-7.29646239	2.95E-13	1.80E-11	-1.94
KRTAP1-5	1559.251049	0.611370222	7.289295058	3.12E-13	1.89E-11	1.53
CLU	940.5140731	0.636165625	7.260041108	3.87E-13	2.34E-11	1.55
ERO1A	4021.534485	0.476523369	7.243115354	4.38E-13	2.64E-11	1.39
MAF	118.3738418	-1.186263827	-7.241936401	4.42E-13	2.66E-11	-2.28
MYH3	166.4914852	1.047235401	7.231281529	4.78E-13	2.86E-11	2.07
ACADVL	8364.869066	0.55392053	7.218957988	5.24E-13	3.12E-11	1.47
VAC14	716.2356036	-0.689738204	-7.218621533	5.25E-13	3.12E-11	-1.61
LOX	105121.523	0.555896898	7.201608916	5.95E-13	3.53E-11	1.47
MTHFD1	873.4204335	-0.634558283	-7.196255779	6.19E-13	3.65E-11	-1.55
PCYT2	901.2407812	-0.604379232	-7.191472627	6.41E-13	3.77E-11	-1.52
SLC17A9	571.5200261	0.787505531	7.168111897	7.60E-13	4.46E-11	1.73

MT-ND4	90422.69786	-0.497353723	-7.155216771	8.35E-13	4.88E-11	-1.41
ENPP2	1374.665442	-0.567788724	-7.149378753	8.72E-13	5.06E-11	-1.48
THBS2	49747.24174	-0.606297634	-7.149183353	8.73E-13	5.06E-11	-1.52
IFIT3	168.9252285	-1.069975114	-7.118229433	1.09E-12	6.32E-11	-2.10
NAV2	446.4928483	-0.922958862	-7.116747258	1.11E-12	6.37E-11	-1.90
EPAS1	19512.89182	-0.593799887	-7.113364726	1.13E-12	6.50E-11	-1.51
C19orf10	7409.766184	0.642373273	7.105937688	1.20E-12	6.84E-11	1.56
PODN	737.5774136	-0.662359786	-7.104990307	1.20E-12	6.86E-11	-1.58
PLAUR	2446.858	0.58816024	7.104281347	1.21E-12	6.87E-11	1.50
COL4A3BP	1344.162206	-0.622677765	-7.094731466	1.30E-12	7.34E-11	-1.54
RP11-887P2.3	1246.292671	0.624368672	7.09223185	1.32E-12	7.45E-11	1.54
CPNE8	202.3194321	-0.948323549	-7.068172799	1.57E-12	8.83E-11	-1.93
PRSS3	82.3941307	1.34963656	7.045633637	1.85E-12	1.03E-10	2.55
CSTB	2994.798555	-0.529356844	-7.032092185	2.03E-12	1.14E-10	-1.44
LAMP2	7484.926492	-0.488743	-7.023215616	2.17E-12	1.21E-10	-1.40
PLXDC2	1709.505782	-0.639230685	-7.022410518	2.18E-12	1.21E-10	-1.56
SQLE	1733.606827	-0.52382704	-7.019359939	2.23E-12	1.23E-10	-1.44
SPP1	77.60797793	-1.32544015	-7.011154717	2.36E-12	1.30E-10	-2.51
DBI	1814.737124	-0.569364643	-7.007784426	2.42E-12	1.33E-10	-1.48
PPT1	1739.757815	-0.529905357	-7.001639176	2.53E-12	1.39E-10	-1.44
HHIP	260.3200705	0.861796774	6.991966024	2.71E-12	1.48E-10	1.82
SGCE	1085.409867	0.601563992	6.987718268	2.79E-12	1.52E-10	1.52
NREP	4523.674921	0.462595359	6.976386822	3.03E-12	1.64E-10	1.38
THY1	26000.82809	0.444667948	6.97523846	3.05E-12	1.65E-10	1.36
C14orf159	316.323247	-0.79581172	-6.970824542	3.15E-12	1.70E-10	-1.74
IGFBP6	4887.157831	0.573906171	6.968396994	3.21E-12	1.72E-10	1.49
KIRREL3	409.2392126	0.777815574	6.959736353	3.41E-12	1.83E-10	1.71
KIF20A	126.709962	-1.131488748	-6.953231126	3.57E-12	1.91E-10	-2.19
TBC1D12	532.48984	-0.698875019	-6.947363357	3.72E-12	1.98E-10	-1.62
RAB29	661.5645069	-0.651362791	-6.931261521	4.17E-12	2.21E-10	-1.57
ANLN	320.5831867	-0.857953299	-6.929514962	4.22E-12	2.23E-10	-1.81
IGFBP2	31901.08224	0.562271372	6.906681813	4.96E-12	2.61E-10	1.48
SMIM3	2999.074659	0.486986724	6.898547025	5.25E-12	2.76E-10	1.40
LYPD1	753.1242587	0.650212698	6.897105976	5.31E-12	2.78E-10	1.57
KRT7	4975.030737	0.558741944	6.890860216	5.55E-12	2.89E-10	1.47
MSC	263.4416826	-0.852276459	-6.87166063	6.35E-12	3.30E-10	-1.81
AOX1	1236.630598	0.61943121	6.846651835	7.56E-12	3.92E-10	1.54
LEP	113.2360375	1.125968929	6.844241661	7.69E-12	3.98E-10	2.18
CITED2	5065.827736	0.48138798	6.836391839	8.12E-12	4.19E-10	1.40
RRAGC	1141.41632	-0.57562998	-6.826335919	8.71E-12	4.48E-10	-1.49
RP11-490H24.5	821.6720211	0.674749791	6.825535404	8.76E-12	4.49E-10	1.60

FBLN5	2409.447609	-0.499713929	-6.807007246	9.96E-12	5.09E-10	-1.41
CADPS2	97.20942441	-1.175706495	-6.78618533	1.15E-11	5.86E-10	-2.26
PGAM1	3127.98248	0.533790936	6.78504688	1.16E-11	5.89E-10	1.45
NEK7	17224.87804	0.480553525	6.780511427	1.20E-11	6.06E-10	1.40
FOXM1	217.020625	-0.883819239	-6.776120668	1.23E-11	6.23E-10	-1.85
TRIB3	1591.219097	-0.512145769	-6.758429793	1.39E-11	7.02E-10	-1.43
UAP1	4644.05939	0.483828636	6.757184581	1.41E-11	7.06E-10	1.40
GDF15	1958.025935	-0.525424653	-6.740081497	1.58E-11	7.92E-10	-1.44
SLC7A11	1993.356397	-0.654826859	-6.738856576	1.60E-11	7.96E-10	-1.57
LARGE	814.3339421	-0.651944704	-6.731713173	1.68E-11	8.34E-10	-1.57
PLA2G4C	513.0715439	-0.662879585	-6.722769433	1.78E-11	8.84E-10	-1.58
WASHC5	1906.237742	-0.629342906	-6.702268862	2.05E-11	1.01E-09	-1.55
FOXQ1	121.1280535	-1.099206397	-6.701936082	2.06E-11	1.01E-09	-2.14
CCND3	902.8332593	0.569358386	6.700237167	2.08E-11	1.02E-09	1.48
ERGIC1	10604.48579	0.425323554	6.675875046	2.46E-11	1.20E-09	1.34
APOL6	358.6898222	-0.784119988	-6.658335479	2.77E-11	1.35E-09	-1.72
CYP1B1	19003.28498	-0.465777097	-6.654605623	2.84E-11	1.38E-09	-1.38
REEP2	217.9277415	0.945338174	6.649330241	2.94E-11	1.43E-09	1.93
CDC25B	1040.706908	-0.572871124	-6.6458285	3.02E-11	1.46E-09	-1.49
DUSP6	935.9140596	0.599167915	6.645071453	3.03E-11	1.46E-09	1.51
CDH13	4844.678266	-0.614955696	-6.635072409	3.24E-11	1.56E-09	-1.53
AC009302.3	759.7703178	-0.583079461	-6.633672163	3.27E-11	1.57E-09	-1.50
WDR81	902.3230071	-0.606775945	-6.628341157	3.39E-11	1.62E-09	-1.52
SERPINB7	247.4831905	0.883385149	6.627187792	3.42E-11	1.63E-09	1.84
MFSD10	1989.564243	0.549382372	6.627014174	3.43E-11	1.63E-09	1.46
AFF2	99.14069909	-1.255673088	-6.620930625	3.57E-11	1.69E-09	-2.39
ANGPTL2	942.2466431	-0.678067815	-6.62020256	3.59E-11	1.70E-09	-1.60
MECOM	330.6379053	-0.775407397	-6.616235185	3.68E-11	1.74E-09	-1.71
MASP1	260.491254	0.925641994	6.610092866	3.84E-11	1.81E-09	1.90
IDH1	1947.105175	-0.509959341	-6.597046138	4.19E-11	1.97E-09	-1.42
CTGF	75881.28665	0.456504565	6.593747917	4.29E-11	2.01E-09	1.37
TRPM7	1824.281586	-0.612728483	-6.587617859	4.47E-11	2.09E-09	-1.53
CASP4	724.6951019	-0.610984097	-6.583614336	4.59E-11	2.14E-09	-1.53
CYBA	983.2307036	0.642798547	6.568574152	5.08E-11	2.36E-09	1.56
TMEM140	256.7809176	-0.819745189	-6.564871453	5.21E-11	2.41E-09	-1.77
OAS2	186.8169136	-0.980312468	-6.562414351	5.29E-11	2.44E-09	-1.97
MEX3B	327.2686405	-0.815929815	-6.557207922	5.48E-11	2.52E-09	-1.76
ADAM15	3877.114225	0.465974337	6.5550739	5.56E-11	2.55E-09	1.38
CRISPLD1	185.3890661	-0.89696219	-6.550719325	5.73E-11	2.62E-09	-1.86
COL7A1	1295.536373	0.564092272	6.541451588	6.09E-11	2.78E-09	1.48
SDC4	3207.873399	0.556177373	6.533265078	6.44E-11	2.93E-09	1.47

C10orf10	1681.471733	-0.512405989	-6.527372531	6.69E-11	3.04E-09	-1.43
SLFN5	4708.342363	-0.722573703	-6.515979874	7.22E-11	3.27E-09	-1.65
COL13A1	470.7483019	0.660415455	6.508481257	7.59E-11	3.43E-09	1.58
RGMB	4572.474975	0.487391912	6.507338678	7.65E-11	3.44E-09	1.40
CD151	25725.891	0.426442391	6.486689007	8.77E-11	3.93E-09	1.34
CDK4	2061.320566	-0.539466775	-6.487021796	8.75E-11	3.93E-09	-1.45
CKB	369.7568259	0.737771191	6.484840004	8.88E-11	3.97E-09	1.67
MYBL1	948.5507439	0.570820598	6.480992149	9.11E-11	4.06E-09	1.49
PFKFB2	223.5709837	-0.854186397	-6.478827672	9.24E-11	4.11E-09	-1.81
PRRX1	4867.490641	-0.474051136	-6.47777222	9.31E-11	4.12E-09	-1.39
B4GALT7	1159.93282	0.526593383	6.465804082	1.01E-10	4.45E-09	1.44
CHIC2	1014.032857	0.617536162	6.459425239	1.05E-10	4.63E-09	1.53
FAM149A	80.379235	-1.178430584	-6.455408197	1.08E-10	4.74E-09	-2.26
ADM	1359.849724	0.542948862	6.453008471	1.10E-10	4.81E-09	1.46
CNN1	1048.064272	0.591054482	6.450576087	1.11E-10	4.87E-09	1.51
TIMP1	52144.80792	0.522203513	6.443179616	1.17E-10	5.10E-09	1.44
OLFML1	59.03945897	-1.300221902	-6.442684612	1.17E-10	5.11E-09	-2.46
TGM2	29433.74384	0.56553044	6.437391755	1.22E-10	5.27E-09	1.48
RAB7A	7747.168095	-0.42091135	-6.435213089	1.23E-10	5.34E-09	-1.34
GBA	3303.809077	-0.45262656	-6.427635198	1.30E-10	5.59E-09	-1.37
MT2A	6499.837361	0.665485864	6.416192592	1.40E-10	6.00E-09	1.59
FHOD3	558.7487797	-0.731482741	-6.416345516	1.40E-10	6.00E-09	-1.66
GALNT16	233.7050726	-0.809646196	-6.403992635	1.51E-10	6.48E-09	-1.75
TMEM30A	7507.060174	0.45294565	6.403528122	1.52E-10	6.49E-09	1.37
LITAF	2335.056167	-0.458932713	-6.393794451	1.62E-10	6.90E-09	-1.37
NXPH4	342.2055382	0.787074375	6.393362585	1.62E-10	6.90E-09	1.73
PGD	2414.207538	-0.462174654	-6.38604483	1.70E-10	7.22E-09	-1.38
FZD4	693.0005781	-0.625442434	-6.385240654	1.71E-10	7.22E-09	-1.54
MT-ND4L	11508.52458	-0.686572858	-6.38515232	1.71E-10	7.22E-09	-1.61
M6PR	2045.1768	-0.497251545	-6.382757922	1.74E-10	7.32E-09	-1.41
GREM1	4762.061741	0.57062052	6.361104573	2.00E-10	8.41E-09	1.49
LAMP1	15730.54585	-0.41809057	-6.360230356	2.01E-10	8.43E-09	-1.34
SLC2A1	5674.231857	0.493861779	6.344829887	2.23E-10	9.28E-09	1.41
CCNA2	158.3210245	-0.930704198	-6.345099684	2.22E-10	9.28E-09	-1.91
ORMDL3	2226.215897	0.490533078	6.339873064	2.30E-10	9.56E-09	1.40
ERV3-1	298.5697832	-0.857526989	-6.337868222	2.33E-10	9.66E-09	-1.81
P3H3	5403.925789	0.478396892	6.329490936	2.46E-10	1.02E-08	1.39
GYS1	2991.113446	0.459871781	6.326193634	2.51E-10	1.04E-08	1.38
SC5D	1985.282302	-0.459478195	-6.30491219	2.88E-10	1.19E-08	-1.38
PVR	4718.887657	0.444748325	6.291551046	3.14E-10	1.29E-08	1.36
SLC25A4	1378.249453	0.587487526	6.28650142	3.25E-10	1.33E-08	1.50

SDC1	429.9400817	-0.685226129	-6.271769759	3.57E-10	1.46E-08	-1.61
HMOX1	2301.69418	-0.474462142	-6.269842749	3.61E-10	1.47E-08	-1.39
FBXO32	1579.227882	-0.525691761	-6.255135248	3.97E-10	1.61E-08	-1.44
MPP2	108.9364503	1.029908983	6.249445013	4.12E-10	1.67E-08	2.04
CARS2	858.5959588	-0.551405241	-6.246262647	4.20E-10	1.70E-08	-1.47
APLN	73.75330218	1.1804823	6.241482528	4.33E-10	1.75E-08	2.27
MAP2K1	1715.423692	0.482179573	6.237752683	4.44E-10	1.78E-08	1.40
RALA	2158.153612	-0.499201395	-6.237724047	4.44E-10	1.78E-08	-1.41
ADGRL1	290.5381718	0.737431604	6.232609237	4.59E-10	1.84E-08	1.67
LXN	508.8715547	0.624894572	6.224511889	4.83E-10	1.93E-08	1.54
GAS6	40395.49034	0.383151096	6.222169432	4.90E-10	1.95E-08	1.30
PNPLA2	2839.31003	0.455176293	6.218409662	5.02E-10	2.00E-08	1.37
HAPLN3	837.1480617	-0.57324479	-6.213708339	5.17E-10	2.05E-08	-1.49
FTHL10	3861.600034	-0.564045947	-6.212031548	5.23E-10	2.07E-08	-1.48
SFRP2	206.1304499	-0.826487724	-6.209402514	5.32E-10	2.10E-08	-1.77
ANTXR1	8679.137468	-0.548874153	-6.208246969	5.36E-10	2.11E-08	-1.46
WDR19	554.6773697	-0.642275068	-6.206392499	5.42E-10	2.13E-08	-1.56
SLC9A7	2182.29702	0.496072683	6.195762651	5.80E-10	2.27E-08	1.41
PTPRQ	196.070939	0.91744401	6.189224488	6.05E-10	2.36E-08	1.89
CERCAM	10815.19806	0.413480341	6.162539238	7.16E-10	2.79E-08	1.33
HTR2B	26.45861929	-1.445146342	-6.160596574	7.25E-10	2.82E-08	-2.72
CLDN11	441.0011801	0.659885448	6.158536355	7.34E-10	2.85E-08	1.58
ATP6V1A	2436.399533	-0.457900579	-6.157388283	7.40E-10	2.86E-08	-1.37
SLC2A5	135.7679128	0.943917692	6.14780158	7.86E-10	3.03E-08	1.92
MAPKAPK3	1080.736338	-0.513153799	-6.134351466	8.55E-10	3.29E-08	-1.43
FOLR3	63.06815161	-1.201605108	-6.133040317	8.62E-10	3.31E-08	-2.30
LRRC32	3343.382702	-0.537356278	-6.13103324	8.73E-10	3.34E-08	-1.45
CC2D2A	388.3232556	-0.69848266	-6.130956253	8.74E-10	3.34E-08	-1.62
NR4A2	199.6638426	0.853018249	6.125354337	9.05E-10	3.45E-08	1.81
EFS	291.5899563	0.723664429	6.11978056	9.37E-10	3.57E-08	1.65
ZNF395	1318.708939	0.510504752	6.117744734	9.49E-10	3.60E-08	1.42
ZFYVE26	723.9945764	-0.675959271	-6.115940903	9.60E-10	3.64E-08	-1.60
NDRG1	4924.587051	0.413910989	6.109846991	9.97E-10	3.77E-08	1.33
FHL1	6994.62649	0.417791714	6.103592766	1.04E-09	3.91E-08	1.34
HIST1H1C	751.7882803	0.651824519	6.103105937	1.04E-09	3.91E-08	1.57
ADIRF	1684.234541	0.551566593	6.102416269	1.04E-09	3.92E-08	1.47
TXNDC15	3229.045757	0.426590534	6.102328537	1.05E-09	3.92E-08	1.34
CYB5RL	173.0518813	-0.872573146	-6.100821243	1.06E-09	3.94E-08	-1.83
SLC38A6	314.4578812	-0.714218122	-6.097478482	1.08E-09	4.02E-08	-1.64
TBX3	1111.994566	-0.542724165	-6.095485006	1.09E-09	4.06E-08	-1.46
NPDC1	2527.783966	0.527251531	6.086999401	1.15E-09	4.27E-08	1.44

RABAC1	2286.141346	0.555385994	6.08253931	1.18E-09	4.38E-08	1.47
LY6E	4454.808937	0.462548043	6.076790556	1.23E-09	4.53E-08	1.38
VPS13C	1190.126123	-0.719660326	-6.072966876	1.26E-09	4.63E-08	-1.65
PRDX2	3154.749672	0.530440366	6.071381471	1.27E-09	4.67E-08	1.44
TSPAN31	797.0606729	0.532025592	6.052081306	1.43E-09	5.25E-08	1.45
IGF2R	14127.08884	-0.58864385	-6.051216904	1.44E-09	5.27E-08	-1.50
COL8A1	35566.93813	0.480777401	6.050621356	1.44E-09	5.27E-08	1.40
MEDAG	1650.392421	0.453641137	6.046539	1.48E-09	5.40E-08	1.37
INA	171.665117	0.943519802	6.045561392	1.49E-09	5.42E-08	1.92
ANKRD37	660.8902822	0.641648311	6.044740705	1.50E-09	5.42E-08	1.56
FKBP11	1397.667937	0.472051312	6.04506185	1.49E-09	5.42E-08	1.39
PHLDA1	3508.267533	-0.423043049	-6.035887092	1.58E-09	5.72E-08	-1.34
CSRNP1	438.9767443	0.661169996	6.034624643	1.59E-09	5.74E-08	1.58
RP11-16M8.2	763.9414917	0.546188531	6.034610501	1.59E-09	5.74E-08	1.46
CRISPLD2	997.8086704	-0.654289986	-6.029028263	1.65E-09	5.93E-08	-1.57
ACTA2	14785.31965	-0.380051683	-6.01218816	1.83E-09	6.55E-08	-1.30
TAF9B	161.3602692	-0.886828268	-6.0123787	1.83E-09	6.55E-08	-1.85
TMED3	7823.345753	0.504608475	6.011544439	1.84E-09	6.56E-08	1.42
TST	743.7884266	0.579493191	5.990666401	2.09E-09	7.44E-08	1.49
DTX3L	328.9577631	-0.725574288	-5.989100813	2.11E-09	7.50E-08	-1.65
PTGS1	129.8308164	0.963473697	5.982543041	2.20E-09	7.79E-08	1.95
BASP1	5365.261385	-0.488966363	-5.982323191	2.20E-09	7.79E-08	-1.40
ERMN	272.4561959	0.730607126	5.977342565	2.27E-09	8.01E-08	1.66
S100A16	3833.674555	0.453215278	5.972420231	2.34E-09	8.24E-08	1.37
PYCR1	3825.843213	0.413713494	5.96925018	2.38E-09	8.38E-08	1.33
ETFB	998.0435915	0.547223248	5.95967823	2.53E-09	8.87E-08	1.46
ABI3BP	9829.002697	-0.599112092	-5.954400703	2.61E-09	9.14E-08	-1.51
TMED1	1013.327053	0.52886967	5.937217026	2.90E-09	1.01E-07	1.44
SPON2	258.1389507	-0.754099736	-5.936993036	2.90E-09	1.01E-07	-1.69
PDLIM1	1056.09564	0.562251689	5.933985115	2.96E-09	1.03E-07	1.48
NLGN2	2343.823963	0.440017162	5.934131393	2.95E-09	1.03E-07	1.36
RECK	5133.565378	0.477660609	5.933004482	2.97E-09	1.03E-07	1.39
NEU1	1648.333375	-0.512900639	-5.930835398	3.01E-09	1.04E-07	-1.43
RP11-498J9.1	180.0608808	0.872832284	5.928967047	3.05E-09	1.05E-07	1.83
SNX8	1137.848888	-0.502026273	-5.926665592	3.09E-09	1.06E-07	-1.42
PRKG1	258.3146252	-0.743578813	-5.923096191	3.16E-09	1.09E-07	-1.67
ACACA	1655.108467	-0.631471337	-5.922546389	3.17E-09	1.09E-07	-1.55
HIP1	1470.051889	-0.717288321	-5.919023322	3.24E-09	1.11E-07	-1.64
P3H4	3364.797769	0.419937341	5.913179405	3.36E-09	1.15E-07	1.34
SEC61G	2660.023234	0.624354907	5.912212521	3.38E-09	1.15E-07	1.54
BMF	101.7463996	-1.062973644	-5.911526221	3.39E-09	1.15E-07	-2.09

PABPC3	2667.114481	-0.431292752	-5.908441346	3.45E-09	1.17E-07	-1.35
IMPDH1	1768.803229	0.442402889	5.900903886	3.62E-09	1.22E-07	1.36
ENO1	32484.60785	0.444549574	5.899568821	3.64E-09	1.23E-07	1.36
PRKCE	371.2020895	-0.65161465	-5.899136009	3.65E-09	1.23E-07	-1.57
GPC6	1067.299041	-0.598532365	-5.895457975	3.74E-09	1.26E-07	-1.51
HEXB	4361.112205	-0.444421622	-5.89395246	3.77E-09	1.27E-07	-1.36
VOPP1	1722.185882	0.527156085	5.891456453	3.83E-09	1.28E-07	1.44
ATP6V1C1	1626.58381	-0.442492607	-5.891193778	3.83E-09	1.28E-07	-1.36
TMEM263	5024.90479	0.400600615	5.88138998	4.07E-09	1.36E-07	1.32
COA7	473.5992248	-0.604801491	-5.878075714	4.15E-09	1.38E-07	-1.52
PNMA2	630.070052	0.579216977	5.864033781	4.52E-09	1.50E-07	1.49
TXNDC16	186.6022017	-0.831307735	-5.854939023	4.77E-09	1.58E-07	-1.78
AMIGO2	2005.397243	-0.450558417	-5.849760268	4.92E-09	1.63E-07	-1.37
COL6A3	107126.2226	-0.592697849	-5.844782571	5.07E-09	1.67E-07	-1.51
FLCN	760.6460848	-0.540872696	-5.843531398	5.11E-09	1.68E-07	-1.45
RP5-864K19.4	74.91331344	-1.12305862	-5.842910892	5.13E-09	1.69E-07	-2.18
KRTCAP2	233.2560486	0.781112717	5.840918214	5.19E-09	1.70E-07	1.72
ARFGAP1	2947.079134	0.435253549	5.829590553	5.56E-09	1.82E-07	1.35
RPS19	7213.012634	0.553554134	5.823966158	5.75E-09	1.88E-07	1.47
MYOF	14560.33927	-0.591086273	-5.822957809	5.78E-09	1.89E-07	-1.51
TRIM47	126.0662034	-0.903766102	-5.816313439	6.02E-09	1.96E-07	-1.87
SEC14L2	643.8008496	0.601450082	5.79698414	6.75E-09	2.19E-07	1.52
UNC5B	2946.587748	-0.529759634	-5.797006931	6.75E-09	2.19E-07	-1.44
MT1L	90.95134964	1.022220869	5.791417533	6.98E-09	2.26E-07	2.03
KIAA1644	3585.444055	-0.635046019	-5.785169859	7.24E-09	2.34E-07	-1.55
C5orf46	212.7489974	0.783884899	5.780076663	7.47E-09	2.41E-07	1.72
RP11-1A15.2	129.064915	-0.890489902	-5.778924219	7.52E-09	2.42E-07	-1.85
TMED4	2026.894772	0.434844009	5.776597109	7.62E-09	2.45E-07	1.35
KRTAP1-1	212.4414677	0.750610374	5.774124755	7.74E-09	2.48E-07	1.68
COL15A1	439.1839895	-0.84503448	-5.772399344	7.82E-09	2.50E-07	-1.80
KCNK15	91.43970049	-0.998755838	-5.760330402	8.39E-09	2.68E-07	-2.00
BST1	1352.234077	0.443816306	5.755084202	8.66E-09	2.76E-07	1.36
CGNL1	124.9941387	-0.927496702	-5.753105726	8.76E-09	2.79E-07	-1.90
APOE	43.41063115	-1.226376631	-5.751898623	8.82E-09	2.80E-07	-2.34
WISP2	414.95042	-0.60023586	-5.750944217	8.87E-09	2.81E-07	-1.52
IRAK1	3947.725912	-0.394819001	-5.74601626	9.14E-09	2.89E-07	-1.31
ADGRA2	1743.380992	-0.554865782	-5.744766308	9.20E-09	2.91E-07	-1.47
LAYN	1343.10452	0.477620444	5.742451114	9.33E-09	2.94E-07	1.39
L29074.1	2464.068746	-0.584716397	-5.739782895	9.48E-09	2.98E-07	-1.50
FLRT2	912.6566734	-0.558312226	-5.738165225	9.57E-09	3.01E-07	-1.47
UBAP2L	4221.499192	-0.452144145	-5.737821258	9.59E-09	3.01E-07	-1.37

CCL2	785.7619397	0.528880616	5.735214878	9.74E-09	3.05E-07	1.44
HES1	99.9858305	-0.985179227	-5.725249978	1.03E-08	3.22E-07	-1.98
RHBDD2	1459.462212	0.466419069	5.72300206	1.05E-08	3.26E-07	1.38
RP11-492E3.2	71.64338946	1.097162924	5.720267723	1.06E-08	3.31E-07	2.14
SELT	2483.407222	0.428414009	5.719711197	1.07E-08	3.31E-07	1.35
CYBRD1	8206.399951	-0.43901393	-5.718719932	1.07E-08	3.33E-07	-1.36
OSBPL8	4087.90406	-0.506369076	-5.716641245	1.09E-08	3.36E-07	-1.42
APOLD1	195.9702634	0.829221929	5.715603053	1.09E-08	3.38E-07	1.78
RFNG	1262.251586	0.451646551	5.708140839	1.14E-08	3.52E-07	1.37
EPGN	332.3252983	0.709243057	5.707192871	1.15E-08	3.53E-07	1.63
WASHC2A	1503.679249	-0.550281744	-5.706148451	1.16E-08	3.55E-07	-1.46
TUBA4A	1192.029583	0.563471068	5.70551923	1.16E-08	3.56E-07	1.48
TCAF2	111.8719142	0.930570678	5.704780817	1.16E-08	3.56E-07	1.91
RASSF1	823.3347461	0.518216643	5.699220804	1.20E-08	3.68E-07	1.43
SLC38A7	1225.148694	-0.525211224	-5.692001689	1.26E-08	3.83E-07	-1.44
FMNL2	822.3995791	-0.60286529	-5.690455635	1.27E-08	3.86E-07	-1.52
AC092066.1	368.045969	0.655193543	5.679329748	1.35E-08	4.11E-07	1.57
NOG	327.8314263	0.646771725	5.678676492	1.36E-08	4.12E-07	1.57
IGSF1	149.5144457	0.844980008	5.676480625	1.37E-08	4.16E-07	1.80
FKBP10	21936.44397	0.366402636	5.66872311	1.44E-08	4.35E-07	1.29
C11orf87	678.5467541	0.577189895	5.663736402	1.48E-08	4.47E-07	1.49
RP5-827C21.1	868.3022238	0.536998699	5.660998601	1.50E-08	4.53E-07	1.45
F2R	1301.803557	-0.485945059	-5.66044401	1.51E-08	4.54E-07	-1.40
DYRK3	396.0238989	-0.603393202	-5.657279964	1.54E-08	4.61E-07	-1.52
LIMK1	2813.154017	-0.401700808	-5.65280074	1.58E-08	4.73E-07	-1.32
TRIM25	2247.400491	-0.472994751	-5.652321103	1.58E-08	4.73E-07	-1.39
B4GALT4	1650.71585	0.44716984	5.649263069	1.61E-08	4.80E-07	1.36
KANK2	4109.287567	-0.469190795	-5.649258933	1.61E-08	4.80E-07	-1.38
OSGIN1	203.6378733	-0.742483487	-5.647122818	1.63E-08	4.85E-07	-1.67
PLA1A	54.06148896	-1.150075428	-5.646626525	1.64E-08	4.86E-07	-2.22
RP11-81A1.3	109.6237541	-0.98935654	-5.645073709	1.65E-08	4.89E-07	-1.99
CLCN5	430.9740991	-0.721199823	-5.6405014	1.70E-08	5.01E-07	-1.65
TBC1D2	998.6011902	-0.488568514	-5.638791582	1.71E-08	5.05E-07	-1.40
DARS2	299.5659852	-0.665413577	-5.638497062	1.72E-08	5.05E-07	-1.59
WARS	5357.391919	-0.405141702	-5.636524566	1.74E-08	5.10E-07	-1.32
ZNF704	158.6924296	-0.821488025	-5.635910461	1.74E-08	5.11E-07	-1.77
NBL1	5233.915554	0.452403375	5.632563148	1.78E-08	5.20E-07	1.37
ADAMTS6	536.814519	0.638671273	5.630863099	1.79E-08	5.25E-07	1.56
CKAP4	22819.64825	0.368417926	5.625012031	1.85E-08	5.42E-07	1.29
GDI1	4254.309502	0.438394041	5.612385955	2.00E-08	5.82E-07	1.36
CRLF1	1885.488277	-0.507495444	-5.608098033	2.05E-08	5.95E-07	-1.42

MFSD3	452.1835125	0.56961015	5.602013702	2.12E-08	6.16E-07	1.48
PLEKHA5	561.7874959	-0.541713232	-5.60138981	2.13E-08	6.17E-07	-1.46
BRSK1	234.9718763	0.73562793	5.600454851	2.14E-08	6.18E-07	1.67
MYLK	7008.899261	-0.575962036	-5.600576632	2.14E-08	6.18E-07	-1.49
ANKRD44	451.8439722	-0.622593607	-5.598975845	2.16E-08	6.22E-07	-1.54
SLC36A1	1031.910995	-0.524639304	-5.59682989	2.18E-08	6.29E-07	-1.44
P4HB	60007.63224	0.339721025	5.596135533	2.19E-08	6.30E-07	1.27
FBXO10	538.8959041	-0.552365364	-5.595073889	2.21E-08	6.32E-07	-1.47
GNS	10726.83105	-0.553369556	-5.595326369	2.20E-08	6.32E-07	-1.47
VPS41	2757.876521	-0.403237799	-5.594363072	2.21E-08	6.33E-07	-1.32
MCM2	287.4074131	-0.674853364	-5.592890519	2.23E-08	6.38E-07	-1.60
RP11-111F16.1	577.296208	-0.539301232	-5.578502971	2.43E-08	6.92E-07	-1.45
OGFRL1	1797.298438	0.465186138	5.577349716	2.44E-08	6.95E-07	1.38
RP11-332P22.2	259.4670882	-0.724106639	-5.573213495	2.50E-08	7.10E-07	-1.65
NT5DC2	5467.232896	0.366378619	5.564222531	2.63E-08	7.46E-07	1.29
LMCD1	900.1440543	0.489422212	5.563574599	2.64E-08	7.47E-07	1.40
ARHGAP29	1669.280274	0.466783718	5.555801256	2.76E-08	7.80E-07	1.38
PPFIBP2	191.6561532	-0.75752753	-5.545991864	2.92E-08	8.23E-07	-1.69
SNX2	1793.419094	-0.432269743	-5.542776841	2.98E-08	8.37E-07	-1.35
HSD11B1	42.49427736	-1.186723214	-5.540639728	3.01E-08	8.46E-07	-2.28
SBSN	99.72660102	-0.951233181	-5.533346274	3.14E-08	8.81E-07	-1.93
AXL	11201.38186	0.374688936	5.527220692	3.25E-08	9.10E-07	1.30
TMEM45A	1540.018673	0.495724261	5.525870982	3.28E-08	9.16E-07	1.41
ELOB	2160.764671	0.551004633	5.523307013	3.33E-08	9.28E-07	1.47
PACSIN3	532.6338009	0.574166264	5.52145272	3.36E-08	9.36E-07	1.49
KIAA1191	3436.488153	0.385004636	5.520433714	3.38E-08	9.40E-07	1.31
SPPL2B	951.1400723	0.470265532	5.512357692	3.54E-08	9.82E-07	1.39
LSAMP	355.0438279	-0.65697778	-5.508058161	3.63E-08	1.01E-06	-1.58
FAM180A	1592.724843	-0.508650981	-5.507236466	3.65E-08	1.01E-06	-1.42
VKORC1	4354.450513	0.47927279	5.505945438	3.67E-08	1.01E-06	1.39
CAV1	18650.77183	0.381942285	5.504477872	3.70E-08	1.02E-06	1.30
PDK1	742.9732481	0.49958049	5.501876859	3.76E-08	1.03E-06	1.41
IGSF8	1569.282951	0.480075741	5.492836029	3.96E-08	1.09E-06	1.39
SH3BGRL3	12688.9865	0.413481772	5.489473505	4.03E-08	1.11E-06	1.33
MANBA	1650.224523	-0.418674936	-5.481545254	4.22E-08	1.15E-06	-1.34
LBP	40.46660419	-1.222500854	-5.478644997	4.29E-08	1.17E-06	-2.33
KRT34	506.5642251	0.573112126	5.477163731	4.32E-08	1.18E-06	1.49
SPG20	1867.246802	-0.407765318	-5.474602793	4.38E-08	1.19E-06	-1.33
RP11-268J15.5	85.05117383	1.047567943	5.47304935	4.42E-08	1.20E-06	2.07
KRT19	509.8496865	0.626056209	5.469806166	4.51E-08	1.22E-06	1.54
TMEM9	1082.548156	0.474460064	5.464006771	4.66E-08	1.26E-06	1.39

RPL37A	8387.641943	0.544604224	5.456508176	4.86E-08	1.31E-06	1.46
FZD8	1081.982599	-0.472939155	-5.454570238	4.91E-08	1.33E-06	-1.39
CRYBB2P1	560.9603795	0.563542169	5.449651973	5.05E-08	1.36E-06	1.48
RDH5	198.941874	0.784285342	5.442436012	5.26E-08	1.42E-06	1.72
PLEKHG4	773.7520177	0.514639688	5.438920635	5.36E-08	1.44E-06	1.43
GYPC	562.0805674	-0.521318004	-5.4296446	5.65E-08	1.52E-06	-1.44
KIAA1715	2382.540139	0.411180256	5.428965551	5.67E-08	1.52E-06	1.33
COX7A1	350.5404802	0.658818238	5.427713423	5.71E-08	1.53E-06	1.58
YIF1B	1668.106288	0.401838858	5.421679743	5.90E-08	1.57E-06	1.32
KDSR	1615.497228	-0.416881236	-5.421667687	5.90E-08	1.57E-06	-1.34
ATOH8	1182.177288	-0.464016015	-5.421534851	5.91E-08	1.57E-06	-1.38
B4GALT1	21497.84114	0.333881915	5.408034766	6.37E-08	1.69E-06	1.26
QSOX1	18743.24198	0.374137943	5.407525819	6.39E-08	1.70E-06	1.30
P3H1	6925.13596	0.355993011	5.400156271	6.66E-08	1.77E-06	1.28
LMAN1	10412.76173	0.392423479	5.399737037	6.67E-08	1.77E-06	1.31
BRI3	2153.142956	-0.464366371	-5.396976422	6.78E-08	1.79E-06	-1.38
NAV3	1374.868642	-0.596775947	-5.396121456	6.81E-08	1.80E-06	-1.51
ARHGEF25	663.6266268	0.508462478	5.393638784	6.90E-08	1.82E-06	1.42
RP11-415I12.6	1278.985187	-0.430148251	-5.393692027	6.90E-08	1.82E-06	-1.35
B4GALNT4	186.0870268	0.769838991	5.391443423	6.99E-08	1.83E-06	1.71
CDKN2B	2914.343522	-0.436228091	-5.391652371	6.98E-08	1.83E-06	-1.35
GEM	262.9778677	-0.659206033	-5.390068531	7.04E-08	1.84E-06	-1.58
CYB5R1	2711.534256	0.415657123	5.380341057	7.43E-08	1.94E-06	1.33
HLA-B	8970.832403	0.431034851	5.37815724	7.53E-08	1.96E-06	1.35
AC098614.2	3650.709971	-0.418257602	-5.378113783	7.53E-08	1.96E-06	-1.34
ACAA2	1627.11616	0.496265939	5.375747148	7.63E-08	1.98E-06	1.41
LAMA2	717.2338122	-0.62926496	-5.350937473	8.75E-08	2.27E-06	-1.55
COL4A2	45775.77269	-0.397326938	-5.350031435	8.79E-08	2.28E-06	-1.32
NFATC4	3074.561949	0.374487365	5.346195835	8.98E-08	2.33E-06	1.30
SLC38A5	918.9585204	0.491969886	5.341569316	9.21E-08	2.38E-06	1.41
ERLEC1	4329.20028	0.374258127	5.337692204	9.41E-08	2.43E-06	1.30
SIRPA	3506.370787	0.412339269	5.332228081	9.70E-08	2.50E-06	1.33
KCNS3	64.24167773	-1.019378864	-5.315433975	1.06E-07	2.74E-06	-2.03
KCND3	143.5076051	-0.816233841	-5.303124649	1.14E-07	2.93E-06	-1.76
DAPK1	849.2475564	-0.49176303	-5.301838898	1.15E-07	2.94E-06	-1.41
ITGA4	324.7606825	-0.70044101	-5.299150862	1.16E-07	2.98E-06	-1.63
EYA4	129.1878973	-0.84343308	-5.295250927	1.19E-07	3.04E-06	-1.79
SCARB1	750.5321833	-0.485856138	-5.28824275	1.23E-07	3.15E-06	-1.40
EMC7	3232.732397	0.497675175	5.287949578	1.24E-07	3.15E-06	1.41
FZD7	2863.353162	0.390954026	5.280979287	1.28E-07	3.27E-06	1.31
PLBD2	8517.059058	-0.395103797	-5.279427686	1.30E-07	3.29E-06	-1.32

PALMD	559.3220707	0.555717236	5.277890563	1.31E-07	3.32E-06	1.47
KLHL24	837.6941061	-0.551667182	-5.275355371	1.32E-07	3.36E-06	-1.47
ZEB2	2517.534949	-0.437748958	-5.274070475	1.33E-07	3.38E-06	-1.35
ECH1	701.7764529	0.487154964	5.270140504	1.36E-07	3.45E-06	1.40
TMED7	3972.288003	0.394315911	5.269656092	1.37E-07	3.45E-06	1.31
VAMP2	1033.812005	0.538859403	5.26857317	1.37E-07	3.46E-06	1.45
PFKL	5550.054742	0.365603793	5.268496682	1.38E-07	3.46E-06	1.29
SLC25A1	2287.625997	0.403686353	5.264795963	1.40E-07	3.52E-06	1.32
TMSL6	1204.310968	-0.546443218	-5.264649829	1.40E-07	3.52E-06	-1.46
PLCD3	879.585392	-0.477046561	-5.263287122	1.42E-07	3.54E-06	-1.39
PRSS12	3756.961474	0.408918176	5.26205595	1.42E-07	3.56E-06	1.33
WDR54	247.5634531	0.666389975	5.261189839	1.43E-07	3.57E-06	1.59
CYTH3	1693.244426	-0.57994377	-5.257045116	1.46E-07	3.65E-06	-1.49
PARP14	440.9847961	-0.620603808	-5.253267523	1.49E-07	3.72E-06	-1.54
SYNGR2	921.3055175	0.461767373	5.248988438	1.53E-07	3.80E-06	1.38
TP53INP2	1186.668403	-0.452746858	-5.248821196	1.53E-07	3.80E-06	-1.37
MGARP	244.9340133	0.713330961	5.242341436	1.59E-07	3.93E-06	1.64
TKT	3517.30681	-0.370321203	-5.234709023	1.65E-07	4.09E-06	-1.29
RPS6KA2	2356.506082	-0.460143895	-5.232487075	1.67E-07	4.13E-06	-1.38
SH2D5	111.1266452	-0.859627794	-5.232525017	1.67E-07	4.13E-06	-1.81
ARL2BP	2759.51088	0.414393114	5.231820358	1.68E-07	4.14E-06	1.33
RP11-383H13.1	543.0362385	-0.636649356	-5.225637905	1.74E-07	4.27E-06	-1.55
IFI35	112.0840159	-0.912902554	-5.223174785	1.76E-07	4.32E-06	-1.88
PRDX4	4968.445409	0.422206497	5.216112965	1.83E-07	4.48E-06	1.34
VCAM1	3172.093974	-0.384968937	-5.215931518	1.83E-07	4.48E-06	-1.31
NBPF19	731.8737389	-0.54451324	-5.206980401	1.92E-07	4.69E-06	-1.46
CEP55	87.56883502	-0.919063174	-5.203643222	1.95E-07	4.77E-06	-1.89
TVP23B	1668.101676	0.442375344	5.202803327	1.96E-07	4.79E-06	1.36
PGRMC1	2549.043194	0.387546551	5.200441579	1.99E-07	4.84E-06	1.31
UTP20	414.435282	-0.668097723	-5.193447857	2.06E-07	5.02E-06	-1.59
MAFB	92.9854852	-0.9131241	-5.193258105	2.07E-07	5.02E-06	-1.88
TSPAN4	3491.330234	0.36056781	5.189983754	2.10E-07	5.10E-06	1.28
DOCK4	531.5748757	-0.607635979	-5.188809174	2.12E-07	5.12E-06	-1.52
NRG1	2424.114815	0.441177162	5.187935055	2.13E-07	5.14E-06	1.36
TNFAIP6	221.2766475	-0.669310562	-5.18187686	2.20E-07	5.30E-06	-1.59
MMP2	105287.787	-0.372081038	-5.176819928	2.26E-07	5.44E-06	-1.29
ARRDC4	1131.570882	0.426082581	5.175318612	2.28E-07	5.48E-06	1.34
ULBP2	541.6606545	0.575894616	5.174188474	2.29E-07	5.49E-06	1.49
TMEM59	11160.45771	0.377413624	5.174371703	2.29E-07	5.49E-06	1.30
RP11-603K19.1	150.744437	-0.769638633	-5.172652956	2.31E-07	5.53E-06	-1.70
LPIN1	1559.957729	-0.511926381	-5.17052818	2.33E-07	5.59E-06	-1.43

SLC25A13	337.3502833	-0.590154219	-5.169393293	2.35E-07	5.61E-06	-1.51
CAPG	904.616423	-0.549376398	-5.165979437	2.39E-07	5.71E-06	-1.46
HSP90B1	47410.8826	0.401038477	5.16042509	2.46E-07	5.87E-06	1.32
NUSAP1	98.81187753	-0.861449527	-5.158905299	2.48E-07	5.91E-06	-1.82
FAM84A	78.10510895	-0.970779508	-5.151365656	2.59E-07	6.14E-06	-1.96
COL1A2	594833.2485	0.355879592	5.148384599	2.63E-07	6.23E-06	1.28
DNAJC13	2376.530908	-0.509543248	-5.142655942	2.71E-07	6.42E-06	-1.42
EGFL7	621.9939211	0.530791533	5.140260277	2.74E-07	6.49E-06	1.44
MOXD1	528.6569253	0.519868447	5.139833611	2.75E-07	6.50E-06	1.43
CAV2	2556.311679	0.397302203	5.137491164	2.78E-07	6.57E-06	1.32
ERAP1	1438.289309	-0.547911231	-5.134399655	2.83E-07	6.67E-06	-1.46
RHOC	7986.867542	0.419139494	5.12208377	3.02E-07	7.11E-06	1.34
TSC1	1233.438262	-0.431681981	-5.121492899	3.03E-07	7.12E-06	-1.35
LGALS1	29435.87006	0.387859509	5.118928582	3.07E-07	7.21E-06	1.31
HBEGF	687.1392824	0.493352999	5.11746062	3.10E-07	7.26E-06	1.41
NID1	3510.915636	-0.499543941	-5.11557969	3.13E-07	7.32E-06	-1.41
CLPTM1L	3470.853755	0.394200679	5.115144631	3.14E-07	7.32E-06	1.31
FDPS	1729.037209	-0.495267768	-5.114399133	3.15E-07	7.34E-06	-1.41
SLC39A13	2536.622833	0.374566361	5.111015199	3.20E-07	7.46E-06	1.30
ITPR2	640.1512117	-0.569705332	-5.111204354	3.20E-07	7.46E-06	-1.48
MELK	175.5870871	-0.750353091	-5.103693965	3.33E-07	7.74E-06	-1.68
GDF6	834.0100879	0.512238863	5.100659701	3.38E-07	7.85E-06	1.43
PSG1	146.0927469	0.786091772	5.094669686	3.49E-07	8.09E-06	1.72
ELFN1	83.73715394	-0.946994853	-5.087326318	3.63E-07	8.40E-06	-1.93
KIF13A	2376.777727	-0.386477324	-5.084712702	3.68E-07	8.51E-06	-1.31
TMEM25	133.5254381	0.801912547	5.082925364	3.72E-07	8.58E-06	1.74
SERINC2	2614.141751	0.37273009	5.076834765	3.84E-07	8.84E-06	1.29
PDLIM2	2467.962341	0.407038061	5.070466238	3.97E-07	9.13E-06	1.33
FKBP8	8131.08745	0.459255124	5.06974251	3.98E-07	9.14E-06	1.37
TP53I11	4200.883667	0.363337337	5.069865987	3.98E-07	9.14E-06	1.29
C1orf122	1094.332914	0.486056901	5.068408172	4.01E-07	9.19E-06	1.40
KRT81	62.87678961	0.991798304	5.060499948	4.18E-07	9.57E-06	1.99
FAM167A	257.6883336	0.78995402	5.05210656	4.37E-07	9.99E-06	1.73
CARHSP1	843.6302868	0.455034712	5.04264305	4.59E-07	1.05E-05	1.37
STXBP5	1856.056469	-0.450878494	-5.04210241	4.60E-07	1.05E-05	-1.37
STEAP3	1533.188082	-0.469648039	-5.035353785	4.77E-07	1.09E-05	-1.38
RHOB	4506.126429	0.334009333	5.034396469	4.79E-07	1.09E-05	1.26
SPG21	1500.543572	-0.394752832	-5.030962375	4.88E-07	1.11E-05	-1.31
OAS3	127.6509866	-0.884702276	-5.030177836	4.90E-07	1.11E-05	-1.85
CD36	568.9059207	-0.501110022	-5.02885069	4.93E-07	1.12E-05	-1.42
GSDMB	149.2663944	0.809092478	5.023981038	5.06E-07	1.14E-05	1.75

TUSC3	5007.818991	0.346159711	5.021773068	5.12E-07	1.16E-05	1.27
GPI	7026.709999	0.324977201	5.021182695	5.14E-07	1.16E-05	1.25
SAP130	587.9168112	-0.506028408	-5.018405529	5.21E-07	1.17E-05	-1.42
STOML1	609.2332795	0.510565437	5.015204571	5.30E-07	1.19E-05	1.42
IFIT1	125.7174734	-0.798324782	-5.013693659	5.34E-07	1.20E-05	-1.74
TMEM38B	284.6752937	-0.588057024	-5.012339819	5.38E-07	1.21E-05	-1.50
PCDHGC3	1329.480747	-0.608249324	-5.012078492	5.38E-07	1.21E-05	-1.52
TSPAN11	191.3713631	-0.694570074	-5.009475206	5.46E-07	1.22E-05	-1.62
ALDOC	651.1352808	0.488487848	5.008101048	5.50E-07	1.23E-05	1.40
TNIK	221.5420205	-0.66342801	-5.007137287	5.52E-07	1.23E-05	-1.58
MCFD2	15904.77208	0.350834008	5.006261896	5.55E-07	1.24E-05	1.28
AC009945.3	562.3691789	-0.51441827	-5.003035293	5.64E-07	1.26E-05	-1.43
GNB2	4235.476821	0.339301212	5.00205151	5.67E-07	1.26E-05	1.27
PON2	3145.005875	0.386606375	4.999623323	5.74E-07	1.27E-05	1.31
FDFT1	2423.499914	-0.391815376	-4.999725911	5.74E-07	1.27E-05	-1.31
RRAS2	1812.007192	0.407316967	4.999022735	5.76E-07	1.27E-05	1.33
VSIR	1827.789555	0.387852327	4.996289409	5.84E-07	1.29E-05	1.31
AKAP12	3953.391463	-0.413670715	-4.993105713	5.94E-07	1.31E-05	-1.33
FEZ2	2022.204653	0.389705244	4.986299516	6.15E-07	1.36E-05	1.31
OLFML3	7970.015049	-0.346655359	-4.985917097	6.17E-07	1.36E-05	-1.27
TP53I13	903.4218925	0.499008911	4.983834882	6.23E-07	1.37E-05	1.41
TBC1D8	157.4589637	-0.724956261	-4.981489298	6.31E-07	1.39E-05	-1.65
TSPO	6475.253903	0.452090409	4.980103044	6.36E-07	1.39E-05	1.37
AMPD3	176.4556509	0.766922342	4.979497639	6.37E-07	1.39E-05	1.70
MAGED1	15391.64039	0.327971015	4.979467837	6.38E-07	1.39E-05	1.26
NOL8	597.9252376	-0.477813388	-4.976575537	6.47E-07	1.41E-05	-1.39
ANK2	1304.974382	-0.542092901	-4.976646865	6.47E-07	1.41E-05	-1.46
RHOQ	3931.102516	-0.343122639	-4.975734676	6.50E-07	1.42E-05	-1.27
EIF3C	1456.778242	-0.51213887	-4.965965938	6.84E-07	1.49E-05	-1.43
PAG1	155.6848736	-0.755405885	-4.962690529	6.95E-07	1.51E-05	-1.69
SMTN	2509.586638	0.390464491	4.962245967	6.97E-07	1.51E-05	1.31
B4GALT5	1529.273553	-0.445308754	-4.962108581	6.97E-07	1.51E-05	-1.36
RP3-523C21.1	134.9778345	0.764518676	4.960163576	7.04E-07	1.52E-05	1.70
MID1IP1	921.0697954	-0.425675315	-4.959458748	7.07E-07	1.53E-05	-1.34
SORBS2	1461.033251	-0.464415181	-4.957724632	7.13E-07	1.54E-05	-1.38
NUPR1	4906.561722	-0.39581581	-4.952160896	7.34E-07	1.58E-05	-1.32
PMM1	803.6316819	0.484241643	4.944255481	7.64E-07	1.64E-05	1.40
RP11-838N2.4	24.26843265	-1.131487803	-4.944290167	7.64E-07	1.64E-05	-2.19
ST6GAL1	148.0161424	-0.746523354	-4.942072718	7.73E-07	1.66E-05	-1.68
SNTB1	965.0720162	-0.423720452	-4.940647248	7.79E-07	1.67E-05	-1.34
KCNJ15	82.67395579	-0.878554263	-4.93916818	7.85E-07	1.68E-05	-1.84

ZNF618	690.2659383	-0.598175671	-4.934767359	8.02E-07	1.72E-05	-1.51
ALDOA	5874.985251	0.352627399	4.930366259	8.21E-07	1.75E-05	1.28
SAMHD1	557.4574939	-0.502877413	-4.92989642	8.23E-07	1.76E-05	-1.42
GOLT1B	2864.184652	0.381707552	4.926671986	8.36E-07	1.78E-05	1.30
HSPA5	42280.19143	0.355466187	4.923327719	8.51E-07	1.81E-05	1.28
JAM2	185.5104933	0.665817997	4.921168834	8.60E-07	1.83E-05	1.59
OCIAD2	357.2553994	0.608408183	4.919739849	8.67E-07	1.84E-05	1.52
SLF2	756.1631319	-0.461524033	-4.918796126	8.71E-07	1.84E-05	-1.38
SSR4	4335.753312	0.418676065	4.915674441	8.85E-07	1.87E-05	1.34
SCARF2	2686.652893	0.402433204	4.915578581	8.85E-07	1.87E-05	1.32
GINM1	2207.662124	0.354315388	4.91372531	8.94E-07	1.88E-05	1.28
FAM129B	17591.02555	0.346568671	4.913269089	8.96E-07	1.88E-05	1.27
KCTD9	1156.687469	0.41769867	4.911089336	9.06E-07	1.90E-05	1.34
DSTNP2	409.6394745	-0.528287603	-4.902736608	9.45E-07	1.98E-05	-1.44
VPS18	1114.78287	-0.401119166	-4.898439895	9.66E-07	2.02E-05	-1.32
RGS5	112.659186	0.805720953	4.896157589	9.77E-07	2.05E-05	1.75
GSR	1134.144714	-0.413145148	-4.895404395	9.81E-07	2.05E-05	-1.33
COX7A2	1340.526142	0.480230808	4.893107711	9.93E-07	2.07E-05	1.39
GPT2	1248.017342	0.401820536	4.890828546	1.00E-06	2.09E-05	1.32
ETV1	334.4132589	-0.563775248	-4.887865272	1.02E-06	2.12E-05	-1.48
SSR3	9764.401782	0.337577207	4.887364798	1.02E-06	2.13E-05	1.26
C16orf62	1569.346337	-0.378349364	-4.881121409	1.05E-06	2.19E-05	-1.30
PLIN3	5968.775668	0.323797893	4.880300447	1.06E-06	2.20E-05	1.25
KCNK6	1545.221215	0.40326824	4.876682716	1.08E-06	2.24E-05	1.32
PXK	943.8147568	-0.421564535	-4.874703984	1.09E-06	2.26E-05	-1.34
CLDN1	304.2895011	-0.583606542	-4.871893832	1.11E-06	2.29E-05	-1.50
PIDD1	296.7373525	0.584549973	4.870973745	1.11E-06	2.29E-05	1.50
ARL8B	2490.856126	-0.350486844	-4.87052685	1.11E-06	2.30E-05	-1.27
HSPH1	2587.654633	-0.349263536	-4.868692474	1.12E-06	2.31E-05	-1.27
EPDR1	2328.170604	-0.345976661	-4.866896494	1.13E-06	2.33E-05	-1.27
CD109	3182.343909	-0.508941791	-4.863139387	1.16E-06	2.37E-05	-1.42
TMEM126B	567.2594954	-0.503601929	-4.861885142	1.16E-06	2.38E-05	-1.42
TRANK1	283.9762829	-0.626405375	-4.861820128	1.16E-06	2.38E-05	-1.54
PPP1R15A	2318.549344	-0.405504929	-4.858936308	1.18E-06	2.42E-05	-1.32
AC073869.22	146.5029569	-0.754291204	-4.85732756	1.19E-06	2.43E-05	-1.69
SPECC1	911.3225141	-0.426038665	-4.851635039	1.22E-06	2.50E-05	-1.34
QPCTL	526.0749249	0.477910618	4.84928741	1.24E-06	2.52E-05	1.39
MKNK2	1415.280891	-0.414722648	-4.849032146	1.24E-06	2.52E-05	-1.33
SPOCK1	18908.63235	-0.419969302	-4.848610545	1.24E-06	2.53E-05	-1.34
SPIRE1	1321.407119	-0.447929928	-4.845206889	1.26E-06	2.57E-05	-1.36
ASAP3	879.0250429	-0.45495059	-4.84193121	1.29E-06	2.61E-05	-1.37

UBAP2	665.7718949	-0.496815773	-4.841773485	1.29E-06	2.61E-05	-1.41
GAS2L3	36.73107118	-1.049660837	-4.839627158	1.30E-06	2.63E-05	-2.07
RP11-1033A18.1	3030.086605	-0.364209525	-4.837179198	1.32E-06	2.66E-05	-1.29
ANKRD13A	2047.395107	0.416084076	4.829296301	1.37E-06	2.76E-05	1.33
GMPPA	1260.715558	0.413434315	4.829101541	1.37E-06	2.76E-05	1.33
WSB1	2687.259575	0.40126529	4.827054119	1.39E-06	2.79E-05	1.32
STX1A	700.1464758	0.444891878	4.825767702	1.39E-06	2.80E-05	1.36
C3orf58	273.7898101	0.575309908	4.825352967	1.40E-06	2.81E-05	1.49
AC016722.2	103.1133618	0.827861437	4.819217212	1.44E-06	2.89E-05	1.78
VEGFC	3589.119046	0.359536573	4.818978688	1.44E-06	2.89E-05	1.28
KRT16	188.0655483	0.714252869	4.818463898	1.45E-06	2.89E-05	1.64
CD81	12817.11193	0.39776433	4.813938144	1.48E-06	2.96E-05	1.32
PPME1	2020.549496	0.391593589	4.81314051	1.49E-06	2.97E-05	1.31
PCBP4	1528.819494	0.367183896	4.808260541	1.52E-06	3.03E-05	1.29
BUB1B	80.16309213	-0.860555195	-4.80542864	1.54E-06	3.07E-05	-1.82
BCL10	706.6053773	0.475263886	4.803602695	1.56E-06	3.10E-05	1.39
VLDLR	1013.430715	0.418305033	4.802055487	1.57E-06	3.12E-05	1.34
SFXN1	1255.633416	0.384752958	4.802275286	1.57E-06	3.12E-05	1.31
CDCA8	76.60854358	-0.871553506	-4.795141049	1.63E-06	3.22E-05	-1.83
GLUL	1162.979521	-0.388237434	-4.788233057	1.68E-06	3.32E-05	-1.31
AIM1	178.2047089	-0.767257287	-4.788091169	1.68E-06	3.32E-05	-1.70
TRAF4	872.2263016	0.437283731	4.787468654	1.69E-06	3.33E-05	1.35
NECTIN2	2191.140323	0.348310521	4.784195431	1.72E-06	3.38E-05	1.27
CSPG4	1157.511207	-0.510561579	-4.784187902	1.72E-06	3.38E-05	-1.42
DAGLB	686.6863711	0.444401484	4.780263553	1.75E-06	3.44E-05	1.36
PLOD1	29959.08124	0.338173251	4.779408955	1.76E-06	3.45E-05	1.26
ANXA10	87.78555392	0.868157644	4.775131422	1.80E-06	3.52E-05	1.83
KCTD11	1362.815585	0.38998621	4.772839948	1.82E-06	3.55E-05	1.31
MDN1	697.9401379	-0.590741271	-4.772543739	1.82E-06	3.55E-05	-1.51
AK1	1053.627726	0.407640757	4.770110414	1.84E-06	3.59E-05	1.33
SEMA5A	1907.035863	-0.601811291	-4.76700673	1.87E-06	3.64E-05	-1.52
TPP2	1070.17363	-0.420019958	-4.76266051	1.91E-06	3.71E-05	-1.34
SIL1	3543.880053	0.430143385	4.761650392	1.92E-06	3.72E-05	1.35
RELT	205.6098507	0.657296258	4.759686253	1.94E-06	3.75E-05	1.58
RARRES1	60.46460141	0.943630141	4.757647311	1.96E-06	3.78E-05	1.92
UBL5	1579.162994	0.50502514	4.757686608	1.96E-06	3.78E-05	1.42
MPDZ	1512.392378	-0.512655707	-4.757720023	1.96E-06	3.78E-05	-1.43
EGFR	2768.163738	-0.532726855	-4.757206825	1.96E-06	3.78E-05	-1.45
KIDINS220	2835.39393	-0.441301217	-4.754090201	1.99E-06	3.84E-05	-1.36
PPP1R16A	591.255131	0.476824879	4.751119066	2.02E-06	3.88E-05	1.39
PARP9	244.1603215	-0.599701665	-4.748027849	2.05E-06	3.94E-05	-1.52

ADGRB2	620.1412101	0.465324542	4.746955992	2.07E-06	3.95E-05	1.38
ADARB1	2485.032067	0.379828689	4.746848058	2.07E-06	3.95E-05	1.30
BCAP31	4965.064911	0.409644652	4.7444525	2.09E-06	4.00E-05	1.33
SYT11	2798.191476	0.351810682	4.742561206	2.11E-06	4.03E-05	1.28
NPAS1	726.499989	0.468908088	4.741906789	2.12E-06	4.04E-05	1.38
SETBP1	174.0042451	-0.822303173	-4.741790928	2.12E-06	4.04E-05	-1.77
CEBPB	997.6438599	-0.532166565	-4.739185544	2.15E-06	4.08E-05	-1.45
RPS19P1	1770.867033	0.448749058	4.737247999	2.17E-06	4.12E-05	1.36
GALNT5	13300.77638	0.401013994	4.73605501	2.18E-06	4.14E-05	1.32
DLGAP5	74.33596011	-0.872642734	-4.734224802	2.20E-06	4.17E-05	-1.83
COMT	4296.4778	0.348081451	4.732894011	2.21E-06	4.19E-05	1.27
NSMAF	1173.468198	-0.395471815	-4.732310703	2.22E-06	4.20E-05	-1.32
TSPAN10	281.5944127	-0.577080824	-4.724628786	2.31E-06	4.35E-05	-1.49
KDELR3	5688.010432	0.323532308	4.723629373	2.32E-06	4.37E-05	1.25
RNF215	619.1600325	0.455085089	4.72093623	2.35E-06	4.42E-05	1.37
ZNF185	162.8369061	0.699728247	4.720221305	2.36E-06	4.43E-05	1.62
OSTC	4909.659187	0.393306454	4.720433036	2.35E-06	4.43E-05	1.31
TMEM192	657.6261262	-0.466035307	-4.718100658	2.38E-06	4.47E-05	-1.38
FAM13B	1230.170581	-0.444510957	-4.71750612	2.39E-06	4.48E-05	-1.36
SMYD3	542.9150954	-0.484701881	-4.715460501	2.41E-06	4.51E-05	-1.40
AKAP13	1118.842067	-0.493110425	-4.714855197	2.42E-06	4.52E-05	-1.41
PORCN	826.0441805	0.45328798	4.713772363	2.43E-06	4.54E-05	1.37
CYTIP	47.8677896	0.981336115	4.707446244	2.51E-06	4.67E-05	1.97
UPP1	175.0543133	-0.685408416	-4.705194333	2.54E-06	4.72E-05	-1.61
SLC39A7	9345.970553	0.344658077	4.704360264	2.55E-06	4.73E-05	1.27
CYP51A1	1555.717799	-0.373324218	-4.703923876	2.55E-06	4.74E-05	-1.30
PSME2	654.3262886	0.534332711	4.703539393	2.56E-06	4.74E-05	1.45
C16orf13	780.6561589	0.45816873	4.702011863	2.58E-06	4.77E-05	1.37
DIP2B	1011.816311	-0.56274374	-4.699572179	2.61E-06	4.82E-05	-1.48
FCGRT	713.8249749	0.440684079	4.695903807	2.65E-06	4.90E-05	1.36
ZFAND2A	371.2154318	0.596946115	4.692405986	2.70E-06	4.98E-05	1.51
RABEP1	2119.318108	-0.362084582	-4.69147321	2.71E-06	5.00E-05	-1.29
CLCN6	454.1738424	-0.506495831	-4.689918123	2.73E-06	5.03E-05	-1.42
RASAL2	1206.118742	-0.533964101	-4.688927778	2.75E-06	5.05E-05	-1.45
CTB-52I2.4	407.2369135	-0.588344252	-4.687102699	2.77E-06	5.09E-05	-1.50
RP11-327L3.5	1503.14936	-0.392204248	-4.684685768	2.80E-06	5.15E-05	-1.31
NDUFA13	792.5372313	0.58447986	4.683994388	2.81E-06	5.16E-05	1.50
MMRN2	60.48806866	0.909639101	4.679367829	2.88E-06	5.27E-05	1.88
IL1R1	2129.028149	-0.429553146	-4.677959679	2.90E-06	5.30E-05	-1.35
COL5A3	391.8012459	-0.511413407	-4.677749928	2.90E-06	5.30E-05	-1.43
COL4A1	46580.97978	-0.400108066	-4.673835892	2.96E-06	5.39E-05	-1.32

RP11-488C13.1	640.8643152	-0.499391973	-4.672762002	2.97E-06	5.41E-05	-1.41
RP1-239B22.5	48.45204353	-0.956263857	-4.672959681	2.97E-06	5.41E-05	-1.94
SOX4	1706.420047	-0.398996887	-4.672433739	2.98E-06	5.41E-05	-1.32
CIRBP	3026.336464	0.39450738	4.672203137	2.98E-06	5.41E-05	1.31
RUSC1	525.4270563	0.462187139	4.671779698	2.99E-06	5.42E-05	1.38
BOD1	1049.818659	-0.440620148	-4.669921018	3.01E-06	5.46E-05	-1.36
PRKDC	3046.423025	-0.50112267	-4.66929295	3.02E-06	5.47E-05	-1.42
RP11-612L3.1	1127.514618	-0.385509042	-4.66537279	3.08E-06	5.57E-05	-1.31
MYCT1	100.0753462	0.802899082	4.659979237	3.16E-06	5.71E-05	1.74
FAM111A	406.931279	-0.495215535	-4.658192206	3.19E-06	5.75E-05	-1.41
PTOV1	1885.389849	0.376426702	4.656231021	3.22E-06	5.80E-05	1.30
ITGA10	1039.181179	0.453229234	4.654436197	3.25E-06	5.84E-05	1.37
ATP2B1	2958.229102	-0.349542887	-4.654065184	3.25E-06	5.85E-05	-1.27
MZT2B	1499.871339	0.409076925	4.652880118	3.27E-06	5.87E-05	1.33
FAM102A	1035.026845	-0.378493658	-4.646657252	3.37E-06	6.05E-05	-1.30
CERS5	1324.910551	0.38665616	4.646233903	3.38E-06	6.05E-05	1.31
MAP3K5	377.9661254	-0.49861092	-4.641303383	3.46E-06	6.19E-05	-1.41
RP11-354P17.9	293.1227861	0.600435308	4.635854287	3.55E-06	6.35E-05	1.52
ABL2	1262.780596	-0.511659309	-4.631773542	3.63E-06	6.47E-05	-1.43
RGS3	2162.264614	0.338760979	4.631454479	3.63E-06	6.47E-05	1.26
PDIA6	17610.98104	0.358217048	4.630580287	3.65E-06	6.49E-05	1.28
IL21R	302.1109959	-0.544869059	-4.630632451	3.65E-06	6.49E-05	-1.46
FTH1P7	2591.68032	-0.435376875	-4.628762872	3.68E-06	6.54E-05	-1.35
KIAA2013	1904.490162	0.362189065	4.625261403	3.74E-06	6.63E-05	1.29
LACTB	1333.190025	-0.378822914	-4.625319699	3.74E-06	6.63E-05	-1.30
SUGCT	827.9092288	-0.474107791	-4.625094864	3.74E-06	6.63E-05	-1.39
SLC9A3R2	1308.69928	0.433176019	4.623582727	3.77E-06	6.67E-05	1.35
RRAS	2751.867853	0.355617072	4.62098731	3.82E-06	6.75E-05	1.28
ARSJ	2119.121098	0.406614598	4.620734502	3.82E-06	6.75E-05	1.33
SLC38A1	2701.456773	-0.516614642	-4.619225001	3.85E-06	6.79E-05	-1.43
STARD5	146.6294493	-0.713462121	-4.618099796	3.87E-06	6.82E-05	-1.64
CREB3	1747.626447	0.356269838	4.616373572	3.91E-06	6.87E-05	1.28
SLC16A6	36.58586115	-1.024346456	-4.61503742	3.93E-06	6.91E-05	-2.03
AC004057.1	1126.476996	0.488631453	4.612550085	3.98E-06	6.99E-05	1.40
DDR1	1911.10692	0.345441704	4.609815602	4.03E-06	7.07E-05	1.27
HNMT	273.3196412	-0.584277795	-4.605001717	4.12E-06	7.22E-05	-1.50
SRFBP1	171.9147328	-0.662671547	-4.605044247	4.12E-06	7.22E-05	-1.58
PTHLH	38.71596766	0.999824603	4.602795914	4.17E-06	7.29E-05	2.00
TMEM60	244.7323242	-0.580533786	-4.602710608	4.17E-06	7.29E-05	-1.50
NEK6	2786.391634	-0.426979619	-4.602095789	4.18E-06	7.30E-05	-1.34
ECM1	1203.484041	-0.388240722	-4.600225119	4.22E-06	7.36E-05	-1.31

KANSL1L	134.9667992	-0.728080659	-4.59342618	4.36E-06	7.59E-05	-1.66
SLITRK6	99.3242036	0.799303308	4.59156236	4.40E-06	7.64E-05	1.74
MXI1	1090.520717	0.370032397	4.591306269	4.40E-06	7.64E-05	1.29
TLR3	129.8902791	-0.710859241	-4.59057029	4.42E-06	7.66E-05	-1.64
PAMR1	2154.799223	0.351181774	4.589962535	4.43E-06	7.67E-05	1.28
AC018804.5	125.9495285	-0.73741861	-4.588659084	4.46E-06	7.71E-05	-1.67
CTNNAL1	2195.930949	0.366315375	4.580074329	4.65E-06	8.02E-05	1.29
SRM	2696.32693	0.340304097	4.579702705	4.66E-06	8.03E-05	1.27
ALS2	1099.060788	-0.404320018	-4.57796133	4.70E-06	8.09E-05	-1.32
CABP1	29.55718201	1.024552894	4.573519348	4.80E-06	8.25E-05	2.03
PPP2R5B	932.6963214	0.391610801	4.570694043	4.86E-06	8.35E-05	1.31
C20orf82	72.48194423	-0.860492411	-4.568930325	4.90E-06	8.42E-05	-1.82
OSBPL3	809.9650667	-0.433079494	-4.566210704	4.97E-06	8.52E-05	-1.35
TXNDC17	1009.457307	0.469808772	4.565689324	4.98E-06	8.53E-05	1.38
FAM89B	1402.625015	0.381667197	4.564484232	5.01E-06	8.56E-05	1.30
RP11-473N11.2	367.4372998	-0.502244645	-4.564434515	5.01E-06	8.56E-05	-1.42
RP11-644F5.10	198.9514871	0.60652457	4.56076572	5.10E-06	8.70E-05	1.52
SPTLC3	188.7629173	-0.620271548	-4.56092507	5.09E-06	8.70E-05	-1.54
PHF1	1445.67132	0.358300048	4.560115401	5.11E-06	8.71E-05	1.28
DCBLD2	21003.47311	0.356202487	4.557721169	5.17E-06	8.80E-05	1.28
ZNHIT1	1554.205164	0.456525896	4.557296343	5.18E-06	8.81E-05	1.37
MAPK8IP3	1222.525925	0.366502879	4.556865141	5.19E-06	8.81E-05	1.29
EEA1	1659.283697	-0.363706057	-4.556401209	5.20E-06	8.83E-05	-1.29
MLF2	5185.250456	0.341379802	4.554076508	5.26E-06	8.91E-05	1.27
CREBRF	573.4628534	-0.508039128	-4.553716861	5.27E-06	8.92E-05	-1.42
IGF2BP3	143.2918758	0.679441166	4.553035304	5.29E-06	8.94E-05	1.60
UCHL1	5194.672975	0.449948707	4.547964596	5.42E-06	9.15E-05	1.37
GIGYF2	1566.618506	-0.359537067	-4.547737424	5.42E-06	9.15E-05	-1.28
VDR	1716.657866	-0.446888091	-4.546234704	5.46E-06	9.21E-05	-1.36
KIAA0586	324.0053908	-0.538894564	-4.532788926	5.82E-06	9.80E-05	-1.45
SOAT1	1210.740616	-0.362830213	-4.530775024	5.88E-06	9.89E-05	-1.29
AK3	2312.51549	0.323615583	4.53041959	5.89E-06	9.89E-05	1.25
MIF	3736.012636	0.386181873	4.528845356	5.93E-06	9.96E-05	1.31
ACSF2	888.4136808	-0.440191627	-4.527902083	5.96E-06	9.99E-05	-1.36
CYP4V2	280.3298181	-0.608128618	-4.524677017	6.05E-06	0.00010125	-1.52
RER1	4273.539766	0.328937231	4.52339809	6.09E-06	0.00010164	1.26
NOV	81.60586541	-0.810481276	-4.523364308	6.09E-06	0.00010164	-1.75
AHNAK2	897.2396273	-0.878844372	-4.522674881	6.11E-06	0.0001018	-1.84
STX1B	75.39924494	0.833875508	4.522392652	6.11E-06	0.00010184	1.78
GABRE	333.3470289	0.591107518	4.521427061	6.14E-06	0.0001021	1.51
CGREF1	1067.675881	0.396237808	4.52026793	6.18E-06	0.0001025	1.32

FRRS1	125.2963503	-0.746344328	-4.520184571	6.18E-06	0.0001025	-1.68
DOCK11	243.5386184	-0.589524389	-4.518466878	6.23E-06	0.00010323	-1.50
PTPRG	3235.867414	-0.439579995	-4.518133213	6.24E-06	0.00010329	-1.36
SARAF	7975.823175	0.326140694	4.51684306	6.28E-06	0.00010382	1.25
ECHS1	1933.776116	0.375531342	4.505088327	6.63E-06	0.00010952	1.30
SWAP70	1676.620083	-0.355825702	-4.504387436	6.66E-06	0.00010977	-1.28
ANKRD28	1104.166742	-0.38266717	-4.501750663	6.74E-06	0.00011103	-1.30
ADCY4	557.6280481	0.44188833	4.498837492	6.83E-06	0.00011245	1.36
DSCR3	1467.026232	-0.374933538	-4.495777793	6.93E-06	0.00011397	-1.30
PODNL1	634.8251664	-0.468211786	-4.492118663	7.05E-06	0.00011583	-1.38
PLEKHM1	1063.118038	-0.463280936	-4.489030983	7.15E-06	0.00011729	-1.38
NCAM2	81.9773702	-0.808366792	-4.48833165	7.18E-06	0.00011756	-1.75
ADPGK	1476.874487	0.349979127	4.487481442	7.21E-06	0.00011784	1.27
B4GALT2	3103.385417	0.34374975	4.487407238	7.21E-06	0.00011784	1.27
YIF1A	2461.888231	0.370160857	4.486443956	7.24E-06	0.00011826	1.29
MIR22HG	1456.877056	0.370249807	4.486020114	7.26E-06	0.00011838	1.29
TMEM208	703.5306117	0.46845103	4.484216218	7.32E-06	0.00011927	1.38
CAD	1039.225338	-0.470654429	-4.483465169	7.34E-06	0.00011957	-1.39
EPOP	141.1141051	0.676597231	4.481280461	7.42E-06	0.00012069	1.60
RPL39	409.5425755	0.625220933	4.480914763	7.43E-06	0.00012078	1.54
CDKN2A	1241.281105	-0.405370172	-4.479365312	7.49E-06	0.00012154	-1.32
FAM171A1	1772.237399	0.336946257	4.476590708	7.58E-06	0.00012301	1.26
AC011816.4	78.27022789	0.813174389	4.471091228	7.78E-06	0.00012597	1.76
MAPK7	791.6391547	0.396191252	4.466294075	7.96E-06	0.00012858	1.32
ECE1	7350.240051	0.327351837	4.464501788	8.03E-06	0.00012953	1.25
CD3EAP	268.2456331	-0.558959099	-4.464234456	8.04E-06	0.00012957	-1.47
TAF13	1263.671226	0.370774639	4.463548993	8.06E-06	0.00012986	1.29
SVIL	2048.050955	-0.41757826	-4.461611666	8.13E-06	0.00013091	-1.34
DUSP3	2473.082785	-0.32205455	-4.457174727	8.30E-06	0.00013352	-1.25
GPX4	2996.076461	-0.390006609	-4.45176201	8.52E-06	0.0001368	-1.31
SLC22A3	367.0216625	-0.524551354	-4.450558567	8.56E-06	0.00013744	-1.44
PFKM	1501.470103	-0.349785363	-4.44964818	8.60E-06	0.00013789	-1.27
PCM1	1934.520727	-0.326488399	-4.445014875	8.79E-06	0.00014065	-1.25
BACE2	2282.719158	-0.339532666	-4.444979354	8.79E-06	0.00014065	-1.27
KIAA1671	1340.835289	-0.504155542	-4.441155356	8.95E-06	0.00014304	-1.42
AGPAT2	667.811962	0.461232049	4.43877539	9.05E-06	0.00014449	1.38
TMEM167B	1573.473207	0.349827468	4.437318975	9.11E-06	0.00014519	1.27
MVD	854.8770961	-0.424372775	-4.436925973	9.13E-06	0.00014532	-1.34
POLR2L	4063.594052	0.417226907	4.436061974	9.16E-06	0.00014562	1.34
RP3-412A9.11	3083.381077	0.405180899	4.436077231	9.16E-06	0.00014562	1.32
DDB2	667.3826641	0.407904269	4.433847203	9.26E-06	0.00014685	1.33

PANK3	1586.165405	-0.380762678	-4.433969176	9.25E-06	0.00014685	-1.30
DUSP10	376.1099643	-0.479632325	-4.432459531	9.32E-06	0.00014766	-1.39
TSPAN6	887.7235976	0.391961979	4.430190868	9.41E-06	0.00014908	1.31
WDR83OS	1709.146693	0.359229155	4.426520313	9.58E-06	0.00015149	1.28
GFPT2	182.285397	0.649586513	4.42450232	9.67E-06	0.00015277	1.57
FAM234A	2608.615998	0.336436033	4.420360088	9.85E-06	0.00015543	1.26
PARP4	2000.159081	-0.394116273	-4.420369542	9.85E-06	0.00015543	-1.31
TMEM106A	325.1025716	0.505326353	4.41287667	1.02E-05	0.00016076	1.42
SETDB2	232.2883158	-0.570279866	-4.410543504	1.03E-05	0.00016234	-1.48
CNTLN	223.8015358	-0.559429409	-4.402361072	1.07E-05	0.00016827	-1.47
RP11-77G22.3	280.9908223	-0.551364959	-4.401206029	1.08E-05	0.00016886	-1.47
SREBF2	3680.449631	-0.374009122	-4.396866183	1.10E-05	0.00017194	-1.30
GPX7	474.9510797	0.450593034	4.39634782	1.10E-05	0.00017203	1.37
MROH1	829.3840244	-0.404754667	-4.39649907	1.10E-05	0.00017203	-1.32
POLR2C	1566.114458	0.344724205	4.394418432	1.11E-05	0.0001734	1.27
IFIH1	43.26452021	-0.936554153	-4.391676773	1.12E-05	0.00017536	-1.91
AP001422.3	30.05891603	-0.974449762	-4.391575029	1.13E-05	0.00017536	-1.96
CTC-575D19.1	1464.275037	0.408071189	4.388939898	1.14E-05	0.00017693	1.33
CCDC93	1316.469653	-0.451342523	-4.388822284	1.14E-05	0.00017693	-1.37
TMEM9B	1278.563204	0.344043473	4.388117139	1.14E-05	0.00017734	1.27
TOR4A	302.5152421	0.530677111	4.386559802	1.15E-05	0.00017845	1.44
CTD-2033D24.2	86.33108245	-0.762534302	-4.380771763	1.18E-05	0.00018309	-1.70
GSTP1	4902.650694	0.358587651	4.371198358	1.24E-05	0.00019102	1.28
C5orf51	1057.871879	-0.459816536	-4.371114209	1.24E-05	0.00019102	-1.38
IKBIP	2859.954047	0.362151266	4.368582801	1.25E-05	0.00019307	1.29
TMEM158	561.7897014	0.469993112	4.368251778	1.25E-05	0.00019319	1.39
ADH5P4	491.636614	-0.445424468	-4.366373371	1.26E-05	0.00019468	-1.36
IRS1	1390.764527	-0.45185912	-4.363682421	1.28E-05	0.00019691	-1.37
MYPN	718.1416619	0.462450453	4.361748094	1.29E-05	0.00019847	1.38
PIAS3	1567.744973	0.333641031	4.358729969	1.31E-05	0.00020104	1.26
PARD3B	490.7012474	-0.519829823	-4.358480053	1.31E-05	0.00020109	-1.43
TTC7A	987.8187657	0.364826217	4.358257856	1.31E-05	0.00020111	1.29
NUDT14	133.7236322	-0.665745024	-4.35404383	1.34E-05	0.00020464	-1.59
FOXF2	310.3718985	-0.553942729	-4.351540088	1.35E-05	0.00020661	-1.47
HRCT1	74.0591102	0.815453344	4.350225124	1.36E-05	0.00020766	1.76
SLC52A2	1588.453422	0.346863285	4.347328462	1.38E-05	0.00021023	1.27
DIEXF	797.8378359	-0.379505101	-4.344573654	1.40E-05	0.0002125	-1.30
RTKN2	215.2086108	-0.619999402	-4.342581765	1.41E-05	0.00021424	-1.54
ANKZF1	516.5457696	0.442683574	4.342380574	1.41E-05	0.00021424	1.36
PTGER2	154.0112181	0.645927061	4.340331981	1.42E-05	0.00021586	1.56
ESM1	1259.923978	0.396275628	4.339058226	1.43E-05	0.00021691	1.32

SPATA13	129.2933647	-0.680224545	-4.338336442	1.44E-05	0.00021743	-1.60
UBE4B	1690.481129	-0.366741664	-4.3354191	1.45E-05	0.00022013	-1.29
MMAB	328.2059923	-0.509755271	-4.33506801	1.46E-05	0.00022029	-1.42
ZFYVE27	652.0512211	0.405097627	4.326791215	1.51E-05	0.00022811	1.32
ITPR1	508.2524825	-0.584209989	-4.323201267	1.54E-05	0.00023143	-1.50
DAB2IP	826.8158914	-0.375312193	-4.322922348	1.54E-05	0.00023152	-1.30
ITGA1	970.1458077	-0.393411638	-4.318391082	1.57E-05	0.0002359	-1.31
CIB1	1819.429287	0.430618595	4.316155726	1.59E-05	0.00023808	1.35
RPLP0P2	91.25295408	-0.771143423	-4.315834024	1.59E-05	0.00023822	-1.71
GNA13	1424.500747	-0.371968055	-4.314670929	1.60E-05	0.00023926	-1.29
LMNB1	95.35089281	-0.754526806	-4.314387023	1.60E-05	0.00023935	-1.69
DISC1	90.54223792	-0.773540777	-4.313498613	1.61E-05	0.0002401	-1.71
HILPDA	213.9153477	0.576114695	4.311228449	1.62E-05	0.00024236	1.49
LIPA	2001.60977	-0.336959924	-4.309368868	1.64E-05	0.00024419	-1.26
MANF	2544.164068	0.404033525	4.308645778	1.64E-05	0.00024477	1.32
DMPK	1994.378137	0.3579065	4.30561378	1.67E-05	0.00024793	1.28
KCTD5	1086.861919	0.354153126	4.304216363	1.68E-05	0.00024928	1.28
GPX1	2296.744389	-0.409683765	-4.302565973	1.69E-05	0.00025092	-1.33
PDE3A	200.7550273	-0.630246594	-4.301823208	1.69E-05	0.00025154	-1.55
PRSS53	241.2009917	0.583454817	4.300233178	1.71E-05	0.00025312	1.50
BTN3A1	424.8143422	-0.449533017	-4.299824859	1.71E-05	0.00025337	-1.37
FAM174A	555.1990979	0.476189033	4.297071968	1.73E-05	0.00025608	1.39
ATR	666.8729829	-0.418291251	-4.297114024	1.73E-05	0.00025608	-1.34
CDC42EP2	530.4889972	0.433782645	4.296418575	1.74E-05	0.00025661	1.35
APBB1	1423.479604	0.334378107	4.295683273	1.74E-05	0.00025723	1.26
PLK1	84.65937967	-0.754829597	-4.290893795	1.78E-05	0.00026261	-1.69
RP1-89D4.1	887.7957707	-0.419440859	-4.290354906	1.78E-05	0.00026302	-1.34
RP11-598F17.1	1571.37975	-0.337613344	-4.28985487	1.79E-05	0.00026338	-1.26
CSNK1E	1227.125728	0.373067834	4.289400899	1.79E-05	0.00026368	1.30
ATPIF1	1438.793169	0.393412325	4.287741764	1.80E-05	0.00026543	1.31
MXRA5	986.5618869	-0.896899114	-4.285339187	1.82E-05	0.00026807	-1.86
SLC22A15	175.3323359	-0.606747561	-4.284981748	1.83E-05	0.00026827	-1.52
PBX1	309.7988377	-0.521210911	-4.28003802	1.87E-05	0.00027406	-1.44
SEPW1	2885.411359	0.409321129	4.278896691	1.88E-05	0.00027523	1.33
ARHGEF2	3154.193228	-0.325798441	-4.277901935	1.89E-05	0.0002761	-1.25
CSDAP1	357.8628775	-0.478224536	-4.277796964	1.89E-05	0.0002761	-1.39
NR2F2	1724.02732	-0.335577638	-4.276638385	1.90E-05	0.0002773	-1.26
RP11-288G3.4	115.3075315	-0.692783921	-4.2757586	1.90E-05	0.00027816	-1.62
NME3	372.9082811	0.462367633	4.275281151	1.91E-05	0.00027851	1.38
RP11-572P18.1	711.9563933	0.540852504	4.273814994	1.92E-05	0.00027997	1.45
CHCHD9	414.8753665	0.496913158	4.272596258	1.93E-05	0.00028115	1.41

NCKAP5	309.9303199	0.558130238	4.271933209	1.94E-05	0.00028174	1.47
ROS1	104.9462178	0.754135558	4.263620007	2.01E-05	0.00029218	1.69
CORO2B	774.6365787	0.4209551	4.260999088	2.04E-05	0.00029511	1.34
KAZN	446.5364979	-0.477518037	-4.257168325	2.07E-05	0.00029995	-1.39
APOL1	1219.256011	-0.363945134	-4.255712895	2.08E-05	0.00030165	-1.29
IFIT2	105.3092111	-0.70729983	-4.254463953	2.10E-05	0.00030308	-1.63
KRT8	80.18900443	0.767759398	4.253086183	2.11E-05	0.00030442	1.70
RP11-700P18.1	726.4062273	-0.404017061	-4.253152168	2.11E-05	0.00030442	-1.32
HEATR1	906.7450404	-0.447504822	-4.251717342	2.12E-05	0.00030576	-1.36
TCF7	440.8796911	-0.484236636	-4.251770215	2.12E-05	0.00030576	-1.40
GUK1	3936.844443	0.372664454	4.251098817	2.13E-05	0.00030634	1.29
RUSC2	2836.908127	0.323411373	4.240229975	2.23E-05	0.000321	1.25
AC073115.7	73.06701157	0.811740423	4.238012627	2.26E-05	0.00032391	1.76
BAALC	310.6829848	-0.505266159	-4.23695082	2.27E-05	0.00032517	-1.42
CREG1	2213.164649	-0.348074084	-4.230741769	2.33E-05	0.00033313	-1.27
YIPF2	1880.006562	0.359751794	4.221966554	2.42E-05	0.00034518	1.28
ICE1	1201.123592	-0.410726231	-4.221780903	2.42E-05	0.00034518	-1.33
FOXN3	1202.847415	-0.436044091	-4.22035654	2.44E-05	0.00034707	-1.35
NPR3	6802.426624	0.360869945	4.217846592	2.47E-05	0.00035036	1.28
RP11-516A11.1	2061.60465	0.340035595	4.216189845	2.48E-05	0.00035264	1.27
HECW2	617.7889752	-0.431246899	-4.215499147	2.49E-05	0.00035312	-1.35
DUSP4	754.7490841	0.418215087	4.21488315	2.50E-05	0.00035379	1.34
TPST1	1252.067266	-0.341816353	-4.213318017	2.52E-05	0.00035589	-1.27
ATP13A3	10163.89641	-0.390586431	-4.212177163	2.53E-05	0.00035715	-1.31
TCIRG1	1838.25025	0.327617595	4.211612314	2.54E-05	0.00035774	1.25
A1BG	68.36456774	0.80187859	4.2112532	2.54E-05	0.00035801	1.74
CNNM2	278.6571251	-0.567778677	-4.209367821	2.56E-05	0.0003604	-1.48
VPS28	1066.94628	0.384498389	4.205768362	2.60E-05	0.00036587	1.31
TMSB10	41390.67719	0.41272567	4.205444539	2.61E-05	0.00036609	1.33
TMEM129	1513.978812	0.329903549	4.204466152	2.62E-05	0.00036737	1.26
RCBTB1	838.9164006	-0.36847466	-4.202663441	2.64E-05	0.00037	-1.29
TIMELESS	243.7847579	-0.567213588	-4.199774778	2.67E-05	0.00037443	-1.48
RPL41P1	758.5073996	0.472579026	4.198210626	2.69E-05	0.00037671	1.39
CTB-161M19.3	889.319311	-0.432767868	-4.196839327	2.71E-05	0.00037868	-1.35
IMPA2	151.9947748	0.651191361	4.19650862	2.71E-05	0.00037892	1.57
MCC	331.3048239	-0.531517634	-4.1940428	2.74E-05	0.00038274	-1.45
ZFHX3	941.3335315	-0.493511547	-4.193825921	2.74E-05	0.00038279	-1.41
ROMO1	825.3713392	0.52778551	4.192323619	2.76E-05	0.00038501	1.44
TUFT1	716.3596547	0.386019555	4.189140122	2.80E-05	0.00039013	1.31
RP11-884K10.5	517.5115633	0.452179117	4.186204941	2.84E-05	0.00039487	1.37
ZMYND19	421.1918209	0.46363537	4.183870525	2.87E-05	0.00039829	1.38

RP11-402L1.1	796.1600917	-0.426212279	-4.181969894	2.89E-05	0.0004013	-1.34
ABHD2	4210.724896	-0.394112775	-4.18106222	2.90E-05	0.00040224	-1.31
BTN2A1	1070.58968	0.345255631	4.178944524	2.93E-05	0.00040533	1.27
TP53INP1	952.0744184	-0.3770934	-4.179021477	2.93E-05	0.00040533	-1.30
TRPT1	446.0710373	0.450905394	4.175543784	2.97E-05	0.00041109	1.37
SLC7A7	58.87247295	-0.822787543	-4.175238629	2.98E-05	0.0004113	-1.77
CCNB1	306.1207963	-0.49562867	-4.170141028	3.04E-05	0.00042026	-1.41
S100A10	4079.493281	0.36162379	4.168515942	3.07E-05	0.00042292	1.28
AL049757.3	1654.187547	-0.388983919	-4.165510081	3.11E-05	0.00042783	-1.31
COX8A	1820.326505	0.354736989	4.164316853	3.12E-05	0.00042955	1.28
GALNT12	39.01515173	-0.8869558	-4.16421685	3.12E-05	0.00042955	-1.85
NT5C2	2244.816056	-0.325508568	-4.162775764	3.14E-05	0.00043192	-1.25
DCUN1D3	805.072118	0.359998198	4.157882819	3.21E-05	0.00044054	1.28
ERGIC3	6987.070489	0.324478427	4.154398212	3.26E-05	0.00044694	1.25
PGDP1	191.6405146	-0.565217545	-4.153819424	3.27E-05	0.00044771	-1.48
ITGAV	18887.63132	-0.347110441	-4.153507452	3.27E-05	0.00044795	-1.27
NDUFA11	1066.717446	0.432619656	4.153000296	3.28E-05	0.00044858	1.35
PRAF2	1338.653606	0.351278526	4.149321465	3.33E-05	0.00045548	1.28
COX6A1	1203.079511	0.444342598	4.147978392	3.35E-05	0.00045778	1.36
MBD6	745.9141841	-0.385683528	-4.145863141	3.39E-05	0.00046165	-1.31
SHISA4	837.704943	0.384787306	4.142598755	3.43E-05	0.00046751	1.31
DHRS9	39.88703633	0.889136619	4.136382348	3.53E-05	0.00047996	1.85
IL11RA	488.1462328	0.425209661	4.136027516	3.53E-05	0.00048031	1.34
SMC4	682.8658812	-0.375939574	-4.133039121	3.58E-05	0.00048581	-1.30
PATL1	1032.345956	-0.406913375	-4.128675829	3.65E-05	0.00049432	-1.33
DDI2	548.9671848	-0.448200399	-4.128433904	3.65E-05	0.00049444	-1.36
DPCD	407.2273152	0.499427782	4.125100641	3.71E-05	0.00050018	1.41
CHCHD2	3279.680633	0.421594528	4.125142333	3.71E-05	0.00050018	1.34
STAM	984.357381	-0.393557866	-4.125031731	3.71E-05	0.00050018	-1.31
TMX1	1046.050516	0.369058532	4.123621574	3.73E-05	0.00050285	1.29
RP11-569G13.1	399.3584315	-0.460816956	-4.120965813	3.77E-05	0.00050827	-1.38
VPS8	1008.364577	-0.379960808	-4.118867344	3.81E-05	0.0005121	-1.30
AR	120.9708559	-0.676323163	-4.114110376	3.89E-05	0.00052193	-1.60
S1PR3	271.2545852	0.509974006	4.113573657	3.90E-05	0.00052231	1.42
FAM117A	141.2134082	-0.624875319	-4.11362591	3.89E-05	0.00052231	-1.54
CDC20	84.51340551	-0.768582022	-4.109457731	3.97E-05	0.00053128	-1.70
PTGIR	396.1723834	0.494475634	4.108995592	3.97E-05	0.00053191	1.41
FHOD1	1512.757344	0.327907733	4.106923386	4.01E-05	0.00053628	1.26
TMEM194A	294.7458355	-0.491457748	-4.105235227	4.04E-05	0.00053978	-1.41
ARHGAP31	1668.368984	-0.417959029	-4.104836219	4.05E-05	0.00054028	-1.34
TMEM97	186.413452	-0.565100917	-4.104418179	4.05E-05	0.00054039	-1.48

ACSS2	720.4942785	-0.375205097	-4.102706503	4.08E-05	0.00054397	-1.30
PROSER1	500.3286118	-0.442953934	-4.100149469	4.13E-05	0.00054958	-1.36
PRRC2C	3595.542541	-0.482661934	-4.097776055	4.17E-05	0.0005548	-1.40
CNTNAP3B	782.2403844	0.407617183	4.096517667	4.19E-05	0.00055738	1.33
AC097639.4	21.10098653	0.938414787	4.095775235	4.21E-05	0.00055784	1.92
MAGI2	144.2683426	-0.606219063	-4.095869958	4.21E-05	0.00055784	-1.52
NCKIPSD	644.0486004	0.388470056	4.094152566	4.24E-05	0.00056087	1.31
RANGAP1	2627.976709	0.365073341	4.093238382	4.25E-05	0.00056219	1.29
RASSF3	584.1910379	-0.392478036	-4.092098542	4.27E-05	0.00056452	-1.31
WBSCR22	1025.380961	0.371899839	4.090644091	4.30E-05	0.00056673	1.29
ALMS1	297.0732232	-0.539443439	-4.090669318	4.30E-05	0.00056673	-1.45
RPLP2	6322.106048	0.392592959	4.09020506	4.31E-05	0.00056735	1.31
COPE	2583.050194	0.33737318	4.088551995	4.34E-05	0.00057096	1.26
GBAP1	410.6489318	-0.451356624	-4.085442187	4.40E-05	0.00057821	-1.37
RP11-212I21.3	320.954239	-0.576798733	-4.085132161	4.41E-05	0.00057853	-1.49
CALB2	28.23589534	-0.912577494	-4.084050021	4.43E-05	0.00058077	-1.88
PLEKHO1	941.93694	-0.3875214	-4.081915041	4.47E-05	0.00058567	-1.31
METTL7A	124.0076693	-0.65663445	-4.077149718	4.56E-05	0.00059733	-1.58
TBC1D2B	2154.195073	-0.362183171	-4.074960202	4.60E-05	0.00060204	-1.29
STRIP2	168.2408969	-0.638384716	-4.075004201	4.60E-05	0.00060204	-1.56
LDLRAD4	407.9097292	-0.566729287	-4.071045496	4.68E-05	0.00061177	-1.48
NAXD	637.6017935	0.391995027	4.070379185	4.69E-05	0.00061304	1.31
RP11-109P6.1	105.3843952	-0.664711731	-4.064995777	4.80E-05	0.00062687	-1.59
RP11-527H14.3	28.90563482	-0.915630788	-4.06189413	4.87E-05	0.00063378	-1.89
PHPT1	1437.375962	0.416686709	4.061179352	4.88E-05	0.00063523	1.33
CALM2	13884.74601	0.365647945	4.05878762	4.93E-05	0.00064127	1.29
TPX2	381.5114263	-0.446011282	-4.058124396	4.95E-05	0.00064259	-1.36
RP11-480I12.3	148.1207478	0.614248455	4.05493356	5.01E-05	0.00064991	1.53
SPRYD7	419.3531874	0.452575067	4.053156521	5.05E-05	0.00065385	1.37
YLPM1	1471.658955	-0.356489597	-4.052657319	5.06E-05	0.00065474	-1.28
CNOT8	1330.310995	0.353037326	4.052202246	5.07E-05	0.000655	1.28
LRFN4	493.3288916	-0.414916695	-4.050962692	5.10E-05	0.00065797	-1.33
SPINT2	100.7456386	0.676366238	4.049666901	5.13E-05	0.00066061	1.60
FUT10	233.2227099	-0.507677541	-4.048404131	5.16E-05	0.00066367	-1.42
NNT	2128.233298	-0.334778574	-4.045369116	5.22E-05	0.00067129	-1.26
RP11-393M18.2	128.0796113	-0.637023001	-4.042819994	5.28E-05	0.00067759	-1.56
AC012667.1	24.92557461	0.91803881	4.042504851	5.29E-05	0.00067798	1.89
CENPT	1082.758853	0.37180127	4.042012811	5.30E-05	0.00067889	1.29
MAPK13	328.3498883	0.474915489	4.039949796	5.35E-05	0.00068377	1.39
SHCBP1	134.9836855	-0.613155577	-4.039794851	5.35E-05	0.00068377	-1.53
RCAN2	466.9164002	-0.410597391	-4.037677769	5.40E-05	0.00068944	-1.33

MFHAS1	411.1189963	-0.44427675	-4.037260229	5.41E-05	0.00069014	-1.36
PID1	427.2586028	-0.420769913	-4.035669335	5.44E-05	0.0006943	-1.34
ACVR2A	724.347344	0.367515817	4.033449999	5.50E-05	0.00070036	1.29
ARHGAP24	759.1402336	-0.372857904	-4.031980362	5.53E-05	0.00070422	-1.29
POLR1A	1042.250135	-0.437974565	-4.028173649	5.62E-05	0.00071463	-1.35
DYRK2	1070.344935	-0.371087396	-4.026652715	5.66E-05	0.00071837	-1.29
RBM19	477.2479937	-0.405529873	-4.026585645	5.66E-05	0.00071837	-1.32
PIP4K2A	1130.328278	-0.356392929	-4.026206385	5.67E-05	0.00071899	-1.28
SP1	1581.810101	-0.395516161	-4.025079394	5.70E-05	0.00072189	-1.32
CCDC190	73.23233238	0.737403036	4.02438418	5.71E-05	0.00072348	1.67
CTD-2319I12.1	268.4227466	0.575656883	4.023795143	5.73E-05	0.00072455	1.49
FAU	2680.91021	0.37758269	4.023680019	5.73E-05	0.00072455	1.30
SYNGR1	322.4327737	0.456823587	4.022371617	5.76E-05	0.00072803	1.37
EMX2OS	566.3186656	-0.408274574	-4.021232832	5.79E-05	0.00073101	-1.33
ENTPD6	2569.089071	0.323649107	4.020490939	5.81E-05	0.00073276	1.25
PLPP5	1006.924624	0.341676262	4.019931318	5.82E-05	0.00073395	1.27
SKAP2	819.4319123	-0.36091999	-4.017805359	5.87E-05	0.00074004	-1.28
E2F7	402.2257323	-0.463813722	-4.01757074	5.88E-05	0.00074022	-1.38
RMDN3	1242.128063	0.389137087	4.012234217	6.01E-05	0.00075602	1.31
CMB9-22P13.1	144.8961402	0.614466139	4.006211412	6.17E-05	0.00077264	1.53
KCNK1	155.1781471	0.582616628	4.006264686	6.17E-05	0.00077264	1.50
A2M	199.535893	-0.55676793	-4.006429517	6.16E-05	0.00077264	-1.47
TRAF1	281.7548163	0.484786863	4.002884839	6.26E-05	0.00078067	1.40
SCRN2	493.7717464	0.437198861	4.003017827	6.25E-05	0.00078067	1.35
CXCL8	67.77598085	0.747816548	4.00151298	6.29E-05	0.00078404	1.68
RP11-296E7.1	361.4103911	-0.591289938	-4.001677621	6.29E-05	0.00078404	-1.51
DUXAP9	85.67001964	-0.755773772	-3.997983181	6.39E-05	0.00079463	-1.69
VIMP	1811.776992	0.348919226	3.995813708	6.45E-05	0.00080135	1.27
ATG4B	1058.887849	0.330131979	3.988857111	6.64E-05	0.0008246	1.26
C17orf107	192.8076403	0.540167785	3.987235373	6.68E-05	0.00082964	1.45
PDE11A	149.1019724	0.581567759	3.986716145	6.70E-05	0.00083084	1.50
NDUFB7	1187.607804	0.419362745	3.983959134	6.78E-05	0.00083992	1.34
GS1-358P8.4	109.1405774	0.668809247	3.9770368	6.98E-05	0.00086282	1.59
PRPS1	2548.501432	0.344366479	3.973935868	7.07E-05	0.00087349	1.27
RP11-501M7.2	200.8759728	-0.561801216	-3.972738118	7.11E-05	0.00087648	-1.48
MOSPD1	653.5694912	-0.385727069	-3.965322242	7.33E-05	0.00090038	-1.31
IQGAP3	91.7917163	-0.683654467	-3.96530311	7.33E-05	0.00090038	-1.61
RABGAP1	1400.357474	-0.32257812	-3.964521963	7.35E-05	0.00090267	-1.25
SEMA3B	410.0536351	0.425951983	3.959714838	7.50E-05	0.00091901	1.34
TFPI	2515.549156	0.335822994	3.959224797	7.52E-05	0.00092022	1.26
C12orf49	1132.168417	-0.33687251	-3.958034559	7.56E-05	0.00092414	-1.26

KRT14	63.01321318	0.7683852	3.956648825	7.60E-05	0.00092884	1.70
MKL2	364.7313999	-0.507964362	-3.955426012	7.64E-05	0.00093292	-1.42
EMC3	1510.805689	0.346348052	3.955002154	7.65E-05	0.00093389	1.27
UNKL	592.1205783	0.388363935	3.954328629	7.67E-05	0.00093584	1.31
RGL1	1137.354001	-0.388458318	-3.948447965	7.87E-05	0.00095564	-1.31
PIANP	112.1622154	0.643161644	3.948269747	7.87E-05	0.00095566	1.56
NAP1L1P1	465.3490777	-0.416121395	-3.945305086	7.97E-05	0.00096616	-1.33
MIRLET7BHG	199.2442519	0.554599925	3.944107828	8.01E-05	0.00097029	1.47
LTBP4	495.7114807	0.402388673	3.940278402	8.14E-05	0.0009852	1.32
AEBP1	801.3872198	-0.463844911	-3.939628406	8.16E-05	0.00098715	-1.38
ALPK1	226.1037915	-0.515824228	-3.935745536	8.29E-05	0.0010018	-1.43
PSENEN	502.3883129	0.437739983	3.931015694	8.46E-05	0.00102024	1.35
RP11-357C3.3	406.8683702	-0.43257461	-3.926788884	8.61E-05	0.00103758	-1.35
SOD3	55.2483079	-0.79322014	-3.926382096	8.62E-05	0.00103859	-1.73
TNRC6B	611.8027794	-0.442014833	-3.921312271	8.81E-05	0.00105993	-1.36
INPP5A	721.7376461	0.376231831	3.920729538	8.83E-05	0.00106173	1.30
SMC1A	1295.027566	-0.341005989	-3.916339151	8.99E-05	0.00108046	-1.27
SERTAD3	434.4228189	0.436063101	3.915210458	9.03E-05	0.00108475	1.35
EPS8L2	660.9756143	0.376040966	3.91130287	9.18E-05	0.00109929	1.30
ARPC5L	761.7098892	0.387301539	3.910206868	9.22E-05	0.0011035	1.31
DNAJA4	121.0139364	0.629206001	3.908918025	9.27E-05	0.00110782	1.55
SHANK1	89.42850445	0.705245337	3.906824623	9.35E-05	0.00111666	1.63
RP4-702J19.1	224.8578774	-0.500872614	-3.900209245	9.61E-05	0.00114597	-1.42
SMCR8	985.3246154	-0.436681555	-3.897607455	9.71E-05	0.00115753	-1.35
PRICKLE1	531.6029098	0.417062179	3.89585338	9.79E-05	0.00116511	1.34
NCAPG	96.94003875	-0.680465711	-3.894695868	9.83E-05	0.00116902	-1.60
FRMD4A	931.5958217	-0.34704243	-3.891183837	9.98E-05	0.0011828	-1.27
LDHBP	667.8708771	-0.365911541	-3.891164697	9.98E-05	0.0011828	-1.29
FAM102B	197.7439674	-0.520391301	-3.890907246	9.99E-05	0.00118321	-1.43
ANKRD10	438.1501807	-0.428860084	-3.890553718	0.000100016	0.0011841	-1.35
JMJD1C	1139.583104	-0.407848405	-3.889215898	0.000100569	0.0011898	-1.33
RP11-450H5.2	69.63348331	-0.75045193	-3.887420001	0.000101315	0.00119779	-1.68
NAAA	889.3194749	0.33275218	3.886806133	0.000101572	0.00119997	1.26
TMEM179B	810.2011919	0.364940487	3.885663899	0.000102051	0.00120477	1.29
ADRA1D	67.09525239	0.734401623	3.884251441	0.000102646	0.00121056	1.66
PLXND1	5350.850242	-0.322171143	-3.884157361	0.000102685	0.00121056	-1.25
VPS50	470.4270799	-0.412124569	-3.88270835	0.000103299	0.00121694	-1.33
RCN3	17707.38505	0.339546714	3.881812258	0.000103681	0.00122057	1.27
KMT2E	1347.849417	-0.424147831	-3.878562426	0.000105076	0.00123438	-1.34
RNF169	448.848264	-0.444147941	-3.876873564	0.000105807	0.0012421	-1.36
IL6STP1	238.5426452	-0.587089101	-3.875423164	0.00010644	0.00124865	-1.50

ZC3H12C	250.7090054	-0.524385557	-3.873802732	0.00010715	0.0012561	-1.44
KIFC1	88.27775809	-0.695135099	-3.871977954	0.000107956	0.00126466	-1.62
BAIAP2L1	712.9675483	0.358007285	3.871375652	0.000108223	0.0012669	1.28
PI4K2B	423.1744345	0.404660044	3.871017426	0.000108382	0.00126787	1.32
MED1	1232.691327	-0.478672313	-3.86367204	0.000111695	0.00130207	-1.39
GDF5	679.6757127	0.367079951	3.86315553	0.000111932	0.00130392	1.29
LFNG	415.1121825	0.430672962	3.862389837	0.000112283	0.0013071	1.35
DZIP3	350.9225014	-0.443079747	-3.860774958	0.000113028	0.00131486	-1.36
RBMXL1	393.692172	-0.411425035	-3.859756773	0.0001135	0.00131943	-1.33
MN1	480.6017402	-0.438657211	-3.859381383	0.000113674	0.00132054	-1.36
RNF112	37.72051587	0.828751819	3.858799498	0.000113945	0.00132276	1.78
MAFK	1049.614156	0.365367102	3.857316186	0.000114639	0.00132713	1.29
SLC35A2	962.9008955	0.343213434	3.85733081	0.000114632	0.00132713	1.27
SPCS2	447.185925	0.473507041	3.853693809	0.000116349	0.00134599	1.39
MVK	262.1069085	-0.491941761	-3.853235852	0.000116567	0.00134758	-1.41
BAHCC1	550.0125017	-0.420395652	-3.852743955	0.000116802	0.00134936	-1.34
SCRG1	524.1838066	0.397192224	3.851634657	0.000117332	0.00135455	1.32
PTPRF	4191.246782	0.408511369	3.849964433	0.000118135	0.00136199	1.33
NUP188	1235.017061	-0.397325649	-3.849953802	0.00011814	0.00136199	-1.32
SPHK1	1228.261196	-0.367384487	-3.849425088	0.000118395	0.001364	-1.29
RP11-449M6.1	114.4935134	-0.627374672	-3.847661326	0.000119251	0.00137196	-1.54
C6orf1	577.7963887	-0.369085607	-3.84529117	0.000120409	0.00138243	-1.29
TMEM222	1060.95853	0.33813583	3.844682397	0.000120709	0.00138492	1.26
RPSAP58	6316.198739	0.379410149	3.842323222	0.000121875	0.00139734	1.30
AGT	38.74730394	-0.812871912	-3.839471065	0.0001233	0.0014127	-1.76
UQCR11	1390.875714	0.362479375	3.838013897	0.000124033	0.00141958	1.29
RP11-434D2.6	142.7892468	-0.631726931	-3.837931852	0.000124075	0.00141958	-1.55
IL32	93.09167447	-0.673013052	-3.837774535	0.000124154	0.00141958	-1.59
RP11-274H2.5	33.81110749	0.838424971	3.837144398	0.000124473	0.00142225	1.79
PHF3	1192.057424	-0.362696444	-3.836809078	0.000124643	0.00142322	-1.29
RHCE	42.99003337	0.803785845	3.836634023	0.000124732	0.00142326	1.75
TPM2	36616.79129	0.352087232	3.83543981	0.00012534	0.00142922	1.28
DROSHA	1136.894383	-0.328353942	-3.832320649	0.00012694	0.00144648	-1.26
CD320	970.70929	0.336523291	3.831304873	0.000127465	0.00145147	1.26
CLTA	3898.902234	-0.329637164	-3.830558603	0.000127853	0.00145489	-1.26
SYNE3	536.1676288	-0.444288208	-3.830157133	0.000128061	0.00145627	-1.36
CPEB1	68.98428129	-0.718487032	-3.829721174	0.000128289	0.00145786	-1.65
ANK3	507.9149675	-0.455348021	-3.82685806	0.000129789	0.00147191	-1.37
RPL18A	637.1696636	0.369919553	3.823385719	0.000131632	0.00148977	1.29
FOXD1	1330.038342	0.333106924	3.823047652	0.000131812	0.00149081	1.26
CPNE2	350.2864023	-0.429328292	-3.822783776	0.000131953	0.0014914	-1.35

SLC46A1	601.9577871	0.38939771	3.818483562	0.000134275	0.00151456	1.31
AC005014.5	129.6977336	-0.642999984	-3.818008783	0.000134533	0.00151645	-1.56
MAP2	75.71900311	-0.702715339	-3.817048964	0.000135057	0.00152133	-1.63
RP11-704G19.1	593.7177637	-0.368166951	-3.814347535	0.000136543	0.001536	-1.29
NRSN2	1175.210673	0.326834974	3.813888844	0.000136797	0.00153782	1.25
MCM6	585.6772773	-0.369138879	-3.812548025	0.000137541	0.00154291	-1.29
SPRN	61.50572187	0.737034221	3.810782656	0.000138528	0.00154997	1.67
AUTS2	632.8335676	-0.365797318	-3.806074462	0.00014119	0.00157659	-1.29
ESF1	458.5984305	-0.411443526	-3.80397561	0.000142392	0.00158895	-1.33
DGKQ	670.6542344	0.344378956	3.802762095	0.000143092	0.00159569	1.27
GAD1	51.90474773	0.804585999	3.802162032	0.000143439	0.0015985	1.75
MTX1	320.4309526	0.451278455	3.797035772	0.000146437	0.00163082	1.37
ERCC1	1745.801198	0.328095165	3.795762223	0.000147191	0.00163703	1.26
PASK	54.75630285	-0.761723225	-3.795769911	0.000147186	0.00163703	-1.70
HLA-L	217.2385243	0.519178291	3.793506343	0.000148535	0.00164978	1.43
LCAT	221.3087631	0.499675189	3.791741883	0.000149594	0.00165935	1.41
LRRC15	453.7628992	-0.448100066	-3.791501867	0.000149739	0.00165985	-1.36
RP11-730G20.2	967.7965866	-0.336571005	-3.789880846	0.00015072	0.00166961	-1.26
G0S2	92.33928973	-0.647724084	-3.789141484	0.000151169	0.00167348	-1.57
MEGF9	894.592786	-0.402198333	-3.787196154	0.000152357	0.00168439	-1.32
COX6A1P2	1439.348369	0.357645789	3.786996245	0.000152479	0.00168464	1.28
ARHGEF3	280.9075493	-0.454183505	-3.78607584	0.000153045	0.00168977	-1.37
MYEF2	302.0382883	0.440741335	3.785484903	0.000153409	0.00169267	1.36
NAT9	964.5730138	0.33378505	3.78343153	0.000154681	0.00170557	1.26
POTEF	473.4057122	0.429981098	3.782050234	0.000155542	0.00171287	1.35
PRRT3	279.8459726	0.446334622	3.781573598	0.00015584	0.00171496	1.36
RP5-831D17.2	177.6300439	-0.57179447	-3.781300385	0.000156011	0.00171571	-1.49
STAG1	1226.126927	-0.326005276	-3.780631389	0.000156431	0.0017192	-1.25
ARHGAP23	3112.643457	-0.329496623	-3.779410221	0.0001572	0.00172553	-1.26
PTPRU	270.1665712	-0.462968038	-3.779389472	0.000157213	0.00172553	-1.38
DOPEY2	575.1131841	-0.392729789	-3.778160627	0.000157991	0.00173178	-1.31
SLC9A3R1	364.6227139	-0.419018912	-3.777362899	0.000158498	0.00173506	-1.34
HSD17B14	13.44085194	-0.846895635	-3.777377484	0.000158488	0.00173506	-1.80
JAG1	2217.491265	-0.334218004	-3.777191656	0.000158607	0.00173512	-1.26
ASPHD1	177.6507783	0.540809958	3.773367793	0.000161059	0.00175964	1.45
TM4SF4	26.01765925	0.849534793	3.772454562	0.000161649	0.00176494	1.80
RP1-222H5.1	881.1770443	-0.327422116	-3.771940571	0.000161983	0.00176742	-1.25
RP11-367E12.4	326.2142151	-0.432744045	-3.771507184	0.000162264	0.00176819	-1.35
RP11-550F7.1	1101.974854	-0.461641436	-3.771582508	0.000162216	0.00176819	-1.38
SNN	651.2562348	-0.357951599	-3.770116446	0.000163171	0.00177691	-1.28
FBN2	690.0545583	-0.48282688	-3.767092425	0.00016516	0.00179505	-1.40

TAPT1	554.1390259	0.361316054	3.764360935	0.000166976	0.00181361	1.28
BMPR1B	64.28481394	-0.732277654	-3.76324549	0.000167722	0.00181935	-1.66
MIER2	800.036703	0.340336126	3.760059514	0.000169873	0.00184015	1.27
ZNF106	2596.217193	-0.384270326	-3.759916576	0.00016997	0.00184015	-1.31
TSPYL2	1003.653253	0.352678675	3.758525694	0.000170917	0.00184874	1.28
VSIG10	274.6163357	-0.452803006	-3.758041444	0.000171249	0.00185039	-1.37
SYPL2	42.37575923	-0.807702749	-3.757732411	0.00017146	0.00185148	-1.75
PLA2G4A	248.7880336	-0.484038895	-3.757209635	0.000171819	0.00185415	-1.40
TGFBR2	13173.09309	0.32511488	3.756653206	0.000172201	0.00185708	1.25
ZFYVE16	848.8544587	-0.362162489	-3.753791556	0.00017418	0.001876	-1.29
DUS1L	698.4999942	0.343811309	3.752005663	0.000175426	0.00188577	1.27
RHBDF2	240.1669906	-0.499512707	-3.747807774	0.000178387	0.00191637	-1.41
MLLT10	408.7723086	-0.418299481	-3.745204582	0.000180247	0.0019351	-1.34
IGIP	442.5293522	0.429386047	3.744330481	0.000180875	0.00194061	1.35
DCXR	434.6986495	0.454671794	3.743795458	0.000181261	0.00194225	1.37
RP11-333E13.2	141.4640969	-0.59147904	-3.74381133	0.00018125	0.00194225	-1.51
RP1-140K8.5	207.7997849	0.605649816	3.742517522	0.000182186	0.00195034	1.52
RP11-832N8.1	431.4679559	0.440055123	3.742064093	0.000182515	0.00195068	1.36
XPOTP1	588.6827685	-0.40467131	-3.740877971	0.000183379	0.00195866	-1.32
EIF2S3L	1129.541144	-0.368123969	-3.740672174	0.000183529	0.00195901	-1.29
BIRC5	140.058341	-0.558980876	-3.739390632	0.000184467	0.00196776	-1.47
CBLB	1783.474202	-0.381327423	-3.73793718	0.000185536	0.00197791	-1.30
NDUFS8	976.2300076	0.363086024	3.736933398	0.000186278	0.00198455	1.29
TNK2	1074.497927	0.32769594	3.736533929	0.000186574	0.00198644	1.26
SCLY	178.8114363	-0.548201369	-3.736242008	0.000186791	0.00198748	-1.46
SYTL2	349.5206303	-0.418296965	-3.735712854	0.000187184	0.0019904	-1.34
DOCK1	2681.484409	-0.372119266	-3.730764966	0.000190899	0.00202603	-1.29
ZC3HAV1	1145.731595	-0.339692956	-3.73023629	0.0001913	0.002029	-1.27
BDKRB2	768.514552	0.34853422	3.728502277	0.000192621	0.00204171	1.27
FGD6	198.2864333	-0.528905274	-3.727440466	0.000193434	0.00204716	-1.44
MAMLD1	314.8702547	-0.472783231	-3.726123957	0.000194447	0.00205454	-1.39
KIF11	148.0353824	-0.562554981	-3.72587864	0.000194636	0.00205524	-1.48
RP11-278C7.1	560.3078104	-0.392022882	-3.724784963	0.000195482	0.00206287	-1.31
TMEM134	397.3021335	0.458416527	3.723899555	0.000196169	0.00206882	1.37
SPEN	1181.322649	-0.434409517	-3.723063392	0.00019682	0.00207412	-1.35
DPYSL4	470.6260991	0.381975913	3.720546939	0.000198792	0.00208725	1.30
DUSP8	146.6649021	0.553586988	3.719721699	0.000199442	0.00209277	1.47
LINC00294	280.2921614	0.465907976	3.719400604	0.000199696	0.00209411	1.38
DGUOK	695.9564002	0.366230338	3.717047742	0.000201564	0.00211105	1.29
SCD5	449.800703	0.378561541	3.716873837	0.000201703	0.00211118	1.30
KIAA1551	335.7328916	-0.430849744	-3.714721554	0.000203428	0.0021279	-1.35

FOXO1	124.5359307	-0.5962585	-3.713540125	0.00020438	0.00213653	-1.51
CD55	416.5332033	-0.389343435	-3.712478702	0.000205239	0.00214283	-1.31
AC073115.6	75.33896625	0.714070484	3.710366975	0.000206959	0.00215943	1.64
SLC2A12	206.2382783	-0.510627918	-3.706423261	0.000210207	0.00218922	-1.42
PCDHGB2	130.1502097	-0.600435745	-3.705919272	0.000210625	0.00219221	-1.52
MRPL17	1137.918695	0.374533497	3.704490166	0.000211816	0.00220296	1.30
RBX1	880.6844528	0.369806937	3.702030539	0.000213881	0.00222194	1.29
GAS7	62.92113178	-0.715960058	-3.700932078	0.000214809	0.0022302	-1.64
MCOLN1	714.5206795	-0.337422909	-3.697801774	0.000217475	0.00225507	-1.26
ARHGAP11A	196.6162594	-0.514382067	-3.696722084	0.000218401	0.00226187	-1.43
ARPIN	496.4255037	-0.364513818	-3.696313841	0.000218753	0.0022641	-1.29
GSTM3	1176.675257	0.348233004	3.694681462	0.000220163	0.00227466	1.27
C14orf1	739.0017221	-0.377101424	-3.694967347	0.000219915	0.00227466	-1.30
RP11-259K5.2	133.4477258	-0.555791575	-3.690152762	0.000224119	0.00230561	-1.47
RPS21	2249.565676	0.373073027	3.687819371	0.000226184	0.00232517	1.30
CTXN1	223.5439704	0.519651829	3.686741643	0.000227144	0.0023336	1.43
WDR35	247.1022302	-0.45108759	-3.684767746	0.000228911	0.00235032	-1.37
JOSD2	641.5891771	0.395302554	3.683952942	0.000229645	0.00235592	1.32
CNOT1	2842.787062	-0.445639456	-3.683089585	0.000230424	0.00236149	-1.36
RP11-798M19.6	34.69039651	0.801095154	3.671201362	0.000241413	0.00246504	1.74
SLC23A2	987.3866377	-0.433481083	-3.670813893	0.000241779	0.00246727	-1.35
RP11-1136G11.6	265.6799573	-0.472133072	-3.668045205	0.000244412	0.00249261	-1.39
TFPI2	539.0515829	0.39082757	3.667738874	0.000244705	0.00249408	1.31
ARL3	756.193873	0.393774663	3.666615982	0.000245781	0.00250256	1.31
UQCRQ	1709.916282	0.371137892	3.666558277	0.000245837	0.00250256	1.29
PGAM4	241.0493895	0.483750296	3.665798041	0.000246568	0.00250848	1.40
ISOC2	916.428921	0.328559097	3.664105294	0.000248205	0.00252359	1.26
SLC2A8	91.33477916	0.63991359	3.663879913	0.000248423	0.00252386	1.56
PA2G4P4	308.9492707	-0.429773595	-3.66376633	0.000248534	0.00252386	-1.35
LYPD6B	1116.588316	0.332581799	3.66342185	0.000248868	0.00252572	1.26
FER	769.8874479	-0.425009394	-3.663019866	0.000249259	0.00252816	-1.34
SOBP	113.978319	-0.603290469	-3.658957106	0.000253244	0.0025639	-1.52
HSD17B7	66.52622923	-0.699979881	-3.659084304	0.000253118	0.0025639	-1.62
AC090602.1	828.804769	-0.341470952	-3.658603308	0.000253594	0.00256589	-1.27
EBF1	554.3318312	-0.373364041	-3.658145343	0.000254047	0.00256737	-1.30
ARHGAP21	2085.495726	-0.40819233	-3.656445342	0.000255737	0.00258288	-1.33
KIAA1755	222.0124512	0.515548916	3.655012821	0.000257169	0.00259264	1.43
RGAG4	505.4301281	-0.369203599	-3.655138491	0.000257043	0.00259264	-1.29
CENPP	73.21632365	-0.669425043	-3.654231983	0.000257953	0.00259898	-1.59
TMEM141	414.2295935	0.444296413	3.653503535	0.000258686	0.00260479	1.36
DAPK2	66.59725733	-0.690241699	-3.652492831	0.000259707	0.00261192	-1.61

CEP295	241.1274307	-0.470955732	-3.650260864	0.000261974	0.00263156	-1.39
HIST1H2BD	240.9566456	0.454630237	3.649618388	0.00026263	0.00263498	1.37
RPS29	1616.86212	0.401161629	3.649698179	0.000262549	0.00263498	1.32
RP5-848E13.1	474.4402875	-0.366545442	-3.645855164	0.000266504	0.00267064	-1.29
NECAB3	274.7121825	0.432150445	3.644241592	0.000268181	0.00268583	1.35
CHRDL1	751.4737207	0.346007266	3.640871657	0.000271717	0.00271961	1.27
NAT14	337.267968	0.423810666	3.636533363	0.000276332	0.00276415	1.34
EVI2B	33.28537337	-0.802719113	-3.63496825	0.000278015	0.00277932	-1.74
AVPI1	655.2342706	-0.346858588	-3.63432278	0.000278712	0.00278462	-1.27
WDR12	543.7411049	-0.371286037	-3.63379728	0.00027928	0.00278863	-1.29
BRCA1	124.5527408	-0.586021457	-3.633414574	0.000279695	0.0027911	-1.50
DNAJC25	373.2999043	0.406459278	3.631818464	0.000281431	0.0028034	1.33
PTPN23	1366.875104	-0.37445796	-3.624691331	0.000289307	0.00287671	-1.30
SNX7	942.918744	0.341863892	3.623318676	0.000290847	0.0028903	1.27
SPNS1	176.5358731	-0.543783542	-3.622612949	0.000291642	0.00289476	-1.46
BUB1	85.0731446	-0.629116157	-3.622357481	0.00029193	0.0028959	-1.55
TACC3	163.5381554	-0.537120043	-3.621858176	0.000292494	0.00289977	-1.45
SPG11	1023.506017	-0.406107326	-3.621696731	0.000292677	0.00289986	-1.33
ZC2HC1A	320.0737855	-0.418721156	-3.620608427	0.000293911	0.00291007	-1.34
CDH15	140.3953087	0.549503174	3.617454632	0.000297514	0.00294082	1.46
LAMA3	1072.584983	-0.426762244	-3.616167758	0.000298997	0.00295197	-1.34
SNX30	1115.137088	-0.34755182	-3.6147332	0.000300657	0.00296395	-1.27
FBXL7	732.9370951	-0.357540499	-3.614425197	0.000301015	0.00296489	-1.28
TUBG2	500.422218	0.368876262	3.612643513	0.000303091	0.00298183	1.29
PSMB5	4631.005386	0.34189231	3.611744178	0.000304145	0.00298601	1.27
CTD-2319I12.10	85.48161198	-0.641664445	-3.610643199	0.000305439	0.0029961	-1.56
WISP1	877.8785311	-0.349950767	-3.608566588	0.000307893	0.0030184	-1.27
SLCO2B1	43.42620562	-0.744182575	-3.606603921	0.000310231	0.00303775	-1.68
NAMPTL	320.9734429	-0.411693016	-3.605471527	0.000311587	0.00304746	-1.33
NAPEPLD	234.5929721	-0.460239122	-3.604940142	0.000312225	0.00305191	-1.38
NUDT22	496.9576487	0.403167789	3.60411426	0.000313219	0.00305984	1.32
MIB2	1112.445857	0.345117903	3.598465598	0.0003201	0.00312341	1.27
RP11-241I20.3	541.9876356	-0.414155287	-3.596479111	0.000322553	0.00314184	-1.33
ZBED3	158.22678	-0.573587311	-3.592685009	0.000327288	0.00318424	-1.49
AKAP2	117.0674428	-0.593618886	-3.590540941	0.000329992	0.00320682	-1.51
SECTM1	75.76448692	0.691438586	3.589595194	0.000331192	0.00321474	1.61
RBM43	324.3977587	-0.429088826	-3.587230599	0.000334209	0.00324025	-1.35
KLF3	874.3980064	-0.334803346	-3.586004384	0.000335783	0.00325363	-1.26
LRRC20	100.3372276	-0.596729081	-3.58424507	0.000338055	0.00327184	-1.51
NUAK2	141.2664665	0.544945599	3.580078184	0.000343491	0.0033187	1.46
MOK	605.2529801	0.38383542	3.580202242	0.000343328	0.0033187	1.30

USMG5	1435.834222	0.377505479	3.578038097	0.000346183	0.00334018	1.30
PPARG	145.0010291	-0.545927698	-3.577938746	0.000346315	0.00334018	-1.46
USP28	384.9490183	-0.482818338	-3.577580513	0.000346789	0.00334283	-1.40
USP35	783.8692473	-0.362579995	-3.57146651	0.000354988	0.00341988	-1.29
MALL	164.2004927	0.56507879	3.569168852	0.000358116	0.00344405	1.48
RUNX2	348.1697438	-0.440754006	-3.562592542	0.00036721	0.00351588	-1.36
LINC00595	84.7392332	-0.619692595	-3.562551764	0.000367267	0.00351588	-1.54
RP11-571F15.6	1018.410334	-0.346248535	-3.56045556	0.000370212	0.00354203	-1.27
TBC1D24	666.4673543	0.3343845	3.560277845	0.000370463	0.0035424	1.26
ANKRD13D	489.3891629	0.368682969	3.55852568	0.000372942	0.00356204	1.29
ANKRD17	2582.069752	-0.439524163	-3.558559834	0.000372894	0.00356204	-1.36
RP11-97E23.3	141.9873014	-0.555513164	-3.555037475	0.000377925	0.00360345	-1.47
FUOM	89.2970639	0.636696884	3.554352315	0.000378911	0.00360945	1.55
CPNE7	302.748429	-0.424443826	-3.554300218	0.000378986	0.00360945	-1.34
C8orf58	376.036099	0.393764223	3.552555925	0.000381508	0.00363139	1.31
DIAPH2	358.25028	-0.432085247	-3.550582868	0.000384379	0.00365248	-1.35
XAF1	140.1668409	-0.57793811	-3.549809837	0.00038551	0.00365905	-1.49
CCDC97	379.7314959	-0.387117525	-3.549292064	0.000386268	0.00366418	-1.31
NMNAT2	525.2906113	0.364933088	3.548464728	0.000387484	0.00367147	1.29
ARHGEF6	368.5090329	-0.399783128	-3.545634665	0.000391669	0.00370279	-1.32
COL11A2	85.32910079	0.628713713	3.544833266	0.000392862	0.00371196	1.55
ABCC5	683.9888522	-0.376249516	-3.544098602	0.000393958	0.00372022	-1.30
ABCA1	934.6590647	-0.409345242	-3.543697615	0.000394557	0.00372378	-1.33
TMTC1	794.9304714	-0.399534544	-3.543092027	0.000395465	0.00373023	-1.32
PTGER4	104.6003691	-0.575008429	-3.542158479	0.000396867	0.00374134	-1.49
TMEM219	1478.734911	0.357274248	3.540865542	0.000398817	0.00375126	1.28
GRIN2D	54.98466709	0.705388045	3.536663073	0.000405216	0.00380502	1.63
HLA-H	992.9053755	0.361350663	3.532549718	0.000411573	0.00385821	1.28
MOCOS	206.2580675	-0.480917157	-3.529167348	0.000416869	0.00390129	-1.40
CTB-47B8.1	133.7437202	0.536883383	3.524585326	0.000424146	0.00396495	1.45
NDUFB8	336.4714071	0.4701222	3.520992401	0.000429935	0.00401457	1.39
EYA1	65.87241312	-0.670634503	-3.518468697	0.000434045	0.00405068	-1.59
SYDE2	125.3000099	-0.589101406	-3.517821061	0.000435106	0.00405831	-1.50
TBC1D4	904.7772984	0.324769163	3.517391418	0.000435811	0.00406262	1.25
SLC43A3	250.1408566	-0.441184125	-3.513652413	0.000441991	0.00410877	-1.36
AKNA	251.8616432	-0.45278625	-3.513282058	0.000442607	0.00411221	-1.37
SEMA3A	384.3244281	0.412390881	3.511161763	0.000446153	0.00414285	1.33
ADGRA3	430.7783266	-0.431885666	-3.509405634	0.000449109	0.00416798	-1.35
VWA5A	316.2414718	-0.404574512	-3.509146063	0.000449548	0.00416974	-1.32
VAMP5	466.313232	0.428683128	3.508804097	0.000450126	0.00417047	1.35
WRB	856.1798288	0.356109006	3.508834965	0.000450074	0.00417047	1.28

KIF23	263.0017596	-0.437195969	-3.507416334	0.000452481	0.00418996	-1.35
C20orf100	108.1293118	-0.569563781	-3.506392424	0.000454225	0.00420378	-1.48
TRIM16L	328.2559173	-0.400284272	-3.505605464	0.00045557	0.0042139	-1.32
GNL3L	217.7254042	-0.509796429	-3.501707888	0.000462286	0.00426657	-1.42
AP1S1	1838.927105	0.33093209	3.499408527	0.000466292	0.00430116	1.26
CYB561D2	338.5861827	0.390221358	3.496309779	0.000471741	0.00434423	1.31
ASL	595.0405821	0.357425578	3.496451372	0.00047149	0.00434423	1.28
ACOT8	341.7149833	0.407865667	3.494937228	0.000474173	0.00436422	1.33
PI4KAP1	165.3654045	0.501215168	3.492210034	0.000479041	0.00440519	1.42
CHST7	257.8886354	0.431010826	3.492148369	0.000479152	0.00440519	1.35
RHBDL2	54.79391415	0.69560811	3.49071249	0.000481734	0.0044265	1.62
TMEM107	168.2575775	0.525581891	3.489962847	0.000483088	0.00443505	1.44
SMAD6	867.0881368	-0.351974876	-3.489902658	0.000483197	0.00443505	-1.28
VPS9D1	564.6572114	0.367833956	3.486841013	0.000488762	0.0044763	1.29
ZNF468	310.8624374	-0.406458764	-3.485921387	0.000490445	0.00448925	-1.33
AKTIP	551.6431795	0.391611577	3.485277759	0.000491626	0.00449271	1.31
RP4-775C13.1	541.1492075	-0.380870183	-3.485276107	0.000491629	0.00449271	-1.30
FTHL20	483.954618	-0.422206688	-3.483720294	0.000494496	0.00451643	-1.34
USP31	144.0187002	-0.53470559	-3.483186946	0.000495482	0.00452297	-1.45
AIFM1	675.9935074	-0.324241567	-3.48215536	0.000497395	0.00453795	-1.25
GNB3	142.2948021	0.538477499	3.479826905	0.000501738	0.00457507	1.45
BAD	760.4989218	0.350817101	3.477770212	0.000505603	0.0046078	1.28
ARFRP1	909.078888	0.360692379	3.474617098	0.000511583	0.00465722	1.28
ZFPL1	700.731726	0.331210216	3.473523511	0.000513672	0.00466855	1.26
PRR11	115.2181865	-0.559731958	-3.473380607	0.000513946	0.00466855	-1.47
UBR5	2387.528916	-0.341453817	-3.46708517	0.000526135	0.00476932	-1.27
ITSN2	551.1498052	-0.421068108	-3.467061818	0.000526181	0.00476932	-1.34
PHF14	525.345643	-0.378730115	-3.466704655	0.00052688	0.00477048	-1.30
LRRK1	436.2428544	-0.404736472	-3.466826867	0.000526641	0.00477048	-1.32
ANAPC1	738.0317975	-0.445245447	-3.466286453	0.000527701	0.00477532	-1.36
PHIP	792.9610473	-0.353673944	-3.464281634	0.00053165	0.00480845	-1.28
ABCA6	187.9033538	-0.508408437	-3.462427003	0.000535327	0.00483386	-1.42
AURKB	33.569089	-0.764241756	-3.462509608	0.000535163	0.00483386	-1.70
RP11-523D22.1	235.9204356	-0.460777123	-3.461477935	0.000537218	0.0048457	-1.38
ATM	1566.834154	-0.353240179	-3.461286612	0.0005376	0.00484652	-1.28
VCAN	41042.50911	-0.392752639	-3.460012367	0.000540151	0.00486562	-1.31
MRPL55	326.419245	0.402294809	3.459654419	0.000540869	0.00486811	1.32
FCHSD2	1388.688896	-0.33752725	-3.458588313	0.000543014	0.00488216	-1.26
KLF13	1681.865578	-0.323032683	-3.457109731	0.000546002	0.00490111	-1.25
RP13-138P15.3	34.59925826	0.751406861	3.456462236	0.000547316	0.00490763	1.68
TMPO	478.4138689	-0.37875781	-3.453783518	0.000552781	0.00495132	-1.30

MAGI3	259.2130069	-0.441475328	-3.45360952	0.000553138	0.00495186	-1.36
RP1-102E24.9	326.6996414	0.409924527	3.453349065	0.000553672	0.00495249	1.33
TIPARP	2715.310782	0.325368785	3.453286214	0.000553801	0.00495249	1.25
TRPC4	281.9825125	-0.422730441	-3.45300678	0.000554375	0.0049547	-1.34
SPRY1	155.7805874	0.503050328	3.452545608	0.000555324	0.00495549	1.42
SUSD5	396.2217137	-0.365990515	-3.451625241	0.000557221	0.00496977	-1.29
RP11-398K22.9	785.5547948	-0.333662145	-3.451304553	0.000557884	0.00497037	-1.26
CD9	899.7345077	-0.343565441	-3.451394297	0.000557698	0.00497037	-1.27
MYL6	40189.87743	0.346512769	3.450490449	0.000559569	0.00498273	1.27
RBP4	351.0743885	0.400500467	3.44567855	0.000569628	0.0050642	1.32
NATD1	812.3577442	-0.3570454	-3.445383295	0.00057025	0.00506704	-1.28
SPSB1	277.323437	-0.420345924	-3.444143735	0.000572871	0.00508762	-1.34
AGL	728.1676193	-0.360023989	-3.441124242	0.000579302	0.00513927	-1.28
TGFBR3	570.7580582	-0.345661766	-3.438941672	0.000583993	0.00517539	-1.27
RP11-548C21.1	395.8559892	-0.409019015	-3.438622441	0.000584682	0.00517875	-1.33
C5orf42	601.1582955	-0.428802883	-3.436036216	0.000590292	0.00521264	-1.35
GMNN	93.65067078	-0.580060326	-3.435995165	0.000590382	0.00521264	-1.49
GLI3	215.9371514	-0.49007786	-3.435497149	0.000591468	0.00521948	-1.40
LIPG	322.541704	-0.391573145	-3.432041866	0.000599055	0.00527528	-1.31
LCNL1	20.20450696	-0.778362356	-3.431454102	0.000600355	0.00528394	-1.72
EDNRA	109.4826213	-0.552899072	-3.431308886	0.000600676	0.00528399	-1.47
EDF1	3787.125508	0.323292748	3.431154949	0.000601017	0.00528421	1.25
RP11-245M24.1	45.64136093	0.714359948	3.430669158	0.000602094	0.00529089	1.64
ITPKB	160.0187679	-0.485675208	-3.428922337	0.000605983	0.00532226	-1.40
RPP25	41.13847158	-0.725106894	-3.42654926	0.000611303	0.00536053	-1.65
MCM3	471.5949335	-0.370818682	-3.424224907	0.000616556	0.00539621	-1.29
PTPN13	1445.784521	-0.407835455	-3.424295887	0.000616395	0.00539621	-1.33
RP11-522H2.3	264.0527937	-0.503690724	-3.42324356	0.000618786	0.00540976	-1.42
SLC26A2	905.5625803	-0.330928725	-3.421519566	0.000622722	0.00543499	-1.26
CLN6	303.5602187	-0.403396722	-3.420316433	0.000625483	0.00545339	-1.32
MAP6	196.3329299	0.484609861	3.415612233	0.000636388	0.00553979	1.40
MPP4	69.20189126	0.640575547	3.413932781	0.000640324	0.00556101	1.56
ZCCHC11	683.220444	-0.352296411	-3.41407501	0.00063999	0.00556101	-1.28
SH3PXD2B	2743.058658	-0.37489332	-3.413862299	0.000640489	0.00556101	-1.30
FBLN2	991.4550834	-0.344275173	-3.412217079	0.000644368	0.00558597	-1.27
RP11-159D12.2	132.9080194	0.540641366	3.410233752	0.000649072	0.00562383	1.45
C9orf142	326.4780713	0.407228623	3.408985618	0.000652049	0.0056467	1.33
SLC33A1	510.1898919	0.362762828	3.406285111	0.000658534	0.005694	1.29
C12orf57	981.7213296	0.37121603	3.405912277	0.000659434	0.00569883	1.29
CSAD	324.8036868	0.463427611	3.400946529	0.00067153	0.00578839	1.38
FIGN	92.116208	-0.596861055	-3.401077415	0.000671208	0.00578839	-1.51

RP11-815J4.6	52.94797489	-0.693437456	-3.400605412	0.000672368	0.00579263	-1.62
KCNN4	533.7086755	0.343042416	3.3990651	0.000676166	0.00581635	1.27
TMEM171	99.62657788	0.584717391	3.398429784	0.000677739	0.00582092	1.50
RP11-252A24.2	74.40935607	-0.6266932	-3.398577366	0.000677373	0.00582092	-1.54
AC013394.2	580.581992	0.36267266	3.395424228	0.000685223	0.00587913	1.29
FKBP2	1429.98088	0.344878856	3.395113623	0.000686001	0.0058805	1.27
TMEM233	64.38410529	-0.639427884	-3.395079356	0.000686087	0.0058805	-1.56
FLYWCH2	500.4873764	0.370419436	3.394061248	0.000688643	0.00589937	1.29
RP11-177H22.2	131.3861923	-0.522803643	-3.393569335	0.000689881	0.00590695	-1.44
GLE1	480.9404762	-0.341360192	-3.392597238	0.000692334	0.00592491	-1.27
PDPR	1193.846042	-0.400731581	-3.390238494	0.000698318	0.00597002	-1.32
MGA	627.5958843	-0.411876916	-3.390366223	0.000697993	0.00597002	-1.33
PTN	316.711793	-0.398311476	-3.384606322	0.000712804	0.00608141	-1.32
RP11-425L10.1	4615.673167	0.337982325	3.383776238	0.000714962	0.00609671	1.26
ATP5G1	551.040915	0.36621168	3.382747193	0.000717647	0.00611295	1.29
PDRG1	489.727317	0.35929979	3.382485421	0.000718331	0.00611295	1.28
KAT7	636.8641191	-0.351597493	-3.382342214	0.000718706	0.00611302	-1.28
GRPEL1	816.5103547	0.349955432	3.381642599	0.000720538	0.00612549	1.27
KCTD17	598.5671485	0.346841478	3.381088547	0.000721993	0.00613473	1.27
CHKA	206.2568437	-0.453344548	-3.379256032	0.000726823	0.0061695	-1.37
KCNAB2	478.3775395	0.366234035	3.378627933	0.000728485	0.00617733	1.29
BPTF	1720.440678	-0.386291288	-3.374426073	0.000739698	0.00625653	-1.31
YPEL2	459.130653	-0.363248572	-3.374220405	0.000740251	0.00625804	-1.29
ZNF117	554.1628887	-0.39427572	-3.373653399	0.000741777	0.00626777	-1.31
NAMPT	623.2790691	-0.32431942	-3.372703755	0.00074434	0.0062799	-1.25
IFT172	493.0886161	-0.359618431	-3.372728947	0.000744272	0.0062799	-1.28
NUDT16L1	449.066243	0.368078679	3.372005902	0.000746228	0.00629265	1.29
ZNF330	520.5903677	-0.376776773	-3.371279122	0.0007482	0.00630498	-1.30
DBIP1	10.82389839	-0.730856437	-3.371188853	0.000748445	0.00630498	-1.66
VPS11	873.5641344	-0.328473517	-3.37083022	0.00074942	0.00631001	-1.26
RP11-12A1.1	101.4785506	-0.572819515	-3.369480189	0.000753101	0.00633462	-1.49
SHB	279.3641457	0.404870493	3.363961712	0.000768322	0.00644318	1.32
CENPE	140.8262313	-0.514439184	-3.36265622	0.000771964	0.00646723	-1.43
GPATCH8	915.9752223	-0.385922304	-3.362185166	0.000773283	0.00647503	-1.31
AKR1C2	344.3820704	-0.372064135	-3.36064098	0.000777618	0.00650807	-1.29
AQR	802.8078485	-0.346904368	-3.35904631	0.00078212	0.00653844	-1.27
UVRAG	458.272091	-0.375541092	-3.359149161	0.000781829	0.00653844	-1.30
RBM12B	332.3683569	-0.419200081	-3.358939774	0.000782421	0.00653844	-1.34
KRT17	158.7439256	0.511957687	3.358357896	0.00078407	0.00654312	1.43
SUSD1	301.3890284	0.400400223	3.353201636	0.000798825	0.00665223	1.32
MZT1	423.4647251	0.360344818	3.353023225	0.00079934	0.0066532	1.28

POLA1	162.2303878	-0.478769148	-3.352747823	0.000800136	0.00665651	-1.39
LPP	3268.388461	-0.397911134	-3.349599679	0.000809284	0.00671923	-1.32
OSGIN2	532.5333036	-0.332699001	-3.348528884	0.000812418	0.0067419	-1.26
MT1X	134.2635549	0.517485893	3.34831741	0.000813038	0.0067437	1.43
GLTSCR1L	442.0419174	-0.440296743	-3.346540146	0.000818268	0.00677362	-1.36
C1orf52	497.505609	0.34878763	3.345877391	0.000820227	0.00678306	1.27
ASF1B	46.19793552	-0.690096269	-3.344310471	0.000824874	0.00681042	-1.61
CTD-3099C6.9	31.85234924	-0.730551269	-3.343971648	0.000825882	0.00681042	-1.66
MATN2	290.8515337	-0.411096942	-3.343651523	0.000826835	0.00681415	-1.33
MIS18BP1	209.0408134	-0.455955946	-3.343171633	0.000828267	0.00682258	-1.37
CTD-2033D15.2	298.4364644	0.443004102	3.342911392	0.000829044	0.00682562	1.36
SH3BP4	4112.217301	-0.344043636	-3.339888879	0.000838119	0.00689015	-1.27
TSSC4	617.1547861	0.368520099	3.339523035	0.000839224	0.00689584	1.29
NEAT1	21681.25926	0.338723247	3.336869785	0.000847276	0.00695516	1.26
TTF2	227.9639952	-0.473415726	-3.335514316	0.000851418	0.00697545	-1.39
AC144652.1	38.03872709	-0.713302595	-3.333315463	0.000858176	0.00702048	-1.64
ANP32BP1	120.2569636	-0.529133222	-3.327117596	0.000877493	0.00715748	-1.44
RP11-443P15.2	59.20400921	-0.663707759	-3.325250761	0.00088339	0.00719504	-1.58
RP1-170D19.3	303.3541149	-0.400870122	-3.319088414	0.000903118	0.00734498	-1.32
PIKFYVE	936.863323	-0.338040872	-3.316892574	0.000910246	0.00739575	-1.26
AC002116.8	47.06425729	0.672006444	3.316072093	0.000912923	0.00741029	1.59
FBXO2	54.13788119	0.66240977	3.312549153	0.000924499	0.00747519	1.58
PA2G4P2	111.610579	-0.565745511	-3.312558376	0.000924468	0.00747519	-1.48
XYLB	116.2797238	-0.535226022	-3.312250342	0.000925487	0.00747956	-1.45
RP11-410F13.2	633.7000454	0.345917377	3.311420234	0.000928237	0.00749816	1.27
LZTS1	116.7562001	-0.549536806	-3.310600066	0.000930962	0.00751654	-1.46
TGIF1	746.4001605	-0.322399013	-3.30894225	0.000936492	0.00755024	-1.25
ZMIZ1	3459.230888	-0.354901496	-3.308345731	0.000938489	0.00756269	-1.28
FAM83D	47.58302593	-0.681832541	-3.306690149	0.000944053	0.00760019	-1.60
HLA-G	251.7318242	0.412583085	3.305936668	0.000946595	0.00761699	1.33
NHSL2	127.3895736	-0.5704574	-3.305253785	0.000948904	0.0076319	-1.48
ASMTL	522.6435427	0.341743562	3.304406202	0.000951778	0.00764765	1.27
S1PR1	60.6070422	-0.631592996	-3.301520163	0.000961624	0.00771192	-1.55
C9orf16	846.6746943	0.324111593	3.301200009	0.000962722	0.00771702	1.25
TXLNG	367.1109079	-0.359840275	-3.299204264	0.000969593	0.00775721	-1.28
RP11-186B7.4	266.3023926	0.450687169	3.297955839	0.000973915	0.0077806	1.37
WNK1	6604.706916	-0.368497873	-3.296370731	0.000979427	0.0078209	-1.29
6-Sep	246.127659	-0.432054015	-3.295486797	0.000982513	0.0078418	-1.35
TOMM7	2248.763447	0.358053656	3.293948555	0.000987906	0.00788034	1.28
GAS1	778.5246503	-0.379453665	-3.292484274	0.000993065	0.00791467	-1.30
ZNFX1	2602.27663	-0.343725285	-3.290622369	0.00099966	0.00795585	-1.27

RP11-44N21.1	25.35644573	0.738488795	3.287737006	0.001009961	0.00802519	1.67
FBXW9	176.9470985	0.460065048	3.287643991	0.001010295	0.00802519	1.38
MAPK11	323.6895146	0.39516053	3.287711843	0.001010052	0.00802519	1.32
REV3L	889.4932547	-0.388074149	-3.286370774	0.001014873	0.00805773	-1.31
CSNK2B	675.9816233	0.36088062	3.285881003	0.001016639	0.00806792	1.28
MAP1LC3A	574.6460443	0.387749482	3.283341109	0.001025845	0.00812938	1.31
ROBO4	47.87405424	0.674072344	3.282468413	0.001029025	0.008143	1.60
SRXN1	165.8915942	-0.519455564	-3.281912225	0.001031057	0.00815521	-1.43
RP11-795H16.2	107.033137	-0.575087294	-3.277172064	0.001048525	0.00827378	-1.49
RP11-54A9.1	125.9336959	0.529675182	3.276255365	0.001051934	0.00829677	1.44
DHFRL1	218.1108413	-0.427469613	-3.275427725	0.001055021	0.00831719	-1.34
TRPV2	743.9413461	0.323945126	3.27491045	0.001056955	0.0083285	1.25
HRAS	768.3933124	0.341306325	3.274142872	0.00105983	0.00834722	1.27
ZNF33B	156.93411	-0.478526259	-3.273704607	0.001061475	0.00835624	-1.39
DYSF	1090.27862	-0.329460545	-3.273489327	0.001062284	0.00835867	-1.26
CASP1	22.35431387	-0.73796785	-3.273017127	0.00106406	0.0083687	-1.67
MIR4767	23.90039214	-0.738260511	-3.272225125	0.001067046	0.00838823	-1.67
FZD2	679.5473363	-0.324155502	-3.271128167	0.001071194	0.00841292	-1.25
ST3GAL5	415.82607	0.401006716	3.269646912	0.001076818	0.00845312	1.32
IFI27L2	1011.081678	0.335081982	3.269347247	0.001077959	0.00845811	1.26
VARS2	388.3695129	0.378179383	3.268023241	0.001083015	0.00848582	1.30
BRIX1	492.0852836	-0.326742672	-3.266948633	0.001087134	0.00851011	-1.25
TM2D1	691.1613326	0.348736185	3.266551951	0.001088658	0.00851406	1.27
RPS16	4931.781573	0.333648611	3.266584379	0.001088534	0.00851406	1.26
RP3-393P12.2	167.0767464	-0.537553848	-3.262195009	0.001105531	0.00862985	-1.45
GRK6	340.4809657	0.36484491	3.261059226	0.001109969	0.00866045	1.29
TBC1D5	1275.776686	-0.376581046	-3.259019243	0.001117981	0.00871483	-1.30
SRA1	920.0356862	0.332451181	3.257999563	0.001122006	0.00873805	1.26
FAM229B	419.7849678	0.34710425	3.256235376	0.001129001	0.00878024	1.27
C6orf132	423.882034	0.340153437	3.254632316	0.001135393	0.00882174	1.27
RNF181	1202.537639	0.330744028	3.253626054	0.001139422	0.00884481	1.26
RP11-671J11.2	1248.286604	0.353989961	3.251718188	0.001147097	0.008892	1.28
SAMD14	144.5030293	0.481900301	3.248599156	0.001159748	0.00898173	1.40
RP11-434N15.1	124.5235731	-0.537271709	-3.246353369	0.001168936	0.0090445	-1.45
SLC16A13	66.68821572	-0.604228984	-3.240192301	0.001194491	0.00921236	-1.52
GJB5	18.32461271	0.737974404	3.239129453	0.001198951	0.00923823	1.67
SPACA6	132.01063	0.525486767	3.238596315	0.001201195	0.00925125	1.44
RPS27P27	448.6357876	-0.363403697	-3.236677788	0.001209299	0.00930937	-1.29
RPAP2	446.5567241	-0.349582108	-3.235460517	0.001214467	0.00934055	-1.27
RP11-280G19.2	219.7597786	-0.430885325	-3.235507027	0.001214269	0.00934055	-1.35
CTD-2554C21.1	300.6349087	-0.383283739	-3.234961107	0.001216593	0.00935259	-1.30

CCDC188	49.81904946	0.646321894	3.234635121	0.001217983	0.00935897	1.57
BCLAF1P2	441.115601	-0.351955434	-3.231400989	0.00123185	0.00945248	-1.28
PXYLP1	242.715273	0.405799118	3.228939363	0.001242502	0.0095222	1.32
KLF5	182.7681532	-0.445880341	-3.228935698	0.001242518	0.0095222	-1.36
RP11-76P2.3	60.48347364	-0.625966155	-3.228906426	0.001242645	0.0095222	-1.54
PAK1IP1	345.2689833	-0.36181648	-3.226187145	0.001254513	0.00959993	-1.29
SMC6	514.8468115	-0.340443668	-3.225280785	0.001258492	0.00962156	-1.27
PLD1	143.3084622	-0.477136165	-3.225323028	0.001258306	0.00962156	-1.39
CDK1	84.74172723	-0.583229872	-3.224033569	0.001263986	0.00965472	-1.50
NGF	386.218002	0.350877755	3.222654173	0.001270088	0.00969247	1.28
PDP2	455.0744372	-0.332131418	-3.222415211	0.001271148	0.00969613	-1.26
CPS1	211.5173373	-0.438637632	-3.221935466	0.001273278	0.00970795	-1.36
RP13-88F20.1	620.4204149	-0.378812067	-3.221442185	0.001275472	0.00972019	-1.30
PBX3	348.8262765	-0.358250868	-3.218105696	0.001290403	0.00982058	-1.28
OLAH	20.9559282	0.731548067	3.216402576	0.001298086	0.00987456	1.66
NDRG4	157.1234996	0.462886547	3.216057281	0.001299649	0.00987745	1.38
PLEKHA4	730.8146652	-0.337762531	-3.215257347	0.001303276	0.00990051	-1.26
KCNK3	54.07888479	0.646534355	3.21445789	0.001306911	0.00991634	1.57
PRR7	279.8890148	0.464511211	3.21454711	0.001306505	0.00991634	1.38
ACACB	109.5839784	-0.516254901	-3.214407323	0.001307141	0.00991634	-1.43
TGFBRAP1	1023.111931	-0.360733077	-3.213180131	0.001312739	0.00995429	-1.28
AL353997.7	121.0405112	-0.550275419	-3.212772266	0.001314605	0.00996392	-1.46
CTB-67I13.1	422.8442699	0.407416578	3.211978368	0.001318243	0.00998313	1.33
SPRED3	426.170391	0.336155631	3.211958302	0.001318335	0.00998313	1.26
RP11-234N17.1	64.35407629	0.620656997	3.211381091	0.001320986	0.00999867	1.54
SPSB2	158.5228481	-0.462179696	-3.209437994	0.001329947	0.01005284	-1.38
RP11-705B14.1	229.5809708	-0.44412903	-3.208212007	0.00133563	0.01008666	-1.36
RP11-114F3.2	181.8477362	-0.446651615	-3.20783668	0.001337374	0.01009527	-1.36
RP6-99M1.2	273.7535923	0.46424409	3.205720129	0.00134725	0.01015605	1.38
STAC	618.4347629	0.330659066	3.205437769	0.001348572	0.01016143	1.26
RP11-473O3.1	155.4115602	-0.49113249	-3.203728999	0.001356601	0.01020812	-1.41
VPRBP	580.0686858	-0.326176357	-3.20356009	0.001357397	0.01020951	-1.25
HMGB3	277.5467971	-0.400177427	-3.202866886	0.001360669	0.01022951	-1.32
HABP4	457.5515296	-0.336969013	-3.201433714	0.001367455	0.01027129	-1.26
MYEOV2	873.014774	0.347857417	3.198102469	0.001383351	0.01037669	1.27
RP11-234P3.3	97.90030357	-0.532842814	-3.197930657	0.001384176	0.01037821	-1.45
ALKBH8	198.052221	-0.430371172	-3.19674412	0.001389882	0.01041632	-1.35
AC068057.2	45.59632722	0.671108899	3.196568058	0.00139073	0.010418	1.59
MINOS1	775.0784143	0.349283807	3.193038404	0.001407842	0.01053202	1.27
SHPRH	322.5199367	-0.404330033	-3.191763896	0.001414069	0.01057387	-1.32
TMEM91	129.5253499	0.498838587	3.187218022	0.001436484	0.01070793	1.41

SLC29A4	98.9099127	0.528259442	3.186849406	0.001438316	0.0107168	1.44
SLC9A5	199.6617495	0.419501807	3.184560724	0.001449739	0.01078268	1.34
STRA13	352.6922358	0.38789049	3.179885653	0.001473332	0.0109484	1.31
MAPK8IP1	420.6753665	0.331186227	3.179699078	0.001474281	0.01095058	1.26
GUF1	461.5316362	-0.333680493	-3.178678972	0.001479479	0.01097455	-1.26
LBX2-AS1	49.71268505	-0.633763691	-3.177348452	0.001486283	0.01101036	-1.55
AC007238.1	467.7307523	-0.329034903	-3.176532064	0.001490473	0.01103161	-1.26
PLEKHH2	281.2521489	-0.434765729	-3.176625435	0.001489993	0.01103161	-1.35
IBSP	18.63243794	0.722044666	3.175503723	0.001495766	0.01106099	1.65
SIK2	934.4691994	-0.331465635	-3.175182658	0.001497422	0.01106344	-1.26
RTN4RL1	114.7135626	-0.504112512	-3.17518351	0.001497418	0.01106344	-1.42
CRYBG3	818.9787602	-0.361126691	-3.172744595	0.001510053	0.01115183	-1.28
KIF4A	63.53496069	-0.600593325	-3.172483979	0.001511409	0.01115691	-1.52
RP11-305E17.5	164.1396598	-0.504973375	-3.171053372	0.001518872	0.0112021	-1.42
MRPL20	932.1521049	0.334547636	3.168413524	0.001532733	0.01129436	1.26
MSH2	449.3873057	-0.323371117	-3.168067781	0.001534557	0.01130281	-1.25
CEND1	26.83778595	0.70474555	3.166191164	0.001544493	0.01136597	1.63
CIDECP	371.8262027	0.349918195	3.162988691	0.001561584	0.01146863	1.27
ZNF692	292.8258636	0.391658599	3.160637424	0.001574243	0.01154929	1.31
PLSCR1	215.6895705	-0.423136672	-3.159476169	0.00158053	0.01158632	-1.34
UBOX5	205.4438669	0.417749475	3.156721443	0.001595537	0.01167359	1.34
TMEM256	209.4361693	0.447040757	3.152583857	0.001618323	0.01180704	1.36
RP11-114M5.1	113.6228063	-0.534023885	-3.152540392	0.001618564	0.01180704	-1.45
EMC9	180.4009501	0.454487738	3.151615074	0.001623702	0.01183934	1.37
SERTAD1	584.5734315	0.334278717	3.150401005	0.001630465	0.01187829	1.26
BOC	372.4006905	0.337232409	3.149925093	0.001633123	0.01188936	1.26
MIR34AHG	236.5001377	0.443001427	3.149147971	0.001637473	0.01190901	1.36
NFKB1	654.6812562	-0.338592275	-3.148192705	0.001642834	0.01193716	-1.26
USMG5P1	336.6542002	0.39693852	3.147668614	0.001645782	0.01195338	1.32
CECR5	464.8033418	0.327117748	3.147427137	0.001647142	0.01195806	1.25
PHKG2	247.5928435	0.414429219	3.145638394	0.001657248	0.0120262	1.33
GSTM2	230.5189036	0.410981295	3.143009349	0.001672205	0.01212421	1.33
NARFL	340.6790857	0.349066803	3.141353703	0.001681688	0.01218768	1.27
RP11-317N8.2	167.2422207	-0.438173548	-3.139007973	0.001695208	0.01227501	-1.35
HSCB	163.5357407	0.460094091	3.138861487	0.001696056	0.01227583	1.38
RP11-418J17.1	118.7317528	-0.491419505	-3.13351359	0.001727269	0.01248012	-1.41
CLCN4	192.8097442	-0.447182702	-3.129965514	0.001748268	0.01262093	-1.36
N4BP2	121.9596286	-0.498114627	-3.128710134	0.001755754	0.0126506	-1.41
WASH3P	47.16052486	0.66147193	3.127331246	0.001764011	0.0126907	1.58
EZH2	75.03712135	-0.563575979	-3.12651652	0.001768906	0.01272043	-1.48
HSPBAP1	67.75012922	-0.594293003	-3.125398901	0.001775641	0.01275788	-1.51

HIN1L	85.06131338	-0.584972739	-3.123887756	0.001784785	0.01281807	-1.50
ZNF480	287.8972349	-0.415931311	-3.121803716	0.001797467	0.01288918	-1.33
ZNF469	1021.975786	-0.384891589	-3.121512549	0.001799246	0.01289421	-1.31
POLR1B	337.1573176	-0.41641334	-3.120211555	0.001807212	0.01294212	-1.33
PIP5KL1	71.63087596	0.581654751	3.119446162	0.001811914	0.01296732	1.50
AP001062.7	160.9820107	0.444090299	3.118258662	0.001819231	0.01300952	1.36
JADE3	231.3112064	-0.411601777	-3.117898991	0.001821452	0.01301754	-1.33
KIF2C	63.54194144	-0.626049149	-3.117535078	0.001823702	0.01302478	-1.54
FOXN2	312.7929808	-0.393756506	-3.114330904	0.001843626	0.01315582	-1.31
MED10	574.9079841	0.342618141	3.113728743	0.001847392	0.01317707	1.27
ARPC1A	761.4751614	0.332070426	3.112168827	0.001857183	0.01323571	1.26
CD200	64.03149503	0.609242397	3.111441721	0.001861762	0.01326257	1.53
RP5-1119A7.11	39.29212803	-0.670176421	-3.11053325	0.001867499	0.01329777	-1.59
KIF20B	117.0930541	-0.496234094	-3.110304742	0.001868944	0.01330239	-1.41
ATP5I	1518.445561	0.325710826	3.107868105	0.001884421	0.01338973	1.25
IL16	114.611037	-0.503498056	-3.107542478	0.001886499	0.01339879	-1.42
EGR2	132.4525179	-0.481023018	-3.106502141	0.001893149	0.01343461	-1.40
SNRPA1	175.5979963	0.454057315	3.10558208	0.001899049	0.01346529	1.37
INTS2	227.3895602	-0.411335628	-3.102660377	0.001917896	0.01357562	-1.33
DHRS1	402.9409194	0.338928315	3.101434024	0.001925858	0.01361467	1.26
LRBA	904.2461815	-0.364345776	-3.101469185	0.001925629	0.01361467	-1.29
TLL2	137.4991199	0.463297123	3.10121559	0.001927279	0.01361895	1.38
SLC43A1	184.7007427	-0.45226684	-3.1010715	0.001928217	0.01361982	-1.37
KIAA0355	945.4261795	-0.325907286	-3.100037833	0.001934959	0.01366166	-1.25
ANKRD50	1074.246604	-0.35473854	-3.099427978	0.001938947	0.01368404	-1.28
ACP5	15.61474275	-0.701206983	-3.099287853	0.001939864	0.01368473	-1.63
TRIT1	382.0484657	0.34722473	3.097651488	0.001950607	0.01374601	1.27
RP11-144L1.2	292.9362972	-0.362436249	-3.097557915	0.001951223	0.01374601	-1.29
TROAP	32.51646544	-0.682382208	-3.096755661	0.001956511	0.01375569	-1.60
RP11-231C14.3	113.9893161	-0.533287229	-3.096288224	0.001959598	0.0137716	-1.45
QSER1	984.404908	-0.346321612	-3.095944501	0.001961871	0.01377598	-1.27
RP11-351I24.3	218.2497677	0.460376621	3.095198456	0.001966812	0.01379908	1.38
ZNF521	71.04886968	-0.5876998	-3.094848826	0.001969132	0.01380955	-1.50
RP11-627G18.3	35.84238902	0.663274483	3.087122435	0.002021043	0.01413799	1.58
CTD-2287O16.1	1743.233798	0.323210716	3.086351386	0.002026292	0.01416878	1.25
SH3RF3	264.122347	-0.426416337	-3.085653921	0.002031051	0.01419611	-1.34
ZNF611	137.5308088	-0.465472531	-3.084518065	0.002038822	0.01423542	-1.38
BCL2L11	94.90417635	-0.528005692	-3.083272045	0.002047379	0.01426845	-1.44
CKAP2L	58.33461836	-0.59528884	-3.082633832	0.002051774	0.01429312	-1.51
ING4	455.4396102	0.323551801	3.081045342	0.002062752	0.01435762	1.25
ZFAND2B	477.6402617	0.357482999	3.080531189	0.002066317	0.01437644	1.28

USP13	335.3742426	-0.367518259	-3.080358205	0.002067518	0.0143788	-1.29
NTF3	92.3759659	0.54652416	3.07772933	0.002085843	0.01448797	1.46
ZNF827	789.7815545	-0.326354381	-3.076749459	0.002092711	0.01451775	-1.25
ZNF407	349.0974534	-0.393661664	-3.076293293	0.002095916	0.01453394	-1.31
RP11-304L19.5	57.86725744	0.609381064	3.074766075	0.002106677	0.01460251	1.53
GPER1	150.7928773	0.44562029	3.073128076	0.002118276	0.01467073	1.36
TNRC6C	256.3481878	-0.396262242	-3.072812464	0.002120517	0.01468016	-1.32
RP11-274E7.2	154.7387114	0.461638121	3.071653051	0.00212877	0.01472509	1.38
MSNP1	1268.717772	-0.330074724	-3.071203146	0.00213198	0.0147412	-1.26
FANK1	295.2401292	0.371145487	3.070701009	0.002135569	0.0147538	1.29
ATXN1L	908.231649	-0.327994333	-3.065495349	0.002173098	0.01499446	-1.26
ABCB9	255.8105475	0.379245475	3.06456094	0.002179898	0.01502385	1.30
TRAPPC10	708.3164123	-0.322203885	-3.064680501	0.002179026	0.01502385	-1.25
PCSK5	403.6759566	0.340881391	3.063862543	0.002184993	0.01505167	1.27
EPB41L4B	79.03214277	0.545266808	3.062307287	0.002196379	0.01512387	1.46
FBLN7	281.5812581	0.386115882	3.062157419	0.002197479	0.01512521	1.31
SPSB3	388.3115333	0.356003414	3.060617897	0.002208808	0.01517774	1.28
FBXL15	222.7892651	0.438548036	3.059910643	0.00221403	0.01520155	1.36
FAM217B	155.6999854	-0.446909466	-3.059404737	0.002217773	0.01521474	-1.36
ZNF507	430.9165177	-0.350385274	-3.05707213	0.002235105	0.0153085	-1.27
NAP1L5	249.1027439	0.399203414	3.054719459	0.002252711	0.01541013	1.32
EIF5AP4	178.0427236	0.45594213	3.054009216	0.002258051	0.01543402	1.37
NDUFA2	714.26269	0.328283287	3.05310486	0.002264868	0.01547428	1.26
PEAK1	4940.791319	-0.352646523	-3.052917909	0.002266279	0.01547759	-1.28
KIF16B	328.8840706	-0.343478824	-3.052516611	0.002269312	0.01549197	-1.27
ECHDC2	300.56443	0.369422218	3.052150515	0.002272081	0.01550454	1.29
SEPP1	103.1935148	-0.511671481	-3.04956084	0.002291762	0.01560768	-1.43
RPL36P2	72.7143956	-0.559647998	-3.048993899	0.002296091	0.01562371	-1.47
ADAMTSL1	4273.999105	0.355752371	3.046078759	0.002318471	0.01576315	1.28
EXOSC4	270.7967486	0.377243028	3.044857705	0.002327904	0.01581633	1.30
TGFBR3L	30.79385854	0.670220415	3.044146396	0.002333416	0.01584541	1.59
ZKSCAN8	854.6268821	-0.340660258	-3.041862165	0.002351195	0.01593376	-1.27
SHROOM3	305.8927575	0.380058019	3.040635749	0.002360792	0.01598583	1.30
RREB1	590.996973	-0.361700695	-3.037940115	0.002382013	0.01612298	-1.28
F2RL2	295.3968595	-0.369854813	-3.036562091	0.002392928	0.01618375	-1.29
MGAM	162.4394124	0.473817206	3.033281005	0.002419102	0.01634092	1.39
FMO4	38.01663927	-0.646669959	-3.033153845	0.002420122	0.0163412	-1.57
RNF208	55.88540567	0.614770639	3.031569322	0.00243286	0.0164007	1.53
RP11-560O15.1	36.11121224	-0.658621739	-3.027557663	0.002465387	0.01659319	-1.58
AC093673.5	286.922728	0.367623655	3.027228902	0.00246807	0.01660455	1.29
ARFGEF2	1692.733302	-0.339452926	-3.025864733	0.002479232	0.01666953	-1.27

FMO3	37.1597785	-0.640242702	-3.025804701	0.002479724	0.01666953	-1.56
SYT12	380.8134227	0.335155432	3.024984407	0.002486459	0.01670809	1.26
TENM3	1264.4761	-0.378936488	-3.023806321	0.002496162	0.01676654	-1.30
ARID1B	1189.806274	-0.344010984	-3.021671733	0.00251383	0.01687165	-1.27
DLG3	244.1584661	-0.393686204	-3.018432775	0.002540858	0.01702568	-1.31
RP11-280F2.2	292.9909965	0.388452284	3.017582617	0.002547996	0.01705983	1.31
PPP1R14C	52.29533631	-0.603305282	-3.017317761	0.002550223	0.0170679	-1.52
C14orf179	441.3439138	0.335790789	3.015411284	0.00256631	0.01716181	1.26
RP4-773A18.4	145.0388115	0.443935392	3.014406272	0.002574828	0.01719812	1.36
C1orf112	62.1393978	-0.570955493	-3.012926907	0.002587412	0.01726147	-1.49
SNHG7	445.0275596	0.32430946	3.011730475	0.002597631	0.01731581	1.25
C4orf46	234.4578531	-0.379745066	-3.011112099	0.002602927	0.01732904	-1.30
PDGFD	45.37190413	-0.616813811	-3.008607954	0.002624475	0.01745992	-1.53
MANSC1	292.8413313	0.359488736	2.997115817	0.002725471	0.01805274	1.28
BLOC1S1	333.9209317	0.385002931	2.995907624	0.002736293	0.01811005	1.31
RP11-12I24.2	66.49935173	-0.565257338	-2.995635144	0.002738739	0.01811387	-1.48
NOTCH2NL	59.83898278	0.610453096	2.994121758	0.002752361	0.01817819	1.53
RP11-366L20.2	41.16389549	-0.630125004	-2.991728381	0.00277403	0.01829458	-1.55
RP11-309I15.2	394.3898537	-0.365982163	-2.991229113	0.002778569	0.01831006	-1.29
AKAP1	329.9995218	-0.355281504	-2.991042432	0.002780269	0.01831403	-1.28
AC006026.9	97.45686034	-0.5204898	-2.987820171	0.002809748	0.01849363	-1.43
PRKCH	49.58159906	0.602654921	2.987472682	0.002812944	0.01850461	1.52
PIGF	296.8784303	0.368522985	2.987398107	0.002813631	0.01850461	1.29
LY96	432.0428566	-0.36380732	-2.985023934	0.002835561	0.01862684	-1.29
RP11-517I3.2	276.6045838	0.354492734	2.984250434	0.00284274	0.01865199	1.28
UHRF1BP1	598.4908151	-0.393228666	-2.983657827	0.002848251	0.01867348	-1.31
CREBBP	1516.562975	-0.368674318	-2.983087841	0.002853561	0.01870095	-1.29
AC125232.1	80.82430158	0.537097829	2.981598015	0.002867482	0.01878482	1.45
KHDRBS3	111.2972135	-0.48139903	-2.980140412	0.002881163	0.0188443	-1.40
CCDC68	113.9094036	-0.484688978	-2.980446175	0.002878288	0.0188443	-1.40
RP11-123M21.2	35.65536668	-0.645859922	-2.980272054	0.002879925	0.0188443	-1.56
WDR76	52.60621838	-0.585362295	-2.979602602	0.002886226	0.01886322	-1.50
BMP2K	299.8169055	-0.428034291	-2.979365618	0.002888459	0.01887044	-1.35
POLD4	335.3979993	0.336349547	2.978338024	0.002898162	0.01891427	1.26
FRZB	37.30807132	-0.641737293	-2.973555904	0.002943708	0.0191714	-1.56
TCF4	1141.012363	-0.323861236	-2.973431649	0.0029449	0.01917169	-1.25
RP11-114G1.1	32.40814311	-0.650059711	-2.972896565	0.002950038	0.01919766	-1.57
SNHG8	1004.561967	0.328407487	2.969581756	0.002982054	0.01937323	1.26
RP11-124N14.3	16.31306056	-0.669427551	-2.969349992	0.002984305	0.01937536	-1.59
FZD5	72.47321339	-0.551792131	-2.967975394	0.002997683	0.01942202	-1.47
PALB2	246.606294	-0.372547414	-2.966605301	0.003011072	0.01948856	-1.29

ARHGEF12	4990.206125	-0.324225405	-2.96496003	0.003027223	0.01957034	-1.25
TNFRSF25	77.37671869	0.537906612	2.964315935	0.003033567	0.01960345	1.45
RXFP1	85.13386882	0.523734748	2.963474962	0.003041868	0.01962839	1.44
USP54	216.0762268	-0.434490936	-2.963509927	0.003041523	0.01962839	-1.35
XPO4	524.130911	-0.334947446	-2.962207827	0.003054415	0.01969279	-1.26
DOCK3	95.34315121	-0.495248011	-2.959953723	0.003076853	0.01980687	-1.41
CARD10	127.3772596	0.46078378	2.958944583	0.003086946	0.01984934	1.38
PPP1R14A	191.7048854	0.426189828	2.953851057	0.003138354	0.02015617	1.34
ASPN	96.7453717	-0.490739286	-2.95344849	0.00314245	0.02017089	-1.41
GRB14	62.7280755	0.559659287	2.95281315	0.003148925	0.02020077	1.47
YRDC	360.5040284	0.323539654	2.952532387	0.00315179	0.02020363	1.25
NSUN5P2	220.4527759	0.477982627	2.951167639	0.00316575	0.02027755	1.39
CTC-444N24.11	196.2346374	0.484749682	2.948005259	0.003198316	0.0204783	1.40
CST6	80.90908584	0.530485098	2.946764126	0.00321118	0.02054491	1.44
TACC2	221.1426353	-0.377000026	-2.945979773	0.003219335	0.02057344	-1.30
PIK3C2A	2023.923241	-0.333533894	-2.945371822	0.003225668	0.02059816	-1.26
SMAD9	286.8608299	-0.364956798	-2.942516662	0.003255563	0.02076524	-1.29
AC019178.2	65.721554	-0.55036006	-2.942074025	0.00326022	0.02077944	-1.46
SLC15A3	53.34528284	-0.59676521	-2.942068718	0.003260276	0.02077944	-1.51
P2RX6	289.4202861	0.363977953	2.938406705	0.003299039	0.02100246	1.29
RP11-529E10.6	27.16456168	0.655779529	2.938284733	0.003300338	0.02100272	1.58
TTLL4	225.0935795	-0.392680001	-2.937901552	0.003304419	0.02100469	-1.31
SLC45A1	94.40339094	0.504735186	2.93474641	0.003338204	0.02117723	1.42
AK4P1	407.0981081	0.350346041	2.934592347	0.003339862	0.02117723	1.27
MAPRE2	443.6543678	-0.340648863	-2.933811785	0.003348272	0.02121885	-1.27
LIN7B	123.4083669	0.506042741	2.930347999	0.003385826	0.02142433	1.42
LIMD2	133.6622759	0.445030179	2.93004801	0.003389096	0.0214369	1.36
ARHGEF11	1165.391668	-0.329712958	-2.929836917	0.003391399	0.02144335	-1.26
RP11-829H16.2	140.2557697	-0.500357438	-2.929085135	0.003399613	0.02146921	-1.41
USP24	1155.304749	-0.360498883	-2.92885407	0.003402141	0.02146989	-1.28
TLN2	807.5057049	-0.352385048	-2.927779392	0.003413922	0.02152871	-1.28
MFSD7	63.3128393	0.557913187	2.925604755	0.003437874	0.02165524	1.47
STON1	428.5670342	-0.327649743	-2.925036739	0.003444156	0.02165716	-1.25
TOP1MT	225.0450926	-0.383866325	-2.925266772	0.00344161	0.02165716	-1.30
SLC12A7	207.3644793	-0.39827072	-2.924991011	0.003444662	0.02165716	-1.32
PI16	63.68724121	-0.56451482	-2.923444857	0.003461815	0.02175682	-1.48
PYCRL	330.2668362	0.338568764	2.922564668	0.003471615	0.02179478	1.26
RP11-802E16.3	51.27046611	0.598743551	2.91917405	0.003509602	0.02201574	1.51
CTD-2552B11.4	87.35296243	0.504486214	2.918243978	0.003520088	0.02207323	1.42
FAM195A	219.1644075	0.421348531	2.916741383	0.003537089	0.02215488	1.34
PRDM10	230.3710615	-0.366957661	-2.916471653	0.003540149	0.02216574	-1.29

CORO6	216.0578869	0.381284204	2.91442118	0.003563488	0.02227004	1.30
TOGARAM1	484.4748077	-0.351581757	-2.913913299	0.00356929	0.02228972	-1.28
EXOSC9	321.2162002	-0.336602524	-2.912405325	0.003586569	0.02237253	-1.26
PRKCZ	52.08897357	0.595423298	2.911862824	0.003592804	0.02240306	1.51
RP11-58B17.1	209.5155771	0.404927081	2.907640008	0.003641673	0.02264017	1.32
CDYL2	347.4996513	-0.323990117	-2.906643598	0.003653292	0.02270396	-1.25
UCHL3	253.3852188	0.418783851	2.905919057	0.003661762	0.02274813	1.34
SLC25A42	283.4145051	0.365779151	2.905220514	0.003669944	0.02276069	1.29
CDCA7L	217.0908231	-0.378236422	-2.905500909	0.003666658	0.02276069	-1.30
ZFPM2	112.6885141	-0.495625124	-2.905164996	0.003670595	0.02276069	-1.41
MPC1	363.4175787	0.332198599	2.904196097	0.003681975	0.02282278	1.26
CMKLR1	86.19268292	-0.521966652	-2.902510953	0.003701842	0.02291192	-1.44
CRTC3	931.57327	-0.334178167	-2.901151917	0.003717936	0.02299449	-1.26
EP400	1097.73976	-0.337217402	-2.899996518	0.003731668	0.02305381	-1.26
MSR1	44.60520033	0.609974783	2.899202792	0.003741128	0.02310372	1.53
CCDC69	207.6251584	-0.384271925	-2.898179658	0.003753355	0.02312792	-1.31
MED12	663.2450602	-0.388759252	-2.898283525	0.003752112	0.02312792	-1.31
MIPOL1	86.55771893	-0.514197263	-2.892131448	0.003826378	0.02349123	-1.43
DMXL2	616.3900399	-0.356219955	-2.890867649	0.003841799	0.02356858	-1.28
RP1-193M11.1	183.61853	-0.417991268	-2.889855707	0.003854187	0.02361856	-1.34
CEACAM19	126.4502699	0.457925072	2.88886712	0.003866324	0.02367557	1.37
CEP162	130.9155494	-0.437908246	-2.885881856	0.003903186	0.02385685	-1.35
RHOQP1	88.15120269	-0.523835271	-2.885476877	0.003908212	0.02386209	-1.44
AFMID	397.8299684	0.362904595	2.884509839	0.003920235	0.02392675	1.29
ZNF81	136.255911	-0.528082832	-2.883564087	0.003932026	0.02397452	-1.44
ARAP3	89.11162935	0.512570993	2.883122477	0.003937543	0.02399732	1.43
VWCE	46.5223103	0.58526341	2.882489747	0.00394546	0.0240368	1.50
RP11-467I20.3	204.7821148	0.47440063	2.8811578	0.003962172	0.02412102	1.39
FGFR2	226.1867513	-0.409899764	-2.879123425	0.003987822	0.02424184	-1.33
TLE2	78.2479423	-0.523165312	-2.878694733	0.003993247	0.02426598	-1.44
RP11-325E14.3	449.9745961	-0.378778566	-2.877711786	0.004005709	0.02433286	-1.30
RP1-159M24.1	114.6926231	-0.46274946	-2.875986625	0.004027668	0.02443959	-1.38
UBE2C	68.17717662	-0.54703537	-2.875471486	0.004034246	0.02446173	-1.46
IFI44	251.5256634	-0.361693366	-2.874406257	0.004047879	0.0245177	-1.28
ULK3	334.6385132	-0.324774811	-2.874135196	0.004051355	0.02452986	-1.25
PBK	59.61638847	-0.559578836	-2.871681264	0.004082945	0.02467641	-1.47
NUP214	820.7434924	-0.353728037	-2.871158879	0.004089699	0.02469936	-1.28
TRERF1	181.3626917	-0.421348034	-2.869970462	0.004105101	0.02477447	-1.34
bP-21264C1.3	13.8656008	0.641036276	2.869433656	0.004112076	0.02480759	1.56
RP11-90H3.1	284.0510901	-0.34467759	-2.86886726	0.004119446	0.02484309	-1.27
ARHGAP32	454.0469831	-0.36778417	-2.868658814	0.004122162	0.02485049	-1.29

LMNTD2	29.04152024	0.634837667	2.865387827	0.004164988	0.02502738	1.55
EDARADD	337.7788127	0.322205188	2.865265678	0.004166595	0.02502804	1.25
MRPS6	556.0043682	0.32501276	2.860747212	0.004226439	0.02536637	1.25
FGD1	501.4268121	-0.329767149	-2.860669487	0.004227475	0.02536637	-1.26
RP11-569O4.5	65.71742478	0.554673681	2.85952688	0.004242735	0.02543053	1.47
GRASP	20.80478069	-0.64578734	-2.859217943	0.004246869	0.02544618	-1.56
RP4-765A10.1	262.2728498	-0.385078849	-2.858181352	0.004260768	0.02551116	-1.31
CNTRL	79.86951659	-0.504330477	-2.855958106	0.004290717	0.0256537	-1.42
CARD16	9.22876157	-0.587666927	-2.855047009	0.004303046	0.02570159	-1.50
PLXNA4	534.7153739	-0.376219987	-2.85485035	0.004305711	0.02570654	-1.30
MED13	1771.552274	-0.336998568	-2.848123539	0.004397785	0.02621877	-1.26
RP11-982M15.6	20.2832421	-0.642890135	-2.846005212	0.004427147	0.02636559	-1.56
KCNT2	90.15518182	0.519176251	2.845429416	0.004435159	0.0263903	1.43
GEMIN7	255.0214084	0.389545194	2.845366557	0.004436034	0.0263903	1.31
CTNNBIP1	364.5910682	0.326767319	2.843845067	0.004457272	0.0265072	1.25
SERP2	50.91078916	0.569701342	2.840494572	0.004504364	0.02673016	1.48
DAAM2	1131.66534	-0.343614757	-2.840223339	0.004508196	0.02674108	-1.27
CADM4	48.69922483	0.580955712	2.839375605	0.004520192	0.02677646	1.50
TRBV12-4	10.03214607	0.617039756	2.838649565	0.004530488	0.026809	1.53
PPP1R32	64.26301473	0.533306089	2.837917567	0.004540891	0.02686104	1.45
NDC80	51.63315766	-0.560104879	-2.83716181	0.004551654	0.02691518	-1.47
STXBP4	302.7167512	-0.357997341	-2.83615409	0.004566041	0.02698115	-1.28
ZNF234	242.3956807	-0.34880649	-2.835551557	0.004574663	0.02702254	-1.27
ARL16	426.1669691	0.354068562	2.833667702	0.004601716	0.02714395	1.28
ACD	259.1257525	0.340878689	2.833361187	0.004606131	0.02715082	1.27
ACTC1	137.7863884	-0.429766044	-2.832088617	0.004624503	0.02722069	-1.35
RIPK3	41.90253154	0.588896837	2.831151578	0.004638074	0.02727174	1.50
GDAP1	92.59702189	-0.483427591	-2.828648888	0.004674495	0.02744726	-1.40
TWIST1	260.2471775	-0.346199786	-2.828226939	0.004680661	0.02747255	-1.27
BRWD3	276.6019798	-0.375798991	-2.827976498	0.004684325	0.027476	-1.30
PCDHB13	122.553162	-0.451939104	-2.824513162	0.00473525	0.02774548	-1.37
PARL	194.2224119	0.41275057	2.823156198	0.00475534	0.0278339	1.33
ADAMTS15	113.9945206	0.462510379	2.821837834	0.004774932	0.02791696	1.38
PCLAF	41.99393589	-0.589371687	-2.821791963	0.004775615	0.02791696	-1.50
PIR	289.4320635	-0.339016177	-2.821268432	0.004783416	0.0279395	-1.26
CENPI	52.13161282	-0.562493607	-2.819380549	0.004811644	0.02807492	-1.48
ITGB3BP	135.9017405	0.462204603	2.81925145	0.004813579	0.02807641	1.38
STRBP	131.2480812	-0.436875977	-2.817498059	0.004839941	0.02822032	-1.35
CTD-3035D6.1	174.2676053	-0.410779091	-2.816344663	0.004857353	0.02830208	-1.33
ATG4D	271.0782054	0.337214293	2.815790008	0.004865746	0.0283411	1.26
PRDM16	193.7888898	-0.390351532	-2.81542302	0.004871307	0.02835372	-1.31

ABCA3	302.5656012	0.332143894	2.815196623	0.00487474	0.02836382	1.26
TNFSF9	129.6430471	-0.440688616	-2.809354663	0.004964093	0.02884354	-1.36
RP11-64P14.6	119.48486	-0.46088092	-2.808671793	0.004974633	0.02888469	-1.38
AC109631.1	17.30209751	-0.627686953	-2.808432175	0.004978337	0.02889615	-1.55
VPS33A	285.5589375	-0.330887199	-2.80766287	0.004990244	0.0289552	-1.26
PRR15	90.7145671	0.477039424	2.804702257	0.005036308	0.02920219	1.39
AC002480.3	164.9637817	0.405017174	2.804775182	0.005035169	0.02920219	1.32
AC069513.3	235.667943	0.356206269	2.804002729	0.005047248	0.02925547	1.28
RP11-705C15.1	50.2475108	-0.557063849	-2.801825168	0.00508144	0.0293823	-1.47
FDPSL5	46.1033973	-0.576882228	-2.800001528	0.005110236	0.02950795	-1.49
ZNF697	355.7129343	-0.357531824	-2.79933866	0.00512074	0.02955788	-1.28
DDIAS	36.67521241	-0.607318444	-2.799232559	0.005122423	0.02955788	-1.52
DLX3	127.0800393	-0.439736479	-2.798836323	0.005128713	0.02958396	-1.36
SNHG15	148.0778872	0.424590519	2.797784038	0.00514545	0.02967026	1.34
RGS7	102.8512472	0.51107228	2.796127161	0.005171905	0.02979195	1.43
MIPEP	306.1223539	-0.324904976	-2.795618848	0.005180045	0.02981828	-1.25
CARD6	360.768444	-0.328391417	-2.795098168	0.005188396	0.02985606	-1.26
MZF1	332.810049	0.347696119	2.794642854	0.005195708	0.02987756	1.27
AC099540.1	90.90706122	-0.507556845	-2.793384526	0.005215965	0.02997341	-1.42
LRIG2	343.0007401	-0.342660649	-2.792849384	0.005224601	0.03001271	-1.27
COX10	203.5065103	-0.366812278	-2.792533297	0.005229708	0.03003172	-1.29
CHAF1A	205.4123619	-0.371456868	-2.792341368	0.005232812	0.03003922	-1.29
KRTAP4-9	10.92122871	0.612466532	2.791188417	0.005251489	0.03013608	1.53
RP11-206F17.2	179.7663046	-0.399347059	-2.79047803	0.005263027	0.03019192	-1.32
STARD8	67.74193875	-0.522039122	-2.790028466	0.005270341	0.0302235	-1.44
THEMIS2	31.34532519	-0.607510861	-2.787785584	0.005306966	0.03040222	-1.52
NSMCE2	295.2569195	-0.341253342	-2.786303619	0.005331291	0.03051377	-1.27
IFI27L1	336.5742716	0.371182345	2.784845483	0.005355324	0.03062673	1.29
RP11-125H8.1	67.01587911	-0.53164368	-2.783362109	0.005379873	0.03074607	-1.45
ZBTB43	509.8378895	-0.337291113	-2.772870525	0.005556422	0.03156071	-1.26
RP11-34P1.2	357.5755364	-0.357495178	-2.772435319	0.005563858	0.0315922	-1.28
FAM173A	204.408065	0.373404328	2.771889587	0.005573194	0.03162666	1.30
ANKRD36	59.28498854	-0.57091269	-2.771748678	0.005575607	0.03162666	-1.49
NPAT	342.9297045	-0.375000829	-2.769273265	0.005618149	0.03183554	-1.30
RP1-186E20.2	205.3009727	0.43443331	2.769067355	0.005621701	0.03184487	1.35
RAPGEFL1	72.41140541	0.5190215	2.768661459	0.005628709	0.03187376	1.43
MIR6723	31.45142306	0.609146515	2.76810795	0.005638278	0.03191712	1.53
SCAND1	469.5493277	0.331316662	2.767305947	0.005652168	0.03197408	1.26
CACNA1C	722.4213148	-0.362992166	-2.765186548	0.005689025	0.03213905	-1.29
CNIH3	40.48210711	-0.580766736	-2.763052198	0.005726361	0.03231718	-1.50
INVS	366.785887	-0.323396491	-2.761101826	0.005760671	0.03248886	-1.25

AP003419.11	14.11478998	0.621537792	2.757877521	0.005817799	0.03273383	1.54
TRPS1	562.5677484	-0.326657711	-2.755193271	0.005865747	0.03298126	-1.25
HJURP	72.69675276	-0.508257163	-2.754794182	0.005872907	0.03299082	-1.42
ARL13B	270.6770511	-0.326920122	-2.753436289	0.005897325	0.03311422	-1.25
AC097523.3	20.98708415	-0.620876169	-2.753129141	0.005902861	0.03313417	-1.54
PTGFR	279.1717953	-0.32395536	-2.750359095	0.005952999	0.03339316	-1.25
AC021016.7	29.64517552	-0.612452981	-2.749981109	0.00595987	0.03342049	-1.53
RP11-90L1.8	44.09596527	0.574793126	2.745300301	0.006045555	0.03385551	1.49
C1D	233.8670082	0.378923782	2.743646584	0.006076091	0.03399233	1.30
CBWD2	363.6600805	-0.387406765	-2.742717001	0.006093317	0.0340649	-1.31
RPRD2	787.6018867	-0.322037637	-2.741902673	0.006108444	0.0341162	-1.25
RP11-142G7.1	185.5528658	-0.384096843	-2.741238005	0.006120815	0.03417387	-1.31
TMEM240	27.8600454	0.605653903	2.73905228	0.006161657	0.03433305	1.52
URB2	229.4475462	-0.376024754	-2.739227654	0.006158371	0.03433305	-1.30
ZNF641	276.0360133	-0.32932251	-2.738354548	0.006174747	0.03439451	-1.26
KAT6A	970.7116471	-0.343781961	-2.737845153	0.006184319	0.03443635	-1.27
AP1G2	101.8890123	0.453962777	2.736895155	0.006202206	0.03450145	1.37
RP11-426C22.4	48.98591398	-0.562176028	-2.736958056	0.00620102	0.03450145	-1.48
KMT5C	102.2299643	0.449040862	2.736487838	0.006209889	0.03450971	1.37
LYPLAL1	251.5840438	-0.355930758	-2.736527755	0.006209136	0.03450971	-1.28
AC005682.5	39.87737495	0.575335748	2.735683251	0.006225091	0.03457713	1.49
CTA-407F11.6	67.1222441	-0.521750793	-2.733809685	0.006260622	0.03475696	-1.44
ZNF608	61.82093703	-0.539197219	-2.733629772	0.006264043	0.0347644	-1.45
NME1-NME2	42.97000253	0.581080361	2.733344893	0.006269464	0.03478293	1.50
LSM12	205.3248329	0.35608857	2.733220972	0.006271824	0.03478447	1.28
RP11-115C21.2	50.97710192	0.556914071	2.731903583	0.006296957	0.03490068	1.47
INTS7	334.2753398	-0.345424692	-2.731468735	0.006305272	0.03493518	-1.27
ZNF780A	279.7173206	-0.331469245	-2.730134858	0.006330842	0.03506523	-1.26
ATPAF2	253.2290205	0.337562873	2.728698951	0.006358472	0.03517887	1.26
LAMC2	167.3524615	0.37921241	2.727674416	0.006378253	0.03525768	1.30
RP11-1148L6.8	61.36624297	0.52161285	2.727002545	0.006391255	0.03531787	1.44
TRIM68	240.5018434	-0.347635118	-2.725521877	0.006419992	0.0354415	-1.27
ZNF28	324.5416684	-0.328236103	-2.725331173	0.006423702	0.03545026	-1.26
DUXAP10	49.62531777	0.602587568	2.724163452	0.006446459	0.03555236	1.52
C21orf91	291.7351202	-0.324741668	-2.72398787	0.006449888	0.03555953	-1.25
CLN3	116.4194273	-0.437487419	-2.723199962	0.006465291	0.03562094	-1.35
DKK2	81.23236436	0.478792941	2.722079895	0.006487246	0.03573012	1.39
CHST10	167.926628	-0.377169815	-2.712438093	0.006679026	0.03664583	-1.30
MNS1	59.53923098	-0.516772931	-2.711563277	0.006696676	0.03672619	-1.43
FRYL	836.5586529	-0.330739745	-2.709447507	0.006739537	0.03686443	-1.26
CCDC85C	267.5749048	0.327106009	2.707224989	0.006784826	0.03708787	1.25

BIRC3	60.92457335	-0.523776886	-2.707116185	0.00678705	0.03708789	-1.44
RP11-32B5.1	278.2640018	-0.398111263	-2.706699405	0.006795576	0.03712233	-1.32
FAT4	743.1188787	-0.344712755	-2.704626006	0.006838133	0.03730601	-1.27
SNX16	217.1396923	-0.351538354	-2.703119722	0.0068692	0.03743882	-1.28
KLHDC7B	20.40094411	-0.610234919	-2.702017479	0.006892014	0.03752643	-1.53
PDE4B	221.8954892	0.354857265	2.701843614	0.006895619	0.03753033	1.28
EBF2	192.721091	0.379493838	2.69847035	0.006965894	0.03783006	1.30
ATP8B3	68.2004521	0.514047436	2.698208775	0.00697137	0.0378475	1.43
SLX1A	40.77361573	0.59971845	2.69796201	0.00697654	0.03786326	1.52
CHST6	154.5453222	-0.405003762	-2.695944639	0.007018933	0.03803534	-1.32
RP11-173E2.1	152.0210534	-0.45822891	-2.695911479	0.007019632	0.03803534	-1.37
IL7	24.4457977	-0.601117531	-2.692522118	0.007091383	0.0383278	-1.52
CTD-2037K23.2	36.61707434	-0.575039522	-2.691306674	0.007117274	0.03843972	-1.49
PDK3	76.58956963	0.480704198	2.688037521	0.007187332	0.03874294	1.40
SRCAP	144.3520108	-0.467470739	-2.687259388	0.007204098	0.03882079	-1.38
BHLHB9	119.2903331	-0.425618353	-2.683770279	0.007279711	0.03915247	-1.34
RP11-384B12.3	80.25748053	-0.500024338	-2.683849952	0.007277976	0.03915247	-1.41
CTU2	329.7910356	0.328409896	2.681496506	0.007329368	0.03930904	1.26
HNRNPA1L2	180.5512459	0.382623291	2.680855047	0.007343432	0.03934318	1.30
RGN	50.17480804	0.550717098	2.680475321	0.007351769	0.03935214	1.46
ABHD8	231.276448	0.345702868	2.679857267	0.007365356	0.03941008	1.27
SCN1B	194.9507522	-0.360911904	-2.679438774	0.007374569	0.03944674	-1.28
KMT2C	1110.585136	-0.329114306	-2.679136444	0.007381231	0.03946974	-1.26
GAREM2	213.3007066	0.366075827	2.678732161	0.007390148	0.03949214	1.29
AC004383.4	72.08831215	0.487998904	2.677721771	0.007412476	0.03956084	1.40
RP11-844P9.3	35.29080604	-0.578766511	-2.677795042	0.007410855	0.03956084	-1.49
ALS2CL	191.8540944	0.377590123	2.677508495	0.007417197	0.03957339	1.30
PKMYT1	34.52847901	-0.572374721	-2.676074906	0.007448999	0.03969236	-1.49
RP3-425C14.4	77.57954692	0.491293198	2.675932033	0.007452176	0.03969662	1.41
KALRN	202.2693487	-0.383887427	-2.673627125	0.007503583	0.03990683	-1.30
RP11-344N10.3	54.00403959	0.535684349	2.671461427	0.007552175	0.04011412	1.45
ZNF236	224.8058304	-0.381279627	-2.670659	0.007570251	0.04015911	-1.30
LY6K	70.99731163	0.503877497	2.665295458	0.007692071	0.04063759	1.42
AC012531.25	31.51171484	-0.585914481	-2.664391122	0.007712783	0.04073413	-1.50
DDX18P1	36.9730674	-0.56995571	-2.664147299	0.007718376	0.04075078	-1.48
RP11-195O1.1	183.8445223	0.363732339	2.662180551	0.007763622	0.04093791	1.29
ARHGAP42	153.1958372	-0.447234452	-2.660372194	0.007805434	0.04109204	-1.36
KIAA0040	93.48502364	-0.473436687	-2.659904877	0.007816272	0.04109204	-1.39
GCNA	44.48845074	0.552416398	2.658761194	0.007842853	0.04117375	1.47
KCNMB1	60.33796055	0.509637281	2.657976234	0.007861144	0.04121791	1.42
RP11-174O3.1	120.8270395	0.422568224	2.658118098	0.007857835	0.04121791	1.34

NCAPH	38.01542413	-0.566853005	-2.654096989	0.007952097	0.04162951	-1.48
PPOX	239.0338342	0.334158182	2.652852137	0.007981483	0.04177026	1.26
ZNF845	146.1475057	-0.396981608	-2.651860642	0.008004957	0.04186373	-1.32
SLC5A6	284.7790168	-0.323221257	-2.651201907	0.008020588	0.04192238	-1.25
TSNARE1	99.36425549	-0.452183292	-2.650420852	0.008039156	0.04198003	-1.37
GPR39	97.75883852	0.460476866	2.649030179	0.008072312	0.04211367	1.38
MYCBP2	1797.862708	-0.560849986	-2.646200732	0.00814015	0.04242783	-1.48
DDX60	107.0362577	-0.447641721	-2.64608841	0.008142853	0.04242868	-1.36
CEP350	963.3522925	-0.322224898	-2.645607565	0.008154436	0.04247579	-1.25
P2RX5	89.65009289	0.457117262	2.644325806	0.008185383	0.0426237	1.37
NEK2	25.97844173	-0.593529962	-2.643757457	0.008199138	0.04265544	-1.51
ARHGEF19	70.58915695	0.510678729	2.64121594	0.008260905	0.04289662	1.42
ZNRF2	85.62758208	-0.46964324	-2.637465691	0.008352807	0.04333343	-1.38
IRX2	186.3134565	-0.373666223	-2.636743307	0.008370615	0.0433571	-1.30
CCNB2	69.68432651	-0.48281195	-2.63703667	0.008363379	0.0433571	-1.40
TTC21A	47.84165164	-0.545732619	-2.635378768	0.008404344	0.04349274	-1.46
CCDC81	58.09649425	0.516115528	2.634546448	0.008424978	0.04357252	1.43
RP11-557J10.3	110.0385151	-0.422849051	-2.632020879	0.008487865	0.04381637	-1.34
MCM5	217.5616183	-0.338485947	-2.630100954	0.008535952	0.04403739	-1.26
HIST1H2BE	21.26845673	0.594217512	2.629422337	0.008553007	0.04406652	1.51
TCEA3	63.00705218	0.499056419	2.62931739	0.008555647	0.04406652	1.41
ASPHD2	98.30079437	0.439677427	2.629360304	0.008554567	0.04406652	1.36
DHX58	76.88007198	-0.487841213	-2.629246718	0.008557425	0.04406652	-1.40
WDR90	214.5629522	0.359253517	2.626761111	0.008620183	0.04433502	1.28
RP5-1053E7.3	42.54423185	-0.547875915	-2.626192818	0.008634589	0.04439545	-1.46
STARD10	157.3344624	-0.393594946	-2.62334936	0.008706994	0.04472642	-1.31
RP4-710L4.1	283.0888447	-0.37202405	-2.621874338	0.008744767	0.04489285	-1.29
RP11-325F22.3	11.06992671	0.570566645	2.620736417	0.008774007	0.04501529	1.49
RP11-115J23.1	9.237658978	0.549116609	2.619036634	0.008817848	0.04518471	1.46
RP5-1042K10.14	79.76701524	0.463721786	2.618490884	0.008831965	0.04524317	1.38
RRM2	89.10005904	-0.460425921	-2.617290482	0.008863088	0.04533309	-1.38
CTB-55O6.8	37.80916911	0.564475609	2.614874204	0.008926032	0.04561314	1.48
AC001226.1	283.9615362	-0.325683703	-2.61340092	0.008964607	0.04576827	-1.25
EFNA3	34.11184233	0.566152478	2.612847516	0.008979135	0.04582843	1.48
TPCN2	243.0952723	0.347261055	2.612347105	0.00899229	0.04588156	1.27
FAM174B	43.06372309	0.549742774	2.609922579	0.009056272	0.0461798	1.46
RAPGEF2	988.9354049	-0.328071748	-2.60822792	0.009101233	0.04636661	-1.26
MKX	63.75150333	-0.495170244	-2.606729484	0.009141155	0.04655579	-1.41
CTD-2396E7.8	12.60897226	-0.566529996	-2.605700336	0.009168664	0.04666744	-1.48
CDCA3	54.62336578	-0.511523967	-2.604171505	0.009209666	0.04686185	-1.43
FARP2	335.7986831	-0.365092813	-2.603785193	0.009220052	0.04690042	-1.29

RP11-424C20.2	56.47674107	-0.503238044	-2.602236446	0.009261797	0.04708409	-1.42
FOSB	47.91539494	0.530110226	2.600796658	0.009300756	0.04723902	1.44
WDR7	591.006418	-0.32210226	-2.600264954	0.009315181	0.04728265	-1.25
ENOX2	230.3023646	-0.333783432	-2.599044144	0.009348375	0.04739442	-1.26
AC004696.2	215.7503188	-0.337083347	-2.59911755	0.009346376	0.04739442	-1.26
MAGED4B	674.5619404	0.359068827	2.598685996	0.009358133	0.04742393	1.28
RP4-791C19.1	50.35187139	-0.524568324	-2.59851802	0.009362713	0.04742393	-1.44
RP11-156P1.3	134.9424956	-0.414777462	-2.598244812	0.009370166	0.04744209	-1.33
XX-BC751M15.1	122.9253155	-0.461100294	-2.597943413	0.009378395	0.04746019	-1.38
RP4-641G12.4	91.67634916	-0.446432193	-2.597250873	0.009397326	0.04754159	-1.36
C19orf18	17.88544304	0.589140316	2.596973395	0.009404921	0.04756561	1.50
ASTE1	131.7102097	-0.401014145	-2.596730957	0.009411561	0.04758478	-1.32
RHOJ	132.658472	-0.398315803	-2.593117055	0.009511039	0.04797159	-1.32
HMMR	53.71924464	-0.527915777	-2.592886549	0.009517416	0.04798927	-1.44
RP11-384F7.2	28.65621784	-0.569094678	-2.59124824	0.009562848	0.0482038	-1.48
CCDC85A	139.0029775	-0.39513336	-2.590947429	0.009571211	0.04820683	-1.32
CDR1-AS	103.5561986	-0.435212871	-2.590424801	0.009585756	0.04824668	-1.35
WBP1	156.6562263	0.374706696	2.588900957	0.009628278	0.04837728	1.30
KLHL17	293.4881565	0.323884547	2.588551465	0.009638054	0.04840761	1.25
POLR2A	1153.471945	-0.324193301	-2.587969726	0.009654347	0.04847486	-1.25
RP11-252I13.1	184.2276814	-0.370174013	-2.587695151	0.009662045	0.04849894	-1.29
DNALI1	137.2054285	0.404076838	2.583412998	0.009782814	0.04906091	1.32
RP11-799A12.1	29.7926393	-0.568717775	-2.582281199	0.009814958	0.04917782	-1.48
OSTCL	33.4629371	0.569555556	2.581520045	0.009836628	0.04927162	1.48
ZNF836	72.5763847	-0.466534276	-2.578890043	0.009911833	0.049574	-1.38
BHMT2	82.25759825	0.450183116	2.57787111	0.009941107	0.04970132	1.37
C5orf45	198.7747474	0.345253794	2.575175025	0.010018937	0.04998275	1.27

Supplemental Table S3. Biological Processes Gene Ontology for Upregulated Genes in 1 Gy Irradiated Human Mesenchymal Stem Cells

Positive regulation of vasoconstriction – enrichment score: 11.0

ID	Gene Name	Species
3356	5-hydroxytryptamine receptor 2A(HTR2A)	Homo sapiens
147	adrenoceptor alpha 1B(ADRA1B)	Homo sapiens
146	adrenoceptor alpha 1D(ADRA1D)	Homo sapiens
1906	endothelin 1(EDN1)	Homo sapiens
3952	leptin(LEP)	Homo sapiens
2702	gap junction protein alpha 5(GJA5)	Homo sapiens
4883	natriuretic peptide receptor 3(NPR3)	Homo sapiens
5021	oxytocin receptor(OXTR)	Homo sapiens
5742	prostaglandin-endoperoxide synthase 1(PTGS1)	Homo sapiens
5743	prostaglandin-endoperoxide synthase 2(PTGS2)	Homo sapiens

Regulation of glucose metabolic process/homeostasis – enrichment score: 10.7

ID	Gene Name	Species
3486	insulin like growth factor binding protein 3(IGFBP3)	Homo sapiens
3488	insulin like growth factor binding protein 5(IGFBP5)	Homo sapiens
3569	interleukin 6(IL6)	Homo sapiens
3939	lactate dehydrogenase A(LDHA)	Homo sapiens
3952	leptin(LEP)	Homo sapiens
5163	pyruvate dehydrogenase kinase 1(PDK1)	Homo sapiens
5166	pyruvate dehydrogenase kinase 4(PDK4)	Homo sapiens

Positive regulation of cell proliferation – enrichment score: 6.9

ID	Gene Name	Species
255324	epithelial mitogen(EPGN)	Homo sapiens
2246	fibroblast growth factor 1(FGF1)	Homo sapiens
3569	interleukin 6(IL6)	Homo sapiens
8013	nuclear receptor subfamily 4 group A member 3(NR4A3)	Homo sapiens
130497	odd-skipped related transciption factor 1(OSR1)	Homo sapiens
7039	transforming growth factor alpha(TGFA)	Homo sapiens
7422	vascular endothelial growth factor A(VEGFA)	Homo sapiens

Cell response to hypoxia – enrichment score: 6.8

ID	Gene Name	Species
664	BCL2 interacting protein 3(BNIP3)	Homo sapiens
358	aquaporin 1 (Colton blood group)(AQP1)	Homo sapiens
1906	endothelin 1(EDN1)	Homo sapiens
26355	family with sequence similarity 162 member A(FAM162A)	Homo sapiens
79625	neuron derived neurotrophic factor(NDNF)	Homo sapiens
3779	potassium calcium-activated channel subfamily M regulatory beta subunit 1(KCNMB1)	Homo sapiens
3777	potassium two pore domain channel subfamily K member 3(KCNK3)	Homo sapiens
5743	prostaglandin-endoperoxide synthase 2(PTGS2)	Homo sapiens
6546	solute carrier family 8 member A1(SLC8A1)	Homo sapiens
6781	stanniocalcin 1(STC1)	Homo sapiens
7422	vascular endothelial growth factor A(VEGFA)	Homo sapiens

Positive regulation of cell adhesion – enrichment score: 5.5

ID	Gene Name	Species
54566	erythrocyte membrane protein band 4.1 like 4B(EPB41L4B)	Homo sapiens
3084	neuregulin 1(NRG1)	Homo sapiens

7052	transglutaminase 2(TGM2)	Homo sapiens
7422	vascular endothelial growth factor A(VEGFA)	Homo sapiens
1674	desmin(DES)	Homo sapiens
1832	desmoplakin(DSP)	Homo sapiens
2312	filaggrin(FLG)	Homo sapiens
3861	keratin 14(KRT14)	Homo sapiens
3868	keratin 16(KRT16)	Homo sapiens
3875	keratin 18(KRT18)	Homo sapiens
3885	keratin 34(KRT34)	Homo sapiens
3855	keratin 7(KRT7)	Homo sapiens
3856	keratin 8(KRT8)	Homo sapiens
144501	keratin 80(KRT80)	Homo sapiens
3887	keratin 81(KRT81)	Homo sapiens
10763	nestin(NES)	Homo sapiens

Extracellular matrix organization – enrichment score: 4.5

ID	Gene Name	Species
9510	ADAM metallopeptidase with thrombospondin type 1 motif 1(ADAMTS1)	Homo sapiens
81792	ADAM metallopeptidase with thrombospondin type 1 motif 12(ADAMTS12)	Homo sapiens
11174	ADAM metallopeptidase with thrombospondin type 1 motif 6(ADAMTS6)	Homo sapiens
92949	ADAMTS like 1(ADAMTSL1)	Homo sapiens
4982	TNF receptor superfamily member 11b(TNFRSF11B)	Homo sapiens
151887	coiled-coil domain containing 80(CCDC80)	Homo sapiens
1278	collagen type I alpha 2 chain(COL1A2)	Homo sapiens
1286	collagen type IV alpha 4 chain(COL4A4)	Homo sapiens
1289	collagen type V alpha 1 chain(COL5A1)	Homo sapiens
1305	collagen type XIII alpha 1 chain(COL13A1)	Homo sapiens
3491	cysteine rich angiogenic inducer 61(CYR61)	Homo sapiens
2200	fibrillin 1(FBN1)	Homo sapiens
2246	fibroblast growth factor 1(FGF1)	Homo sapiens
3036	hyaluronan synthase 1(HAS1)	Homo sapiens
3679	integrin subunit alpha 7(ITGA7)	Homo sapiens
4015	lysyl oxidase(LOX)	Homo sapiens
84171	lysyl oxidase like 4(LOXL4)	Homo sapiens
79812	multimerin 2(MMRN2)	Homo sapiens
79625	neuron derived neurotrophic factor(NDNF)	Homo sapiens
5352	procollagen-lysine,2-oxoglutarate 5-dioxygenase 2(PLOD2)	Homo sapiens
26577	procollagen C-endopeptidase enhancer 2(PCOLCE2)	Homo sapiens
55214	prolyl 3-hydroxylase 2(P3H2)	Homo sapiens
5033	prolyl 4-hydroxylase subunit alpha 1(P4HA1)	Homo sapiens
8434	reversion inducing cysteine rich protein with kazal motifs(RECK)	Homo sapiens
5054	serpin family E member 1(SERPINE1)	Homo sapiens
7045	transforming growth factor beta induced(TGFBI)	Homo sapiens
7448	vitronectin(VTN)	Homo sapiens

Positive regulation of cell migration – enrichment score: 3.3

ID	Gene Name	Species
29126	CD274 molecule(CD274)	Homo sapiens
285966	TRPM8 channel associated factor 2(TCAF2)	Homo sapiens
81029	Wnt family member 5B(WNT5B)	Homo sapiens
57214	cell migration inducing hyaluronan binding protein(CEMIP)	Homo sapiens
3491	cysteine rich angiogenic inducer 61(CYR61)	Homo sapiens
1906	endothelin 1(EDN1)	Homo sapiens
2246	fibroblast growth factor 1(FGF1)	Homo sapiens
1839	heparin binding EGF like growth factor(HBEGF)	Homo sapiens

3037	hyaluronan synthase 2(HAS2)	Homo sapiens
8482	semaphorin 7A (John Milton Hagen blood group)(SEMA7A)	Homo sapiens
7422	vascular endothelial growth factor A(VEGFA)	Homo sapiens

Positive regulation of apoptosis – enrichment score: 2.7

ID	Gene Name	Species
664	BCL2 interacting protein 3(BNIP3)	Homo sapiens
1843	dual specificity phosphatase 1(DUSP1)	Homo sapiens
1848	dual specificity phosphatase 6(DUSP6)	Homo sapiens
1917	eukaryotic translation elongation factor 1 alpha 2(EEF1A2)	Homo sapiens
26355	family with sequence similarity 162 member A(FAM162A)	Homo sapiens
3486	insulin like growth factor binding protein 3(IGFBP3)	Homo sapiens
3569	interleukin 6(IL6)	Homo sapiens
3939	lactate dehydrogenase A(LDHA)	Homo sapiens
4803	nerve growth factor(NGF)	Homo sapiens
3164	nuclear receptor subfamily 4 group A member 1(NR4A1)	Homo sapiens
10687	paraneoplastic Ma antigen 2(PNMA2)	Homo sapiens
5743	prostaglandin-endoperoxide synthase 2(PTGS2)	Homo sapiens
388	ras homolog family member B(RHOB)	Homo sapiens
7052	transglutaminase 2(TGM2)	Homo sapiens

Negative regulation of apoptosis – enrichment score: 2.7

ID	Gene Name	Species
664	BCL2 interacting protein 3(BNIP3)	Homo sapiens
10370	Cbp/p300 interacting transactivator with Glu/Asp rich carboxy-terminal domain 2(CITED2)	Homo sapiens
7010	TEK receptor tyrosine kinase(TEK)	Homo sapiens
8793	TNF receptor superfamily member 10d(TNFRSF10D)	Homo sapiens
51129	angiopoietin like 4(ANGPTL4)	Homo sapiens
358	aquaporin 1 (Colton blood group)(AQP1)	Homo sapiens
3491	cysteine rich angiogenic inducer 61(CYR61)	Homo sapiens
1843	dual specificity phosphatase 1(DUSP1)	Homo sapiens
1960	early growth response 3(EGR3)	Homo sapiens
8870	immediate early response 3(IER3)	Homo sapiens
3569	interleukin 6(IL6)	Homo sapiens
3875	keratin 18(KRT18)	Homo sapiens
3952	leptin(LEP)	Homo sapiens
4803	nerve growth factor(NGF)	Homo sapiens
130497	odd-skipped related transciption factor 1(OSR1)	Homo sapiens
5563	protein kinase AMP-activated catalytic subunit alpha 2(PRKAA2)	Homo sapiens
81567	thioredoxin domain containing 5(TXNDC5)	Homo sapiens
7039	transforming growth factor alpha(TGFA)	Homo sapiens
7052	transglutaminase 2(TGM2)	Homo sapiens
7422	vascular endothelial growth factor A(VEGFA)	Homo sapiens

Negative regulation of cell proliferation – enrichment score: 1.8

ID	Gene Name	Species
9510	ADAM metallopeptidase with thrombospondin type 1 motif 1(ADAMTS1)	Homo sapiens
3725	Jun proto-oncogene, AP-1 transcription factor subunit(JUN)	Homo sapiens
3814	KiSS-1 metastasis-suppressor(KISS1)	Homo sapiens
104	adenosine deaminase, RNA specific B1(ADARB1)	Homo sapiens
3486	insulin like growth factor binding protein 3(IGFBP3)	Homo sapiens
3569	interleukin 6(IL6)	Homo sapiens
3976	leukemia inhibitory factor(LIF)	Homo sapiens

93649	myocardin(MYOCD)	Homo sapiens
5744	parathyroid hormone like hormone(PTHLH)	Homo sapiens
55214	prolyl 3-hydroxylase 2(P3H2)	Homo sapiens
5743	prostaglandin-endoperoxide synthase 2(PTGS2)	Homo sapiens
5918	retinoic acid receptor responder 1(RARRES1)	Homo sapiens

Supplemental Table S4. Biological Processes Gene Ontology for Downregulated Genes in 1 Gy Irradiated Human Mesenchymal Stem Cells

Cholesterol biosynthesis – enrichment score: 20.5

ID	Gene Name	Species
1718	24-dehydrocholesterol reductase(DHCR24)	Homo sapiens
3156	3-hydroxy-3-methylglutaryl-CoA reductase(HMGCR)	Homo sapiens
3157	3-hydroxy-3-methylglutaryl-CoA synthase 1(HMGCS1)	Homo sapiens
1717	7-dehydrocholesterol reductase(DHCR7)	Homo sapiens
1595	cytochrome P450 family 51 subfamily A member 1(CYP51A1)	Homo sapiens
10682	emopamil binding protein (sterol isomerase)(EBP)	Homo sapiens
2224	farnesyl diphosphate synthase(FDPS)	Homo sapiens
2222	farnesyl-diphosphate farnesyltransferase 1(FDFT1)	Homo sapiens
51478	hydroxysteroid 17-beta dehydrogenase 7(HSD17B7)	Homo sapiens
3638	insulin induced gene 1(INSIG1)	Homo sapiens
3422	isopentenyl-diphosphate delta isomerase 1(IDI1)	Homo sapiens
4047	lanosterol synthase (2,3-oxidosqualene-lanosterol cyclase)(LSS)	Homo sapiens
6307	methylsterol monooxygenase 1(MSMO1)	Homo sapiens
4597	mevalonate diphosphate decarboxylase(MVD)	Homo sapiens
4598	mevalonate kinase(MVK)	Homo sapiens
6713	squalene epoxidase(SQLE)	Homo sapiens
7108	transmembrane 7 superfamily member 2(TM7SF2)	Homo sapiens

Iron ion homeostasis/iron binding – enrichment score: 6.1

ID	Gene Name	Species
1593	cytochrome P450 family 27 subfamily A member 1(CYP27A1)	Homo sapiens
1595	cytochrome P450 family 51 subfamily A member 1(CYP51A1)	Homo sapiens
79901	cytochrome b reductase 1(CYBRD1)	Homo sapiens
114757	cytoglobin(CYGB)	Homo sapiens
2495	ferritin heavy chain 1(FTH1)	Homo sapiens
2512	ferritin light chain(FTL)	Homo sapiens
3162	heme oxygenase 1(HMOX1)	Homo sapiens
307	methylsterol monooxygenase 1(MSMO1)	Homo sapiens
30061	solute carrier family 40 member 1(SLC40A1)	Homo sapiens
26872	STEAP family member 1(STEAP1)	Homo sapiens
6319	stearoyl-CoA desaturase(SCD)	Homo sapiens
6309	sterol-C5-desaturase(SC5D)	Homo sapiens
6648	superoxide dismutase 2, mitochondrial(SOD2)	Homo sapiens

Cellular response to starvation – enrichment score: 5.8

ID	Gene Name	Species
8644	aldo-keto reductase family 1 member C3(AKR1C3)	Homo sapiens
93664	calcium dependent secretion activator 2(CADPS2)	Homo sapiens
3992	fatty acid desaturase 1(FADS1)	Homo sapiens
96459	folliculin interacting protein 1(FNIP1)	Homo sapiens
2629	glucosylceramidase beta(GBA)	Homo sapiens
81788	NUAK family kinase 2(NUAK2)	Homo sapiens
255738	proprotein convertase subtilisin/kexin type 9(PCSK9)	Homo sapiens

Positive regulation of apoptotic process – enrichment score: 5.5

ID	Gene Name	Species
10018	BCL2 like 11(BCL2L11)	Homo sapiens
90427	Bcl2 modifying factor(BMF)	Homo sapiens
834	caspase 1(CASP1)	Homo sapiens
23604	death associated protein kinase 2(DAPK2)	Homo sapiens
1019	cyclin dependent kinase 4(CDK4)	Homo sapiens

201163	folliculin(FLCN)	Homo sapiens
26471	nuclear protein 1, transcriptional regulator(NUPR1)	Homo sapiens
29948	oxidative stress induced growth inhibitor 1(OSGIN1)	Homo sapiens
79156	pleckstrin homology and FYVE domain containing 1(PLEKHF1)	Homo sapiens
5764	pleiotrophin(PTN)	Homo sapiens
6423	secreted frizzled related protein 2(SFRP2)	Homo sapiens
6424	secreted frizzled related protein 4(SFRP4)	Homo sapiens
8878	sequestosome 1(SQSTM1)	Homo sapiens
7098	toll like receptor 3(TLR3)	Homo sapiens
7023	transcription factor AP-4(TFAP4)	Homo sapiens

Negative regulation of inflammatory response – enrichment score: 5.2

ID	Gene Name	Species
55872	PDZ binding kinase(PBK)	Homo sapiens
7130	TNF alpha induced protein 6(TNFAIP6)	Homo sapiens
54	acid phosphatase 5, tartrate resistant(ACP5)	Homo sapiens
100	adenosine deaminase(ADA)	Homo sapiens
348	apolipoprotein E(APOE)	Homo sapiens
2629	glucosylceramidase beta(GBA)	Homo sapiens
4598	mevalonate kinase(MVK)	Homo sapiens
4856	nephroblastoma overexpressed(NOV)	Homo sapiens
5734	prostaglandin E receptor 4(PTGER4)	Homo sapiens

Response to reactive oxygen species – enrichment score: 4.7

ID	Gene Name	Species
1718	24-dehydrocholesterol reductase(DHCR24)	Homo sapiens
100	adenosine deaminase(ADA)	Homo sapiens
348	apolipoprotein E(APOE)	Homo sapiens
2876	glutathione peroxidase 1(GPX1)	Homo sapiens
2879	glutathione peroxidase 4(GPX4)	Homo sapiens
3417	isocitrate dehydrogenase (NADP(+)) 1, cytosolic(IDH1)	Homo sapiens
6648	superoxide dismutase 2, mitochondrial(SOD2)	Homo sapiens
6649	superoxide dismutase 3, extracellular(SOD3)	Homo sapiens
6382	syndecan 1(SDC1)	Homo sapiens

Regulation of autophagy – enrichment score: 4.6

ID	Gene Name	Species
55332	DNA damage regulated autophagy modulator 1(DRAM1)	Homo sapiens
64121	Ras related GTP binding C(RRAGC)	Homo sapiens
834	caspase 1(CASP1)	Homo sapiens
23604	death associated protein kinase 2(DAPK2)	Homo sapiens
3428	interferon gamma inducible protein 16(IFI16)	Homo sapiens

Fatty acid/ Lipid metabolic processes – enrichment score: 4.4

ID	Gene Name	Species
80221	acyl-CoA synthetase family member 2(ACSF2)	Homo sapiens
2170	fatty acid binding protein 3(FABP3)	Homo sapiens
2194	fatty acid synthase(FASN)	Homo sapiens
6307	methylsterol monooxygenase 1(MSMO1)	Homo sapiens
3157	3-hydroxy-3-methylglutaryl-CoA synthase 1(HMGCS1)	Homo sapiens
427	N-acylsphingosine amidohydrolase 1(ASAH1)	Homo sapiens
39	acetyl-CoA acetyltransferase 2(ACAT2)	Homo sapiens
27132	copine 7(CPNE7)	Homo sapiens
3992	fatty acid desaturase 1(FADS1)	Homo sapiens
9415	fatty acid desaturase 2(FADS2)	Homo sapiens

9388	lipase G, endothelial type(LIPG)	Homo sapiens
3949	low density lipoprotein receptor(LDLR)	Homo sapiens
51365	phospholipase A1 member A(PLA1A)	Homo sapiens
5730	prostaglandin D2 synthase(PTGDS)	Homo sapiens
6309	sterol-C5-desaturase(SC5D)	Homo sapiens
1200	tripeptidyl peptidase 1(TPP1)	Homo sapiens

Collagen catabolic process – enrichment score: 4.3

ID	Gene Name	Species
1509	cathepsin D(CTSD)	Homo sapiens
1514	cathepsin L(CTSL)	Homo sapiens
4312	matrix metallopeptidase 1(MMP1)	Homo sapiens
4320	matrix metallopeptidase 11(MMP11)	Homo sapiens
4324	matrix metallopeptidase 15(MMP15)	Homo sapiens
5184	peptidase D(PEPD)	Homo sapiens

Regulation of blood pressure – enrichment score: 3.5

ID	Gene Name	Species
183	angiotensinogen(AGT)	Homo sapiens
1909	endothelin receptor type A(EDNRA)	Homo sapiens
3162	heme oxygenase 1(HMOX1)	Homo sapiens
5973	renin binding protein(RENBP)	Homo sapiens
6648	superoxide dismutase 2, mitochondrial(SOD2)	Homo sapiens

Positive regulation of cell proliferation – enrichment score: 2.9

ID	Gene Name	Species
3357	5-hydroxytryptamine receptor 2B(HTR2B)	Homo sapiens
6926	T-box 3(TBX3)	Homo sapiens
11138	TBC1 domain family member 8(TBC1D8)	Homo sapiens
1646	aldo-keto reductase family 1 member C2(AKR1C2)	Homo sapiens
8644	aldo-keto reductase family 1 member C3(AKR1C3)	Homo sapiens
332	baculoviral IAP repeat containing 5(BIRC5)	Homo sapiens
991	cell division cycle 20(CDC20)	Homo sapiens
994	cell division cycle 25B(CDC25B)	Homo sapiens
2149	coagulation factor II thrombin receptor(F2R)	Homo sapiens
1435	colony stimulating factor 1(CSF1)	Homo sapiens
1019	cyclin dependent kinase 4(CDK4)	Homo sapiens
891	cyclin B1(CCNB1)	Homo sapiens
9133	cyclin B2(CCNB2)	Homo sapiens
8814	cyclin dependent kinase like 1(CDKL1)	Homo sapiens
2305	forkhead box M1(FOXM1)	Homo sapiens
3280	hes family bHLH transcription factor 1(HES1)	Homo sapiens
8660	insulin receptor substrate 2(IRS2)	Homo sapiens
3574	interleukin 7(IL7)	Homo sapiens
4597	mevalonate diphosphate decarboxylase(MVD)	Homo sapiens
4828	neuromedin B(NMB)	Homo sapiens
4915	neurotrophic receptor tyrosine kinase 2(NTRK2)	Homo sapiens
5764	pleiotrophin(PTN)	Homo sapiens
6423	secreted frizzled related protein 2(SFRP2)	Homo sapiens
9481	solute carrier family 25 member 27(SLC25A27)	Homo sapiens
6809	syntaxin 3(STX3)	Homo sapiens
64759	tensin 3(TNS3)	Homo sapiens

Cellular adhesion – enrichment score: 1.7

ID	Gene Name	Species
9788	MTSS1, I-BAR domain containing(MTSS1)	Homo sapiens
7130	TNF alpha induced protein 6(TNFAIP6)	Homo sapiens
8839	WNT1 inducible signaling pathway protein 2(WISP2)	Homo sapiens
176	aggrecan(ACAN)	Homo sapiens
9635	chloride channel accessory 2(CLCA2)	Homo sapiens
3280	hes family bHLH transcription factor 1(HES1)	Homo sapiens
3383	intercellular adhesion molecule 1(ICAM1)	Homo sapiens
9235	interleukin 32(IL32)	Homo sapiens
284217	laminin subunit alpha 1(LAMA1)	Homo sapiens
4685	neural cell adhesion molecule 2(NCAM2)	Homo sapiens
57502	neuroligin 4, X-linked(NLGN4X)	Homo sapiens
10631	periostin(POSTN)	Homo sapiens
6696	secreted phosphoprotein 1(SPP1)	Homo sapiens
1901	sphingosine-1-phosphate receptor 1(S1PR1)	Homo sapiens
10417	spondin 2(SPON2)	Homo sapiens
8406	sushi repeat containing protein, X-linked(SRPX)	Homo sapiens
10024	trophinin associated protein(TROAP)	Homo sapiens

Negative regulation of cell proliferation – enrichment score: 1.6

ID	Gene Name	Species
1718	24-dehydrocholesterol reductase(DHCR24)	Homo sapiens
1050	CCAAT/enhancer binding protein alpha(CEBPA)	Homo sapiens
2149	coagulation factor II thrombin receptor(F2R)	Homo sapiens
2170	fatty acid binding protein 3(FABP3)	Homo sapiens
2495	ferritin heavy chain 1(FTH1)	Homo sapiens
7855	frizzled class receptor 5(FZD5)	Homo sapiens
3437	interferon induced protein with tetratricopeptide repeats 3(IFIT3)	Homo sapiens
8519	interferon induced transmembrane protein 1(IFITM1)	Homo sapiens
5376	peripheral myelin protein 22(PMP22)	Homo sapiens
5920	retinoic acid receptor responder 3(RARRES3)	Homo sapiens
6423	secreted frizzled related protein 2(SFRP2)	Homo sapiens
6424	secreted frizzled related protein 4(SFRP4)	Homo sapiens
6648	superoxide dismutase 2, mitochondrial(SOD2)	Homo sapiens
7023	transcription factor AP-4(TFAP4)	Homo sapiens

Supplemental Table S5. Biological Processes Gene Ontology for Upregulated Genes in 2 Gy Irradiated Human Mesenchymal Stem Cells

Positive regulation of cell proliferation – enrichment score: 8.8

ID	Gene Name	Species
654364	NME1-NME2 readthrough(NME1-NME2)	Homo sapiens
255324	epithelial mitogen(EPGN)	Homo sapiens
3569	interleukin 6(IL6)	Homo sapiens
9241	noggin(NOG)	Homo sapiens
3164	nuclear receptor subfamily 4 group A member 1(NR4A1)	Homo sapiens
5228	placental growth factor(PGF)	Homo sapiens
130497	odd-skipped related transciption factor 1(OSR1)	Homo sapiens
7039	transforming growth factor alpha(TGFA)	Homo sapiens
7422	vascular endothelial growth factor A(VEGFA)	Homo sapiens

Cellular response to hypoxia – enrichment score: 7.0

ID	Gene Name	Species
664	BCL2 interacting protein 3(BNIP3)	Homo sapiens
316	aldehyde oxidase 1(AOX1)	Homo sapiens
51129	angiopoietin like 4(ANGPTL4)	Homo sapiens
81575	apolipoprotein L domain containing 1(APOLD1)	Homo sapiens
1535	cytochrome b-245 alpha chain(CYBA)	Homo sapiens
1958	early growth response 1(EGR1)	Homo sapiens
1906	endothelin 1(EDN1)	Homo sapiens
3939	lactate dehydrogenase A(LDHA)	Homo sapiens
3952	leptin(LEP)	Homo sapiens
26355	family with sequence similarity 162 member A(FAM162A)	Homo sapiens
84709	mitochondria localized glutamic acid rich protein(MGARP)	Homo sapiens
93649	myocardin(MYOCD)	Homo sapiens
51079	NADH:ubiquinone oxidoreductase subunit A13(NDUFA13)	Homo sapiens
4929	nuclear receptor subfamily 4 group A member 2(NR4A2)	Homo sapiens
5228	placental growth factor(PGF)	Homo sapiens
5327	plasminogen activator, tissue type(PLAT)	Homo sapiens
5328	plasminogen activator, urokinase(PLAU)	Homo sapiens
3777	potassium two pore domain channel subfamily K member 3(KCNK3)	Homo sapiens
5352	procollagen-lysine,2-oxoglutarate 5-dioxygenase 2(PLOD2)	Homo sapiens
5179	proenkephalin(PENK)	Homo sapiens
5743	prostaglandin-endoperoxide synthase 2(PTGS2)	Homo sapiens
5166	pyruvate dehydrogenase kinase 4(PDK4)	Homo sapiens
29988	solute carrier family 2 member 8(SLC2A8)	Homo sapiens
6781	stanniocalcin 1(STC1)	Homo sapiens
7422	vascular endothelial growth factor A(VEGFA)	Homo sapiens

Apoptotic signaling pathway – enrichment score: 5.3

ID	Gene Name	Species
1958	early growth response 1(EGR1)	Homo sapiens
3625	inhibin beta B subunit(INHBB)	Homo sapiens
3486	insulin like growth factor binding protein 3(IGFBP3)	Homo sapiens
51079	NADH:ubiquinone oxidoreductase subunit A13(NDUFA13)	Homo sapiens
55367	p53-induced death domain protein 1(PIDD1)	Homo sapiens
84957	RELT tumor necrosis factor receptor(RELT)	Homo sapiens
8793	TNF receptor superfamily member 10d(TNFRSF10D)	Homo sapiens
4982	TNF receptor superfamily member 11b(TNFRSF11B)	Homo sapiens
27242	TNF receptor superfamily member 21(TNFRSF21)	Homo sapiens
11035	receptor interacting serine/threonine kinase 3(RIPK3)	Homo sapiens

Regulation of blood pressure – enrichment score: 4.7

ID	Gene Name	Species
1906	endothelin 1(EDN1)	Homo sapiens
3952	leptin(LEP)	Homo sapiens
5742	prostaglandin-endoperoxide synthase 1(PTGS1)	Homo sapiens
5743	prostaglandin-endoperoxide synthase 2(PTGS2)	Homo sapiens

Extracellular matrix organization – enrichment score: 2.7

ID	Gene Name	Species
9510	ADAM metallopeptidase with thrombospondin type 1 motif 1(ADAMTS1)	Homo sapiens
11174	ADAM metallopeptidase with thrombospondin type 1 motif 6(ADAMTS6)	Homo sapiens
4982	TNF receptor superfamily member 11b(TNFRSF11B)	Homo sapiens
1302	collagen type XI alpha 2 chain(COL11A2)	Homo sapiens
1305	collagen type XIII alpha 1 chain(COL13A1)	Homo sapiens
3491	cysteine rich angiogenic inducer 61(CYR61)	Homo sapiens
3381	integrin binding sialoprotein(IBSP)	Homo sapiens
3679	integrin subunit alpha 7(ITGA7)	Homo sapiens
58494	junctional adhesion molecule 2(JAM2)	Homo sapiens
84171	lysyl oxidase like 4(LOXL4)	Homo sapiens
5352	procollagen-lysine,2-oxoglutarate 5-dioxygenase 2(PLOD2)	Homo sapiens
55214	prolyl 3-hydroxylase 2(P3H2)	Homo sapiens
5033	prolyl 4-hydroxylase subunit alpha 1(P4HA1)	Homo sapiens
8974	prolyl 4-hydroxylase subunit alpha 2(P4HA2)	Homo sapiens
5054	serpin family E member 1(SERPINE1)	Homo sapiens

Negative regulation of cell proliferation – enrichment score: 2.3

ID	Gene Name	Species
9510	ADAM metallopeptidase with thrombospondin type 1 motif 1(ADAMTS1)	Homo sapiens
3576	C-X-C motif chemokine ligand 8(CXCL8)	Homo sapiens
26232	F-box protein 2(FBXO2)	Homo sapiens
3814	KiSS-1 metastasis-suppressor(KISS1)	Homo sapiens
3486	insulin like growth factor binding protein 3(IGFBP3)	Homo sapiens
3569	interleukin 6(IL6)	Homo sapiens
3976	leukemia inhibitory factor(LIF)	Homo sapiens
93649	myocardin(MYOCD)	Homo sapiens
5744	parathyroid hormone like hormone(PTHLH)	Homo sapiens
5743	prostaglandin-endoperoxide synthase 2(PTGS2)	Homo sapiens
5918	retinoic acid receptor responder 1(RARRES1)	Homo sapiens

Immune response – enrichment score: 2.0

ID	Gene Name	Species
3576	C-X-C motif chemokine ligand 8(CXCL8)	Homo sapiens
84957	RELT tumor necrosis factor receptor(RELT)	Homo sapiens
8793	TNF receptor superfamily member 10d(TNFRSF10D)	Homo sapiens
4982	TNF receptor superfamily member 11b(TNFRSF11B)	Homo sapiens
27242	TNF receptor superfamily member 21(TNFRSF21)	Homo sapiens
8862	apelin(APLN)	Homo sapiens
3569	interleukin 6(IL6)	Homo sapiens
3976	leukemia inhibitory factor(LIF)	Homo sapiens
6398	secreted and transmembrane 1(SECTM1)	Homo sapiens
8482	semaphorin 7A (John Milton Hagen blood group)(SEMA7A)	Homo sapiens
64129	tubulointerstitial nephritis antigen like 1(TINAGL1)	Homo sapiens

Supplemental Table S6. Biological Processes Gene Ontology for Downregulated Genes in 2 Gy Irradiated Human Mesenchymal Stem Cells

Cholesterol biosynthesis/metabolism – enrichment score: 12.3

ID	Gene Name	Species
1718	24-dehydrocholesterol reductase(DHCR24)	Homo sapiens
3156	3-hydroxy-3-methylglutaryl-CoA reductase(HMGCR)	Homo sapiens
3157	3-hydroxy-3-methylglutaryl-CoA synthase 1(HMGCS1)	Homo sapiens
1717	7-dehydrocholesterol reductase(DHCR7)	Homo sapiens
348	apolipoprotein E(APOE)	Homo sapiens
51478	hydroxysteroid 17-beta dehydrogenase 7(HSD17B7)	Homo sapiens
3638	insulin induced gene 1(INSIG1)	Homo sapiens
3422	isopentenyl-diphosphate delta isomerase 1(IDI1)	Homo sapiens
4047	lanosterol synthase (2,3-oxidosqualene-lanosterol cyclase)(LSS)	Homo sapiens
3949	low density lipoprotein receptor(LDLR)	Homo sapiens
6307	methylsterol monooxygenase 1(MSMO1)	Homo sapiens
4864	NPC intracellular cholesterol transporter 1(NPC1)	Homo sapiens
10577	NPC intracellular cholesterol transporter 2(NPC2)	Homo sapiens

Fatty acid biosynthesis/metabolism – enrichment score: 7.7

ID	Gene Name	Species
672	BRCA1, DNA repair associated(BRCA1)	Homo sapiens
39	acetyl-CoA acetyltransferase 2(ACAT2)	Homo sapiens
31	acetyl-CoA carboxylase alpha(ACACA)	Homo sapiens
3992	fatty acid desaturase 1(FADS1)	Homo sapiens
9415	fatty acid desaturase 2(FADS2)	Homo sapiens
2194	fatty acid synthase(FASN)	Homo sapiens
5730	prostaglandin D2 synthase(PTGDS)	Homo sapiens
6319	stearoyl-CoA desaturase(SCD)	Homo sapiens

Collagen fibril organization – enrichment score: 6.7

ID	Gene Name	Species
176	aggrecan(ACAN)	Homo sapiens
1311	cartilage oligomeric matrix protein(COMP)	Homo sapiens
1116	chitinase 3 like 1(CHI3L1)	Homo sapiens
10087	collagen type IV alpha 3 binding protein(COL4A3BP)	Homo sapiens
1293	collagen type VI alpha 3 chain(COL6A3)	Homo sapiens
1301	collagen type XI alpha 1 chain(COL11A1)	Homo sapiens
7373	collagen type XIV alpha 1 chain(COL14A1)	Homo sapiens
1306	collagen type XV alpha 1 chain(COL15A1)	Homo sapiens
3676	integrin subunit alpha 4(ITGA4)	Homo sapiens
284217	laminin subunit alpha 1(LAMA1)	Homo sapiens
3908	laminin subunit alpha 2(LAMA2)	Homo sapiens
4320	matrix metallopeptidase 11(MMP11)	Homo sapiens
4324	matrix metallopeptidase 15(MMP15)	Homo sapiens
6423	secreted frizzled related protein 2(SFRP2)	Homo sapiens
5184	peptidase D(PEPD)	Homo sapiens
10631	periostin(POSTN)	Homo sapiens
127435	podocan(PODN)	Homo sapiens
5549	proline and arginine rich end leucine rich repeat protein(PRELP)	Homo sapiens
10417	spondin 2(SPON2)	Homo sapiens
6382	syndecan 1(SDC1)	Homo sapiens
7058	thrombospondin 2(THBS2)	Homo sapiens
7059	thrombospondin 3(THBS3)	Homo sapiens

Cell response to starvation – enrichment score: 5.5

ID	Gene Name	Species
93664	calcium dependent secretion activator 2(CADPS2)	Homo sapiens
3992	fatty acid desaturase 1(FADS1)	Homo sapiens
96459	folliculin interacting protein 1(FNIP1)	Homo sapiens
2308	forkhead box O1(FOXO1)	Homo sapiens
255738	proprotein convertase subtilisin/kexin type 9(PCSK9)	Homo sapiens

Negative regulation of cell death – enrichment score: 3.8

ID	Gene Name	Species
3357	5-hydroxytryptamine receptor 2B(HTR2B)	Homo sapiens
4864	NPC intracellular cholesterol transporter 1(NPC1)	Homo sapiens
8839	WNT1 inducible signaling pathway protein 2(WISP2)	Homo sapiens
4856	nephroblastoma overexpressed(NOV)	Homo sapiens

Cell division – enrichment score: 3.3

ID	Gene Name	Species
701	BUB1 mitotic checkpoint serine/threonine kinase B(BUB1B)	Homo sapiens
699	BUB1 mitotic checkpoint serine/threonine kinase(BUB1)	Homo sapiens
4751	NIMA related kinase 2(NEK2)	Homo sapiens
259266	abnormal spindle microtubule assembly(ASPM)	Homo sapiens
54443	anillin actin binding protein(ANLN)	Homo sapiens
9212	aurora kinase B(AURKB)	Homo sapiens
991	cell division cycle 20(CDC20)	Homo sapiens
55143	cell division cycle associated 8(CDCA8)	Homo sapiens
1063	centromere protein F(CENPF)	Homo sapiens
55165	centrosomal protein 55(CEP55)	Homo sapiens
890	cyclin A2(CCNA2)	Homo sapiens
983	cyclin dependent kinase 1(CDK1)	Homo sapiens
81610	family with sequence similarity 83 member D(FAM83D)	Homo sapiens
55137	fidgetin, microtubule severing factor(FIGN)	Homo sapiens
11004	kinesin family member 2C(KIF2C)	Homo sapiens
3833	kinesin family member C1(KIFC1)	Homo sapiens
64151	non-SMC condensin I complex subunit G(NCAPG)	Homo sapiens
51203	nucleolar and spindle associated protein 1(NUSAP1)	Homo sapiens
5347	polo like kinase 1(PLK1)	Homo sapiens
5581	protein kinase C epsilon(PRKCE)	Homo sapiens
51429	sorting nexin 9(SNX9)	Homo sapiens
23503	zinc finger FYVE-type containing 26(ZFYVE26)	Homo sapiens

Apoptotic process – enrichment score: 2.3

ID	Gene Name	Species
1718	24-dehydrocholesterol reductase(DHCR24)	Homo sapiens
672	BRCA1, DNA repair associated(BRCA1)	Homo sapiens
701	BUB1 mitotic checkpoint serine/threonine kinase B(BUB1B)	Homo sapiens
699	BUB1 mitotic checkpoint serine/threonine kinase(BUB1)	Homo sapiens
90427	Bcl2 modifying factor(BMF)	Homo sapiens
220042	DNA damage induced apoptosis suppressor(DDIAS)	Homo sapiens
55332	DNA damage regulated autophagy modulator 1(DRAM1)	Homo sapiens
2122	MDS1 and EVI1 complex locus(MECOM)	Homo sapiens
1311	cartilage oligomeric matrix protein(COMP)	Homo sapiens
834	caspase 1(CASP1)	Homo sapiens
837	caspase 4(CASP4)	Homo sapiens
23705	cell adhesion molecule 1(CADM1)	Homo sapiens
1116	chitinase 3 like 1(CHI3L1)	Homo sapiens

983	cyclin dependent kinase 1(CDK1)	Homo sapiens
23604	death associated protein kinase 2(DAPK2)	Homo sapiens
2308	forkhead box O1(FOXO1)	Homo sapiens
3092	huntingtin interacting protein 1(HIP1)	Homo sapiens
9833	maternal embryonic leucine zipper kinase(MELK)	Homo sapiens
4171	minichromosome maintenance complex component 2(MCM2)	Homo sapiens
79156	pleckstrin homology and FYVE domain containing 1(PLEKHF1)	Homo sapiens
255738	proprotein convertase subtilisin/kexin type 9(PCSK9)	Homo sapiens
5581	protein kinase C epsilon(PRKCE)	Homo sapiens
6423	secreted frizzled related protein 2(SFRP2)	Homo sapiens
8878	sequestosome 1(SQSTM1)	Homo sapiens
23213	sulfatase 1(SULF1)	Homo sapiens

Oxidation-reduction process – enrichment score: 2.0

ID	Gene Name	Species
1718	24-dehydrocholesterol reductase(DHCR24)	Homo sapiens
3156	3-hydroxy-3-methylglutaryl-CoA reductase(HMGCR)	Homo sapiens
1717	7-dehydrocholesterol reductase(DHCR7)	Homo sapiens
10257	ATP binding cassette subfamily C member 4(ABCC4)	Homo sapiens
80020	FAD dependent oxidoreductase domain containing (FOXRED2)	Homo sapiens
606495	cytochrome b5 reductase like(CYB5RL)	Homo sapiens
3992	fatty acid desaturase 1(FADS1)	Homo sapiens
9415	fatty acid desaturase 2(FADS2)	Homo sapiens
2194	fatty acid synthase(FASN)	Homo sapiens
391059	ferric chelate reductase 1(FRRS1)	Homo sapiens
2495	ferritin heavy chain 1(FTH1)	Homo sapiens
2328	flavin containing monooxygenase 3(FMO3)	Homo sapiens
2329	flavin containing monooxygenase 4(FMO4)	Homo sapiens
3290	hydroxysteroid 11-beta dehydrogenase 1(HSD11B1)	Homo sapiens
51171	hydroxysteroid 17-beta dehydrogenase 14(HSD17B14)	Homo sapiens
51478	hydroxysteroid 17-beta dehydrogenase 7(HSD17B7)	Homo sapiens
4522	methylenetetrahydrofolate dehydrogenase, cyclohydrolase and formyltetrahydrofolate synthetase 1(MTHFD1)	Homo sapiens
6307	methylsterol monooxygenase 1(MSMO1)	Homo sapiens
29948	oxidative stress induced growth inhibitor 1(OSGIN1)	Homo sapiens
6319	stearoyl-CoA desaturase(SCD)	Homo sapiens
6648	superoxide dismutase 2, mitochondrial(SOD2)	Homo sapiens
6649	superoxide dismutase 3, extracellular(SOD3)	Homo sapiens
10493	vesicle amine transport 1(VAT1)	Homo sapiens
